# Supplementary material for: Iron-based nanocatalyst for the acceptorless dehydrogenation reactions
Source: Nat Commun. 2017 Dec 15;8:2147. doi: 10.1038/s41467-017-01603-3 (PMC5732290; doi:10.1038/s41467-017-01603-3)
Supplement: Supplementary file 1 — Description of Additional Supplementary Files [file 41467_2017_1603_MOESM1_ESM.pdf]

Description of Additional Supplementary Files

File Name: Supplementary Movie 1
Description: Hydrogen generation Video file
