# Supplementary material for: Iron-based nanocatalyst for the acceptorless dehydrogenation reactions
Source: Nat Commun. 2017 Dec 15;8:2147. doi: 10.1038/s41467-017-01603-3 (PMC5732290; doi:10.1038/s41467-017-01603-3)
Supplement: Supplementary file 2 — Supplementary Information [file 41467_2017_1603_MOESM2_ESM.pdf]

Supplementary Figures:

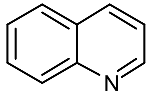

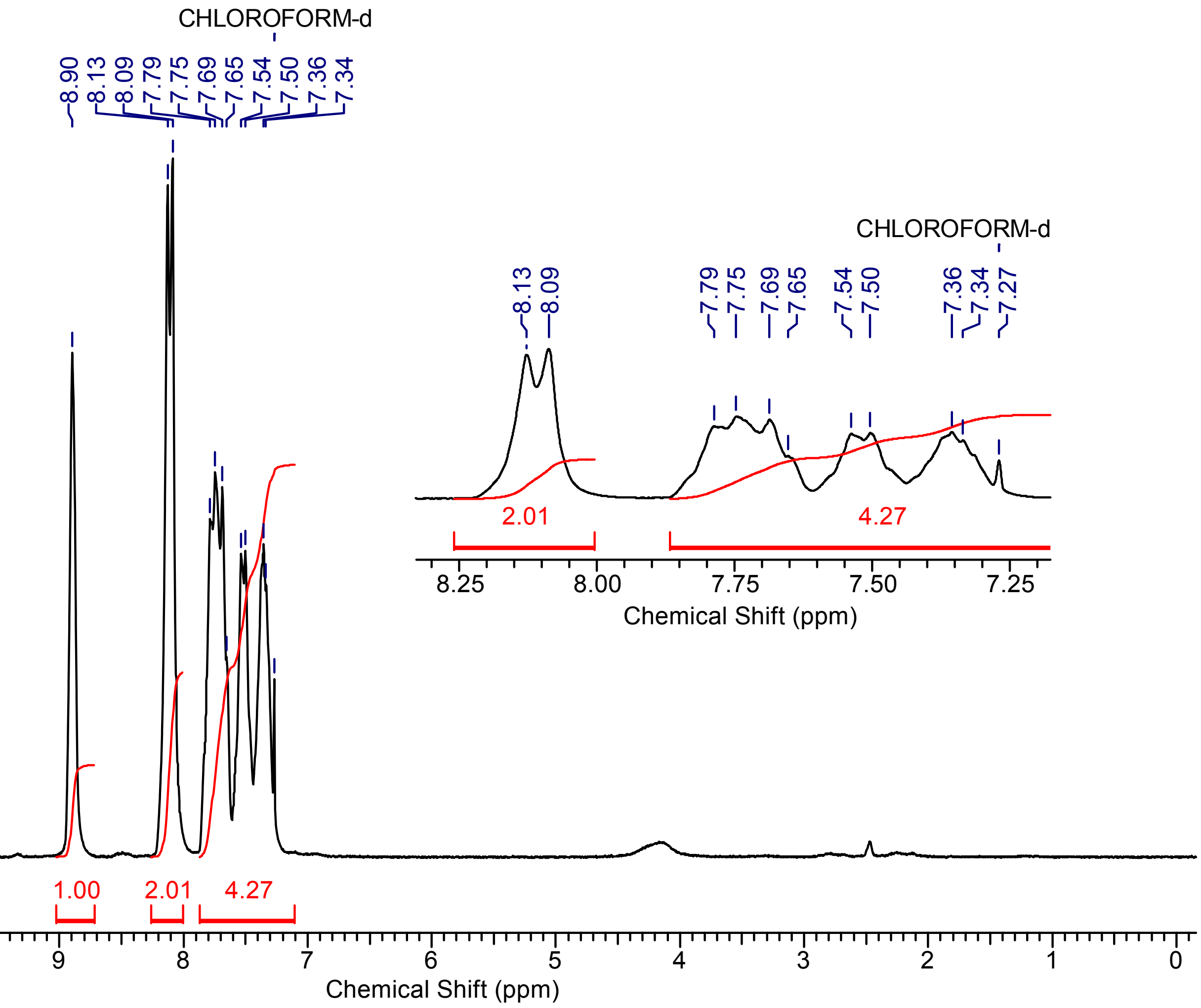

Supplementary Figure 1. ^{1}H NMR of 2a

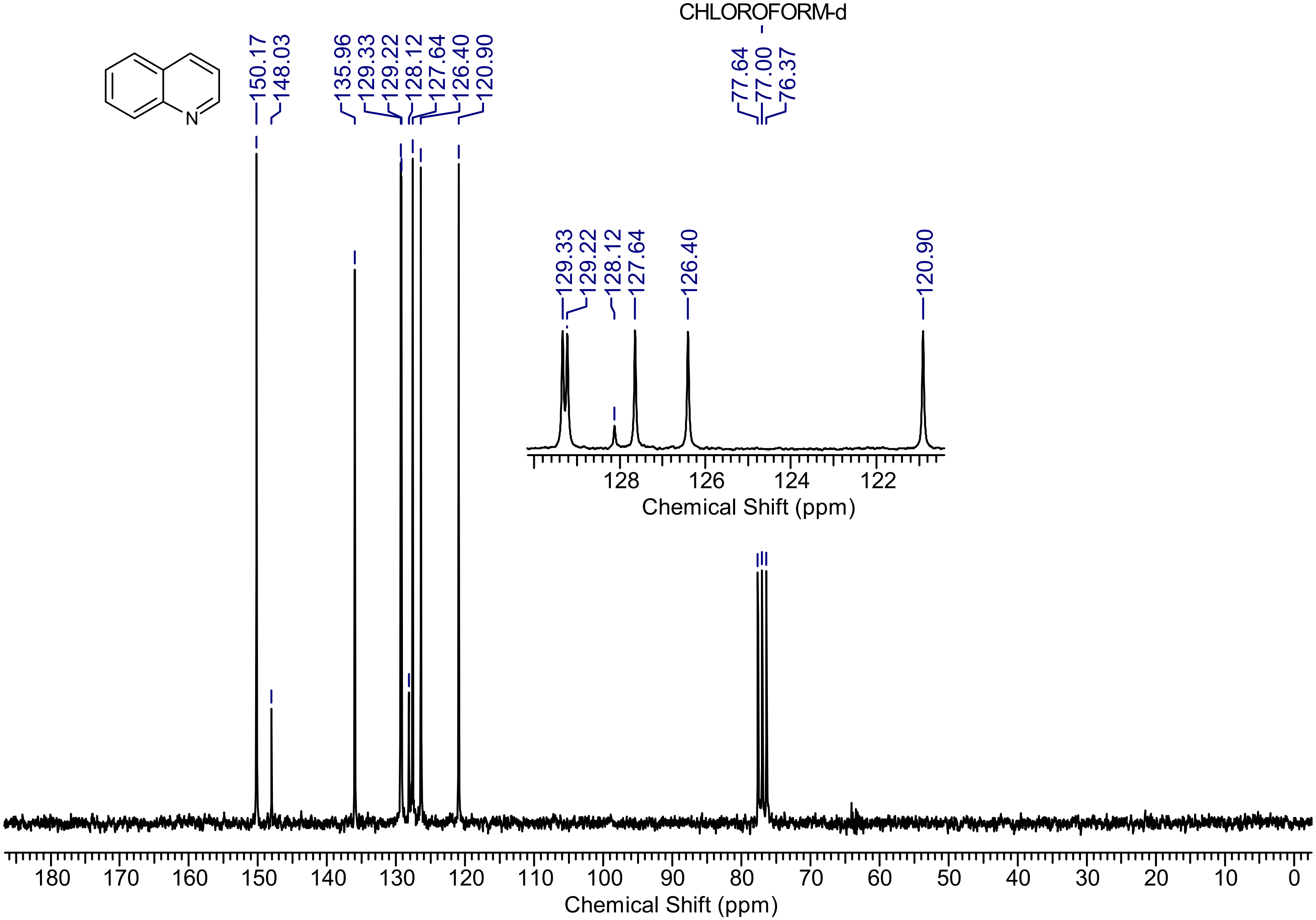

Supplementary Figure 2. ^{13}C NMR of 2a

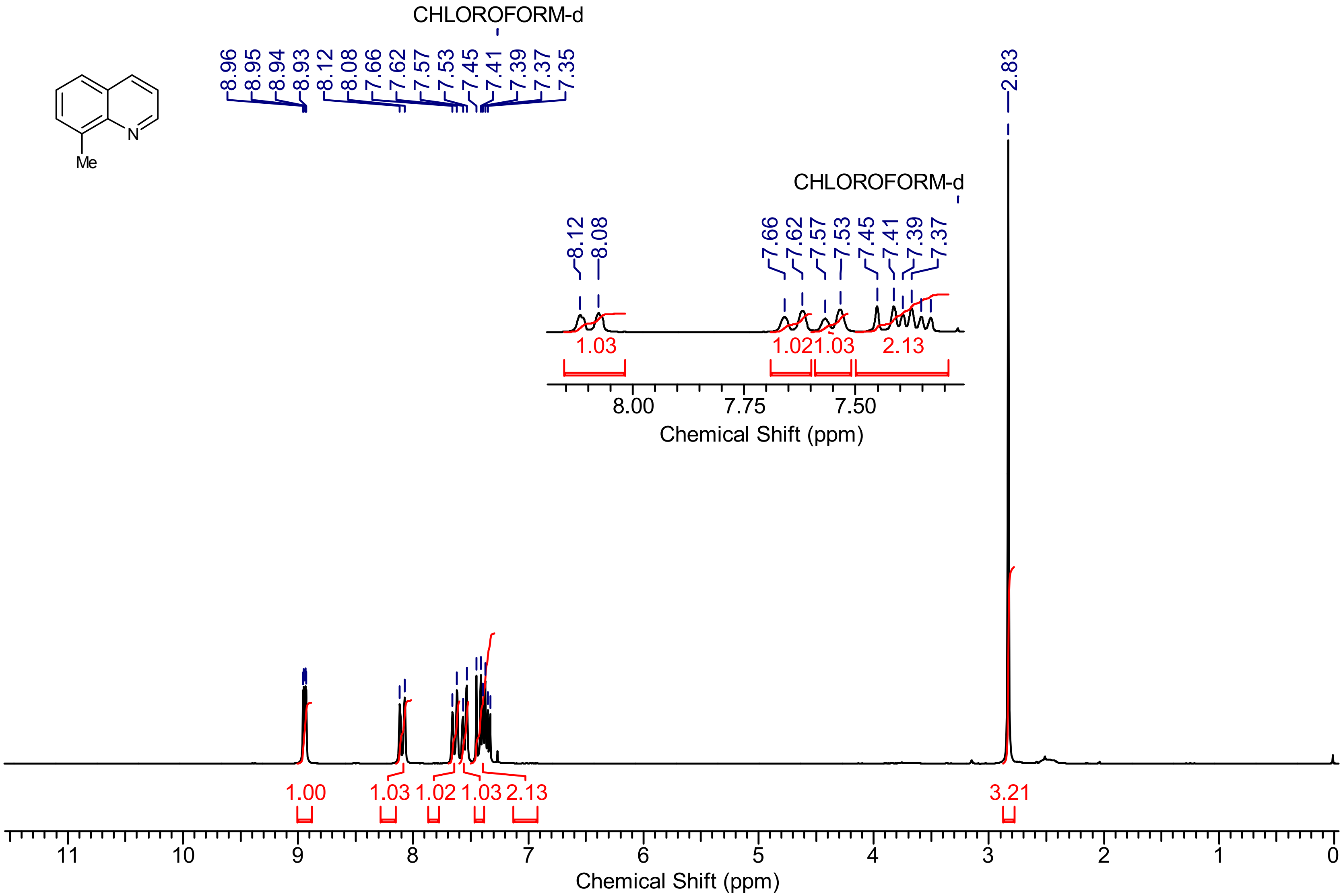

Supplementary Figure 3. ^{1}H NMR of 2b

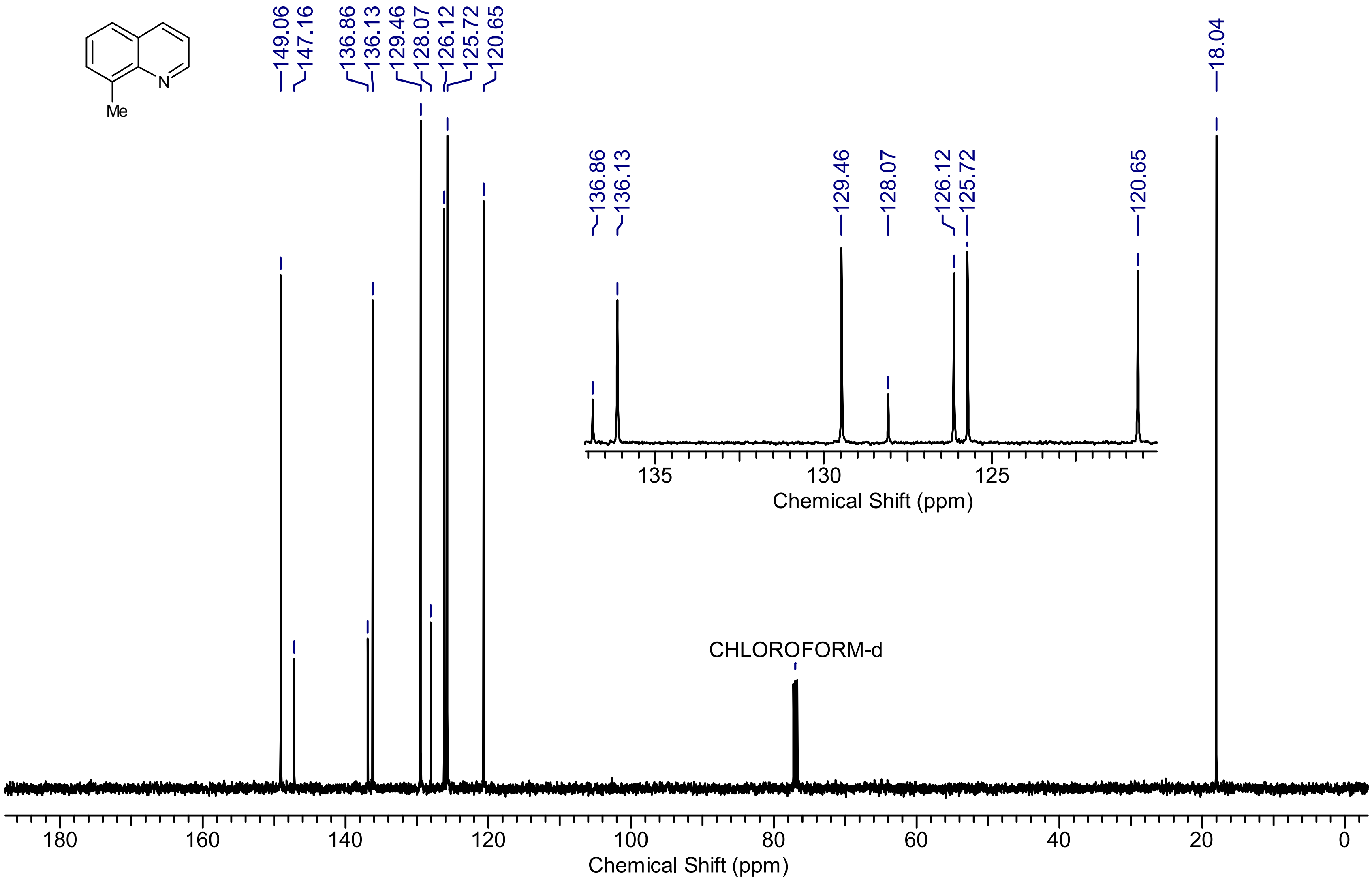

Supplementary Figure 4. ^{13}C NMR of 2b

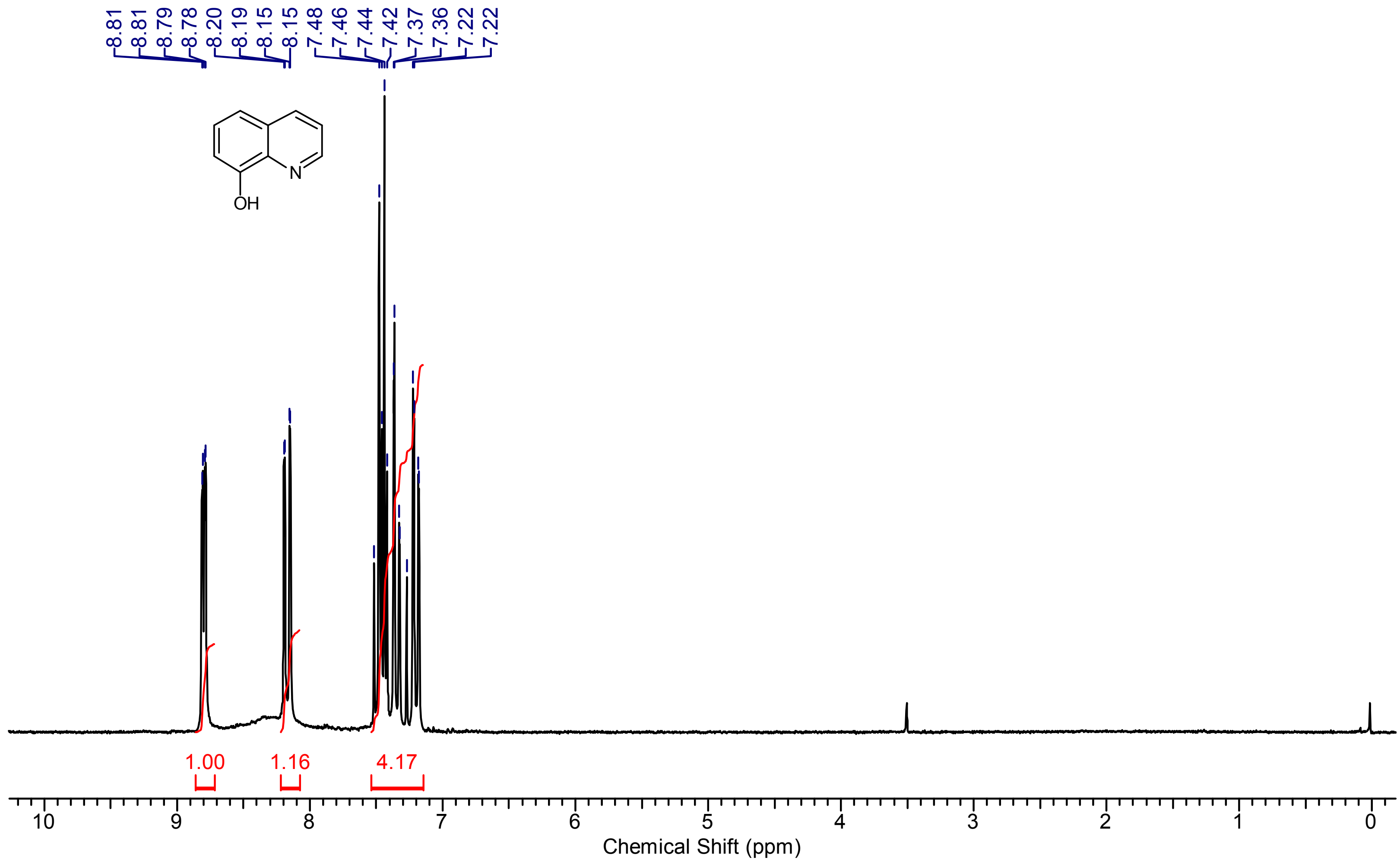

Supplementary Figure 5. ^{1}H NMR of 2d

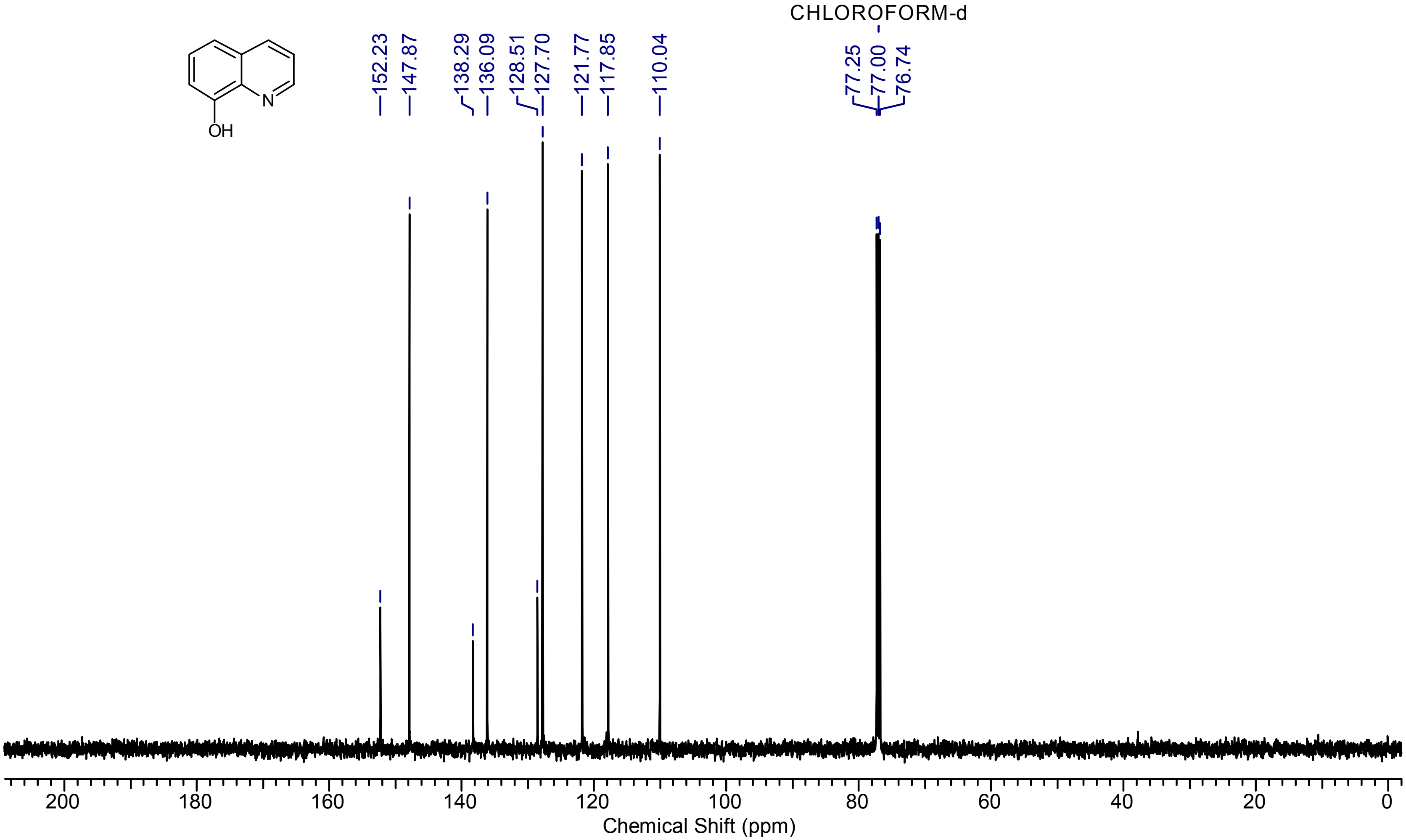

Supplementary Figure 6. ^{13}C NMR of 2d

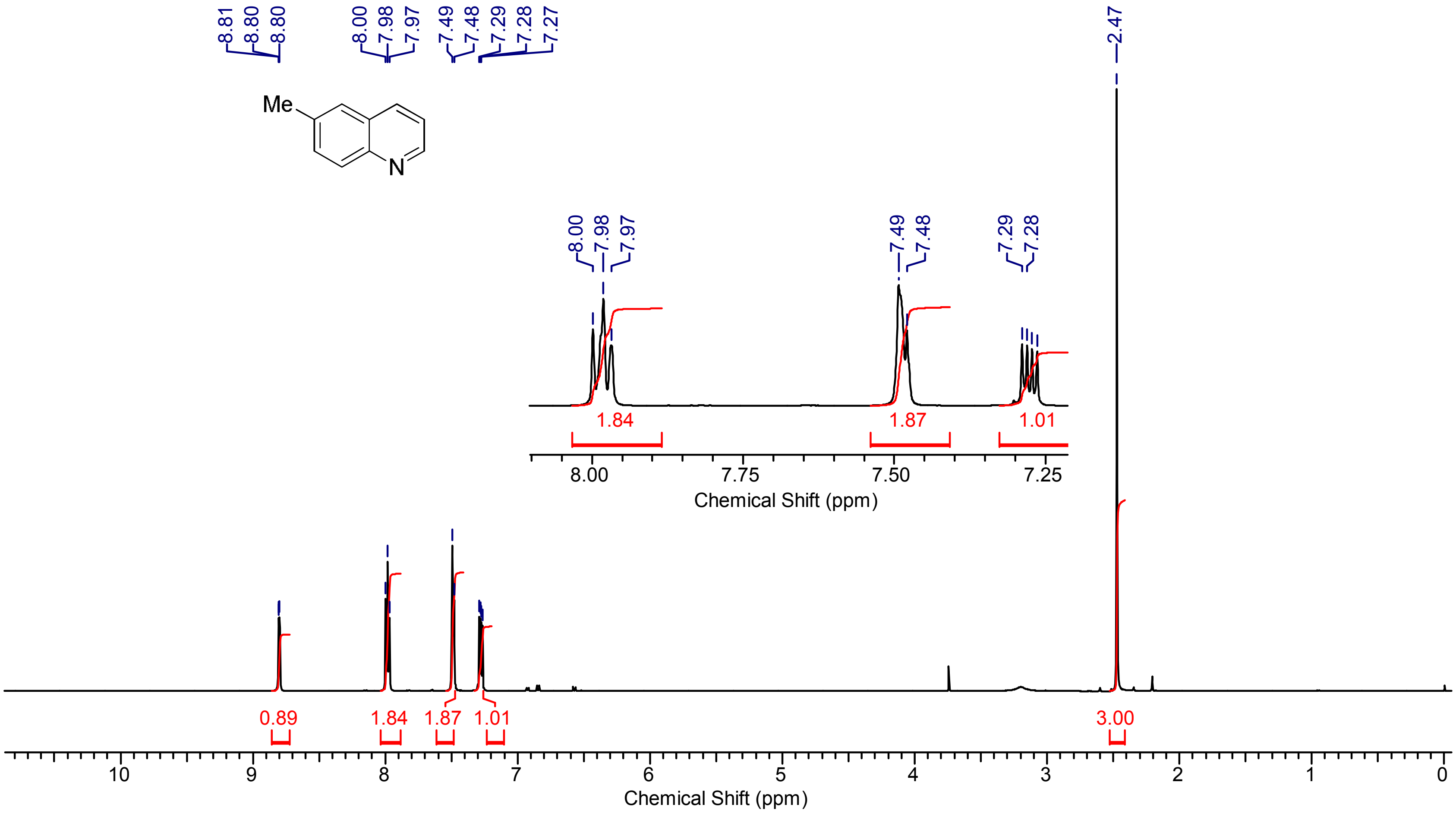

Supplementary Figure 7. ^{1}H NMR of 2e

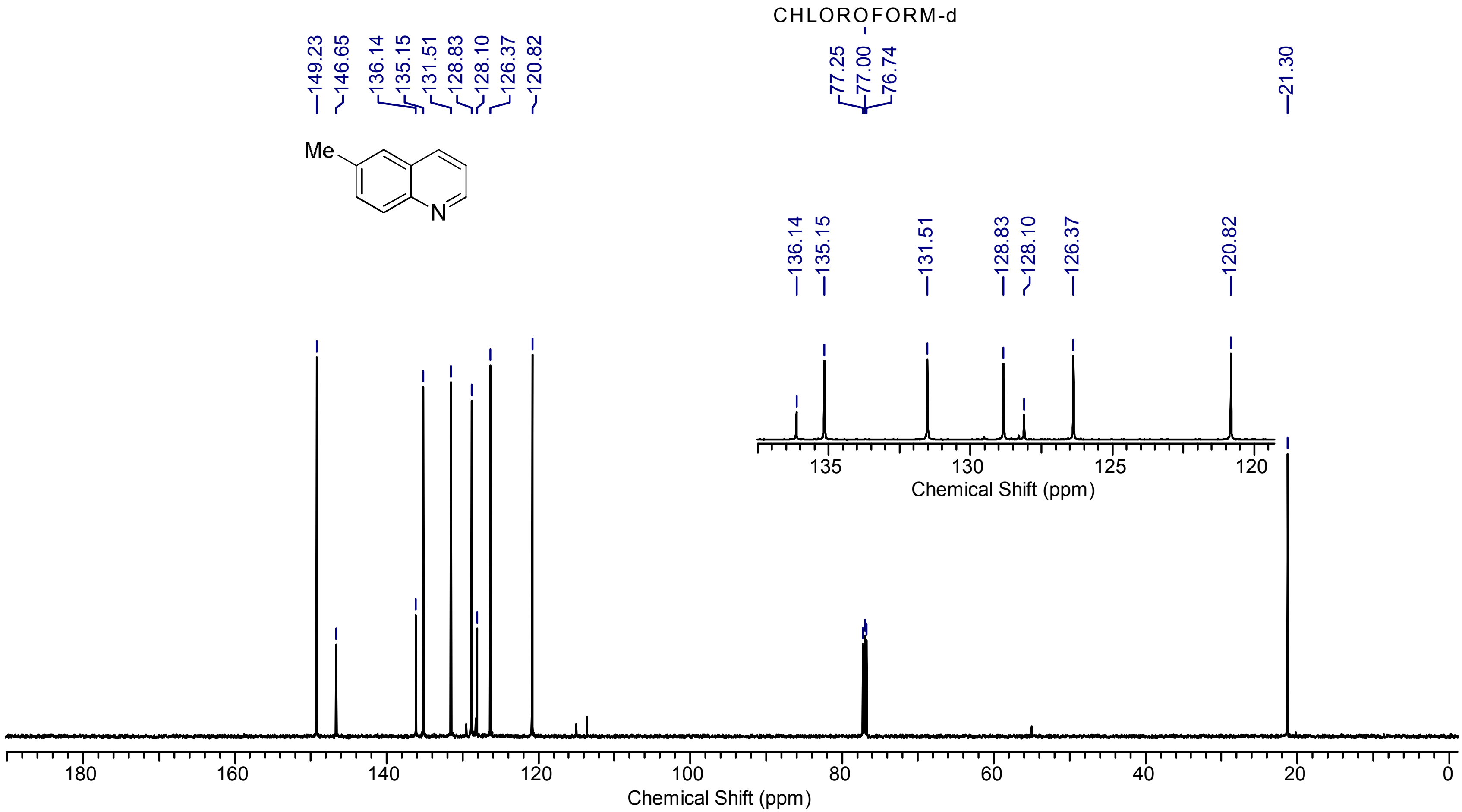

Supplementary Figure 8. ^{13}C NMR of 2e

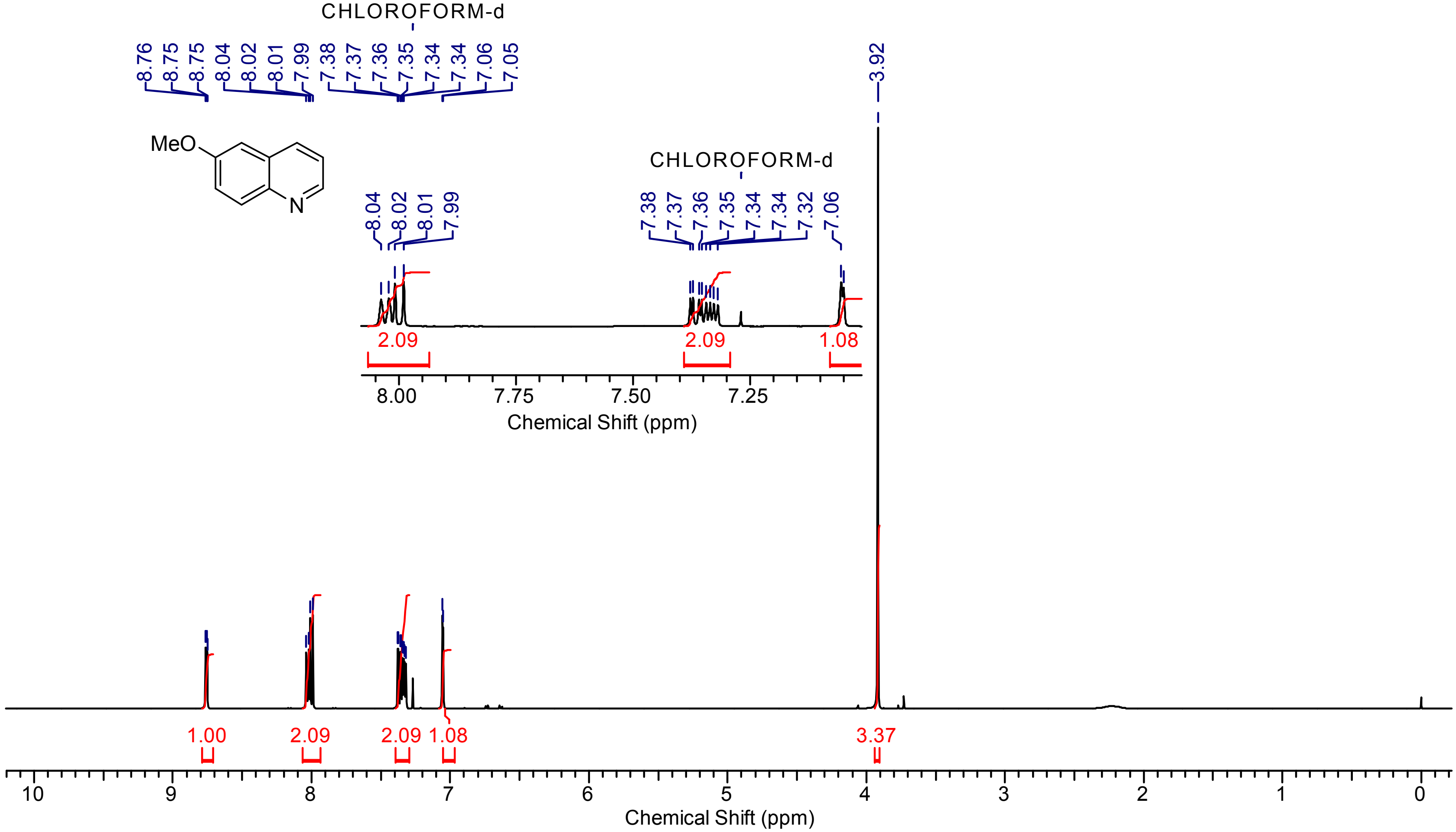

Supplementary Figure 9. ^{1}H NMR of 2f

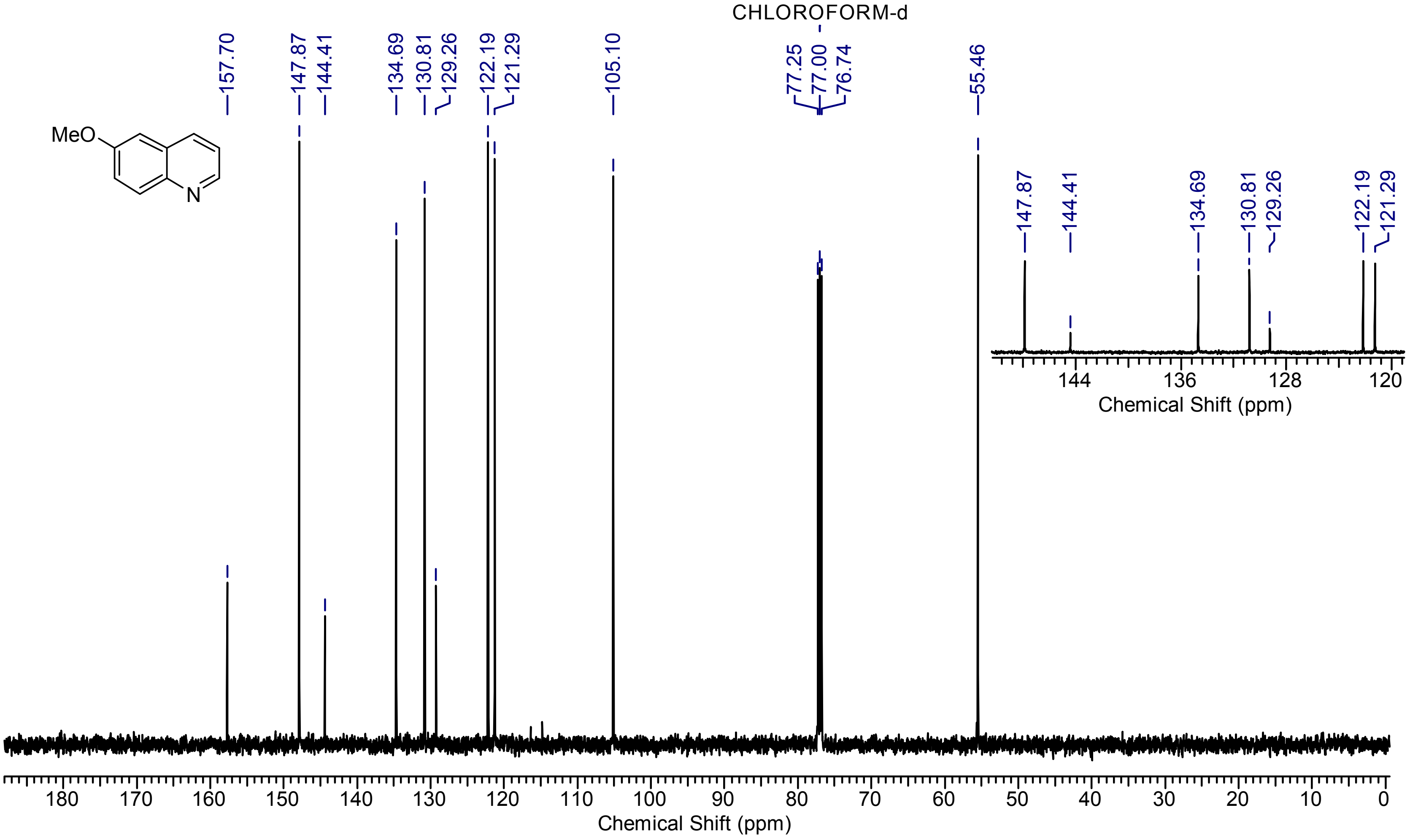

Supplementary Figure 10. ^{13}**CNMR of 2f**

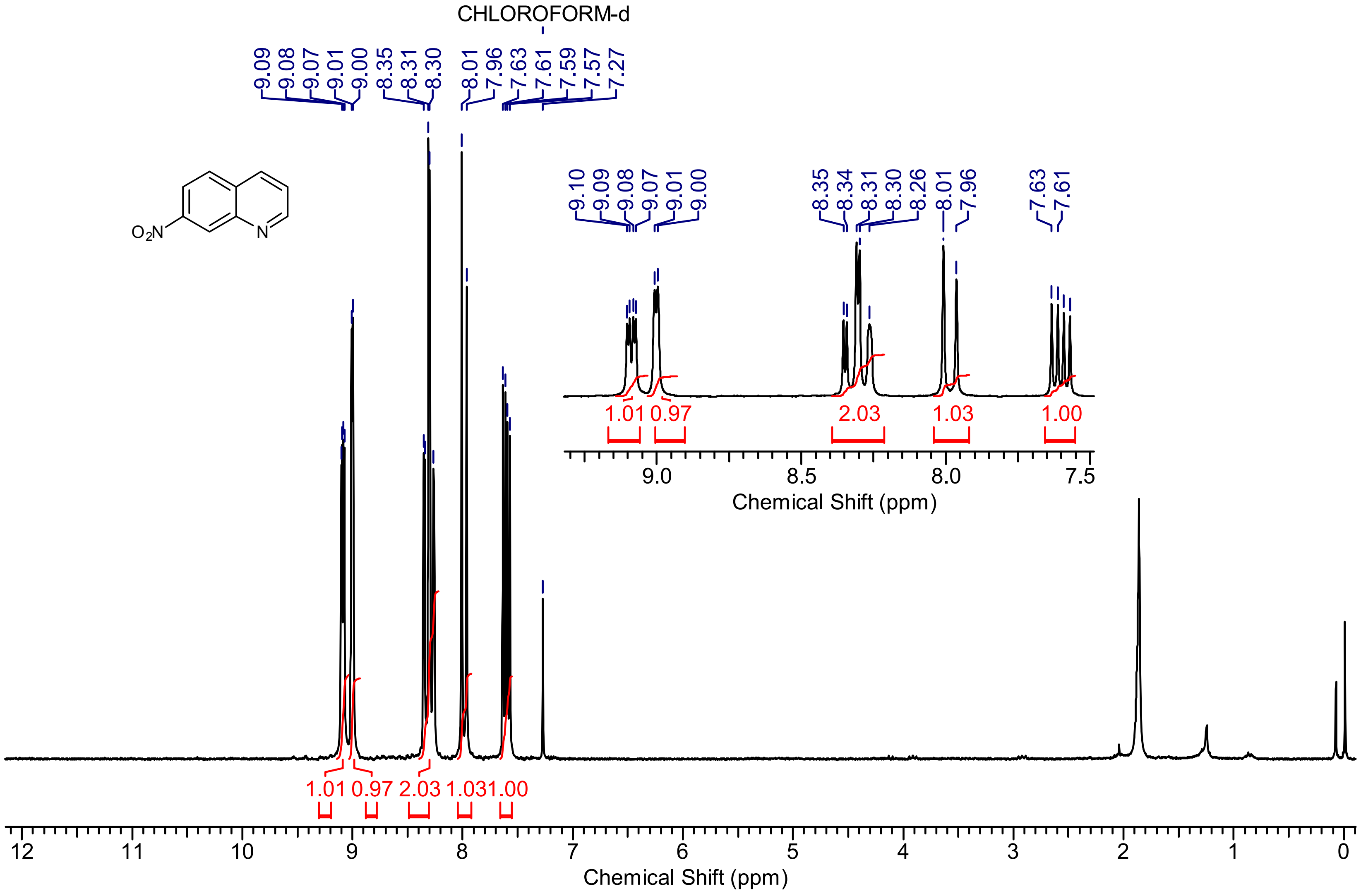

Supplementary Figure 11. ^{1}**H NMR of 2g**

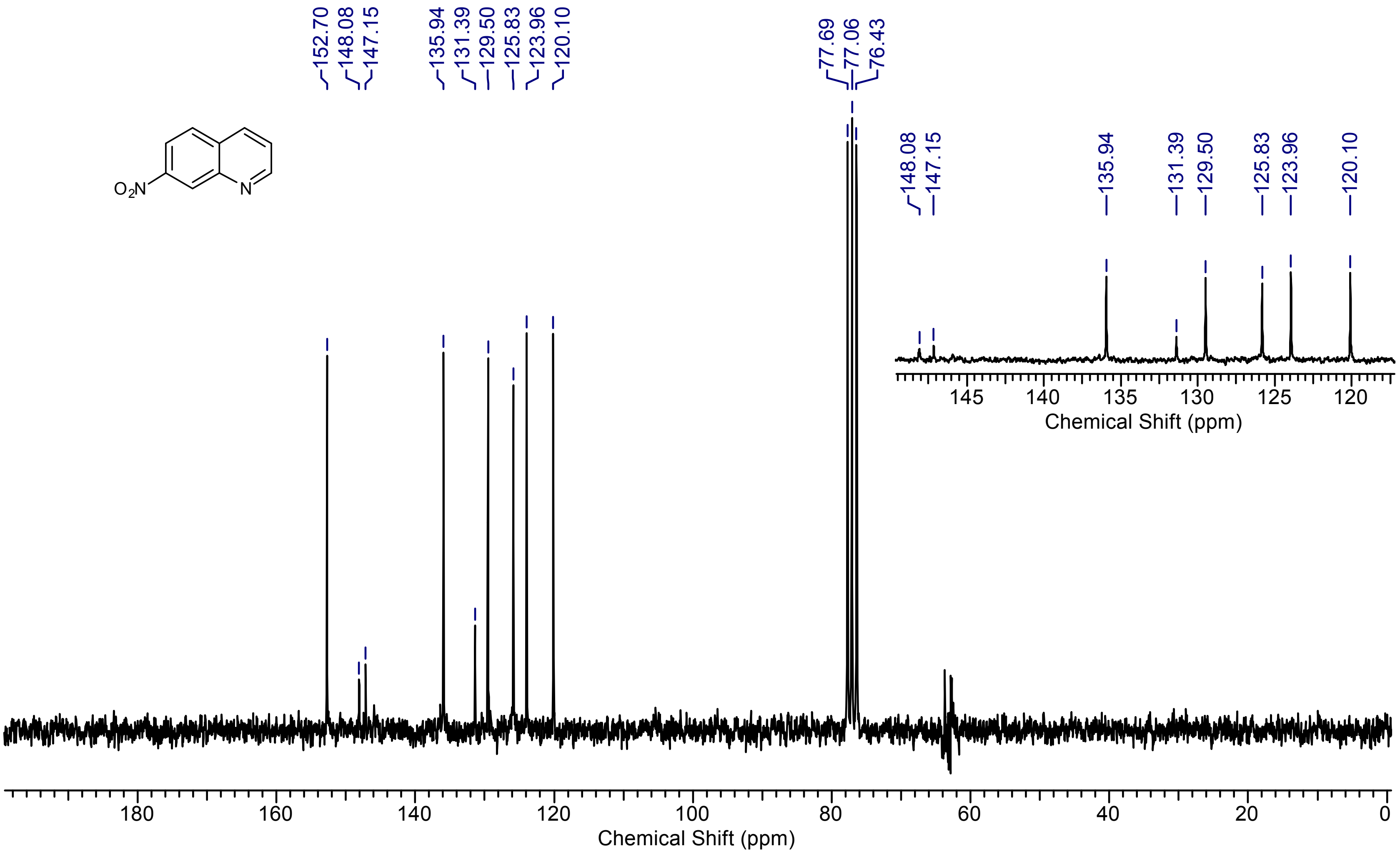

Supplementary Figure 12. ^{13}C NMR of 2g

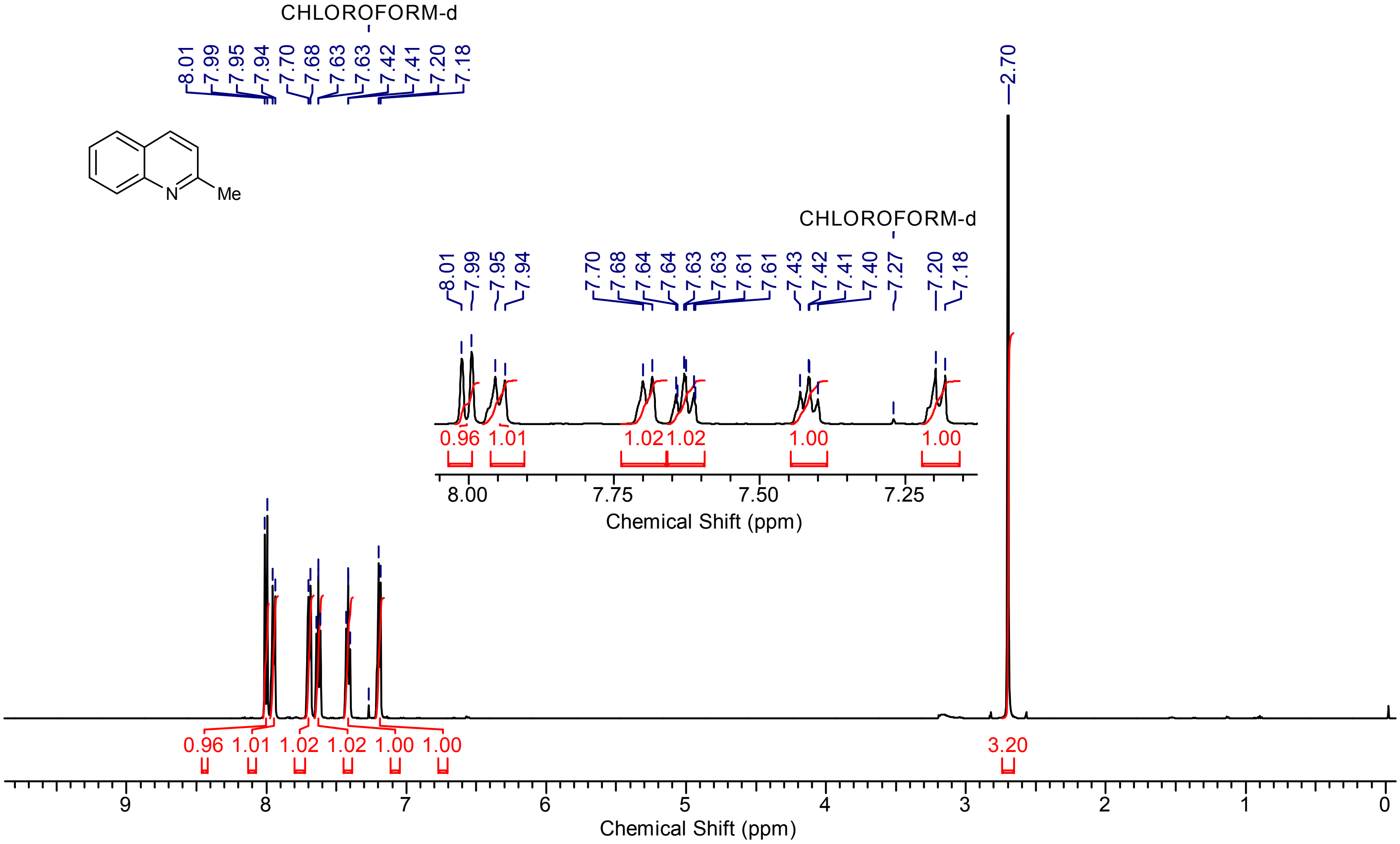

Supplementary Figure 13. ^{1}H NMR of 2h

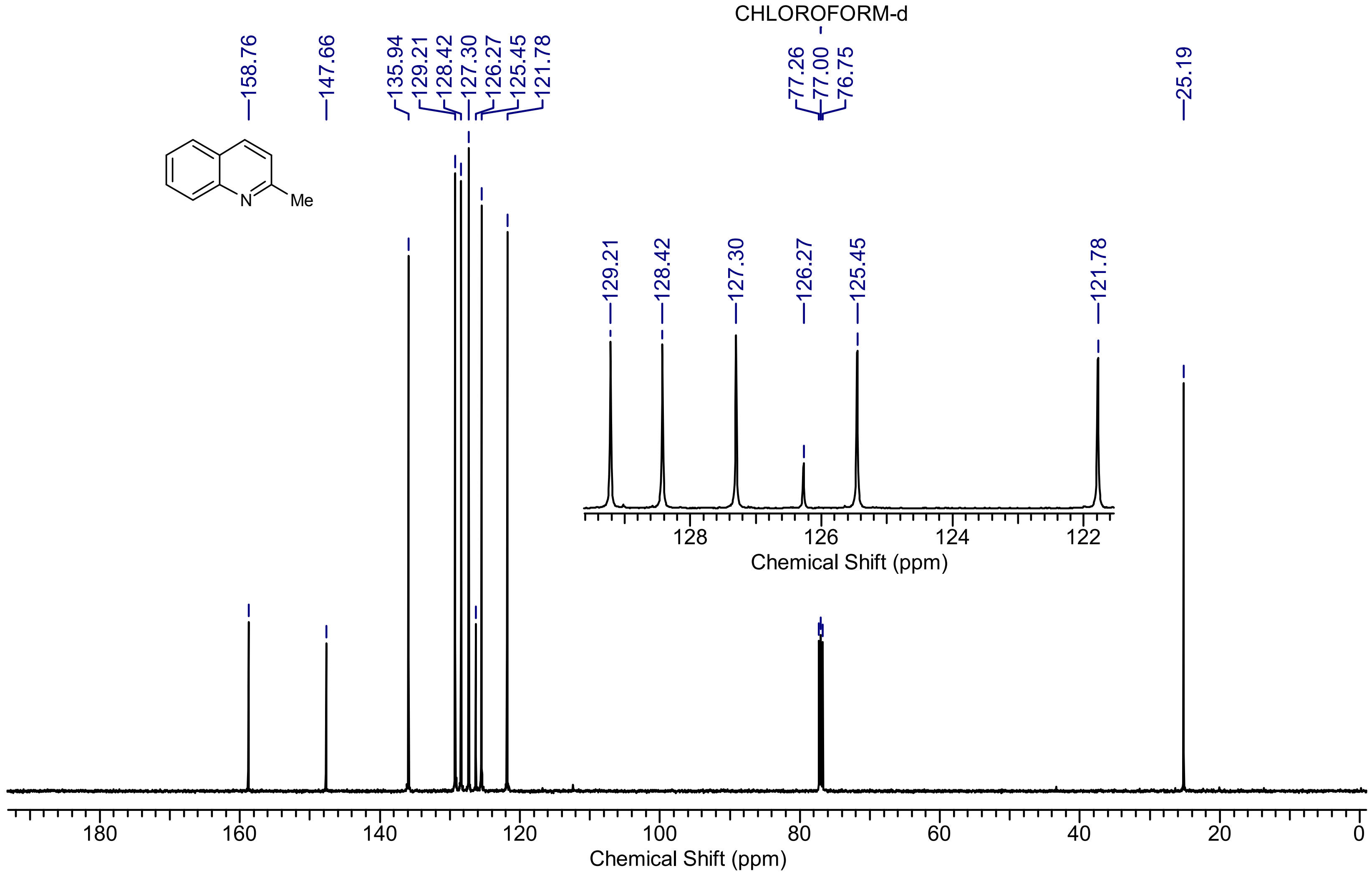

Supplementary Figure 14. ^{13}**C NMR of 2h**

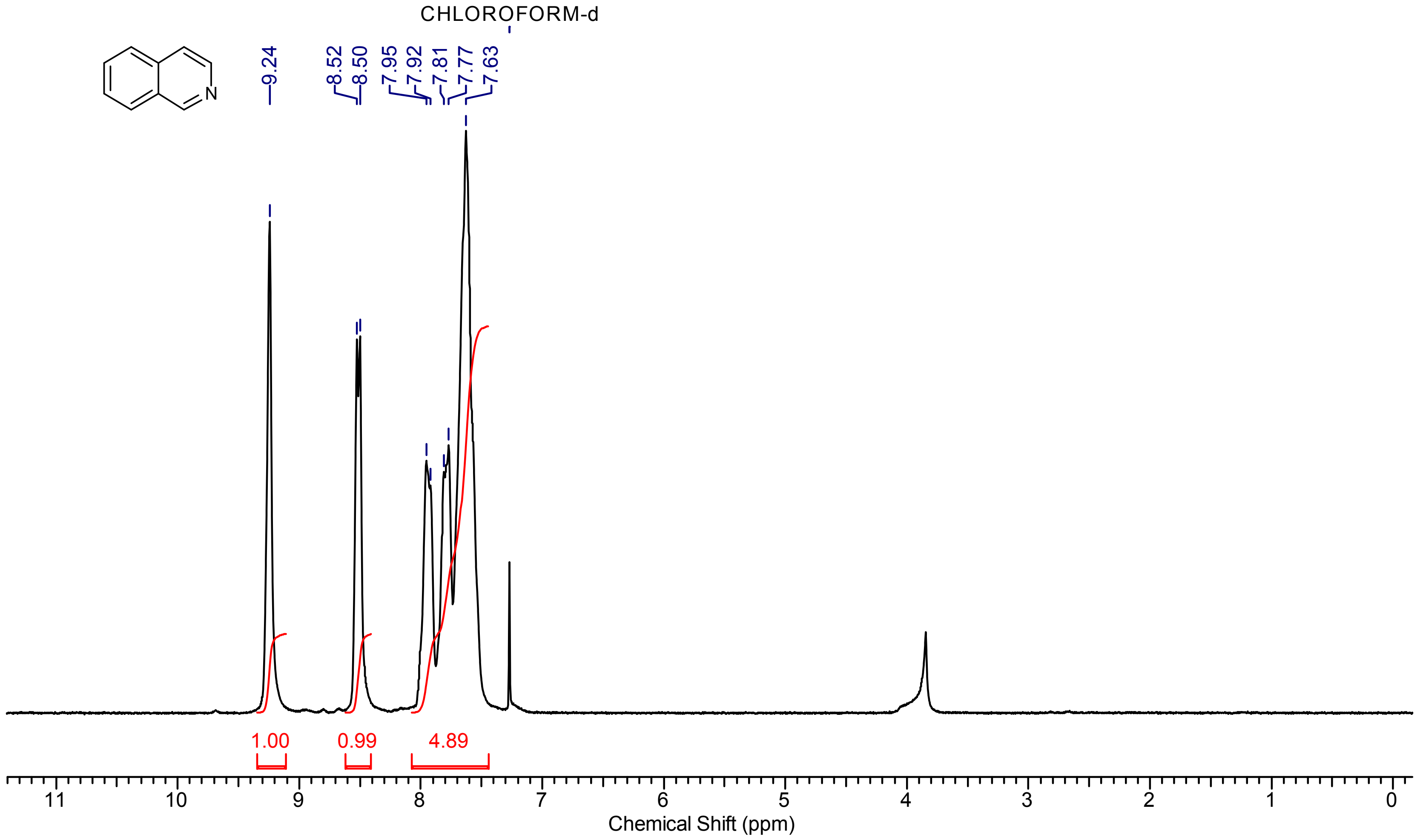

Supplementary Figure 15. ^{1}H NMR of 2i

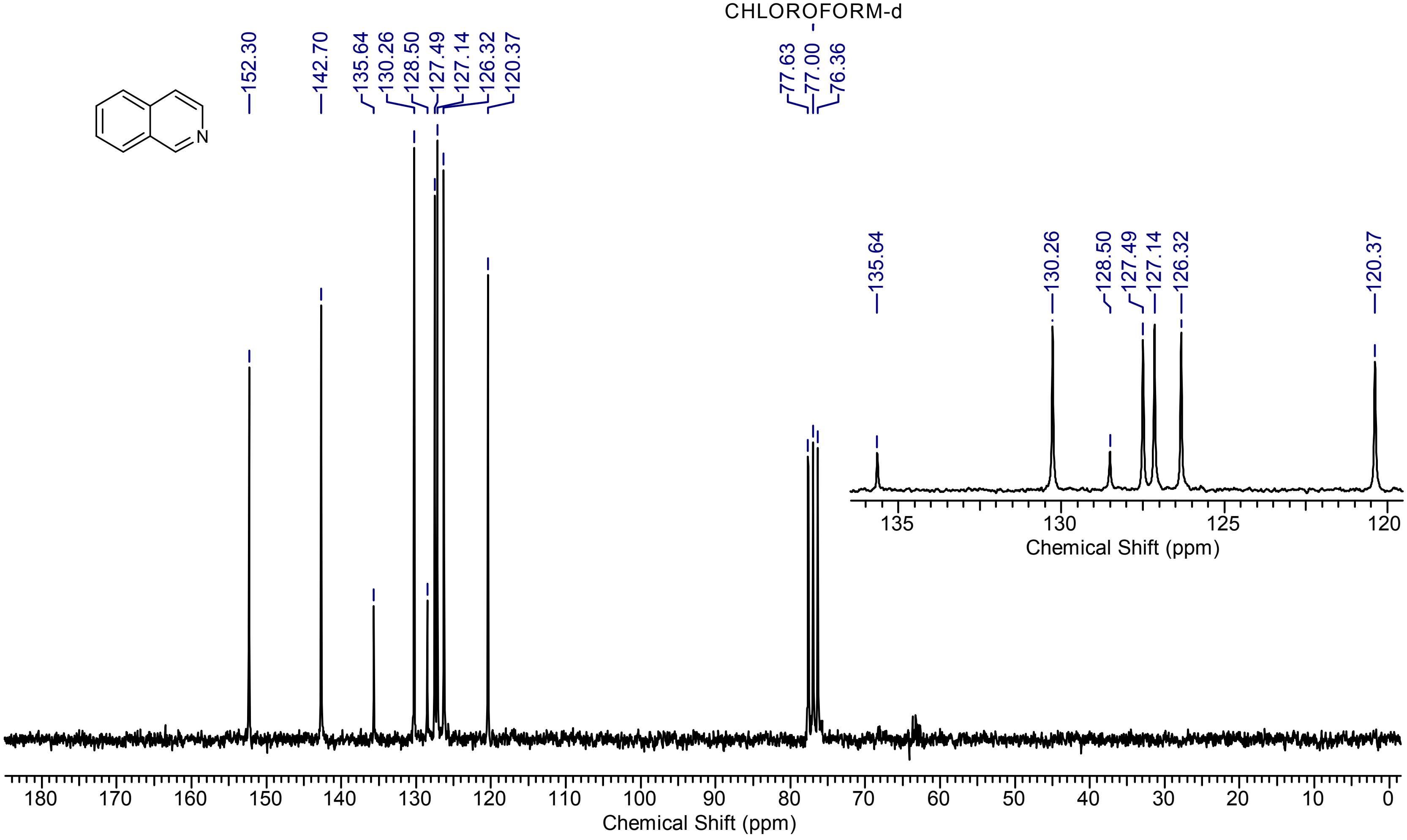

Supplementary Figure 16. ^{13}**C NMR of 2i**

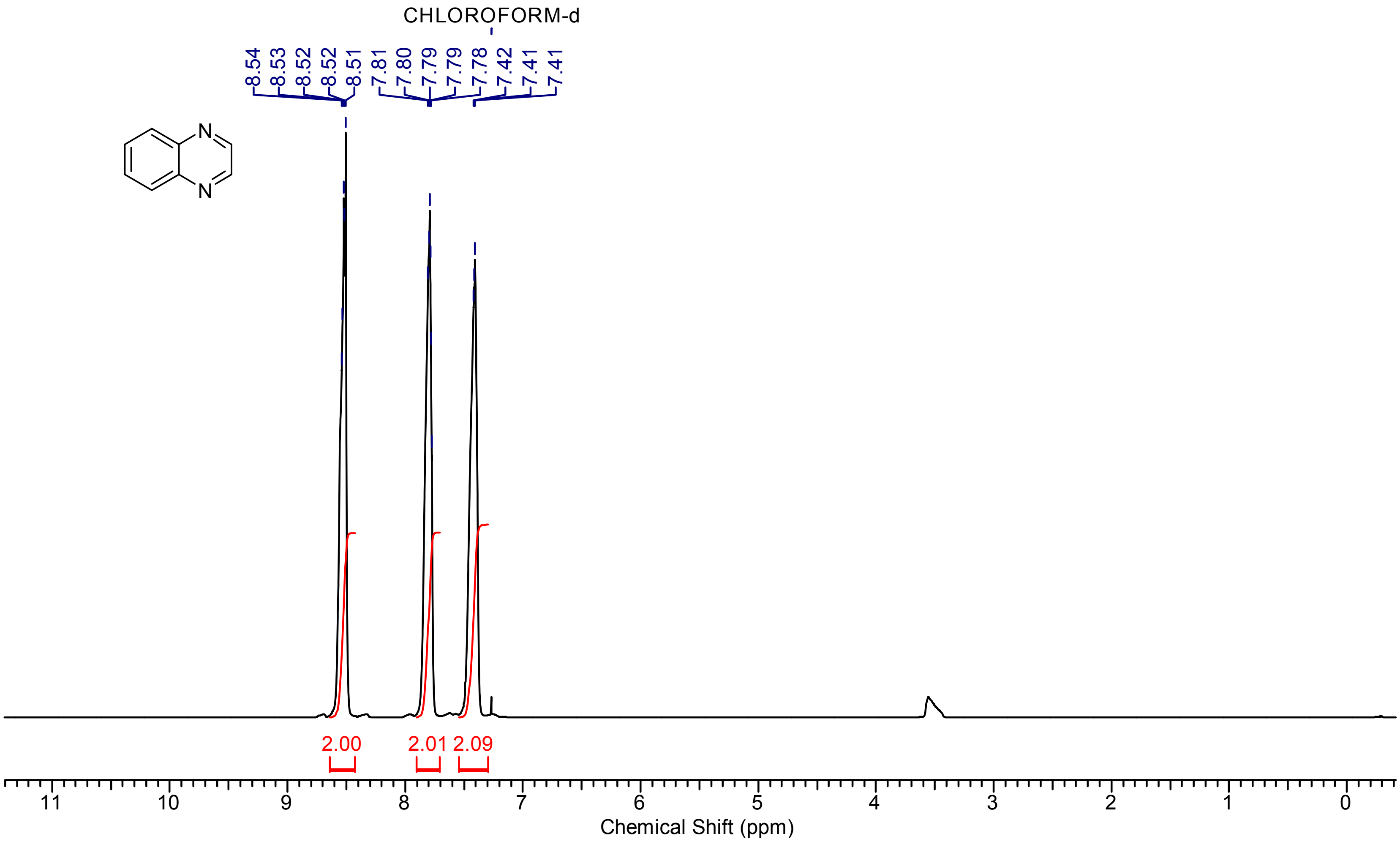

Supplementary Figure 17. ^{1}H NMR of 2j

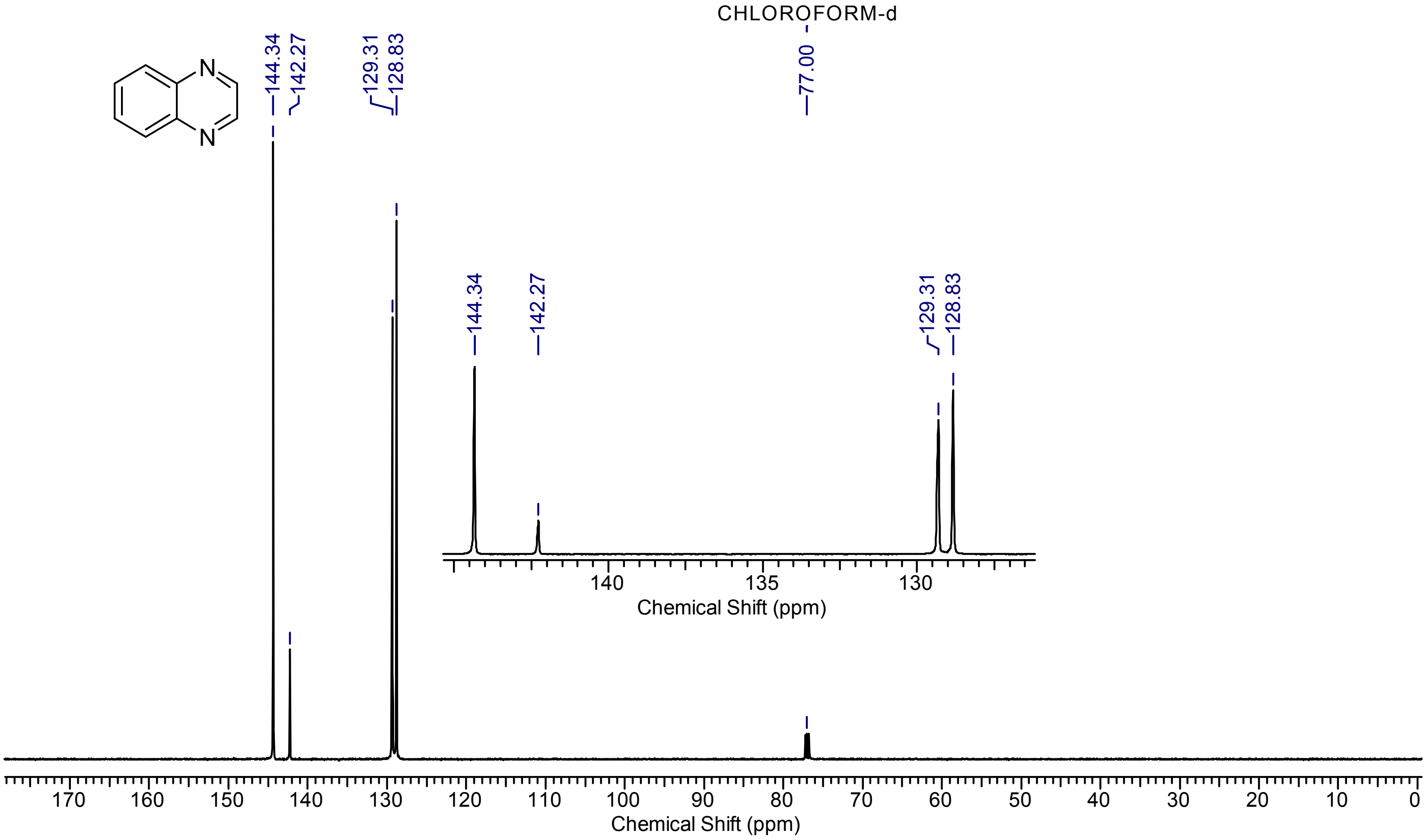

Supplementary Figure 18. ^{13}**C NMR of 2j**

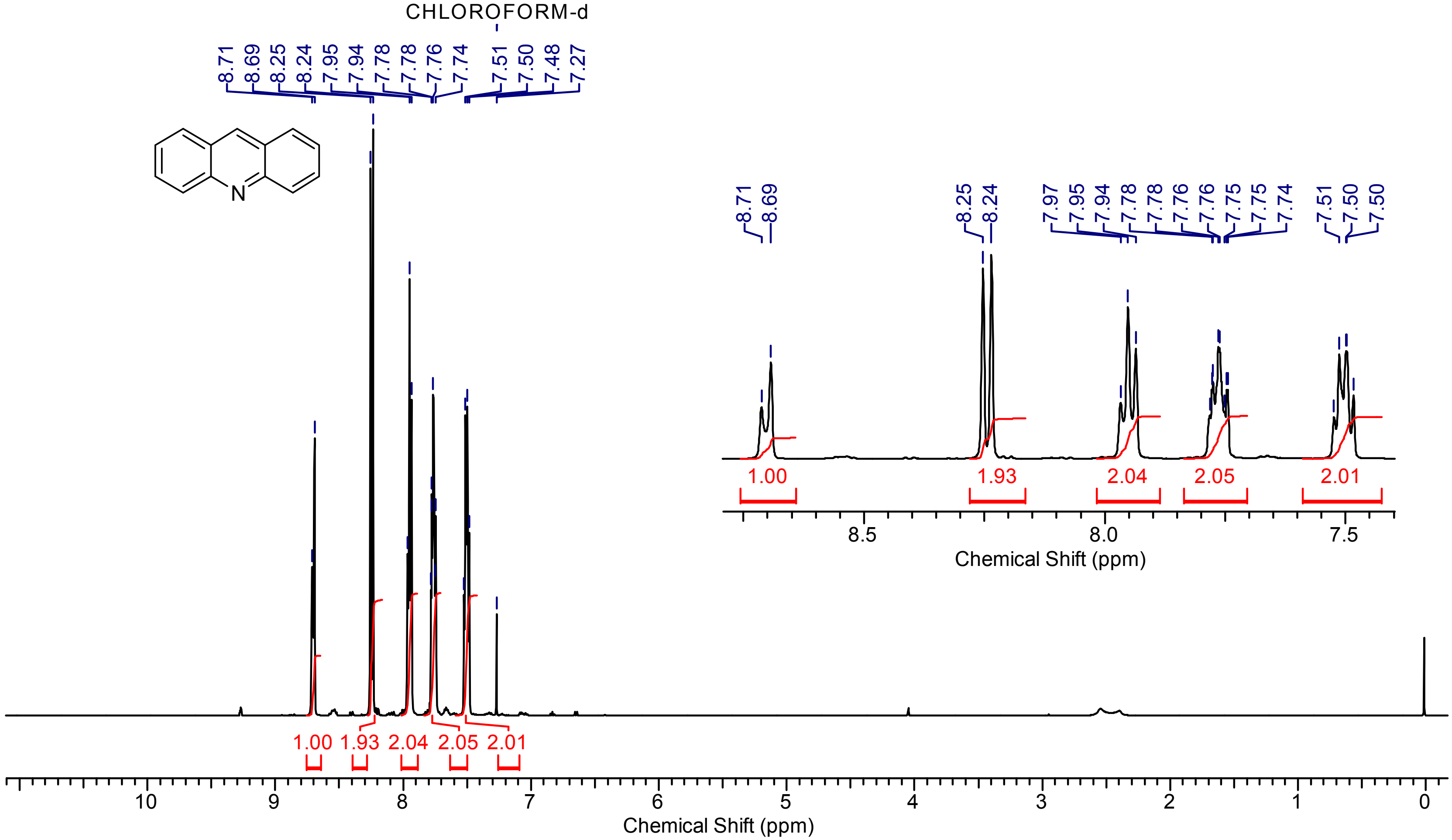

Supplementary Figure 19. ^{1}**H NMR of 2k**

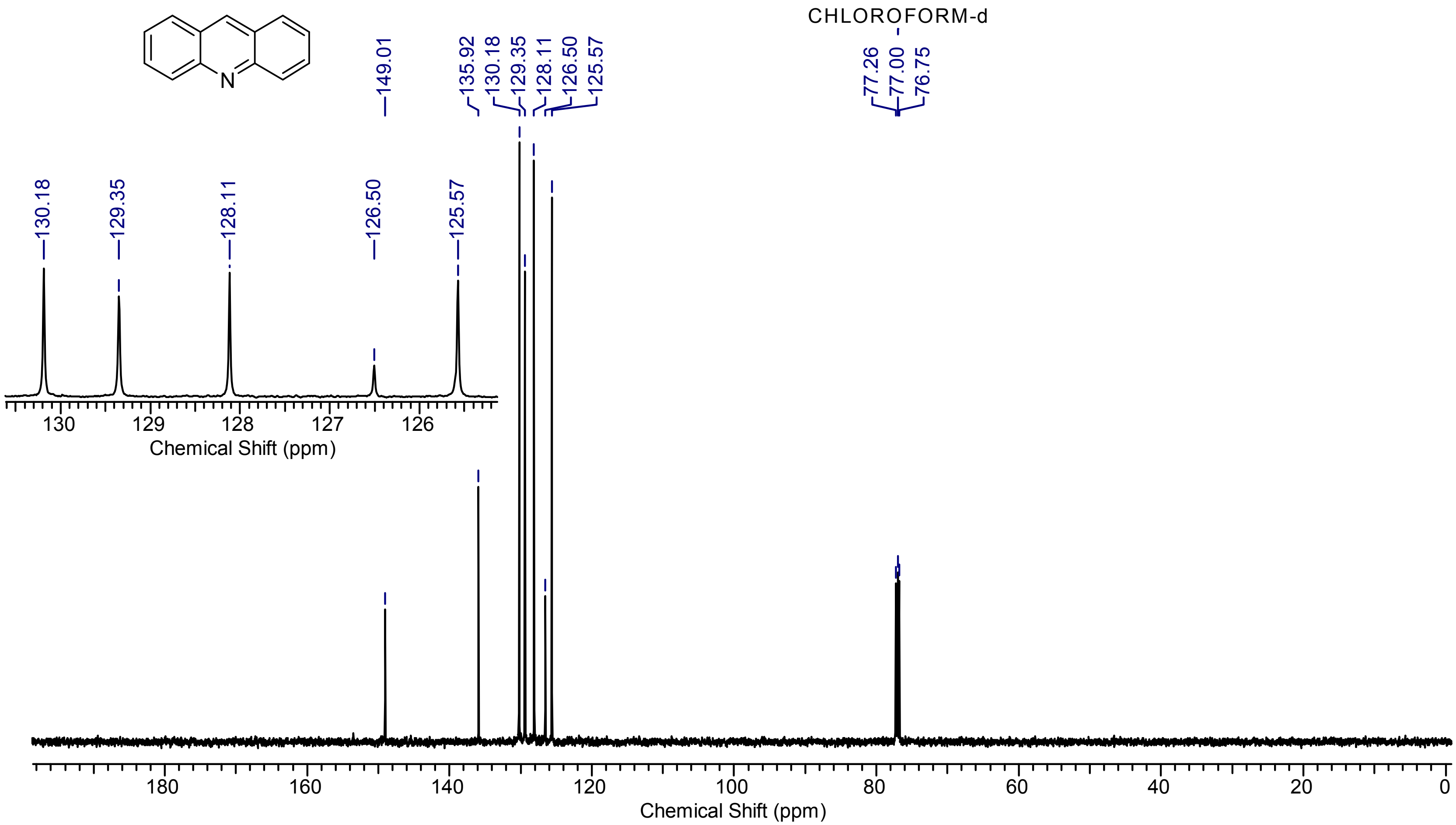

Supplementary Figure 20.^{13}C NMR of 2k

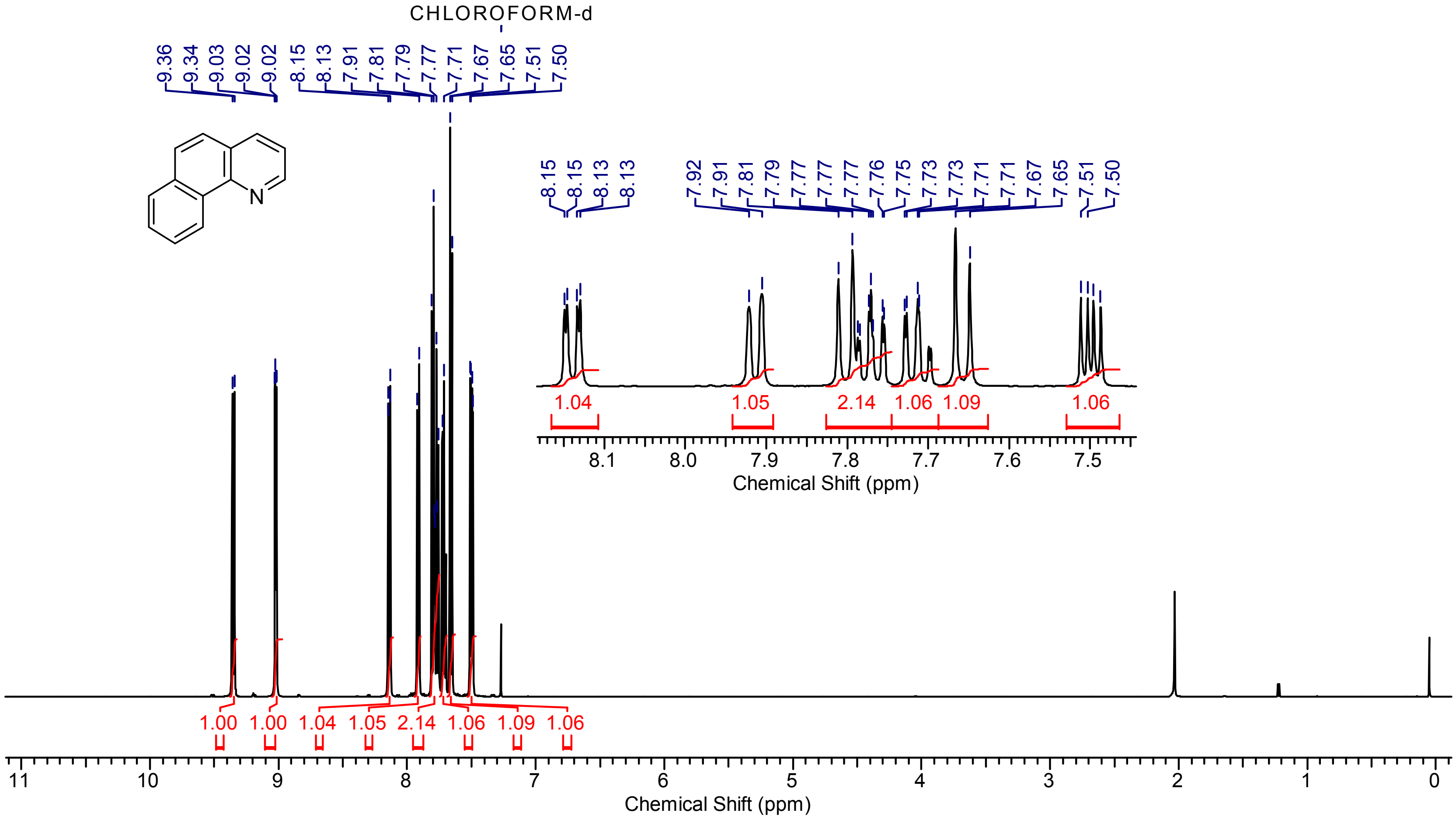

Supplementary Figure 21. ^{1}**H NMR of 2l**

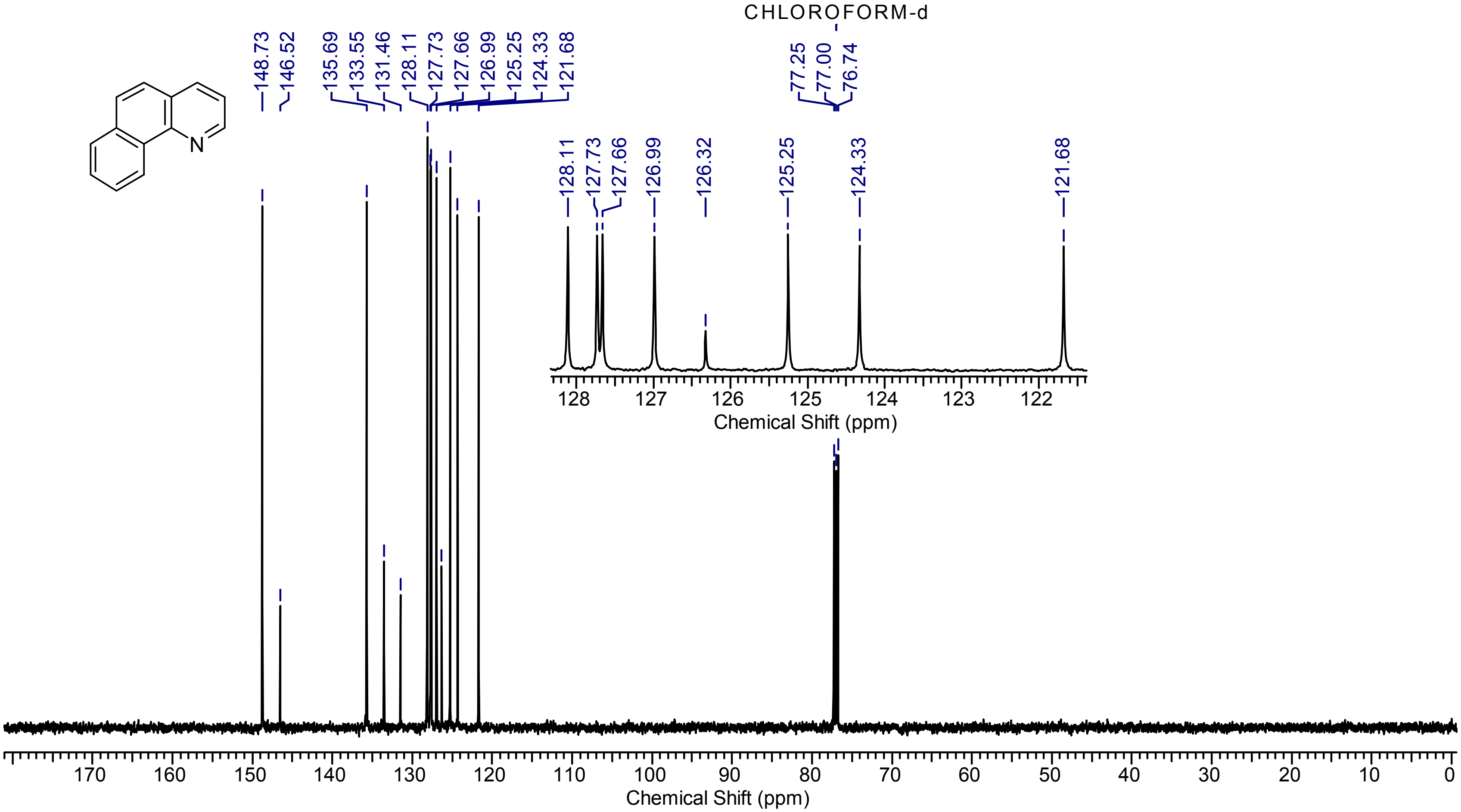

Supplementary Figure 22. ^{13}C NMR of 2l

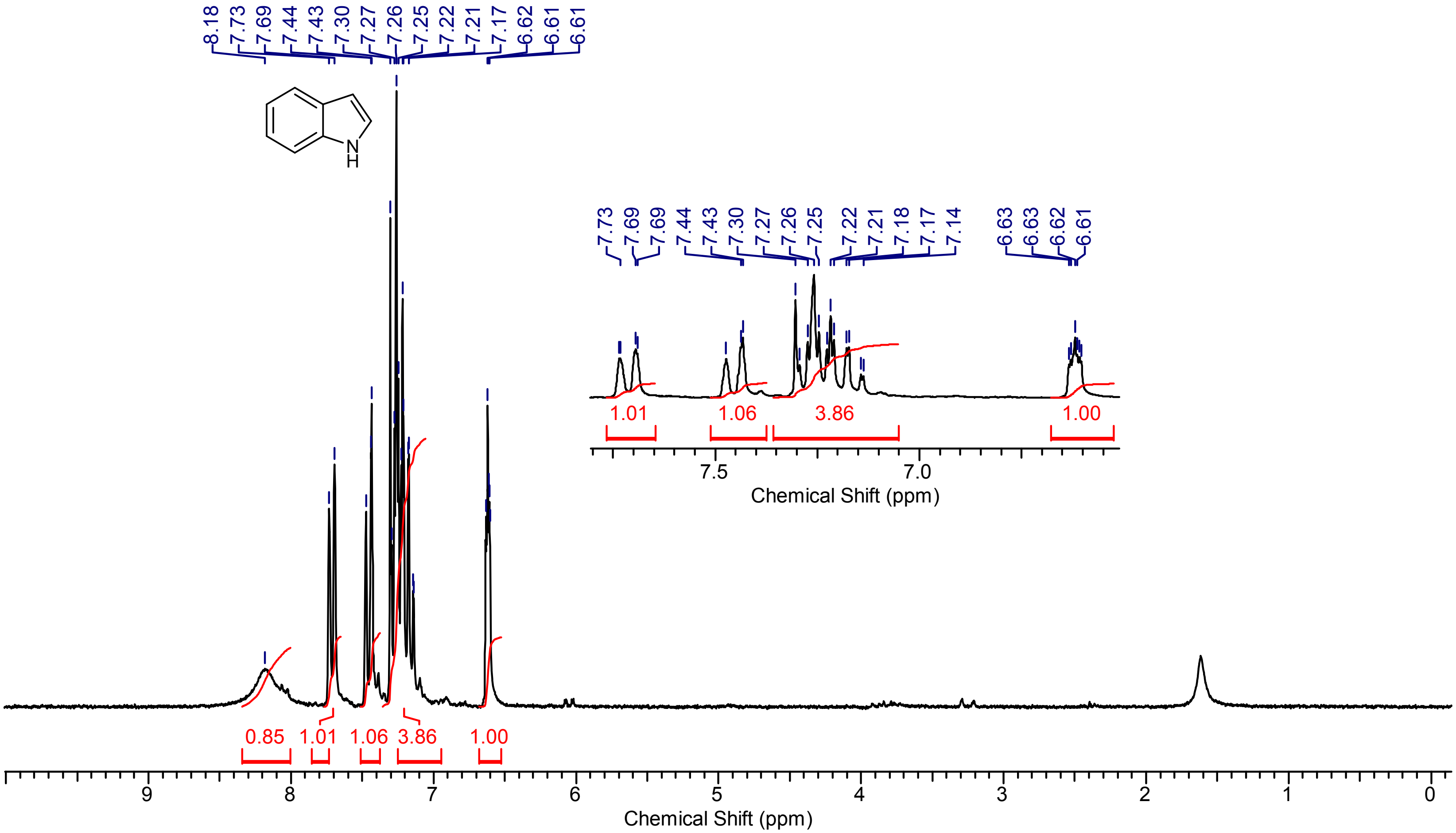

Supplementary Figure 23. ^{1}H NMR of 2m

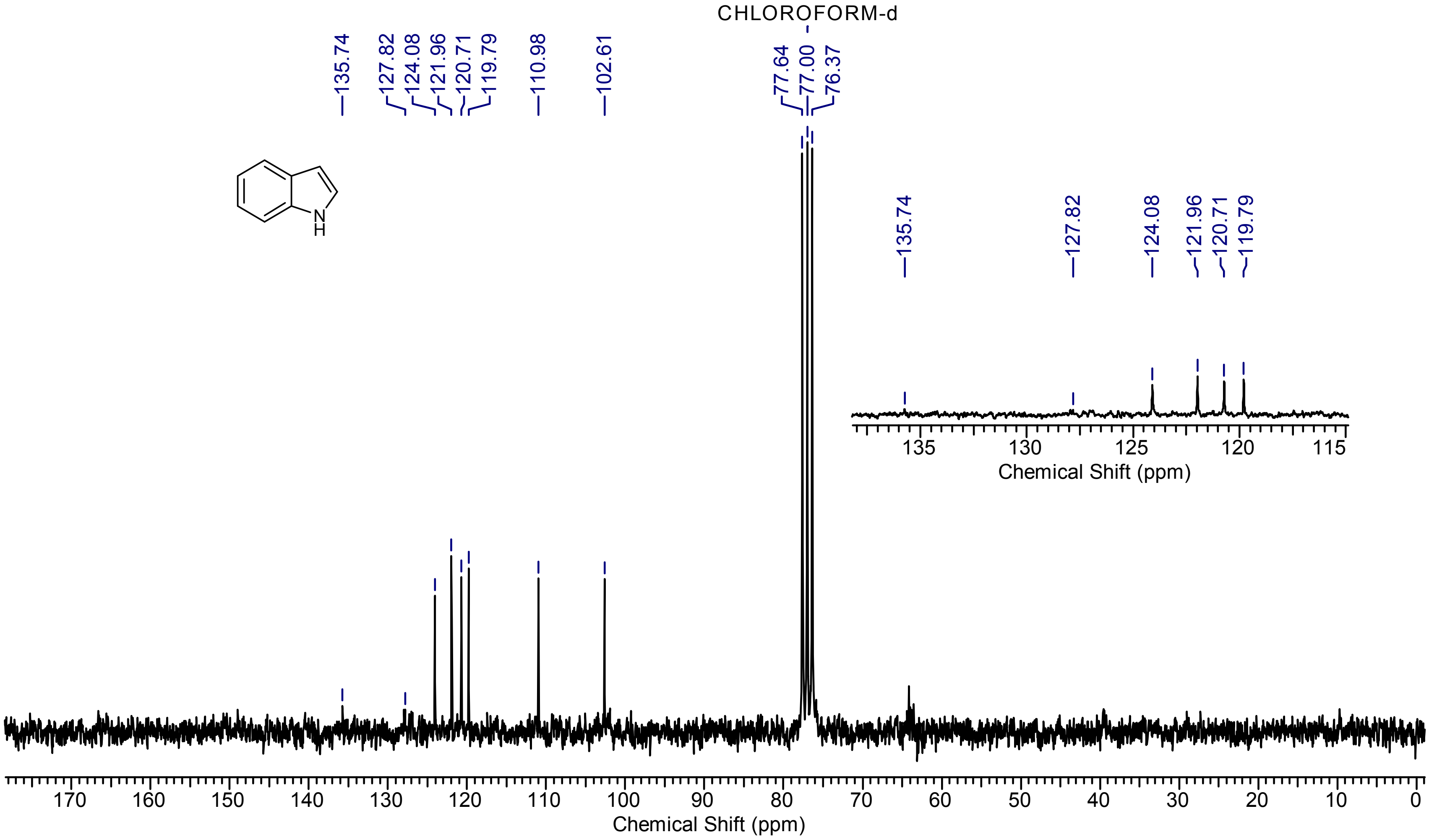

Supplementary Figure 24. ^{13}C NMR of 2m

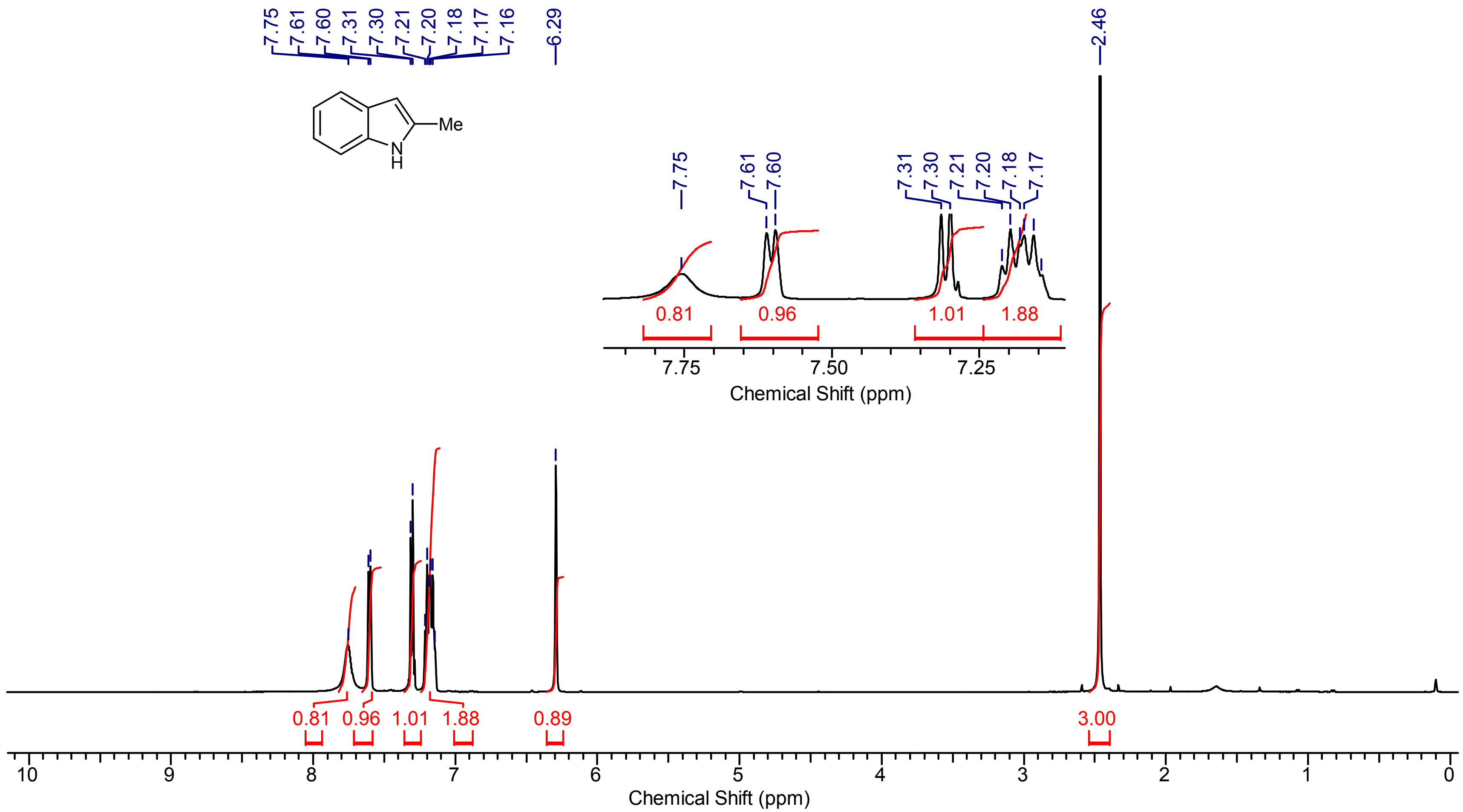

Supplementary Figure 25. ^{1}H NMR of 2n

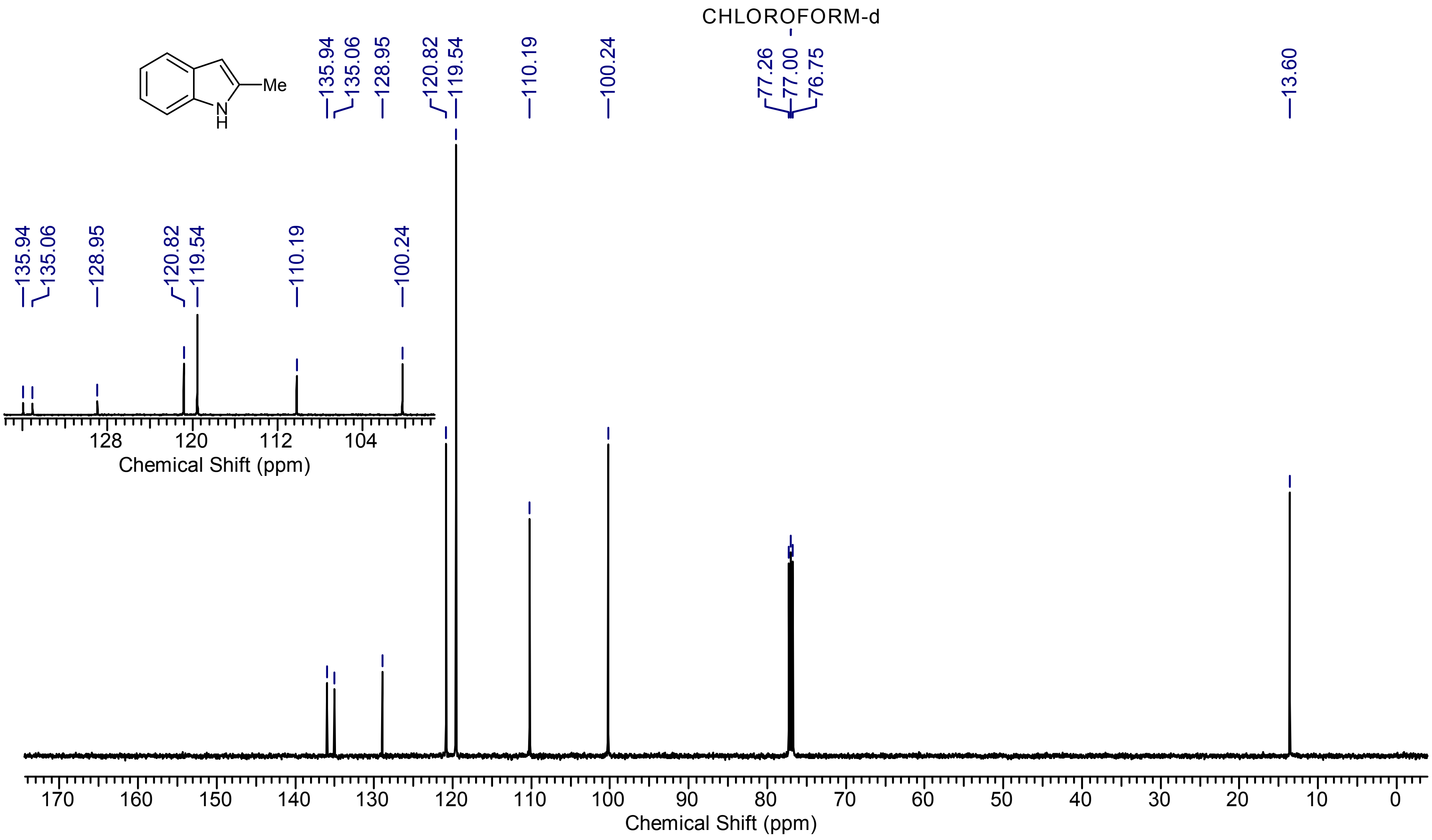

Supplementary Figure 26. ^{13}C NMR of 2n

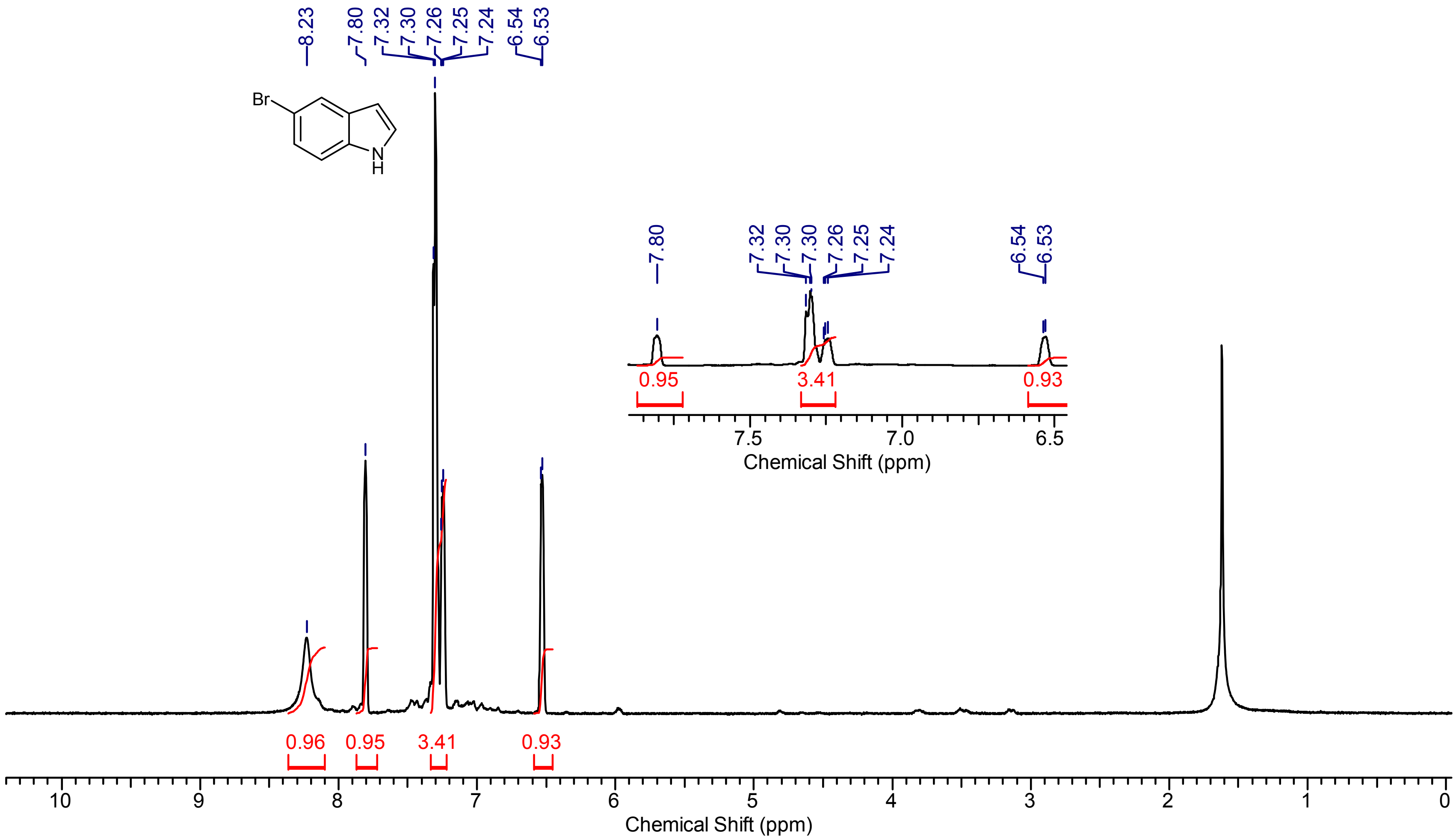

Supplementary Figure 27. ^{1}H NMR of 2o

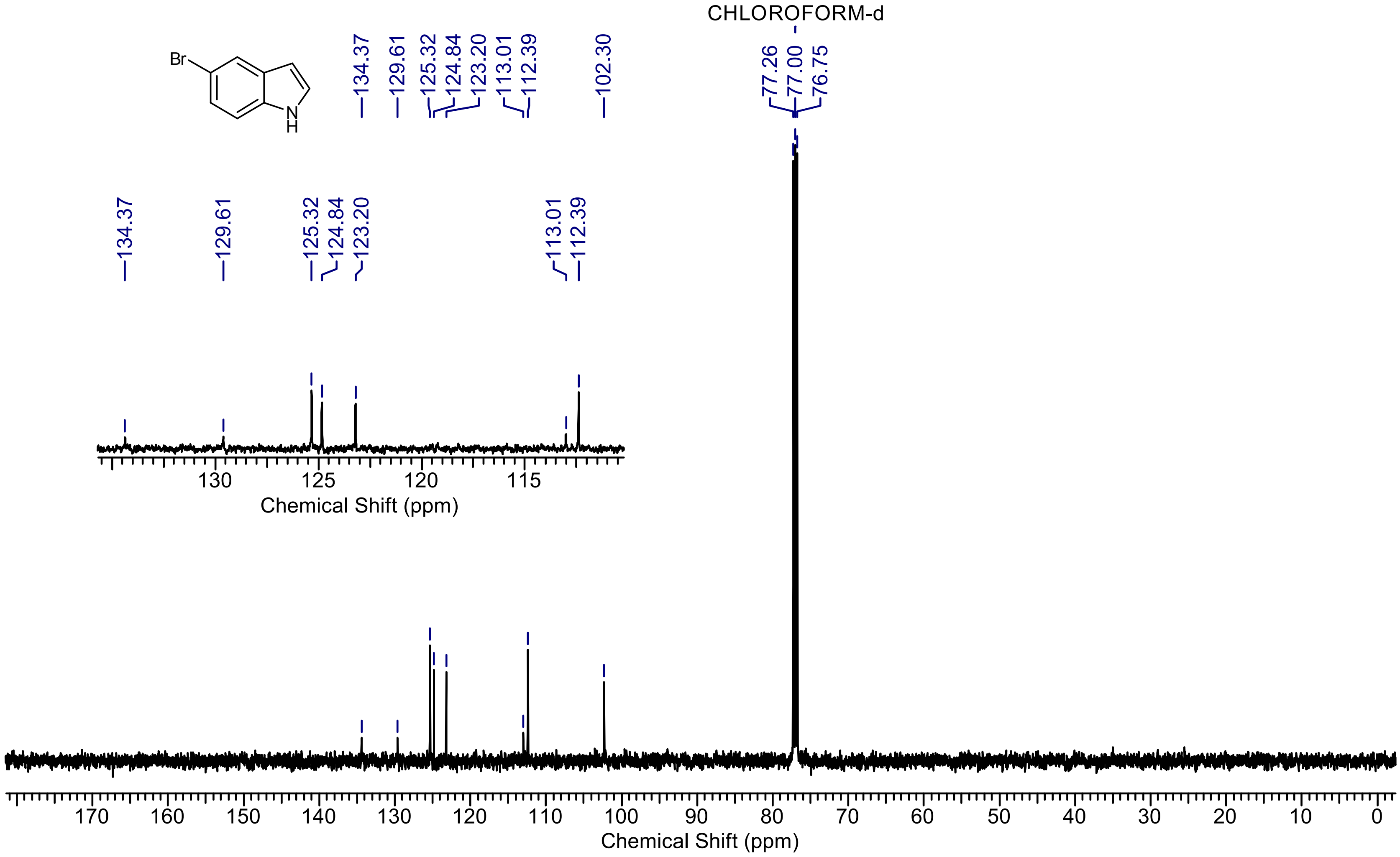

Supplementary Figure 28. ^{13}C NMR of 2o

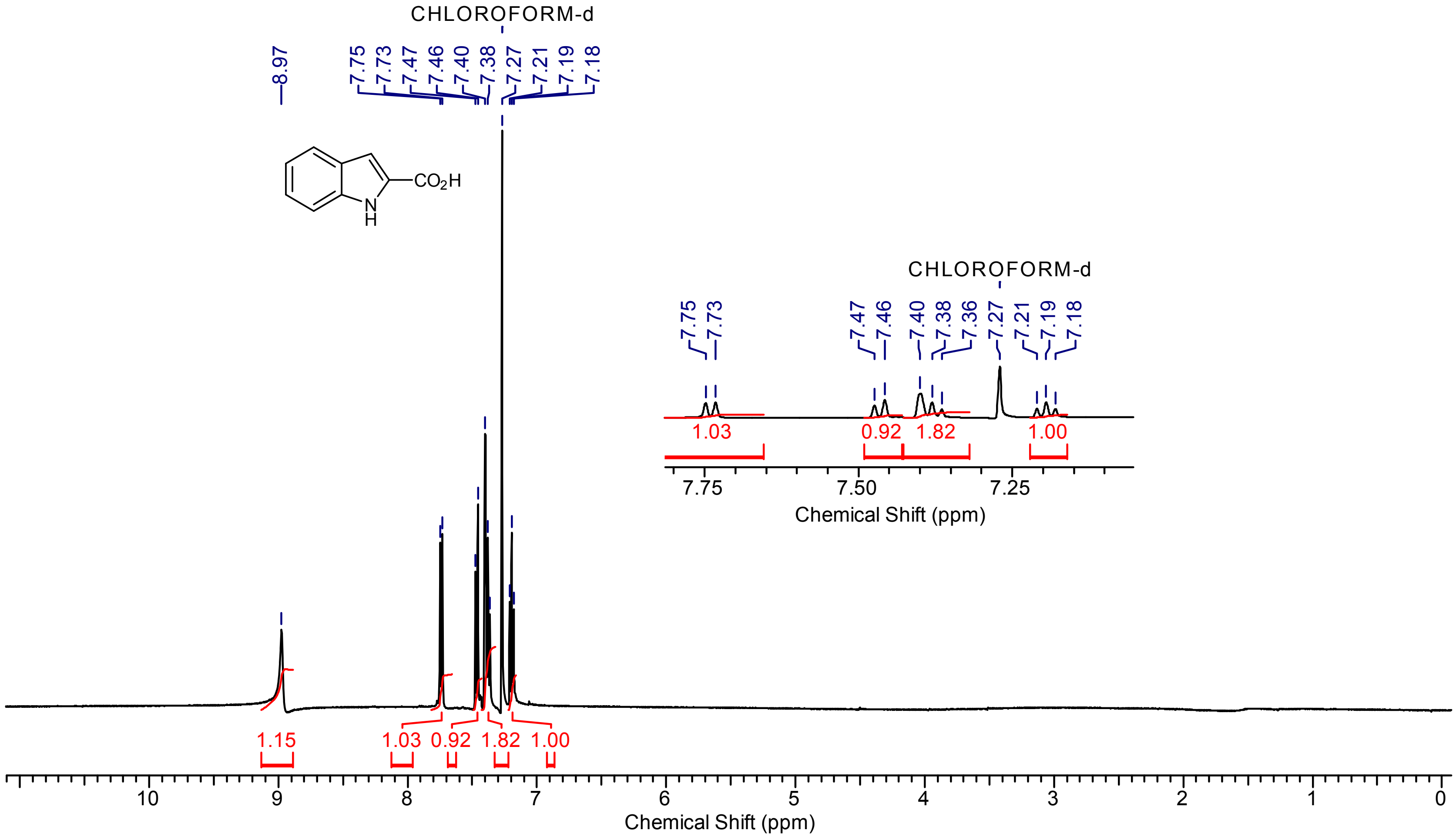

Supplementary Figure 29. ^{1}H NMR of 2p

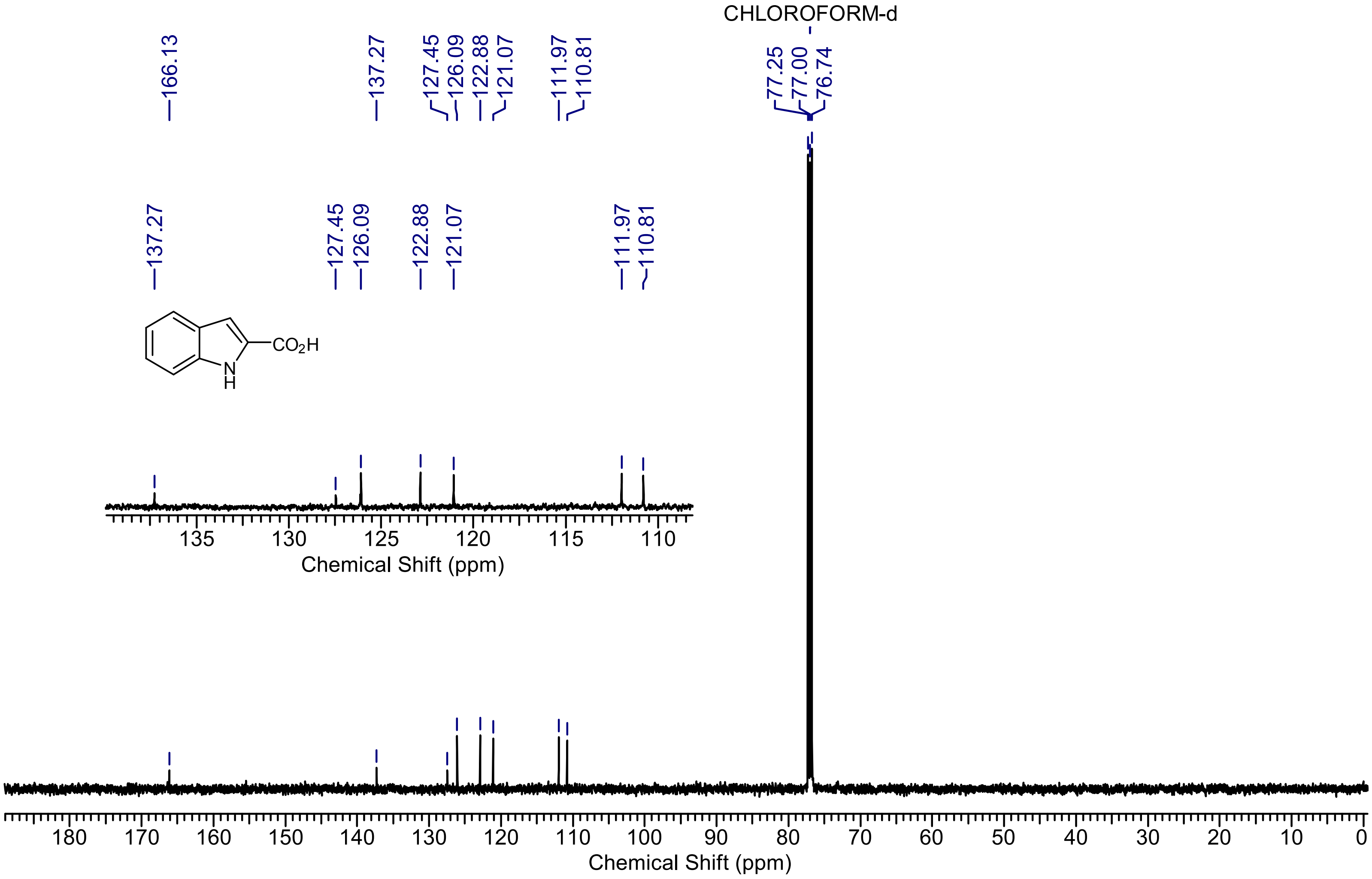

Supplementary Figure 30. ^{13}C NMR of 2p

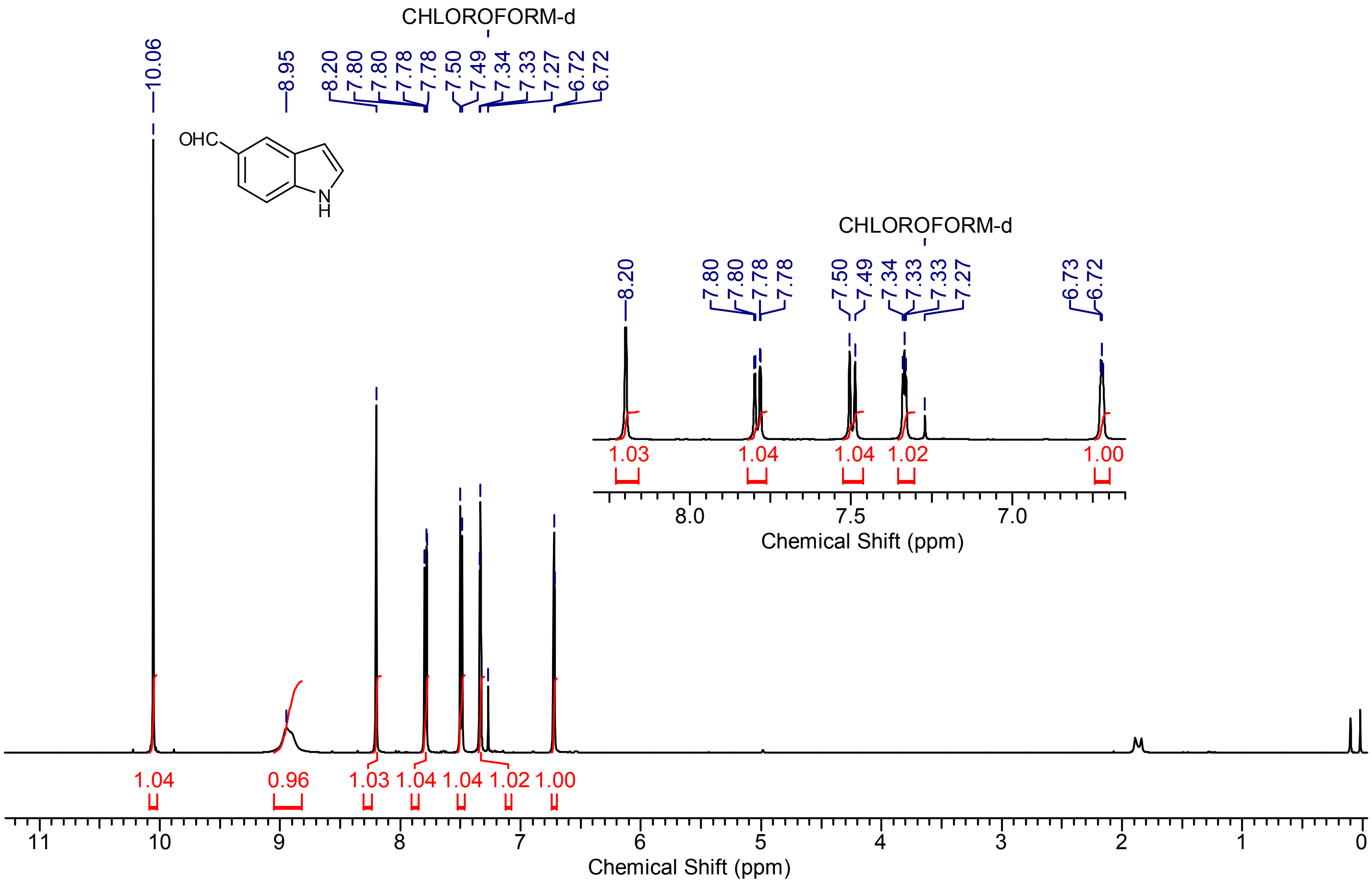

Supplementary Figure 31. ^{1}**H NMR of 2q**

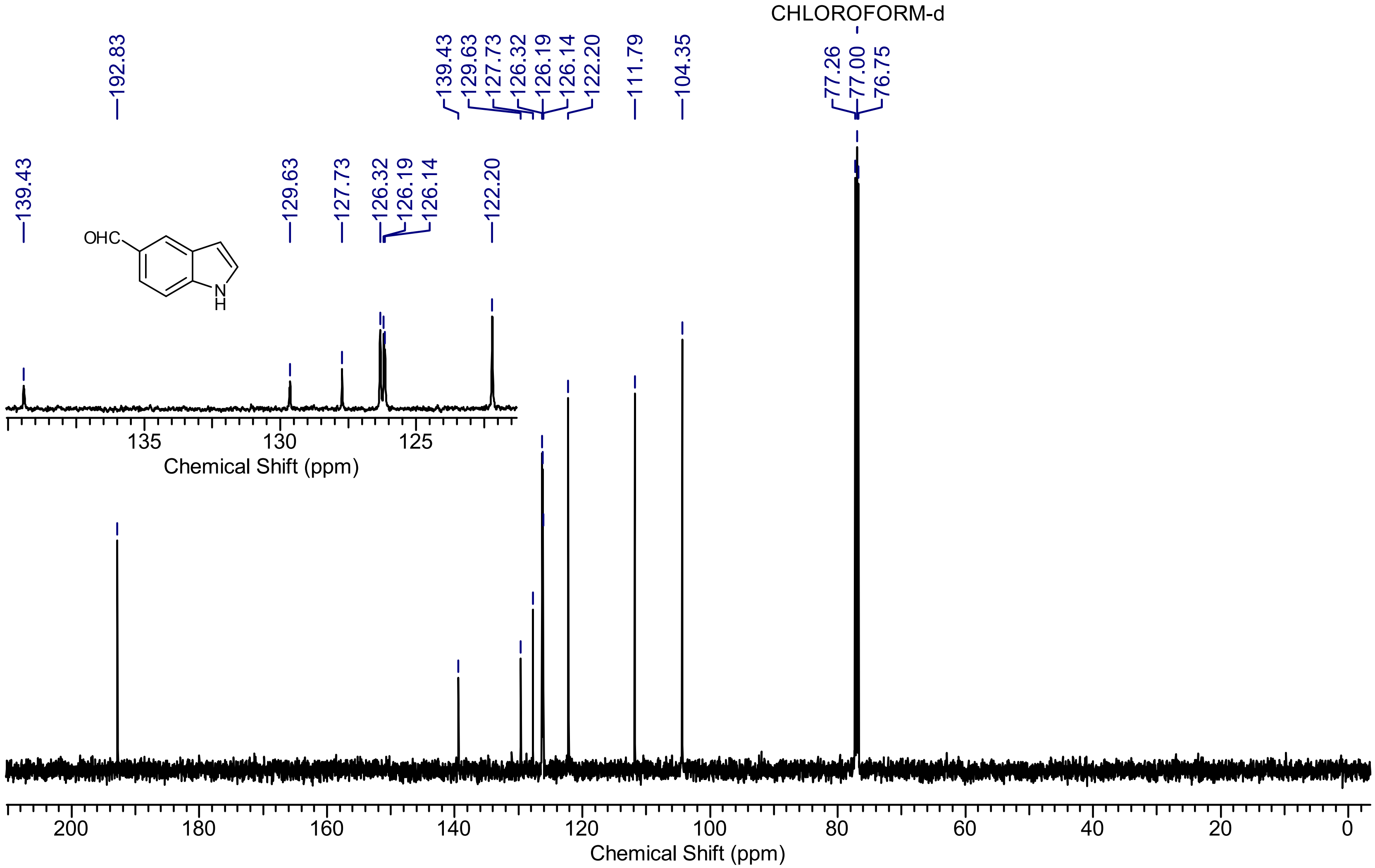

Supplementary Figure 32. ^{13}**C NMR of 2q**

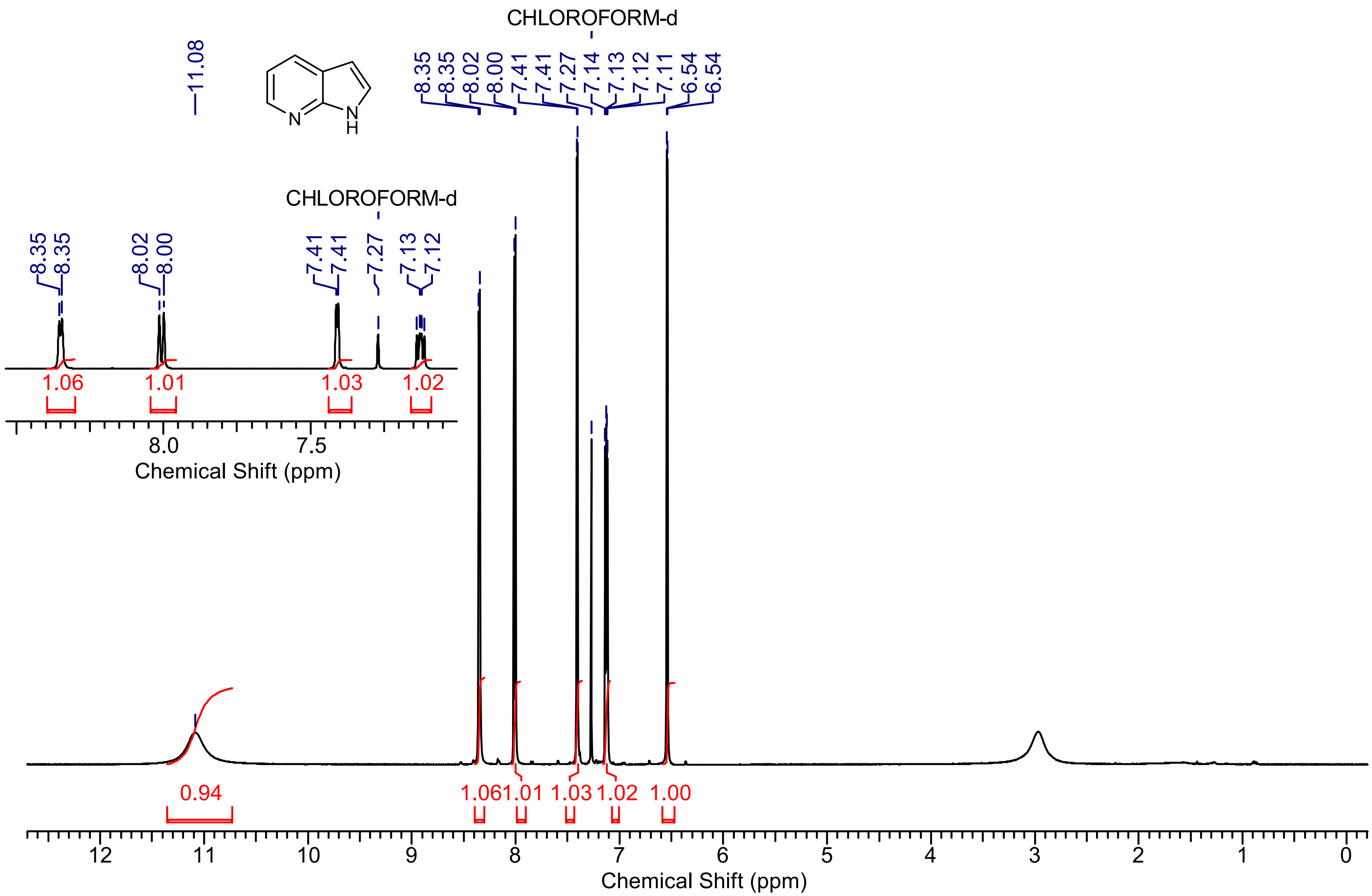

Supplementary Figure 33. ^{1}H NMR of 2r

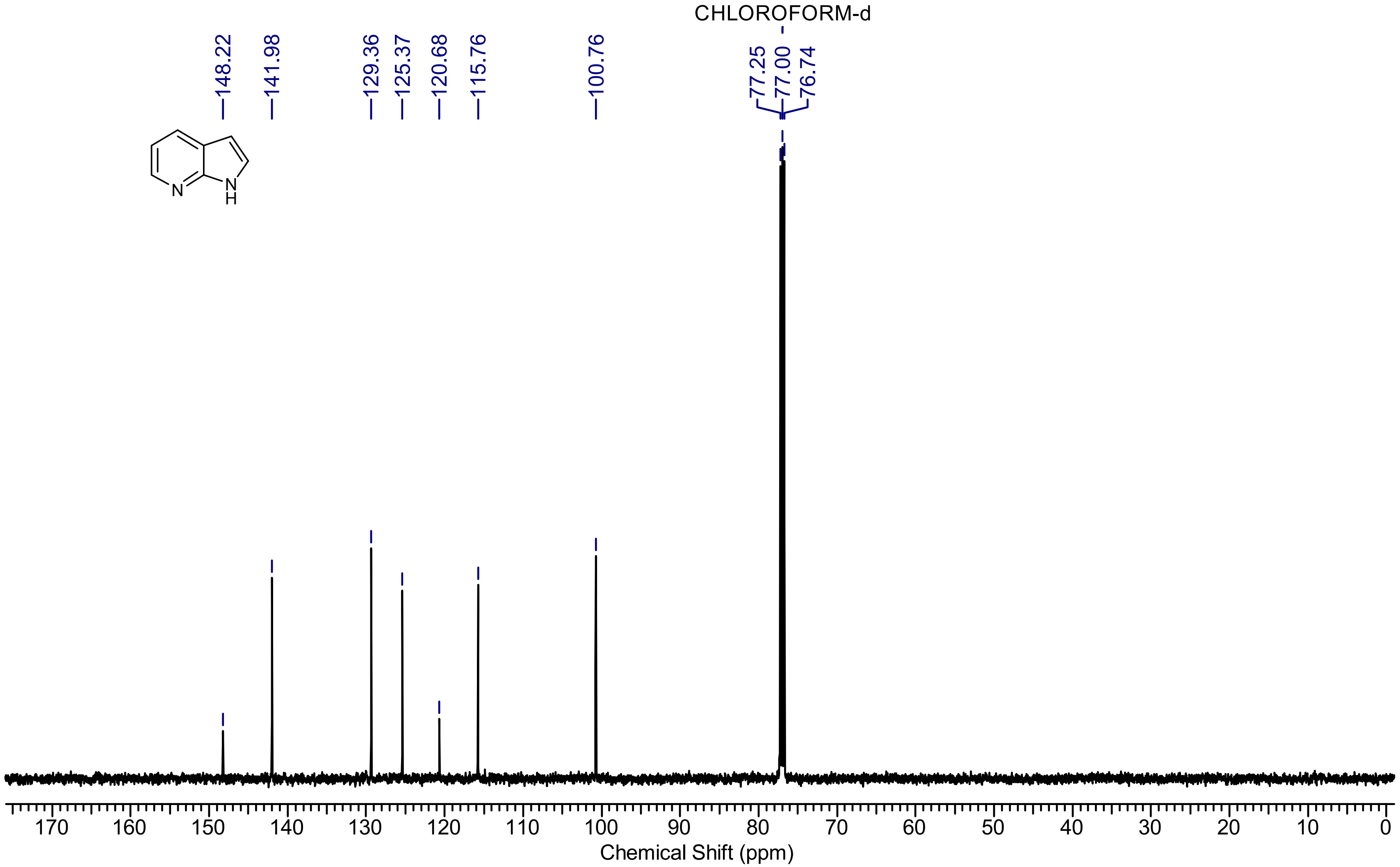

Supplementary Figure 34. ^{13}C NMR of 2r

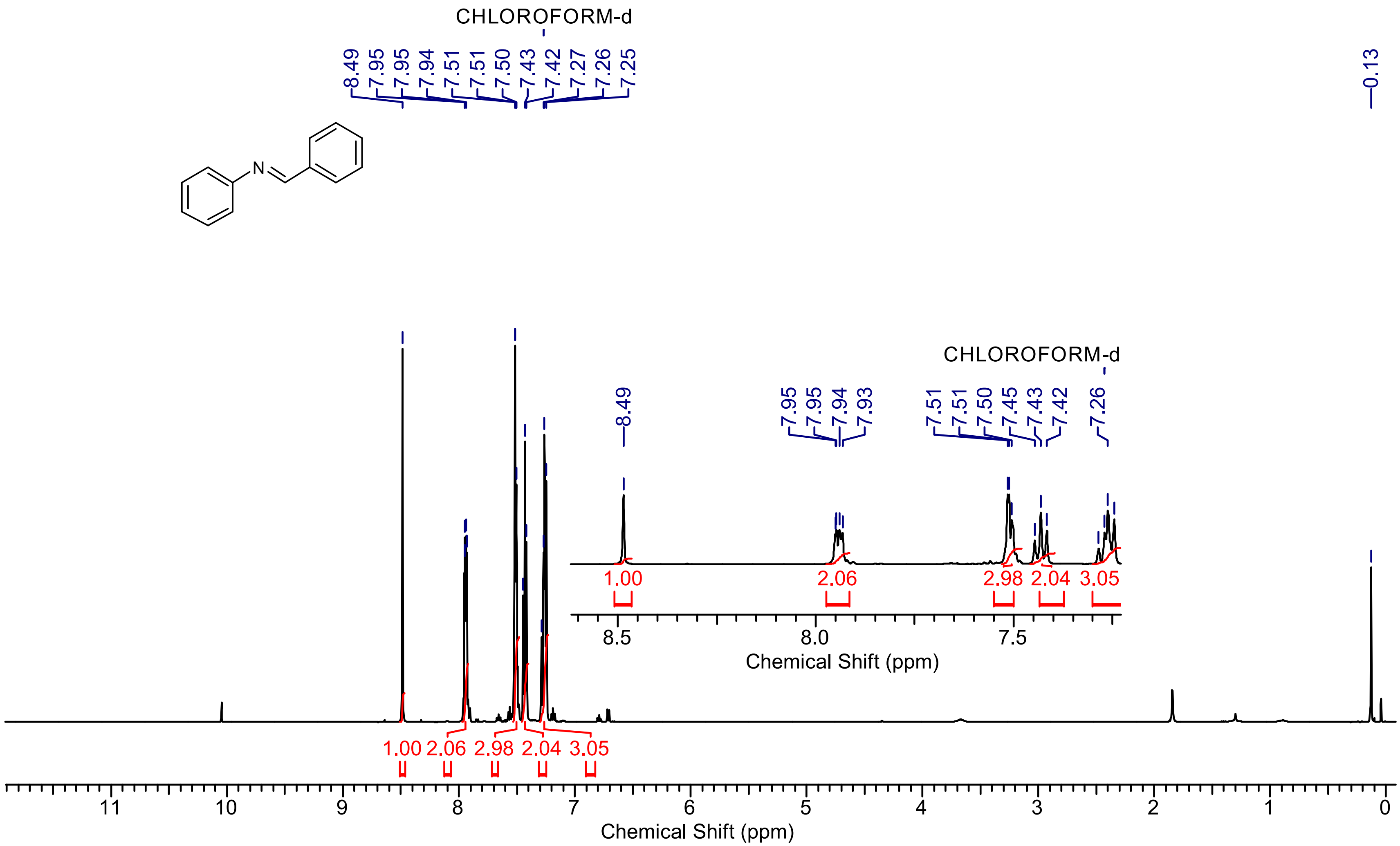

Supplementary Figure 35. ^{1}H NMR of 2s

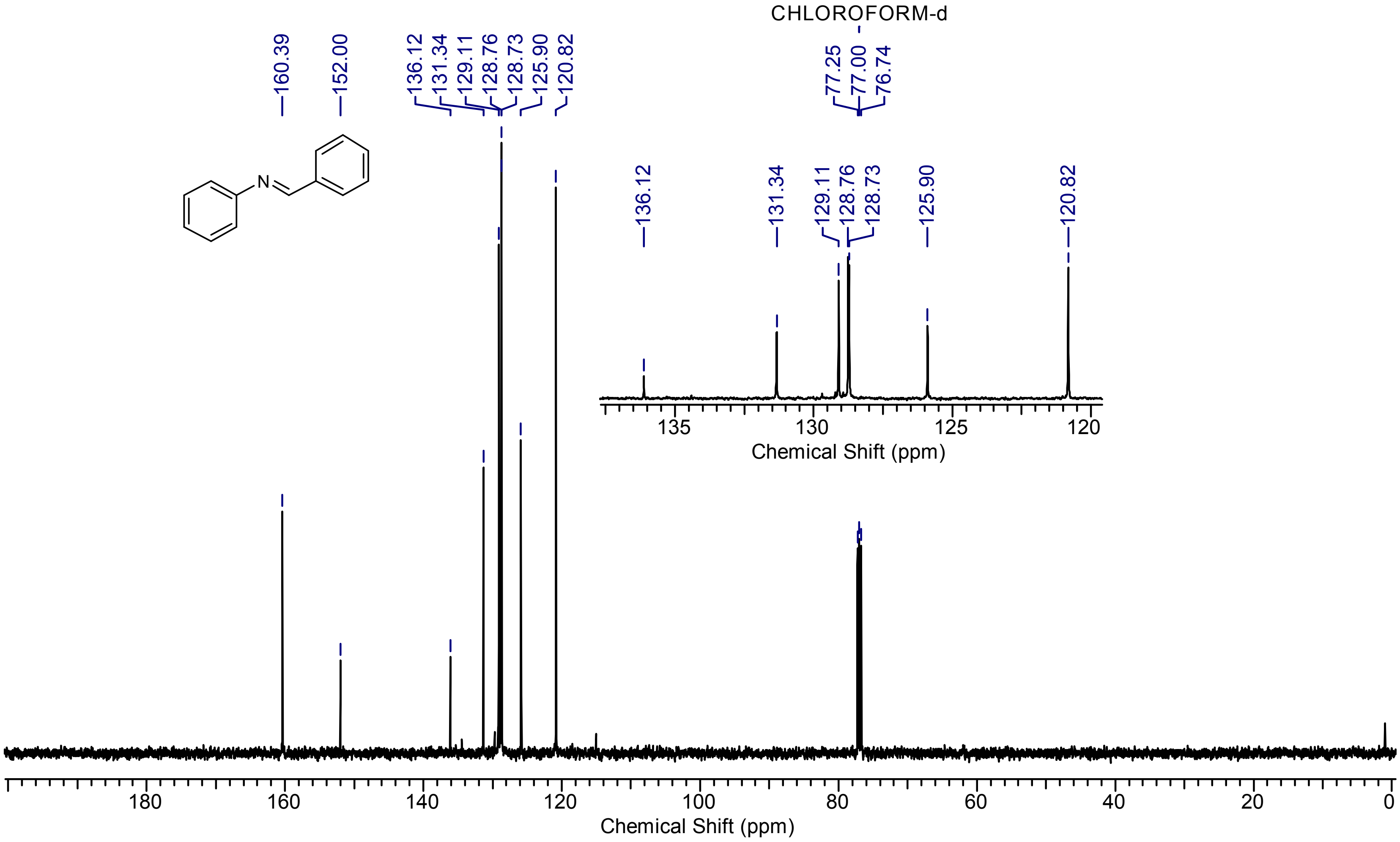

Supplementary Figure 36. ^{13}C NMR of 2s

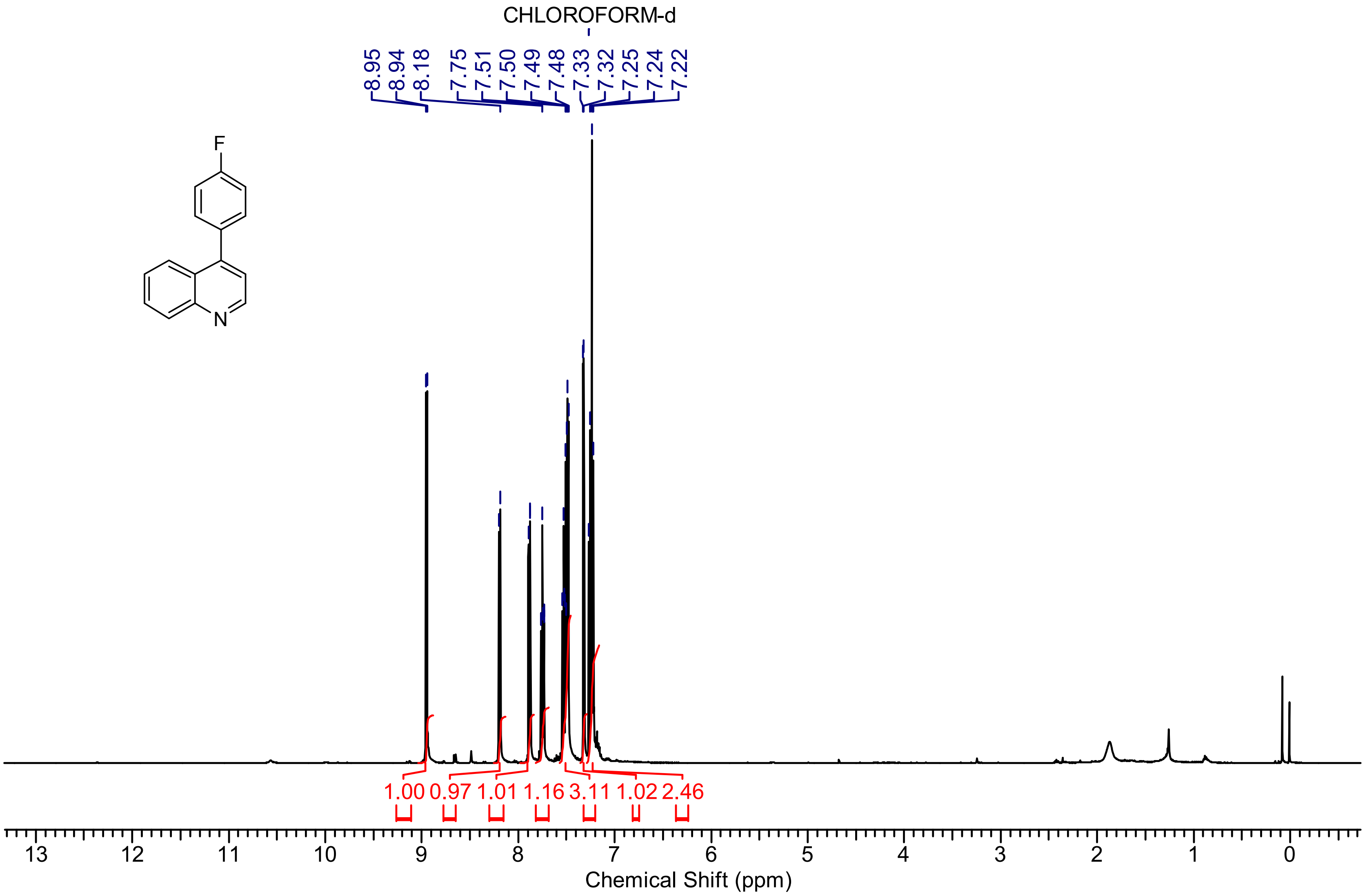

Supplementary Figure 37. ^{1}**H NMR of 4a**

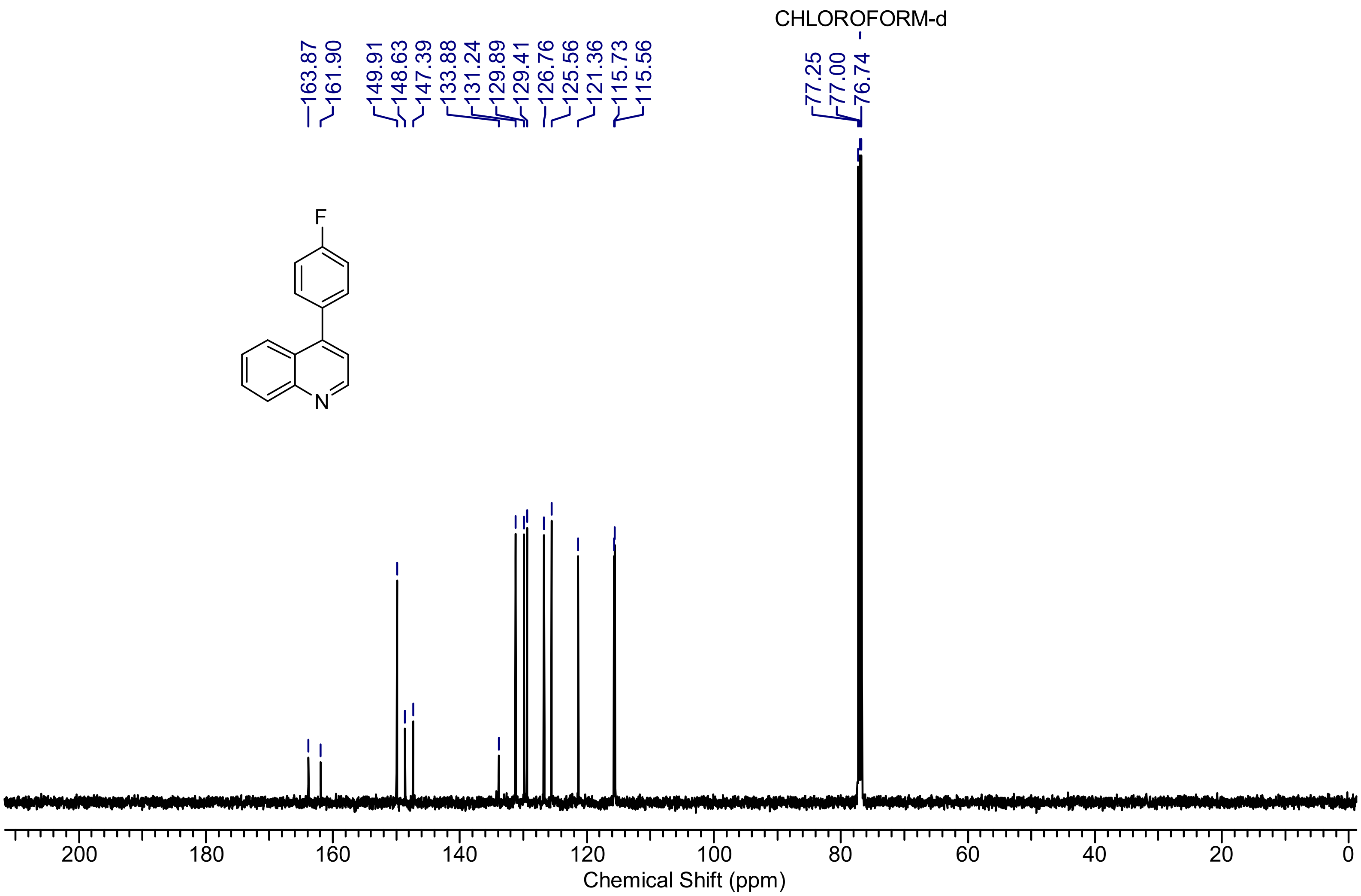

Supplementary Figure 38. ^{13}**C NMR of 4a**

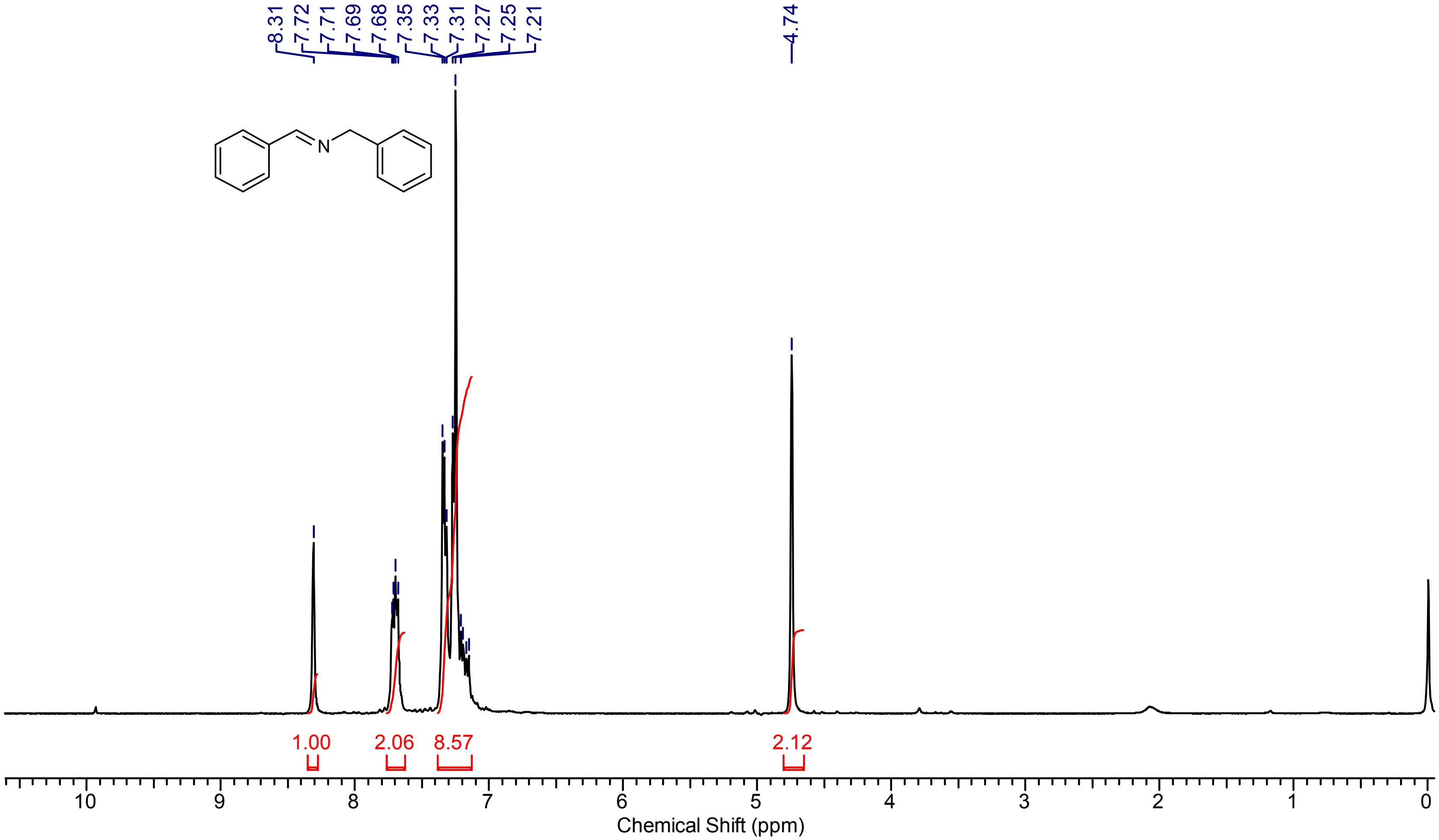

Supplementary Figure 39. ^{1}H NMR of 6a

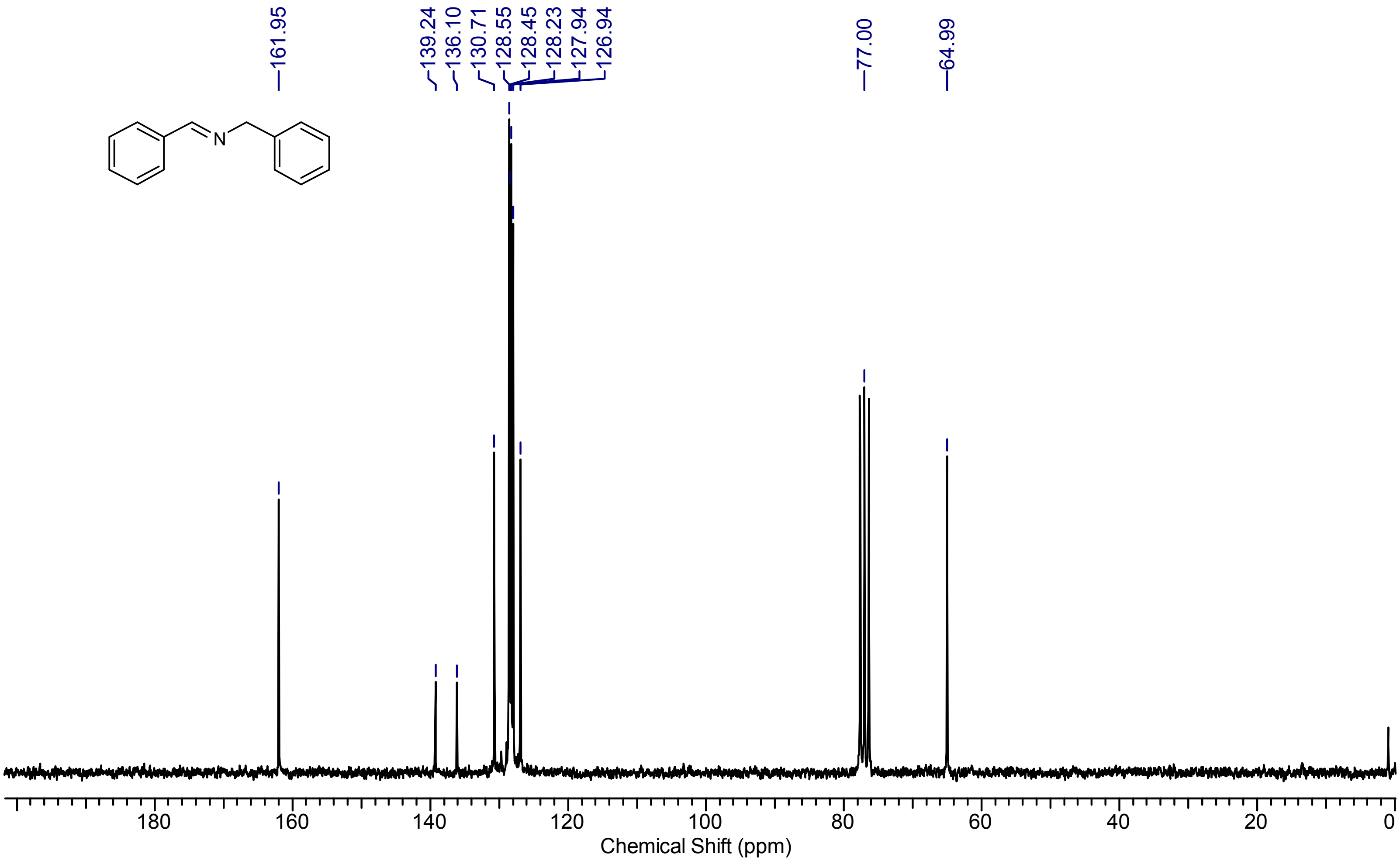

Supplementary Figure 40. ^{13}C NMR of 6a

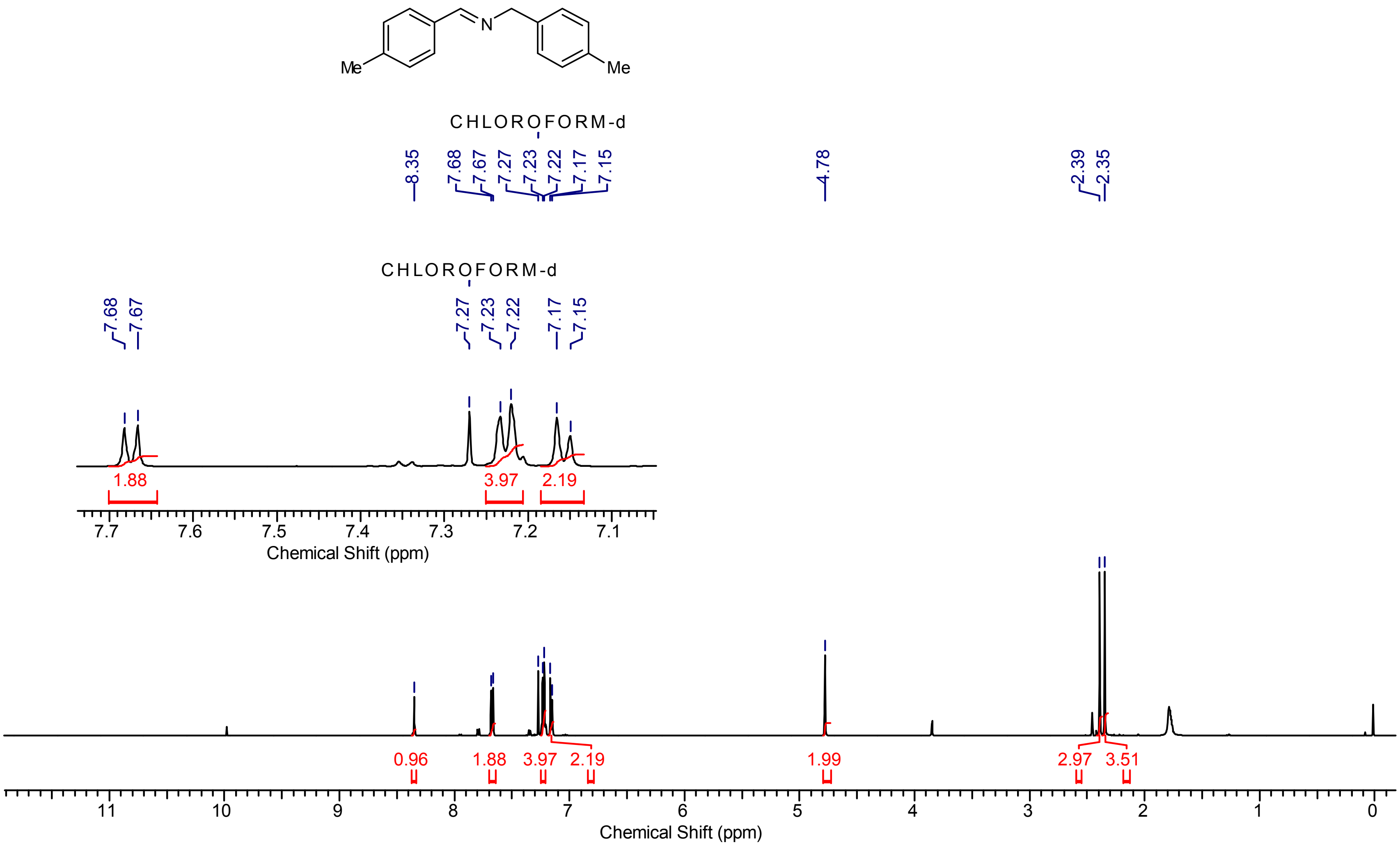

Supplementary Figure 41. ^{1}H NMR of 6b

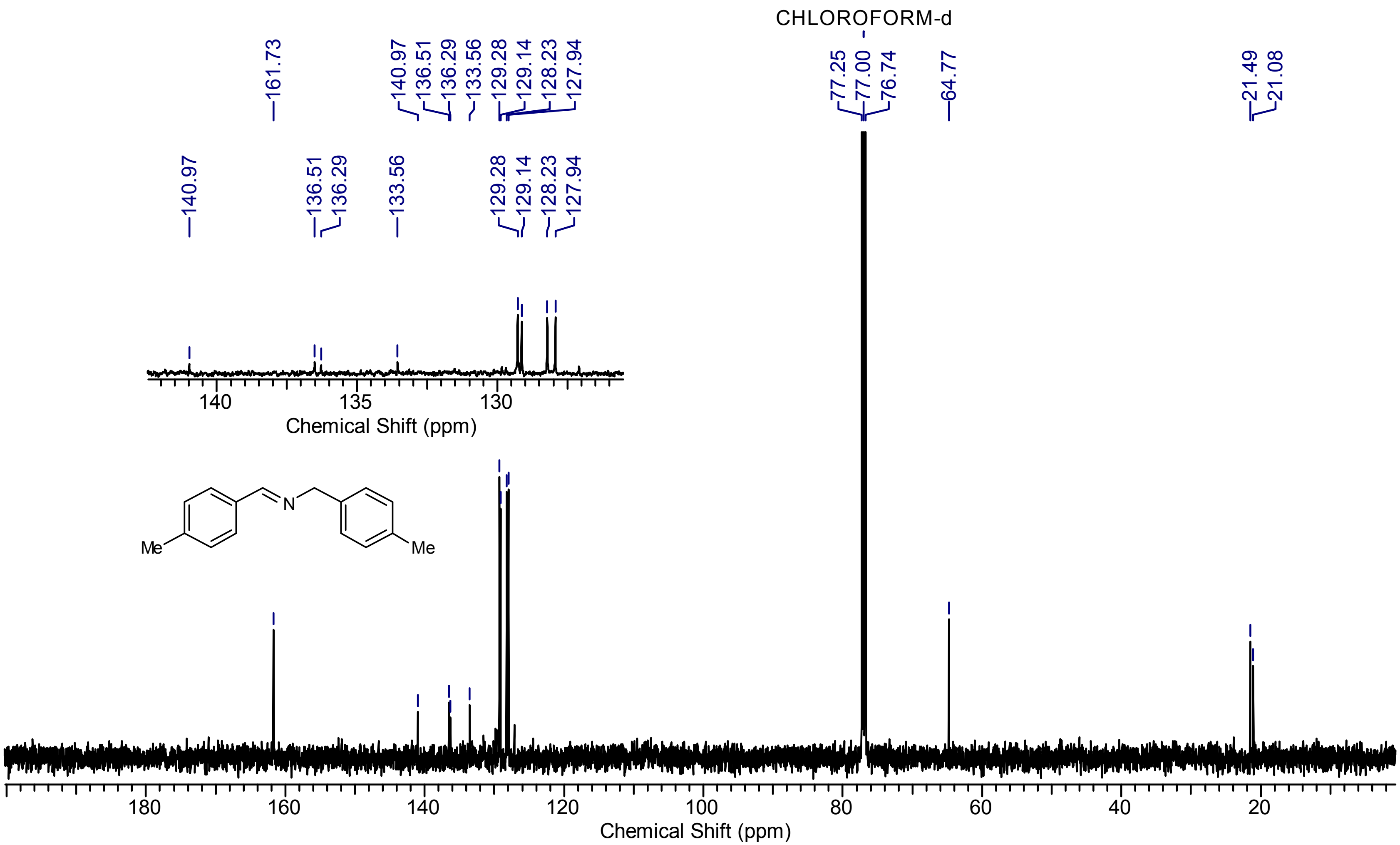

Supplementary Figure 42. ^{13}**C NMR of 6b**

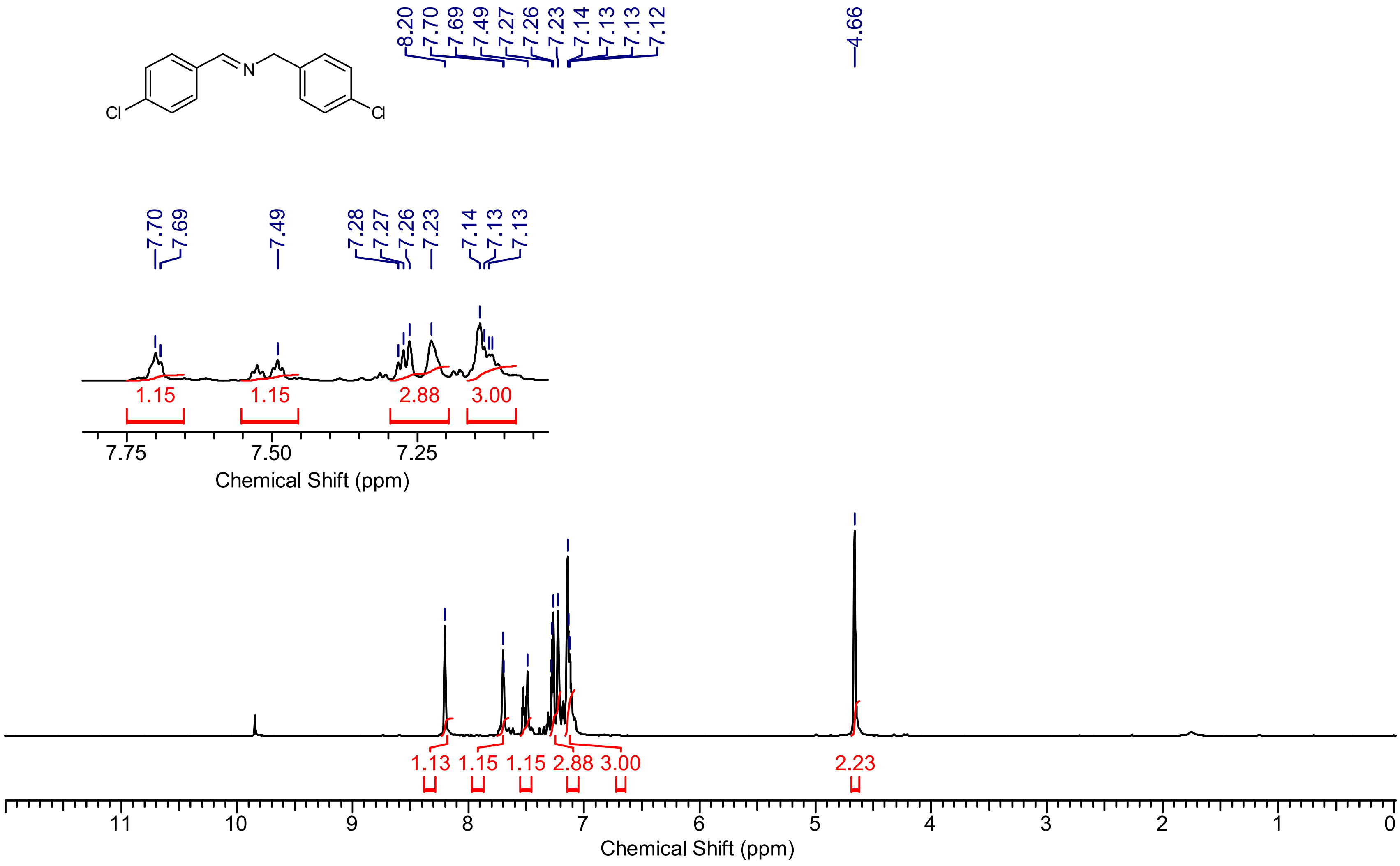

Supplementary Figure 43. ^{1}H NMR of 6c

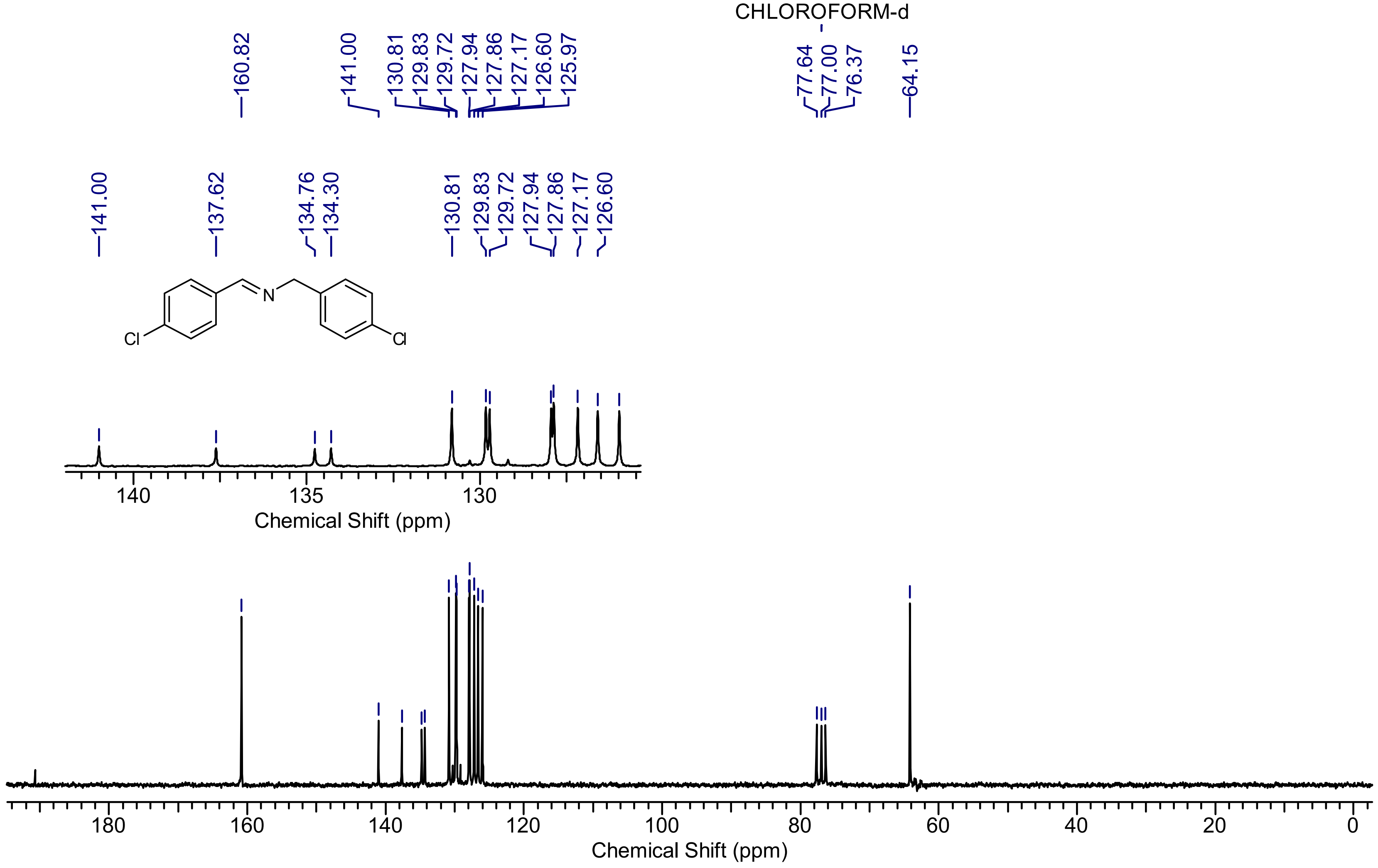

Supplementary Figure 44. ^{13}C NMR of 6c

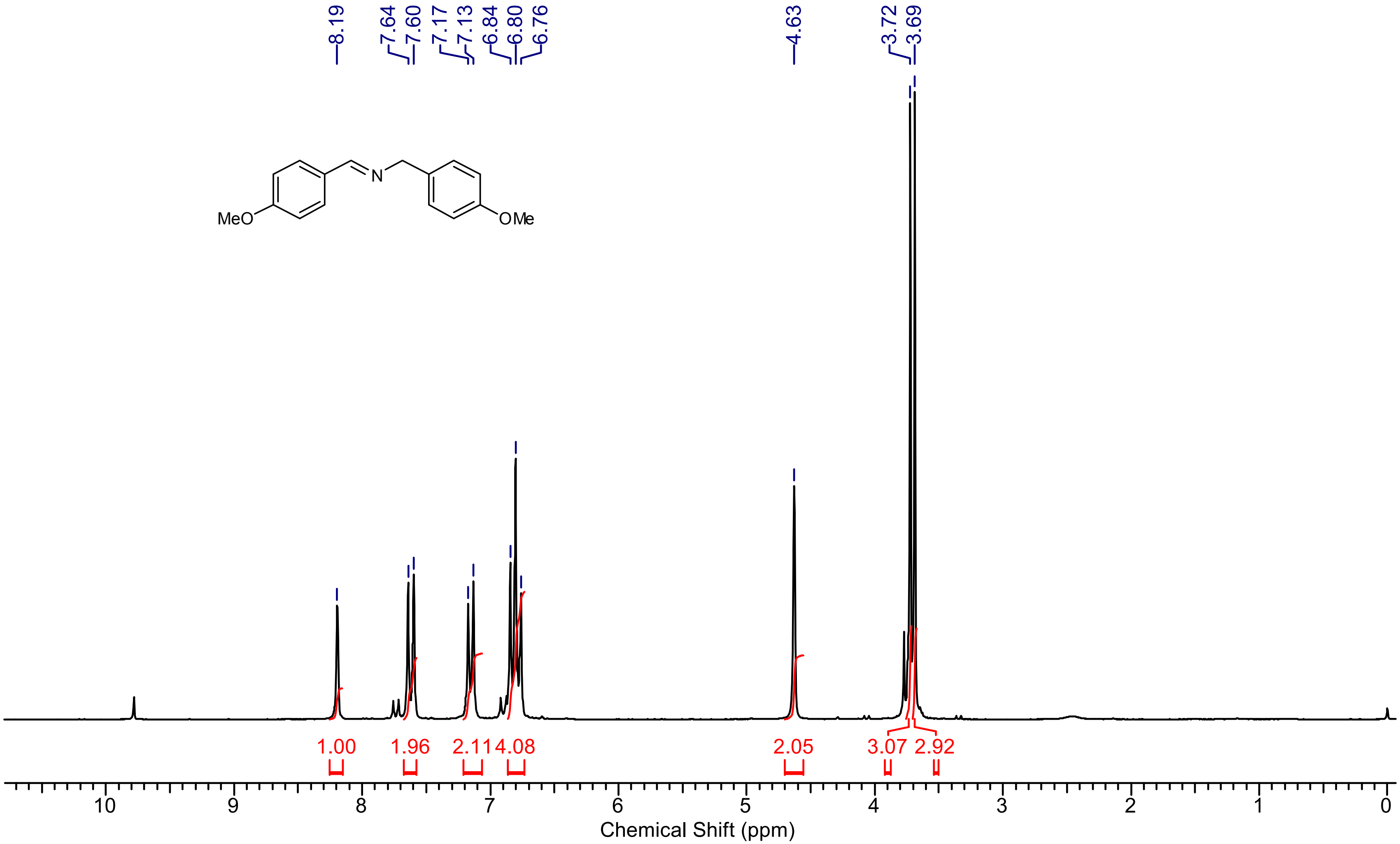

Supplementary Figure 45. ^{1}H NMR of 6d

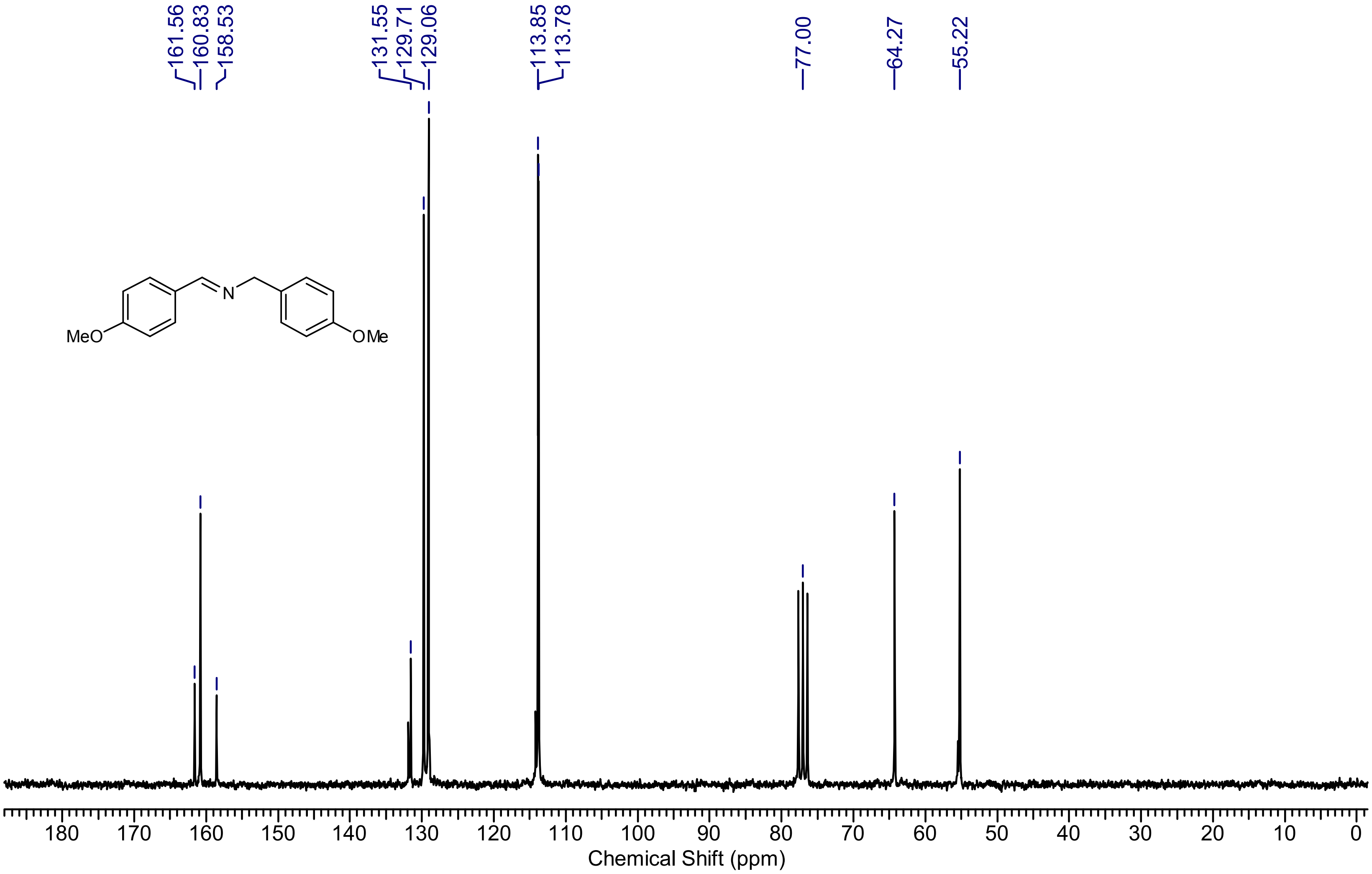

Supplementary Figure 46. ^{13}C NMR of 6d

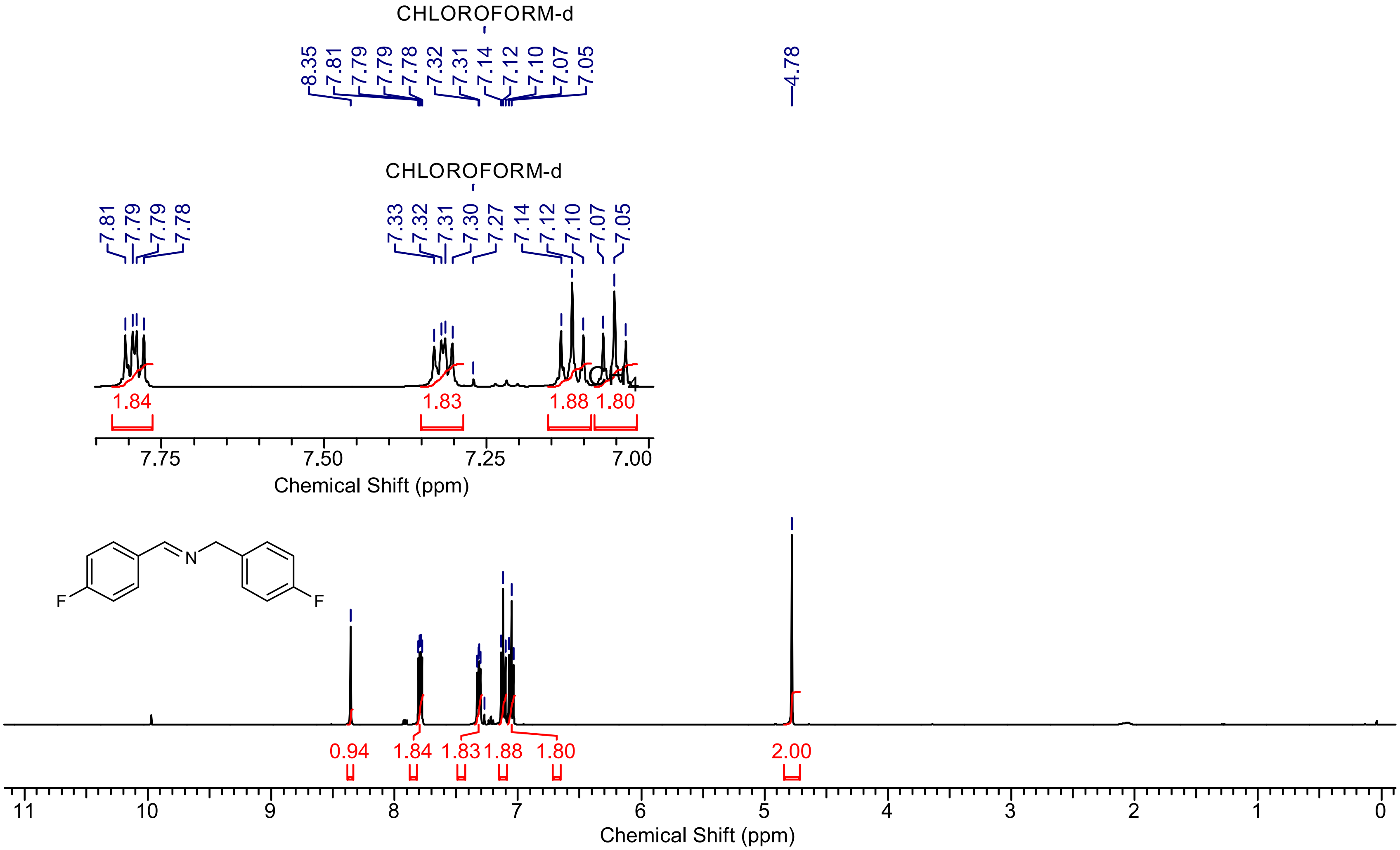

Supplementary Figure 47. ^{1}H NMR of 6e

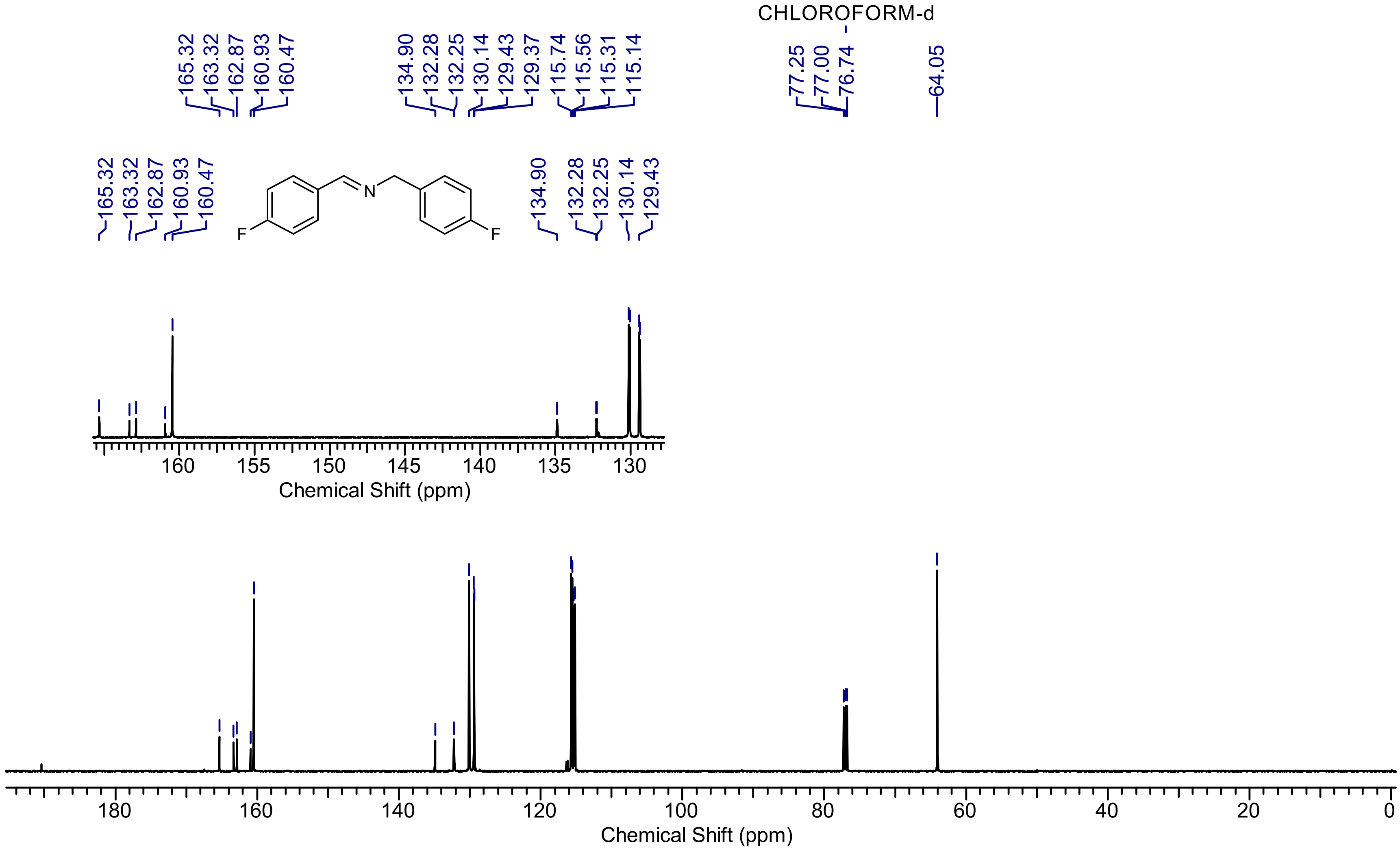

Supplementary Figure 48. ^{13}C NMR of 6e

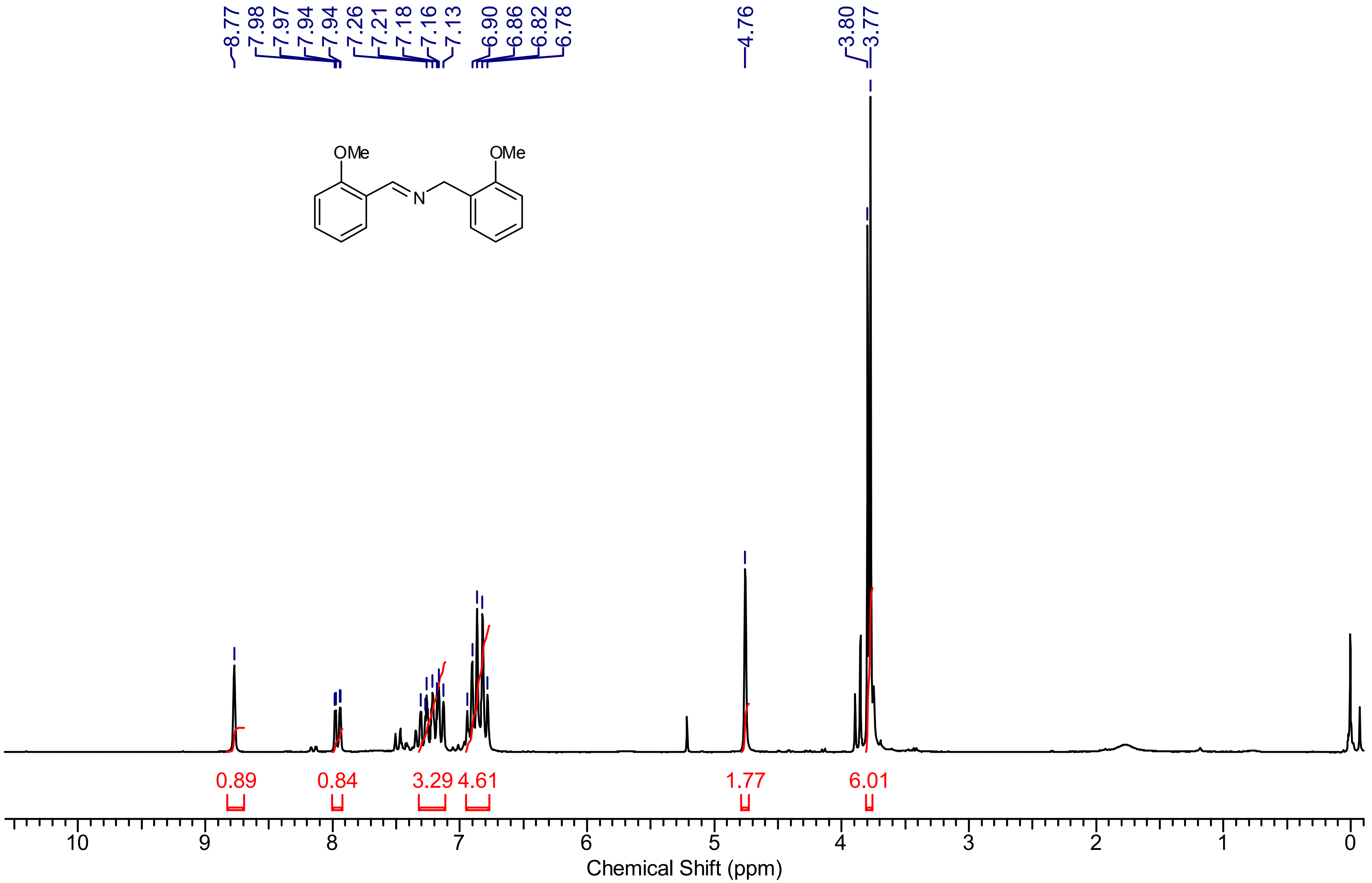

Supplementary Figure 49. ^{1}H NMR of 6f

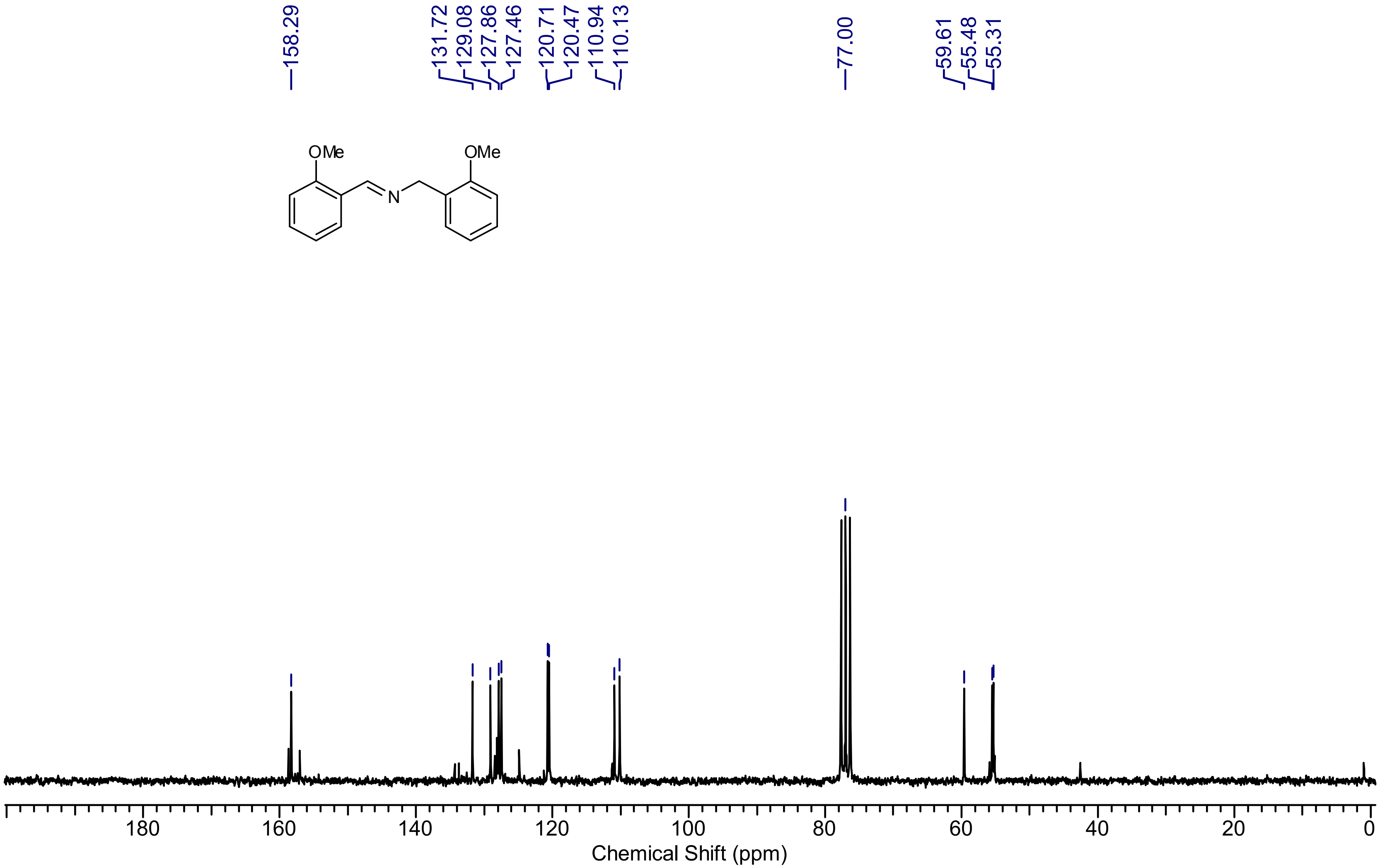

Supplementary Figure 50. ^{13}C NMR of 6f

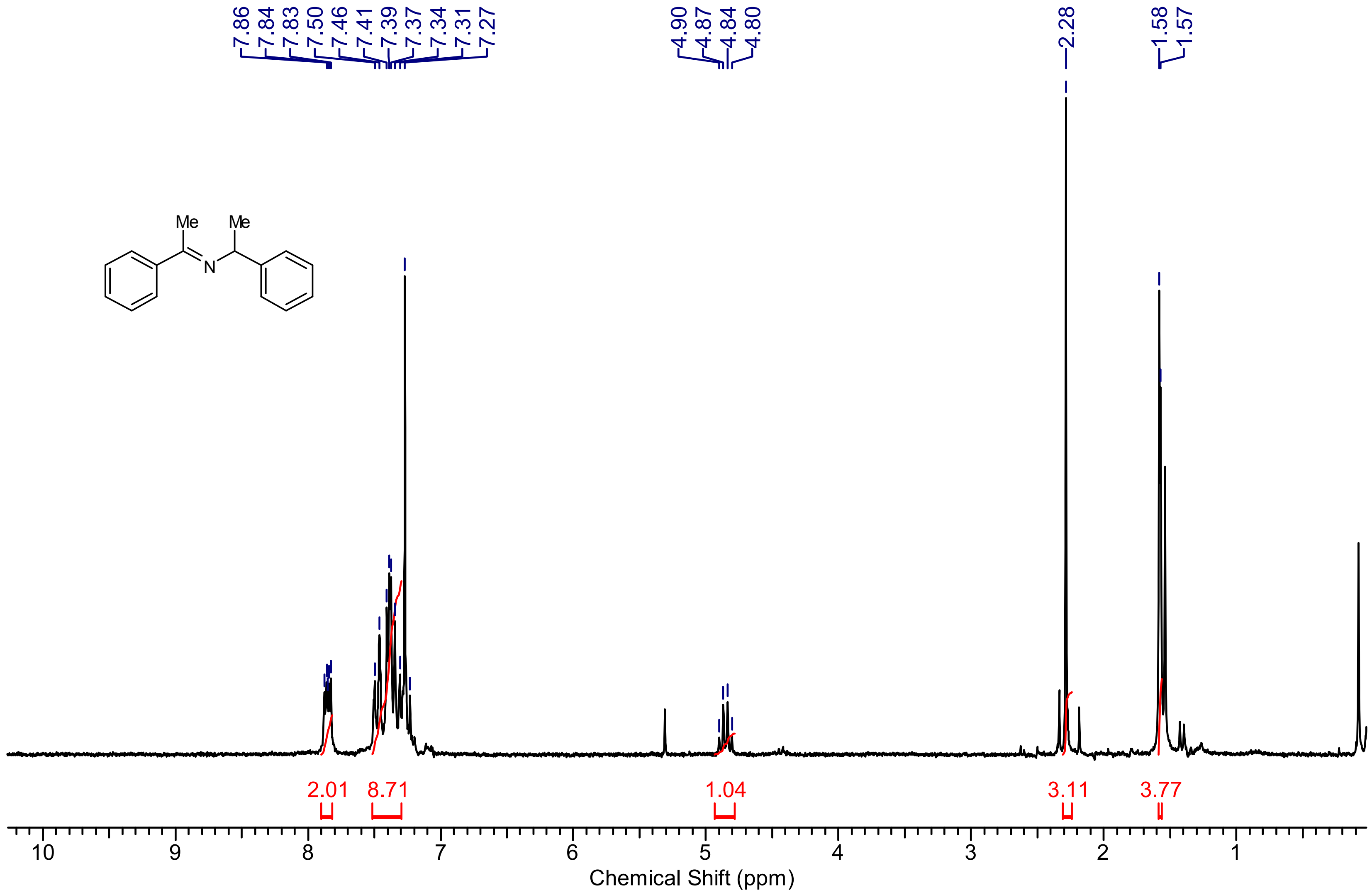

Supplementary Figure 51. ^{1}H NMR of 6h

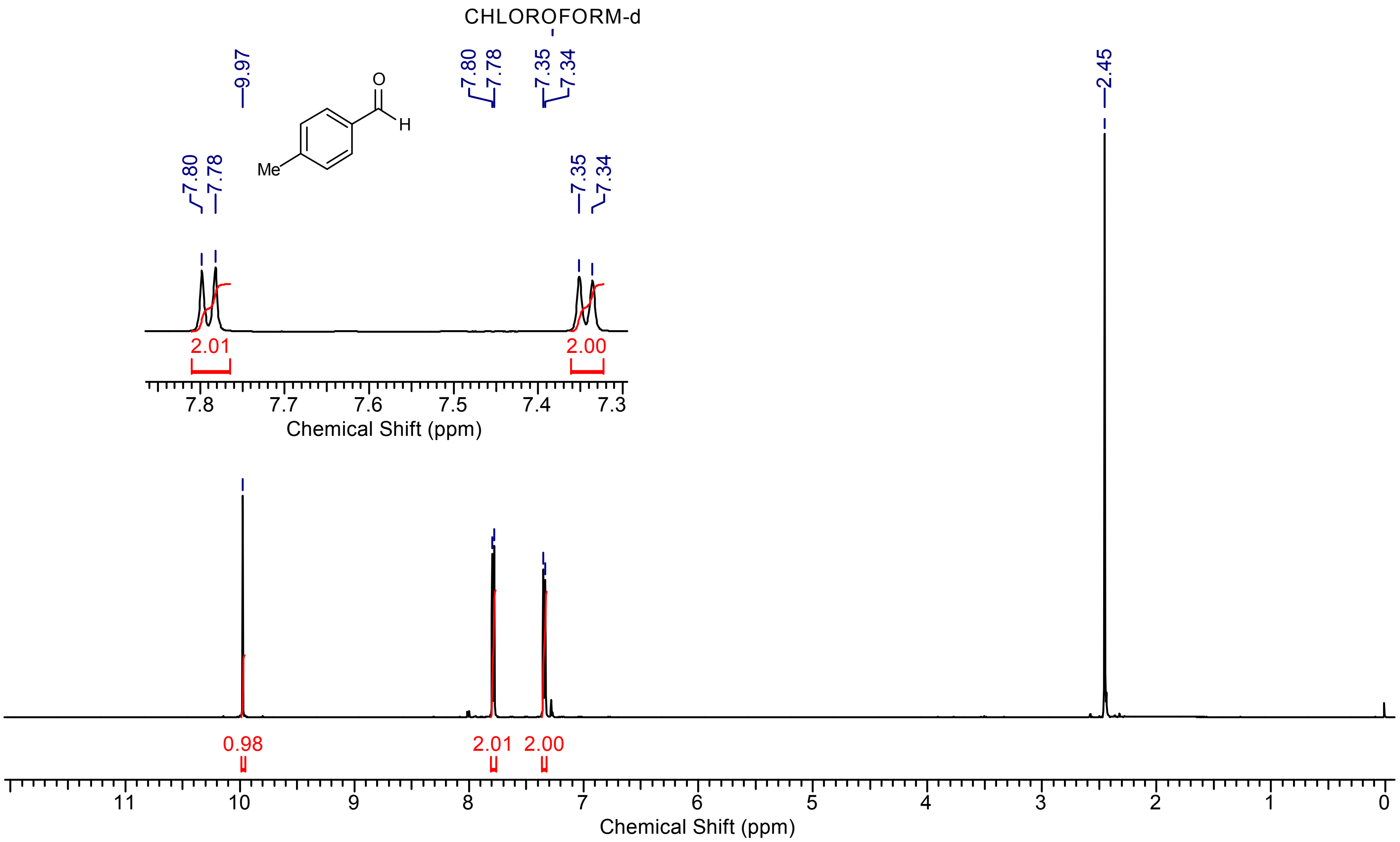

Supplementary Figure 52. ^{1}**H NMR of 9a**

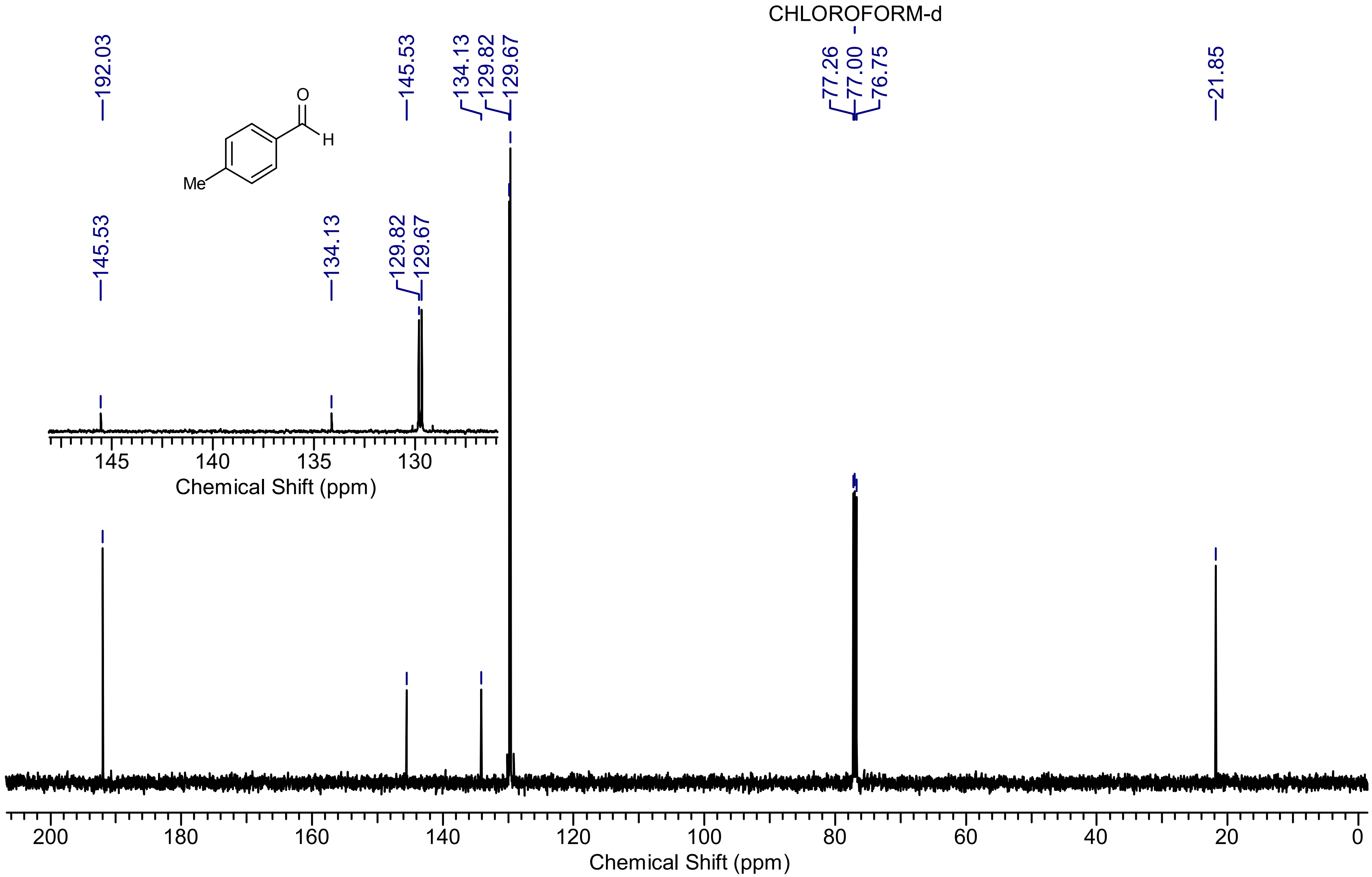

Supplementary Figure 53. ^{1}H NMR of 9a

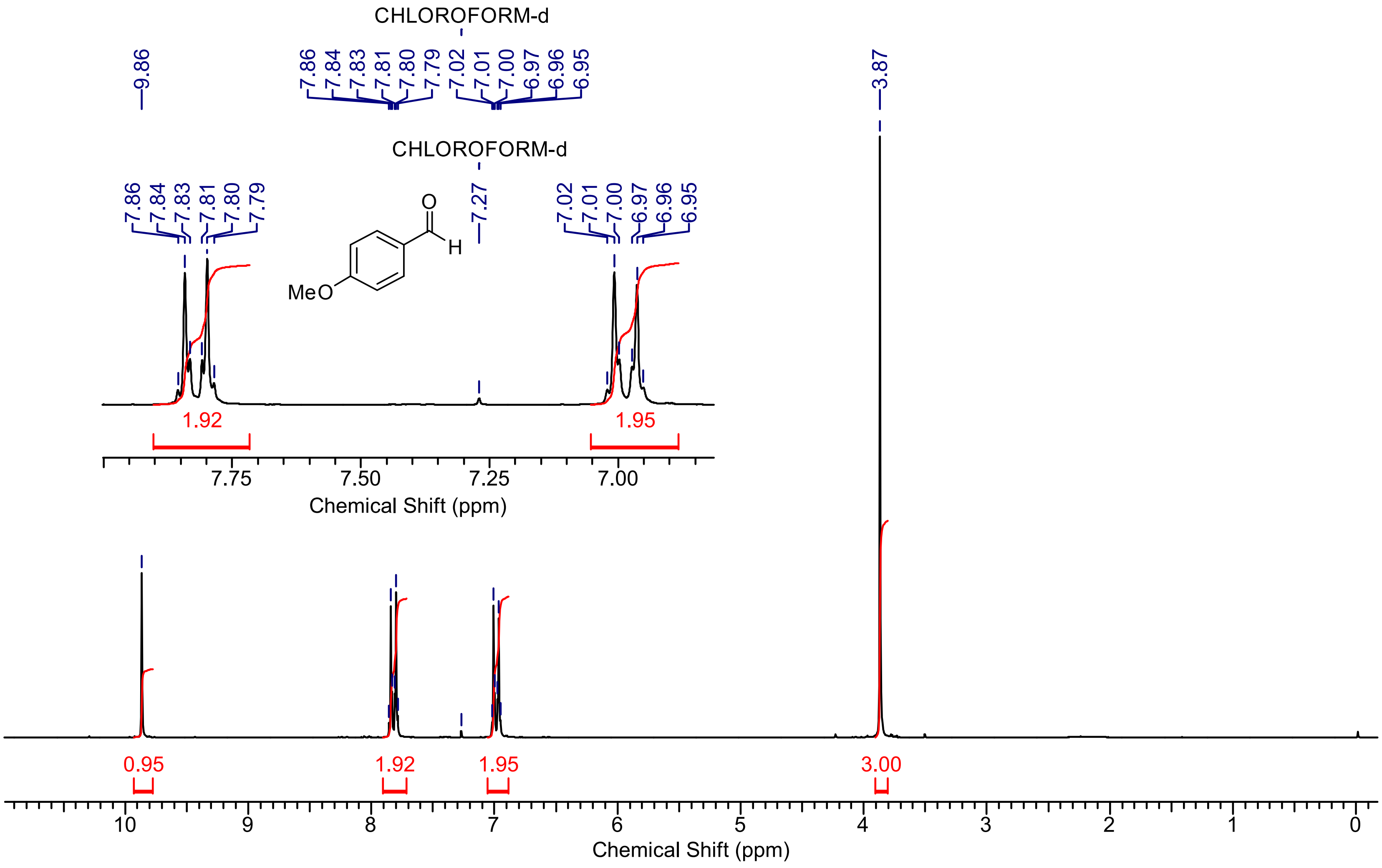

Supplementary Figure 54. ^{1}**H NMR of 9b**

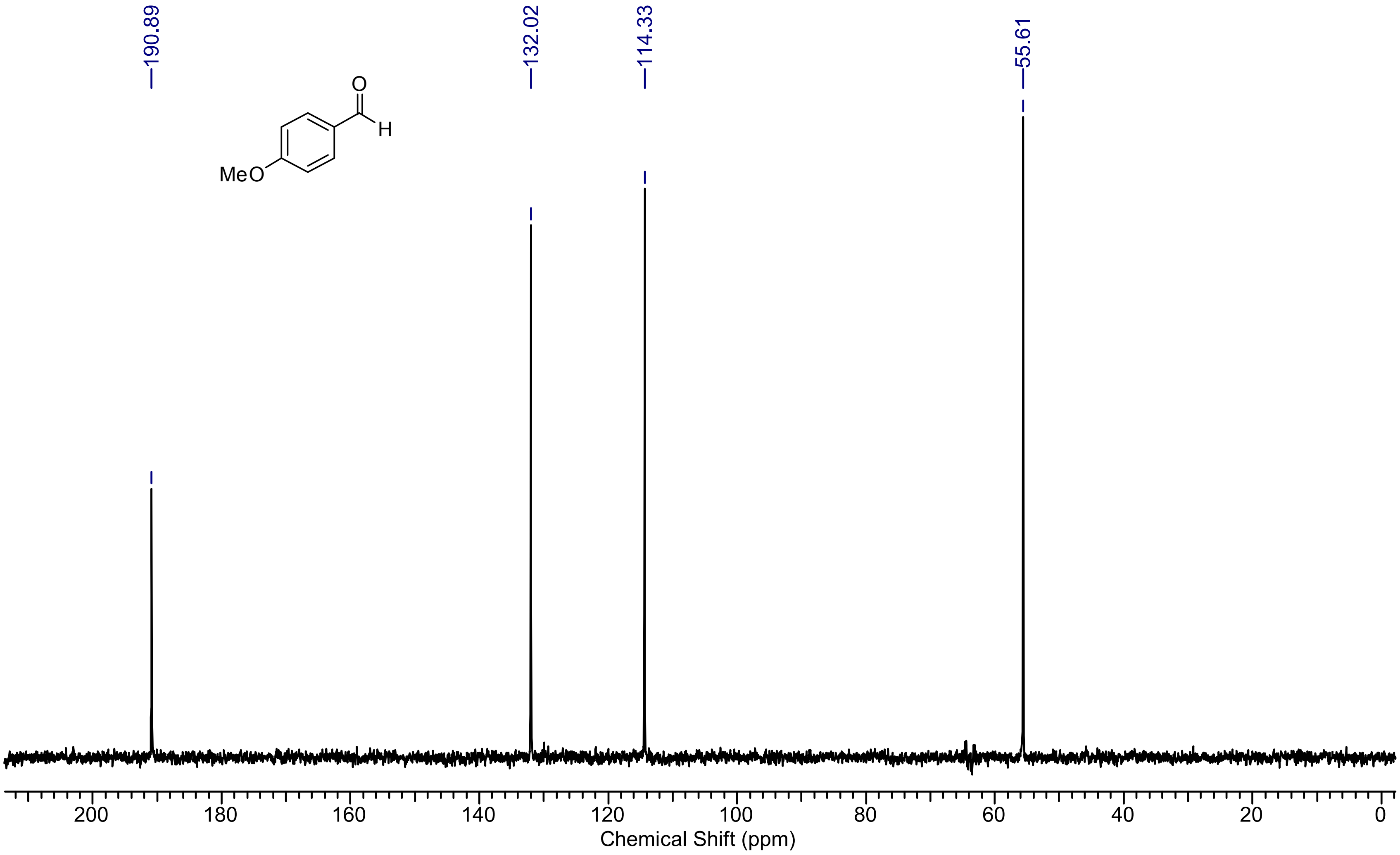

Supplementary Figure 55. ^{13}C NMR of 9b

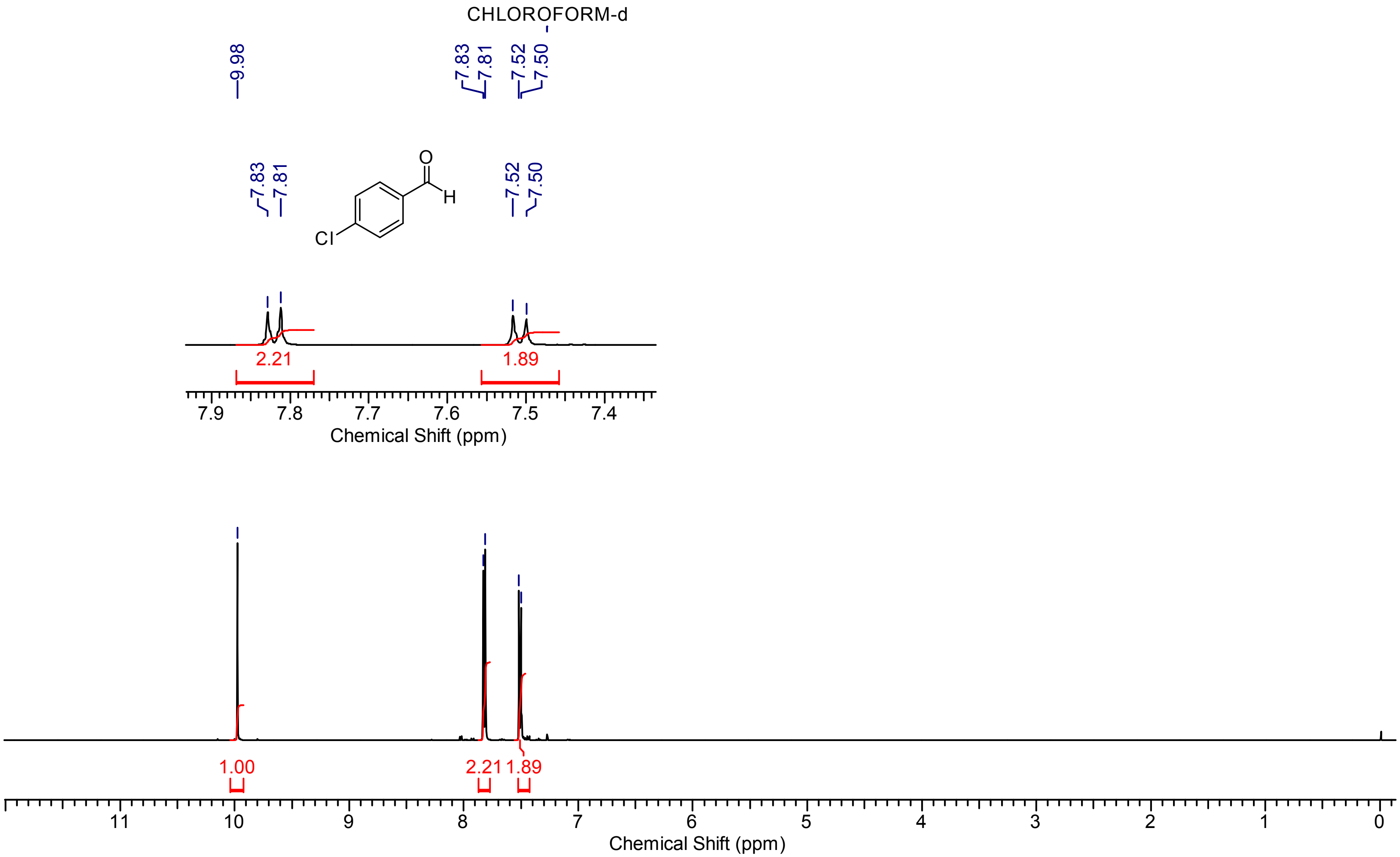

Supplementary Figure 56. ^{1}H NMR of 9c

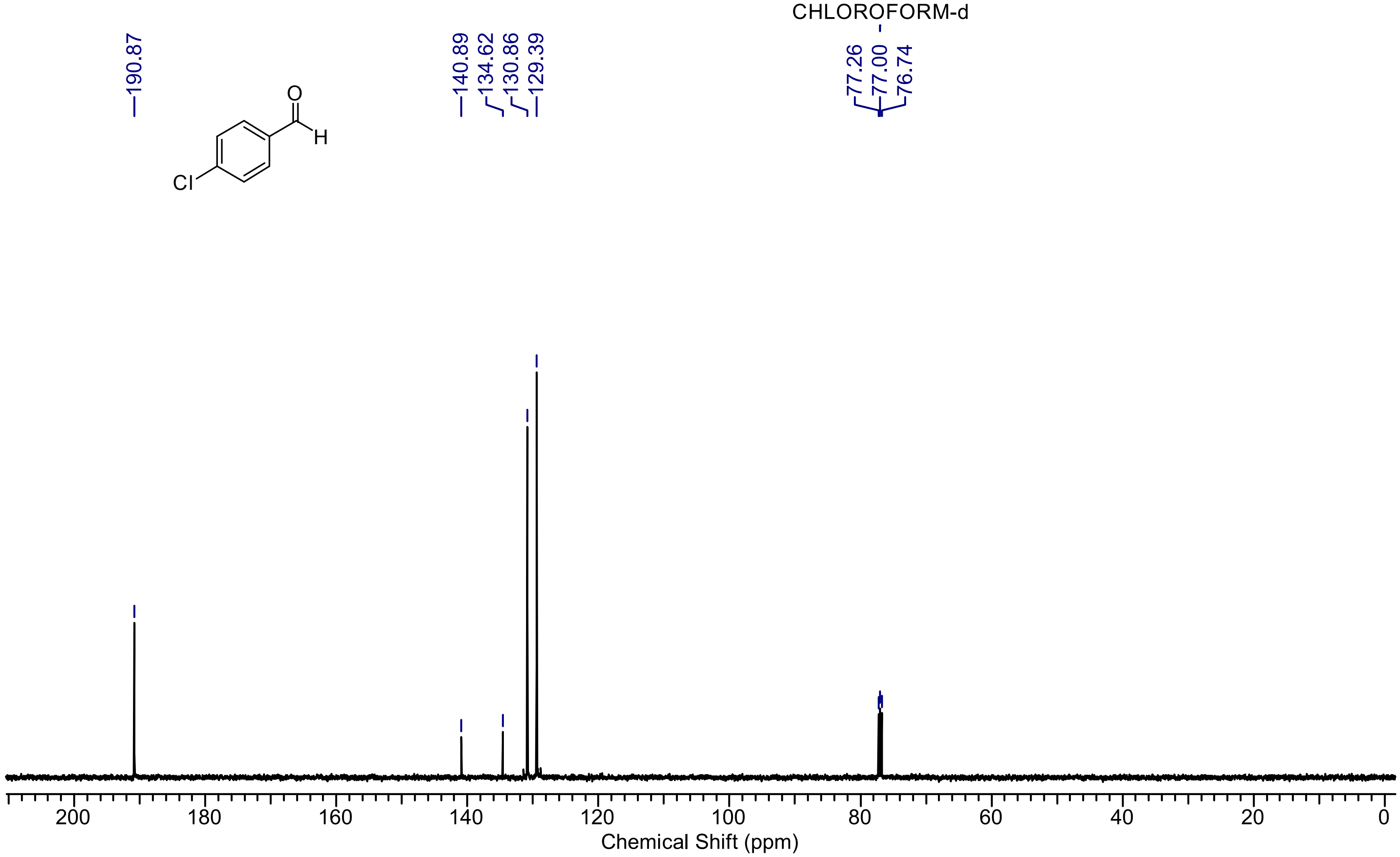

Supplementary Figure 57. ^{13}C NMR of 9c

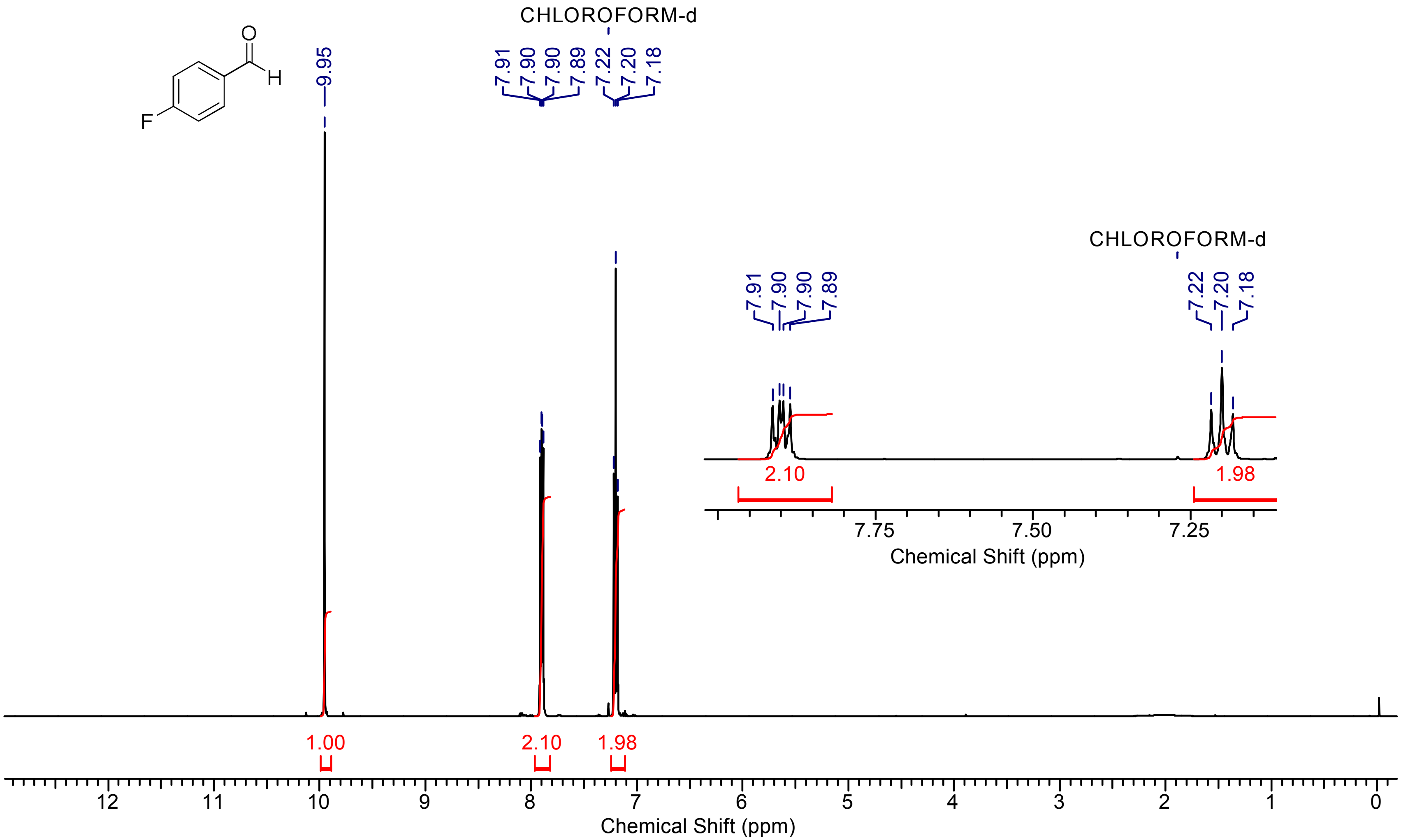

Supplementary Figure 58. ^{1}H NMR of 9d

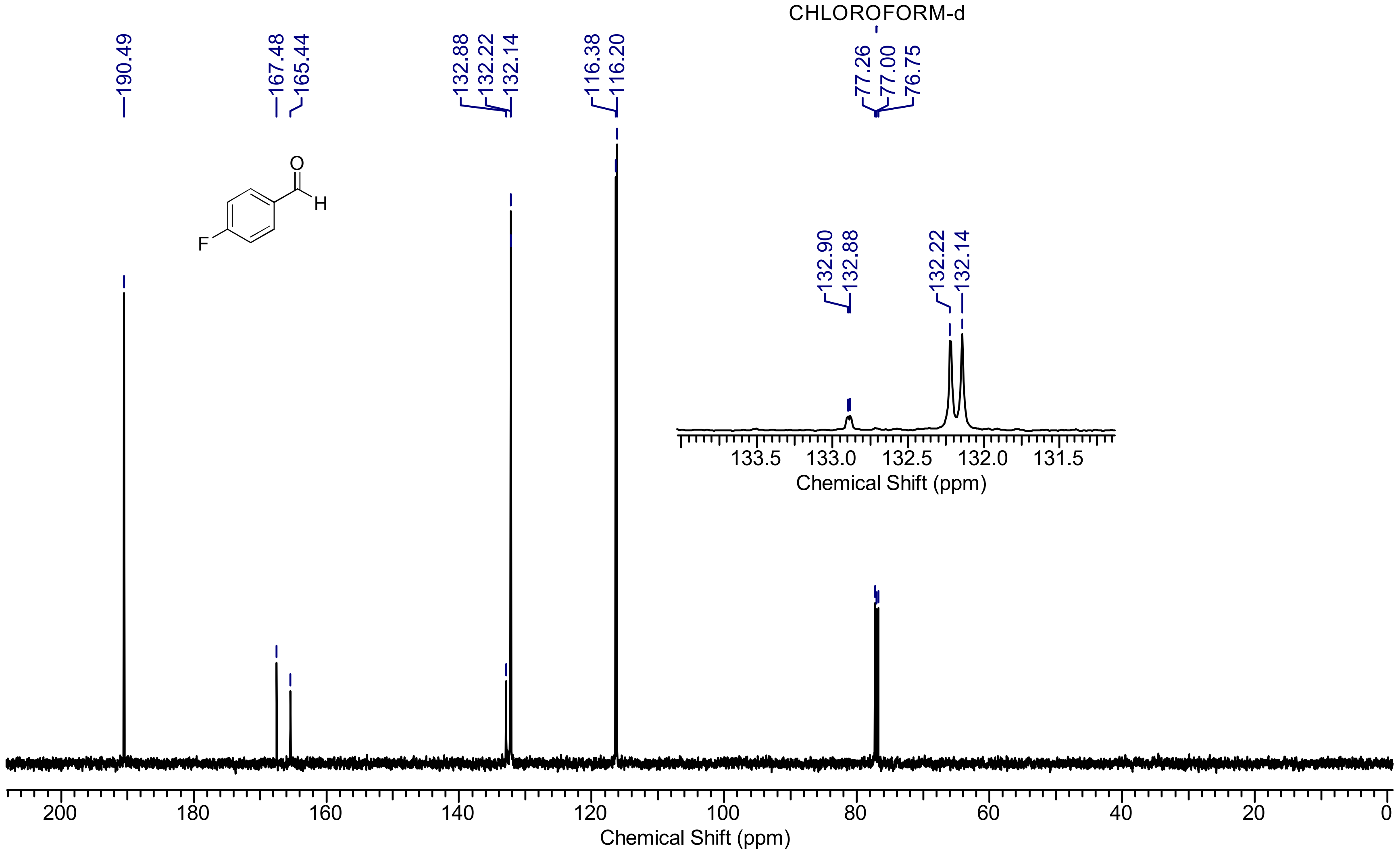

Supplementary Figure 59. ^{13}C NMR of 9d

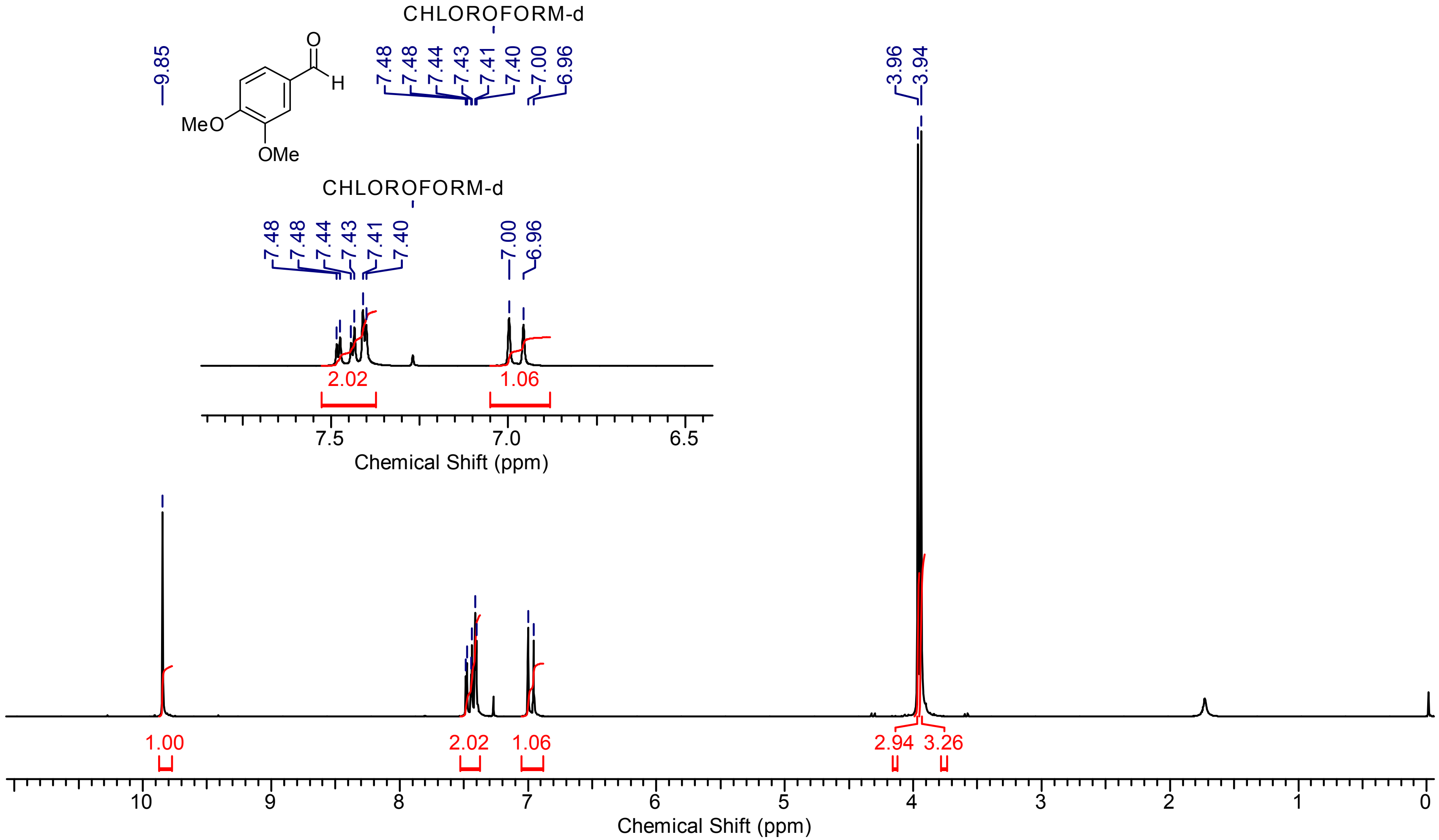

Supplementary Figure 60. ^{1}H NMR of 9e

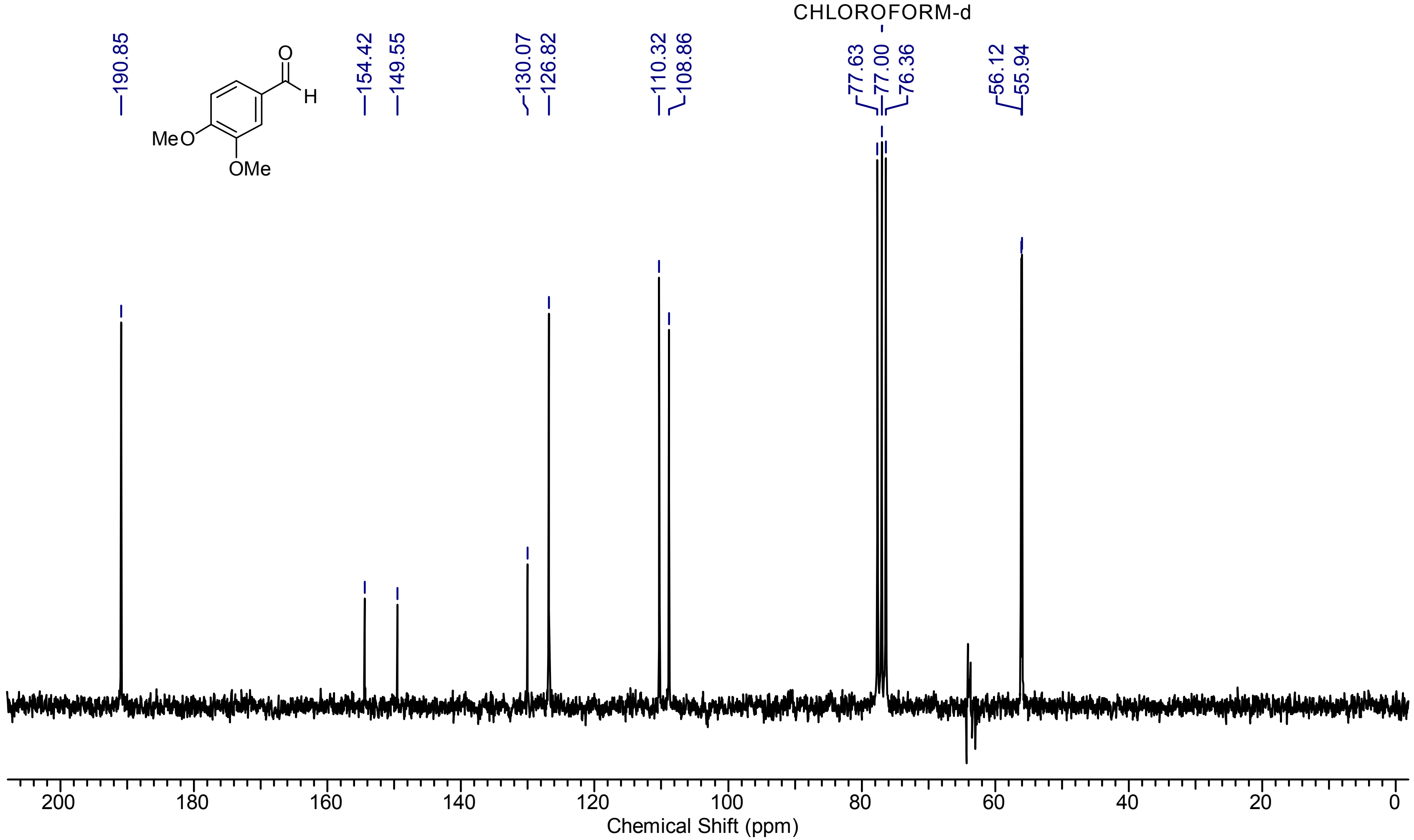

Supplementary Figure 61. ^{13}**C NMR of 9e**

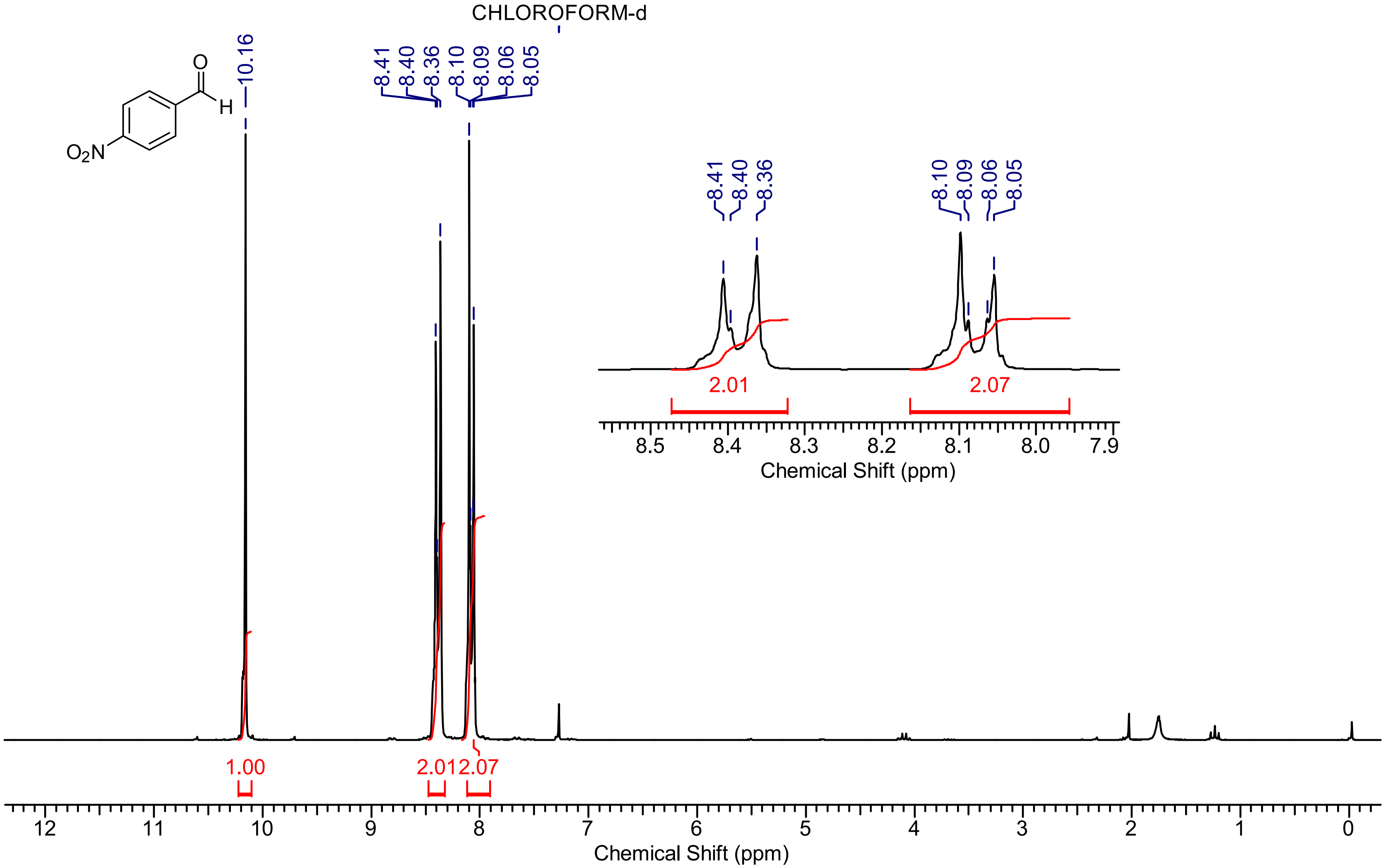

Supplementary Figure 62. ^{1}H NMR of 9f

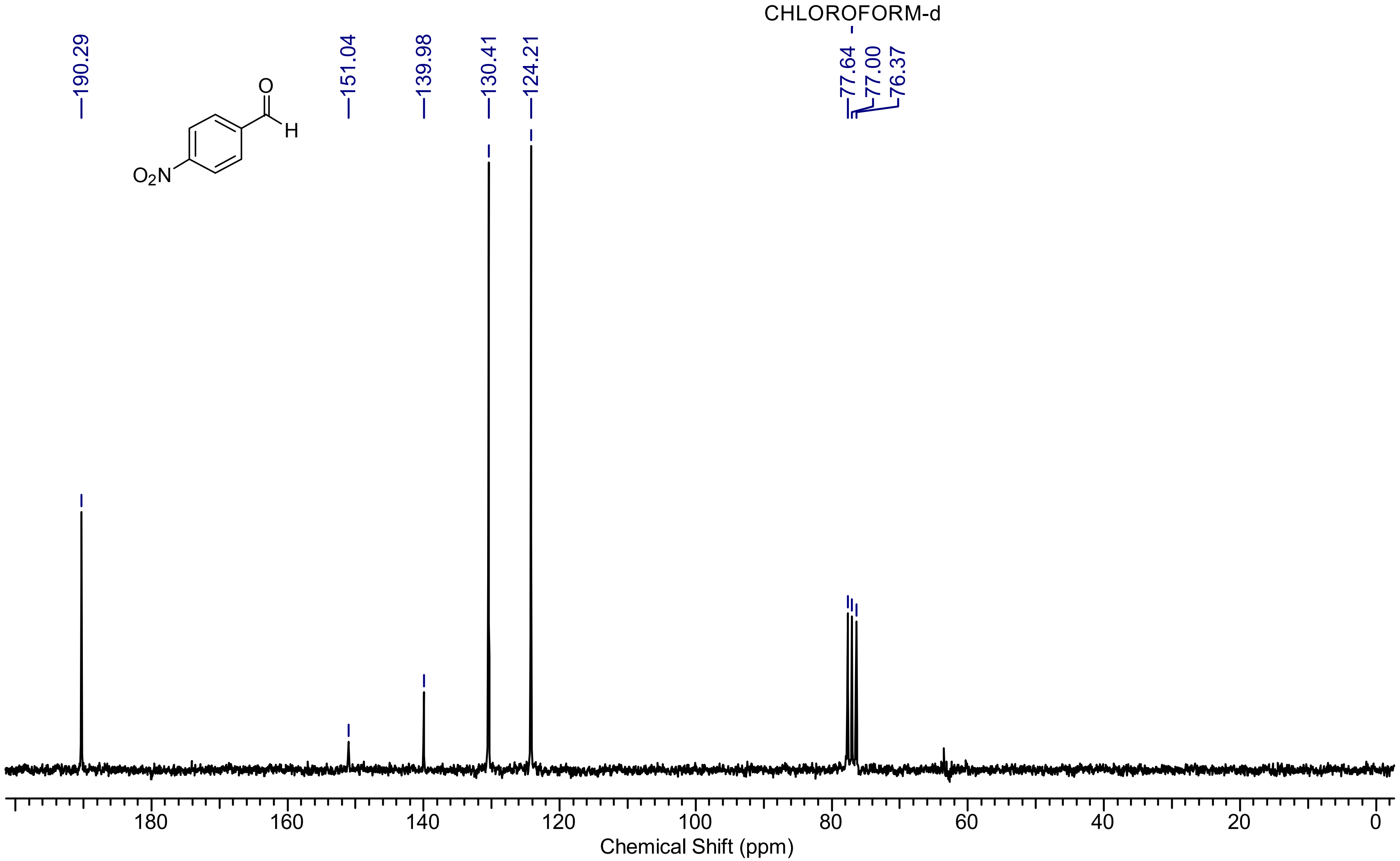

Supplementary Figure 63. ^{13}C NMR of 9f

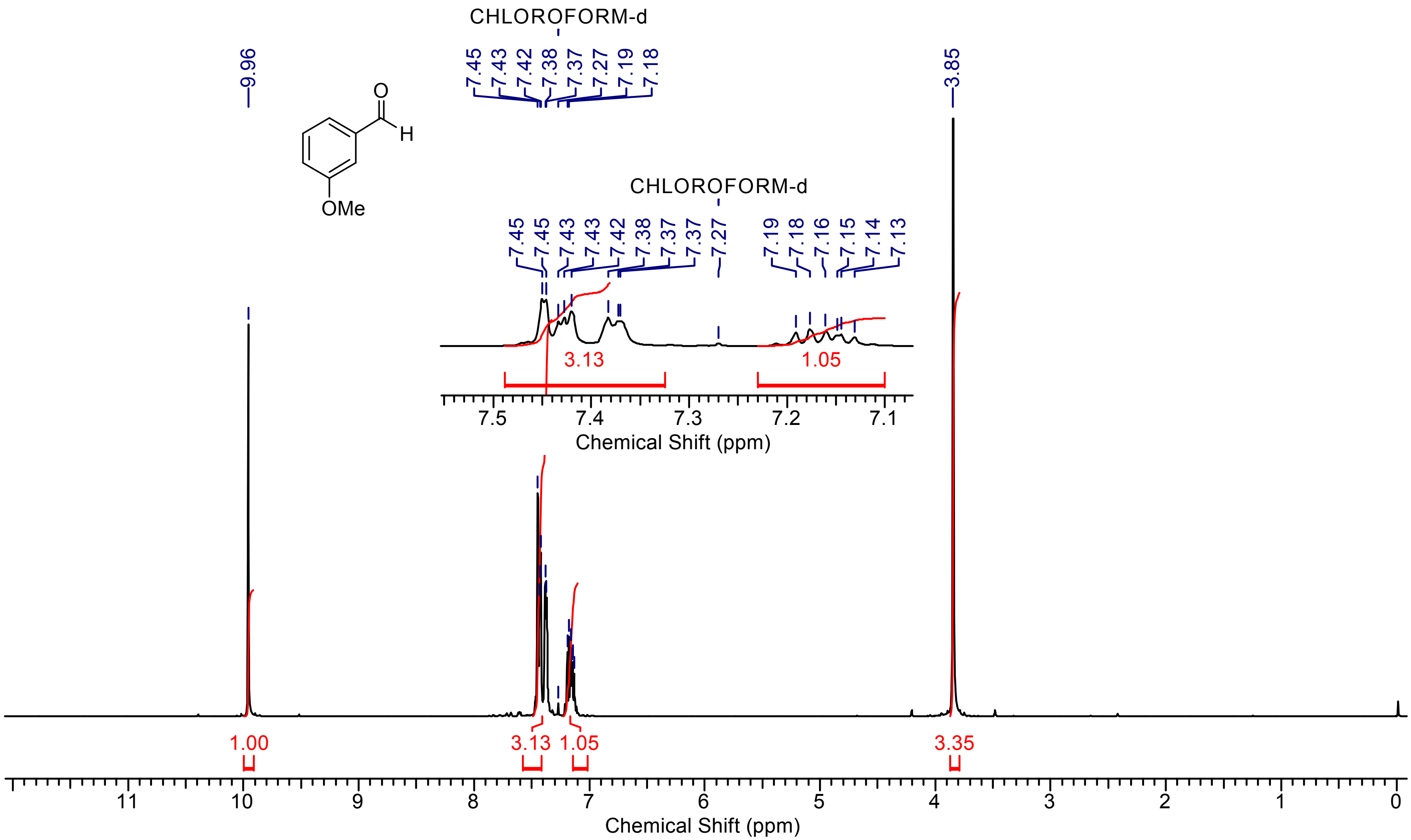

Supplementary Figure 64. ^{1}H NMR of 9h

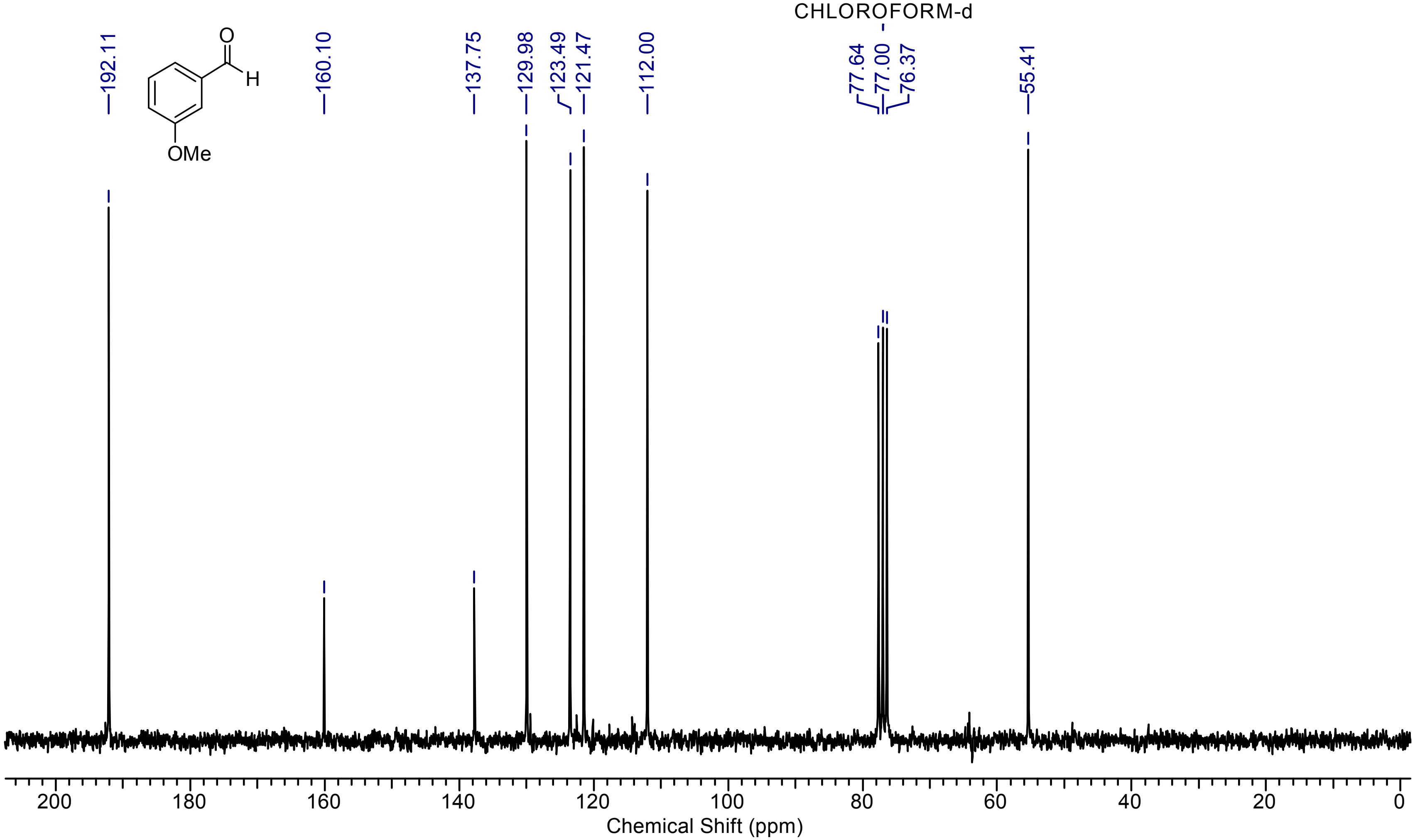

Supplementary Figure 65. ^{13}C NMR of 9h

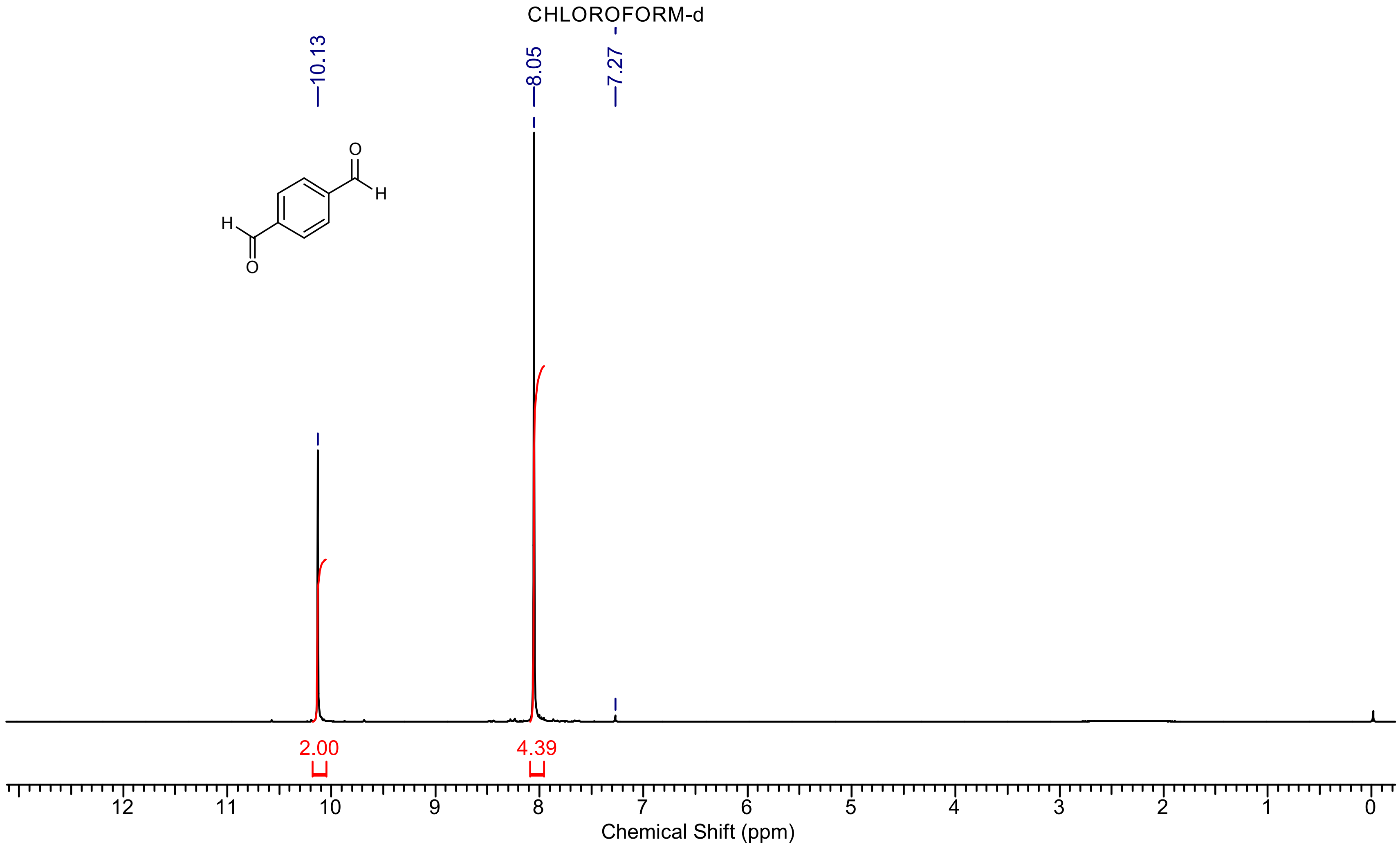

Supplementary Figure 66. ^{1}H NMR of 9i

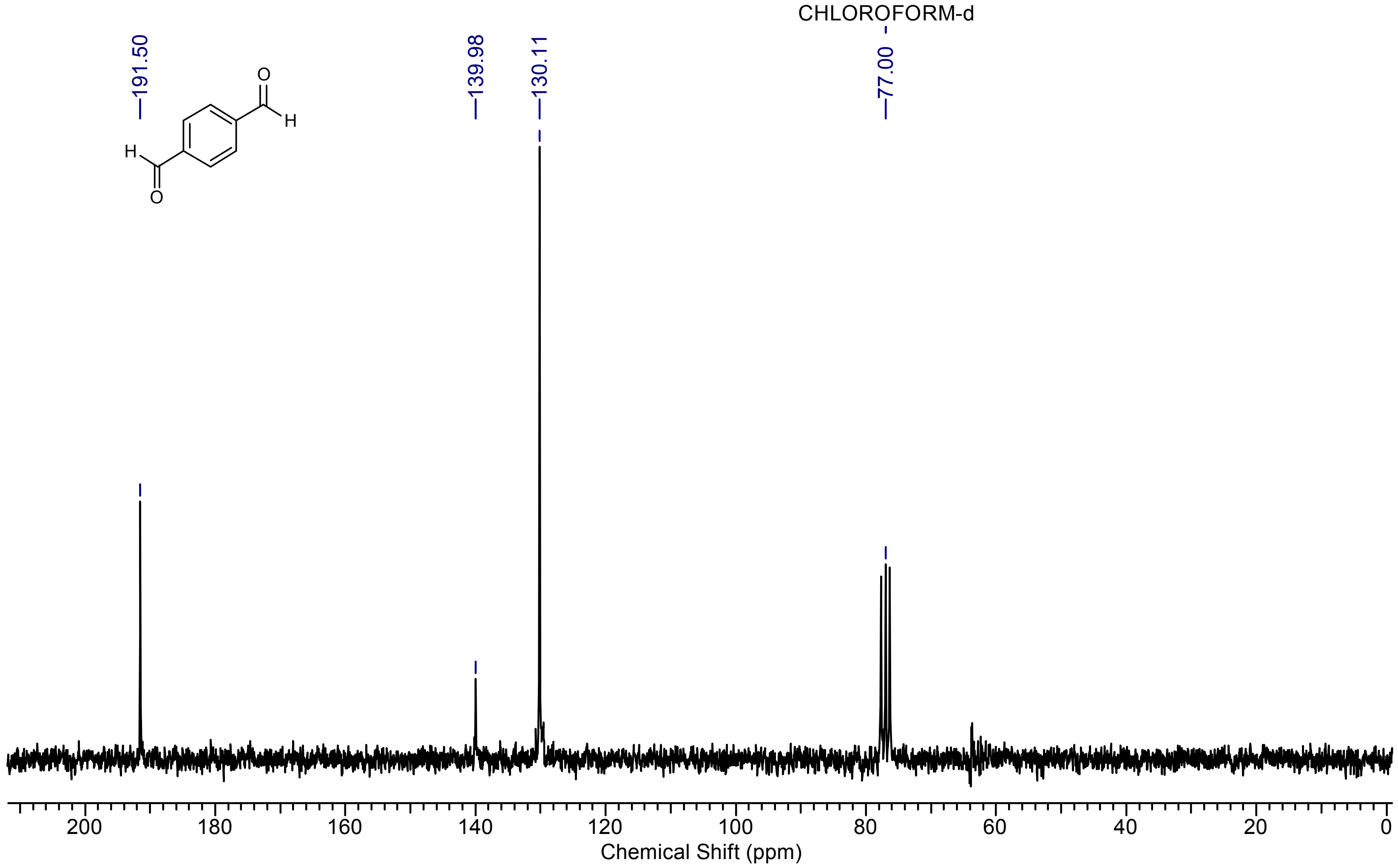

Supplementary Figure 67. ^{13}C NMR of 9i

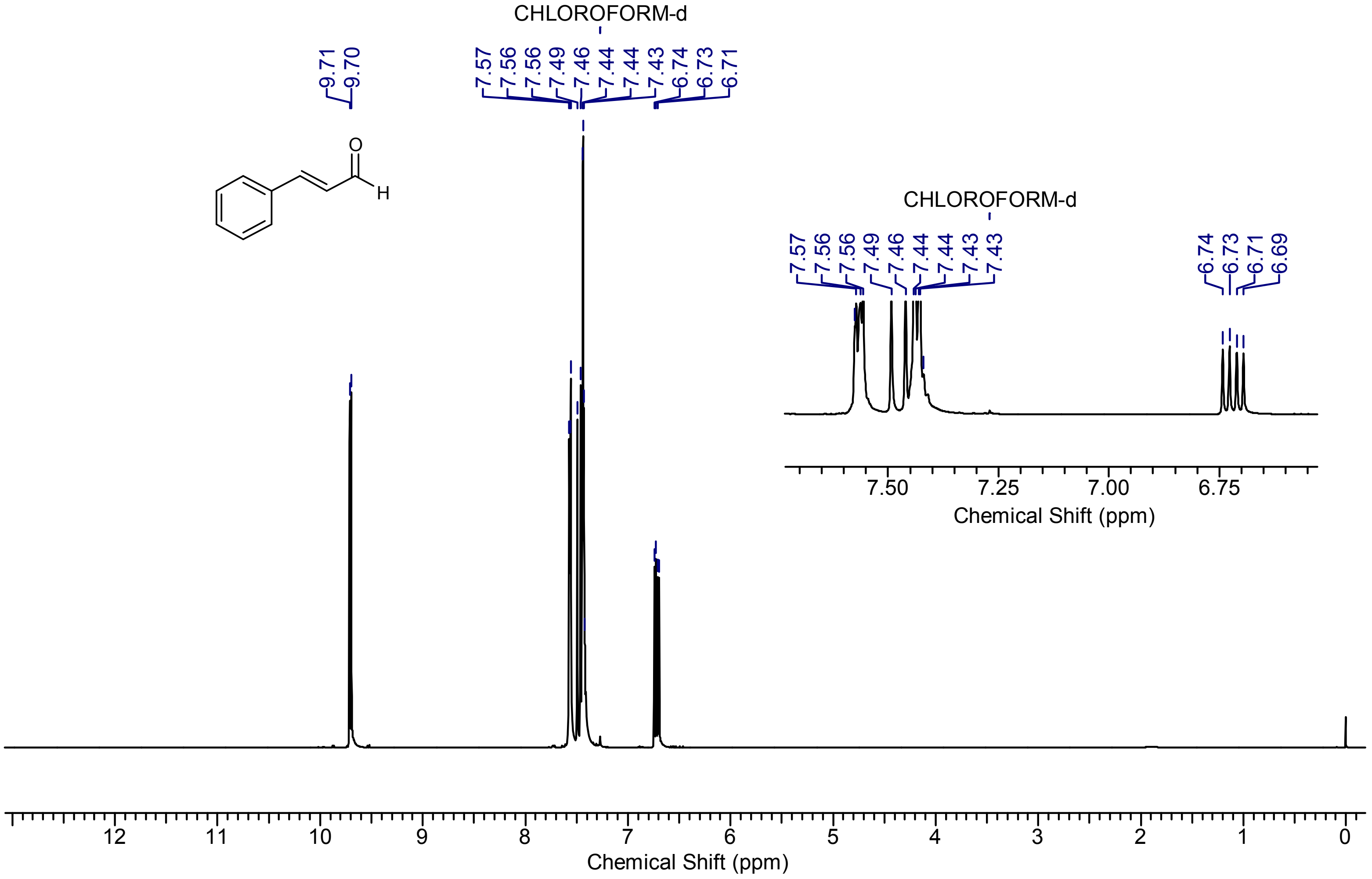

Supplementary Figure 68. ^{1}H NMR of 9j

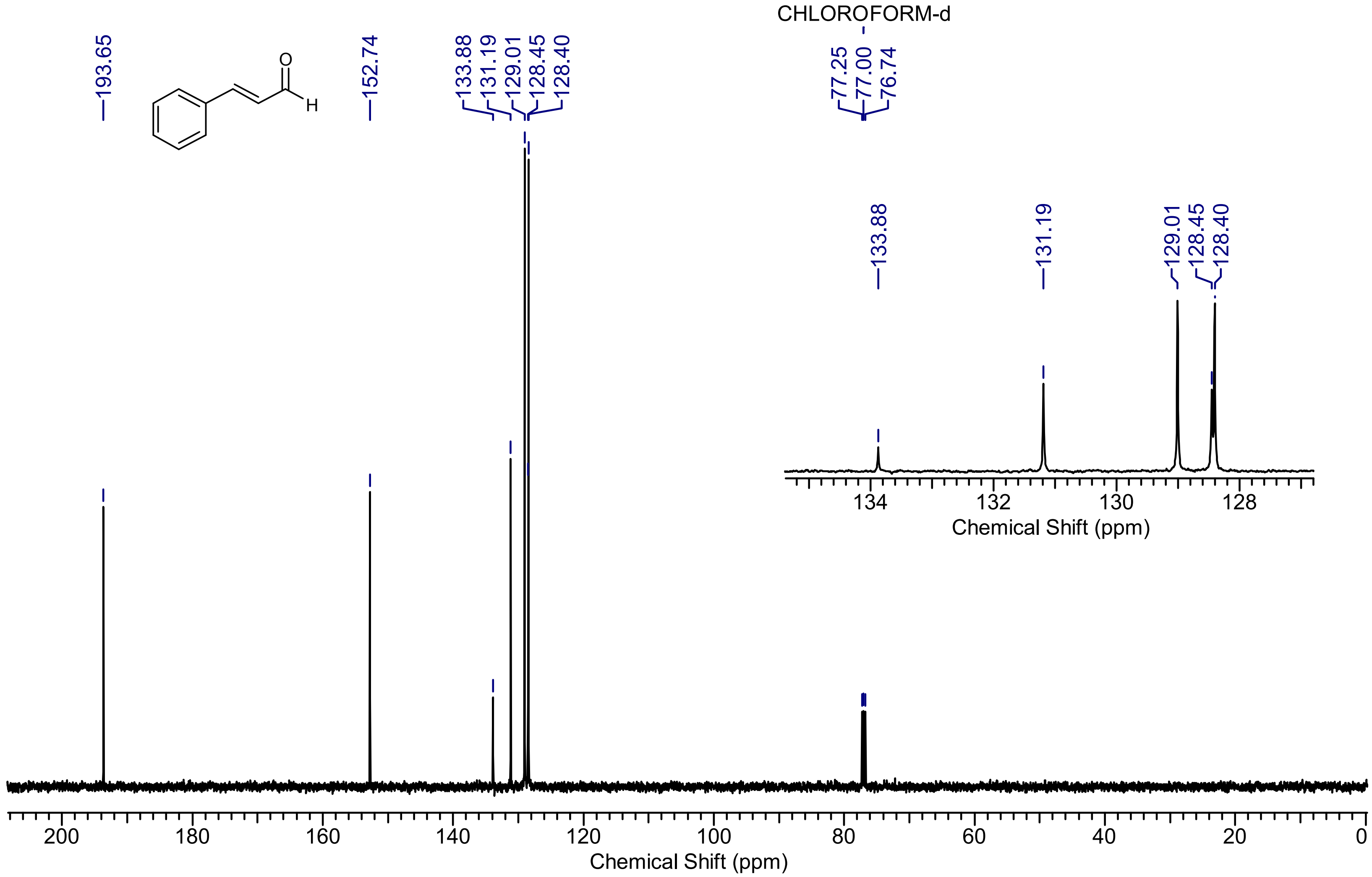

Supplementary Figure 69. ^{13}C NMR of 9j

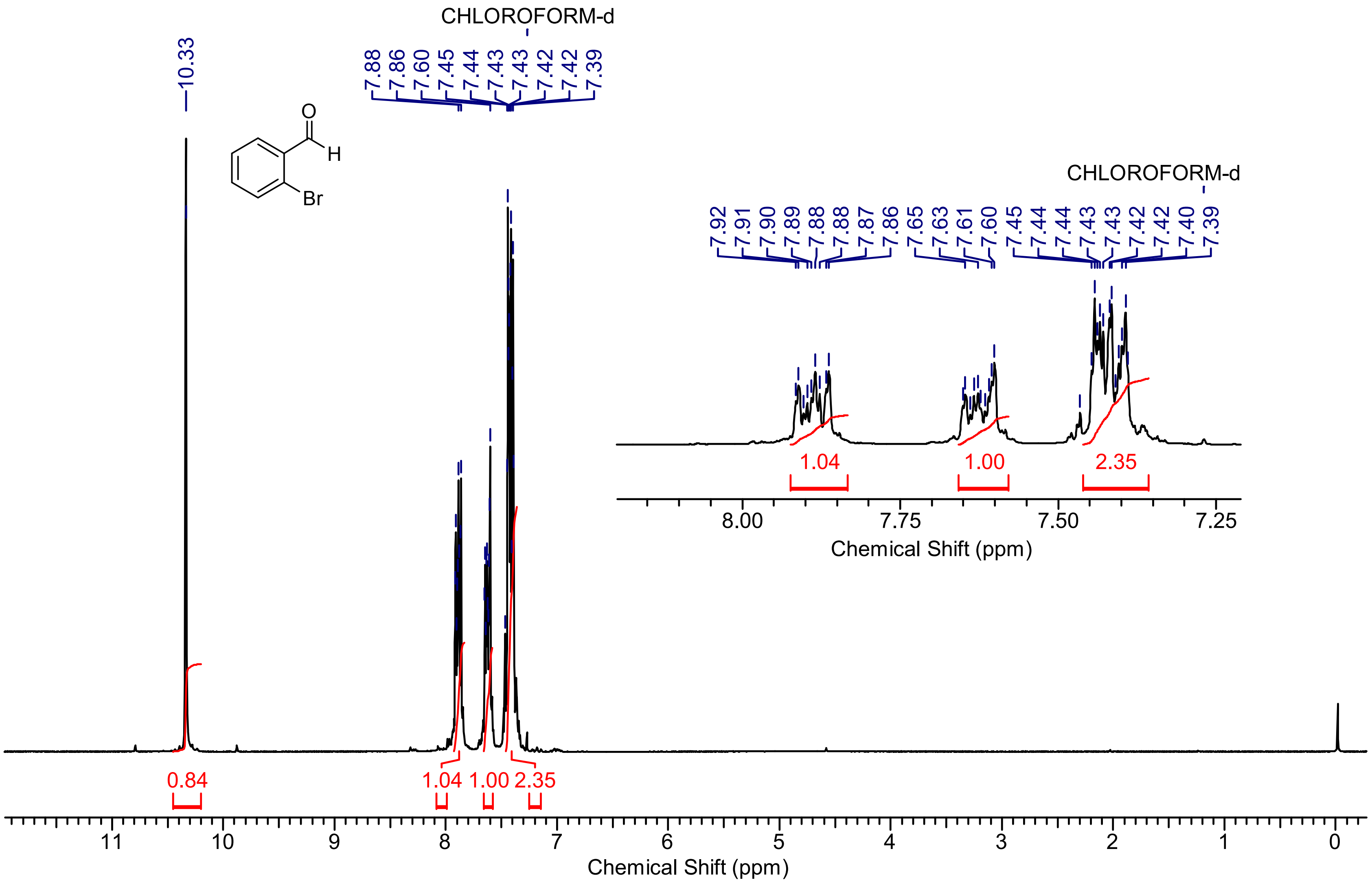

Supplementary Figure 70. ^{1}H NMR of 9k

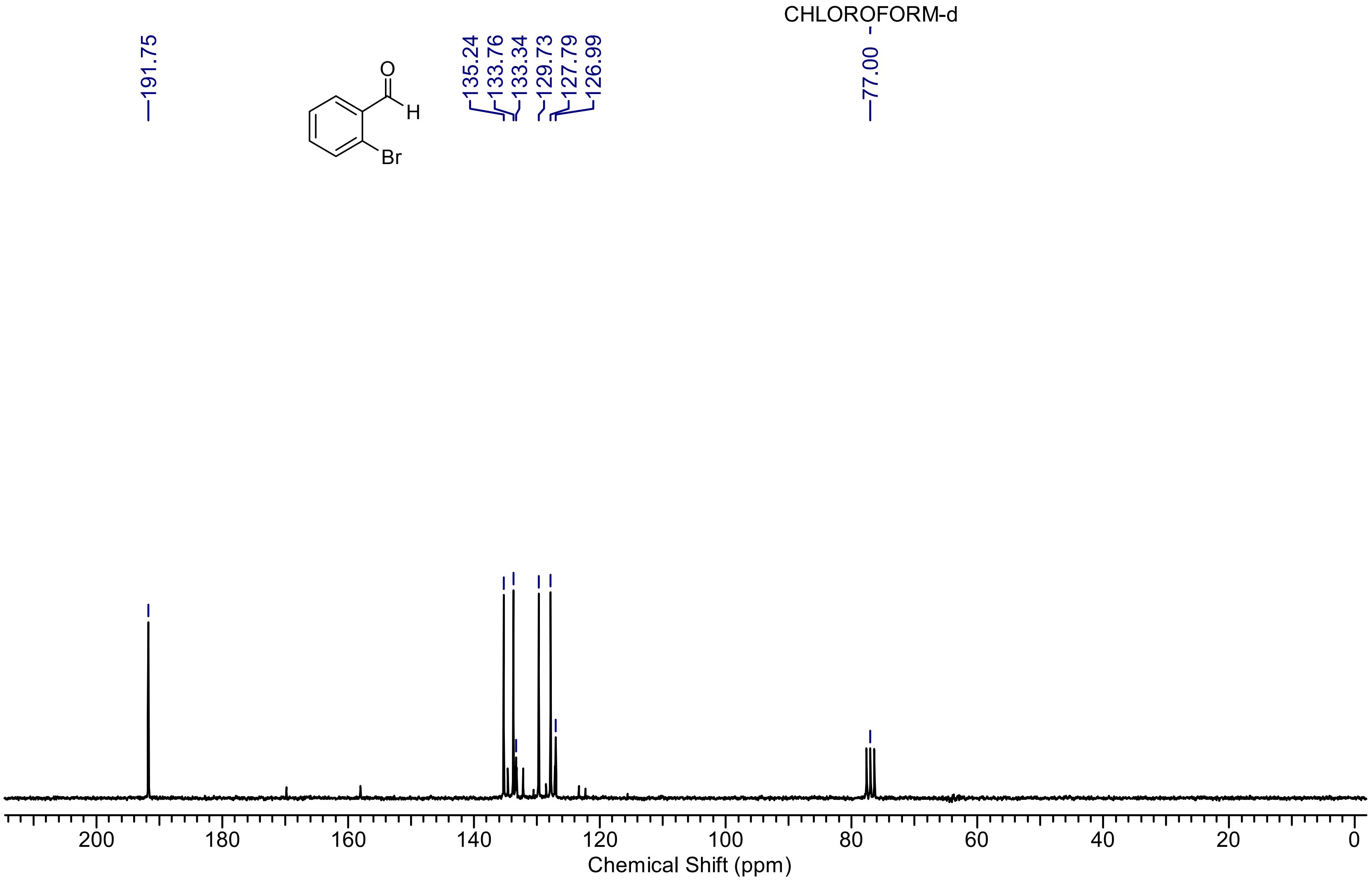

Supplementary Figure 71. ^{13}C NMR of 9k

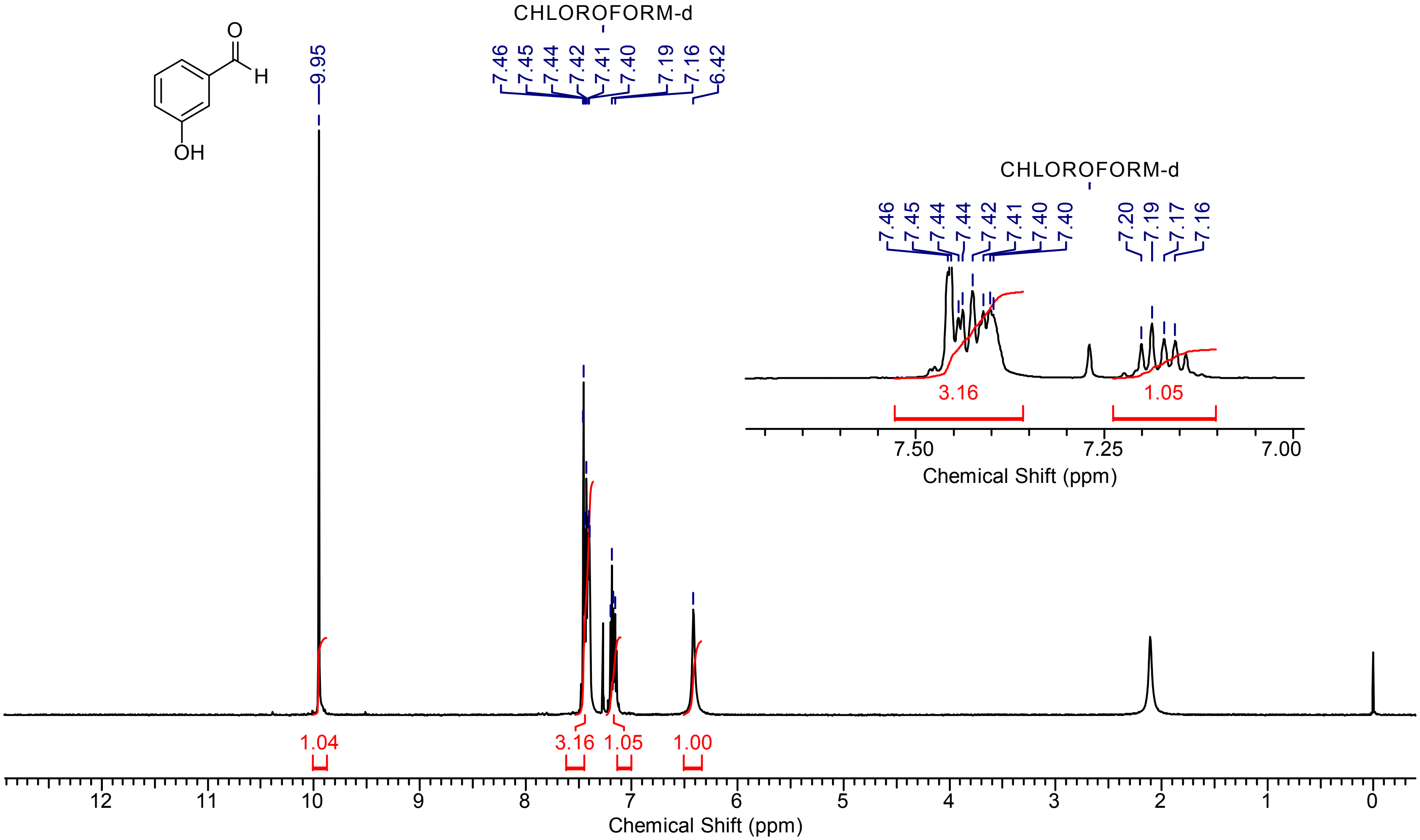

Supplementary Figure 72. ^{1}H NMR of 9n

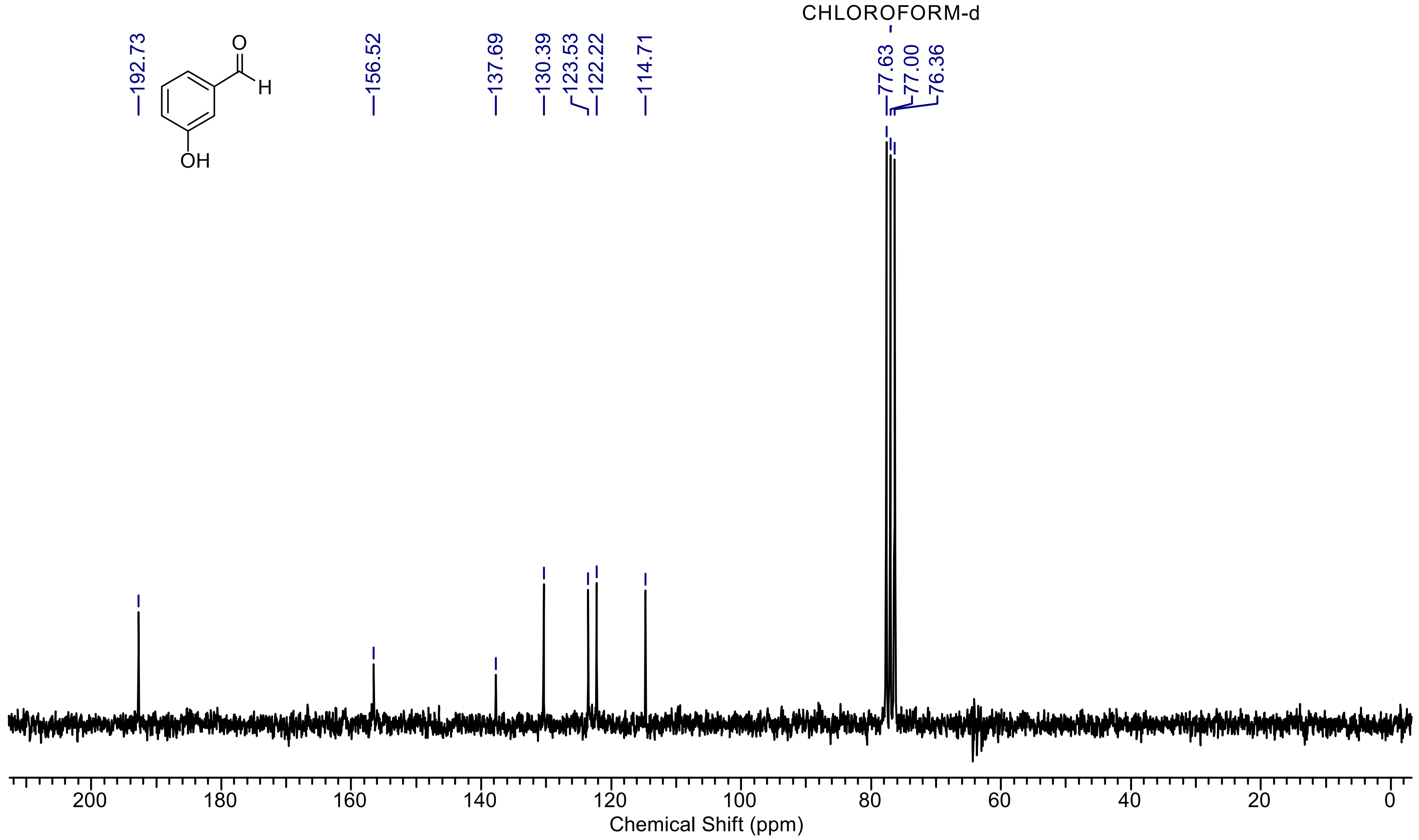

Supplementary Figure 73. ^{13}**C NMR of 9n**

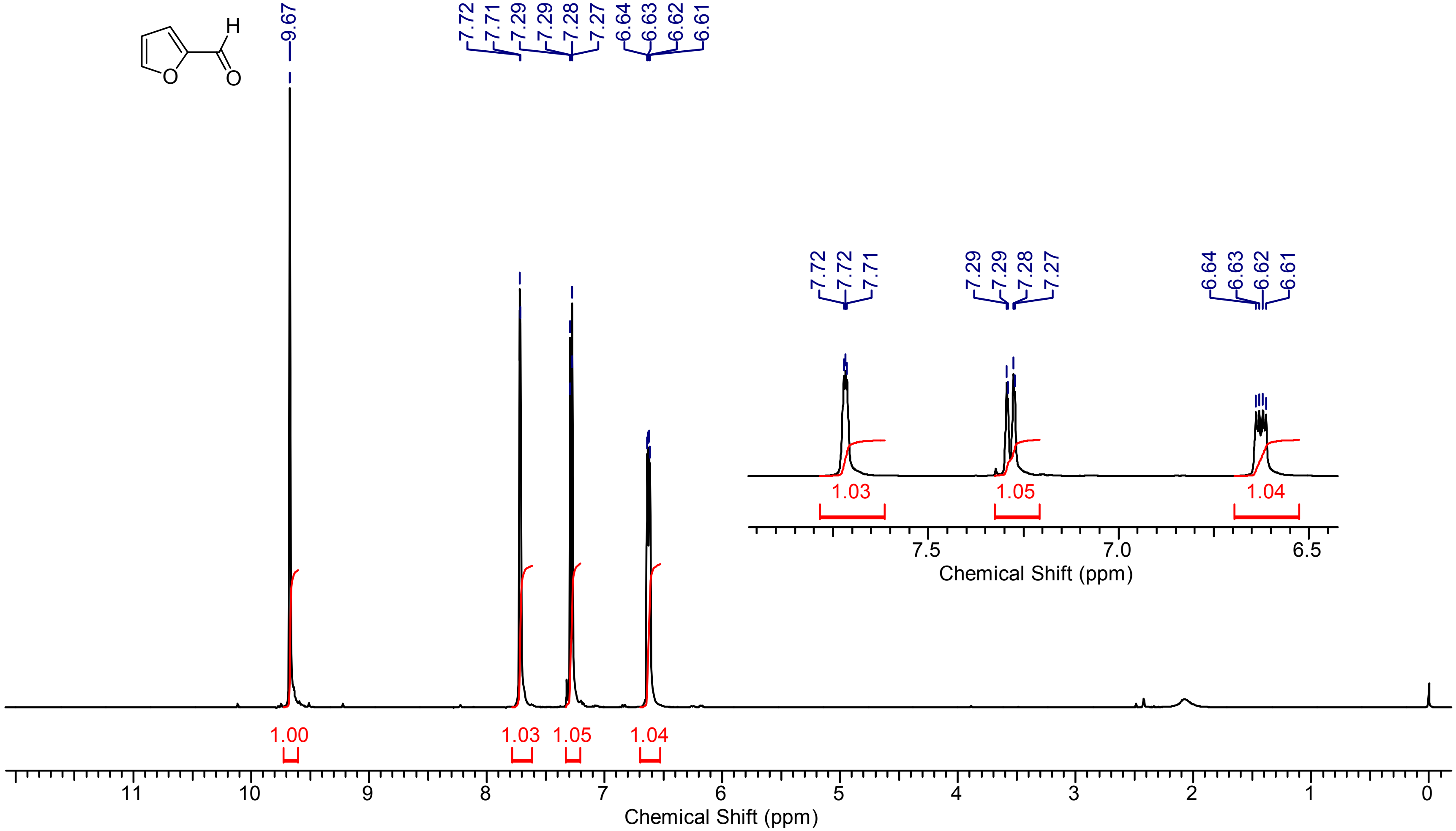

Supplementary Figure 74. ^{1}H NMR of 9o

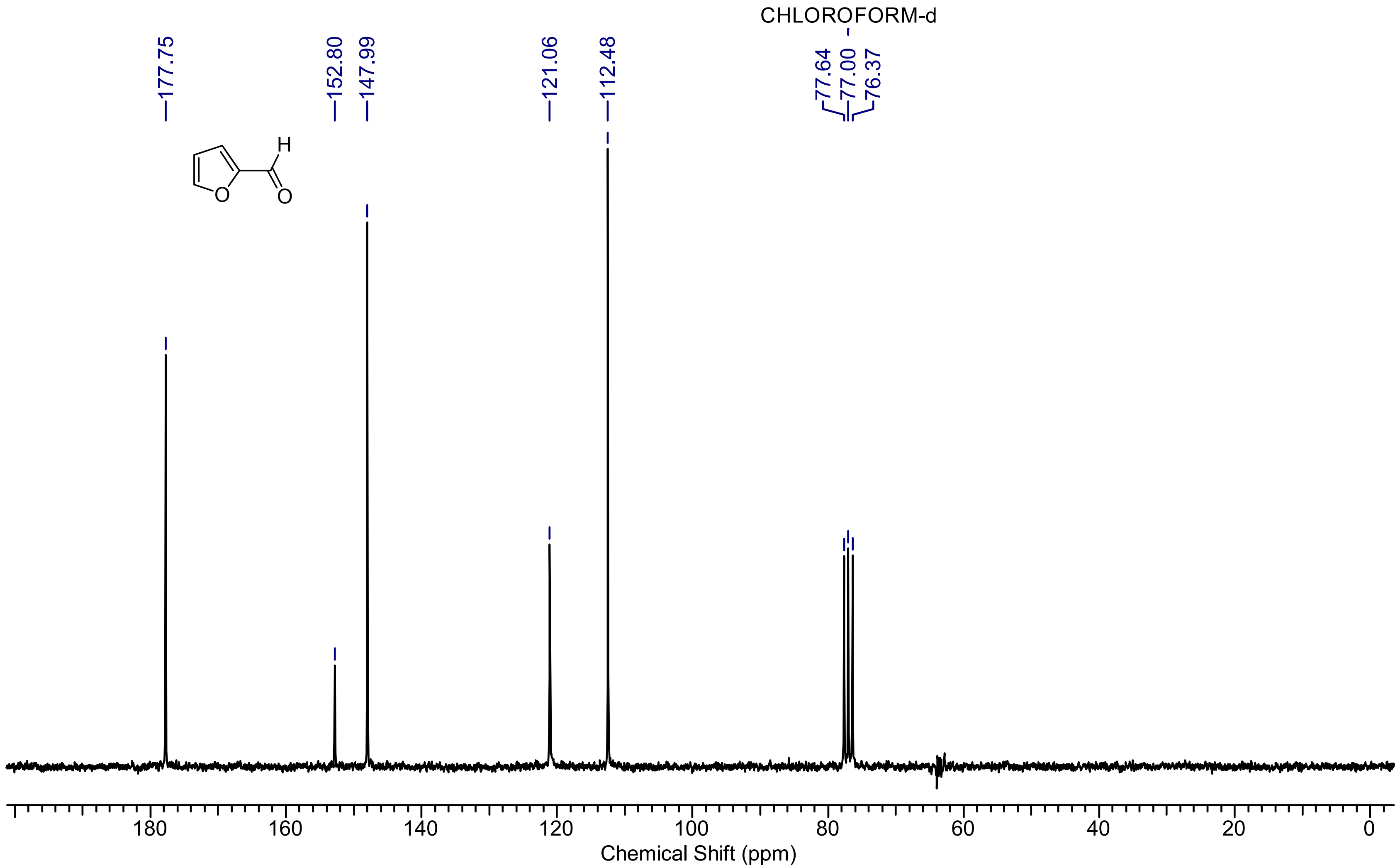

Supplementary Figure 75. ^{13}C NMR of 9o

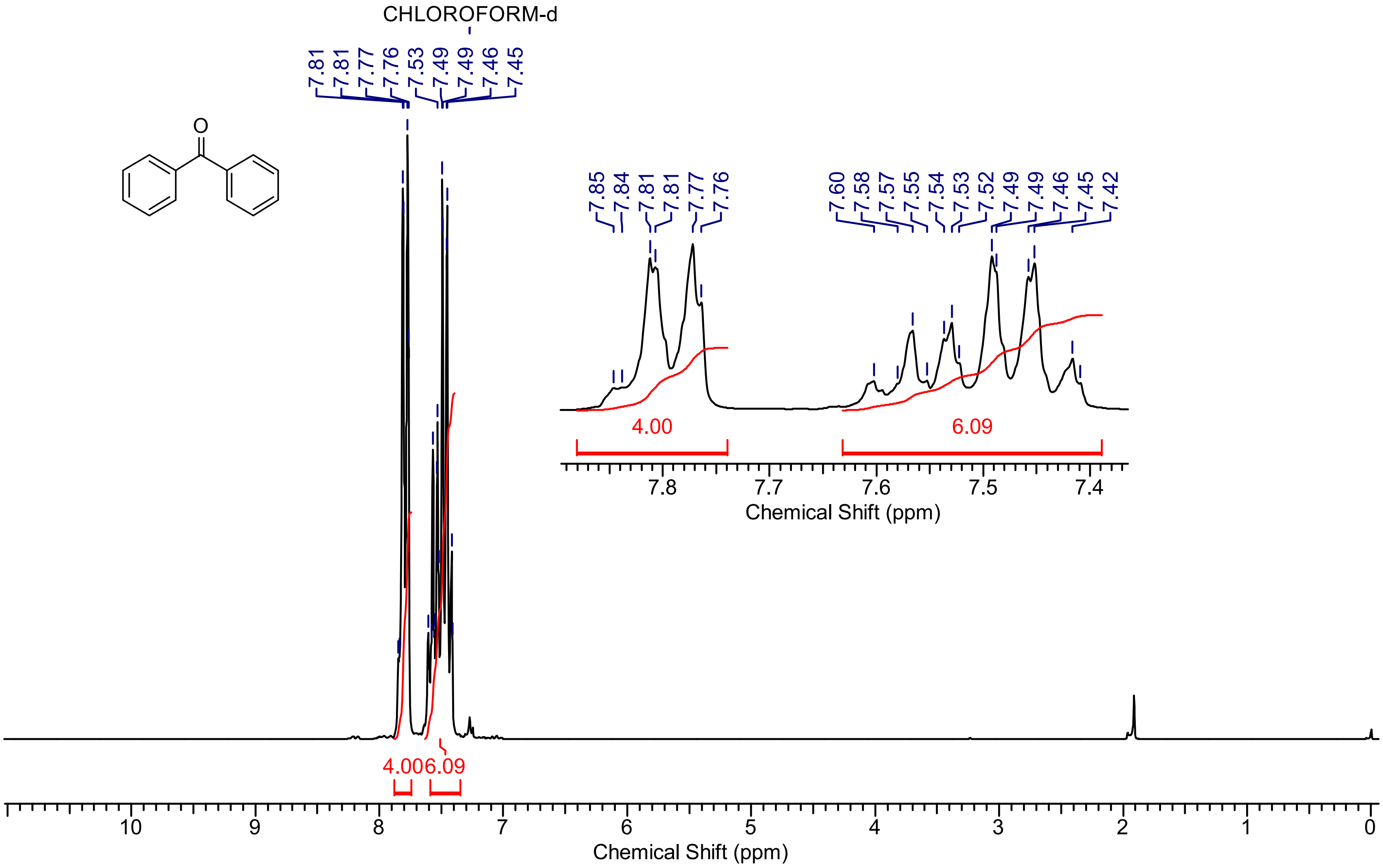

Supplementary Figure 76. ^{1}H NMR of 11a

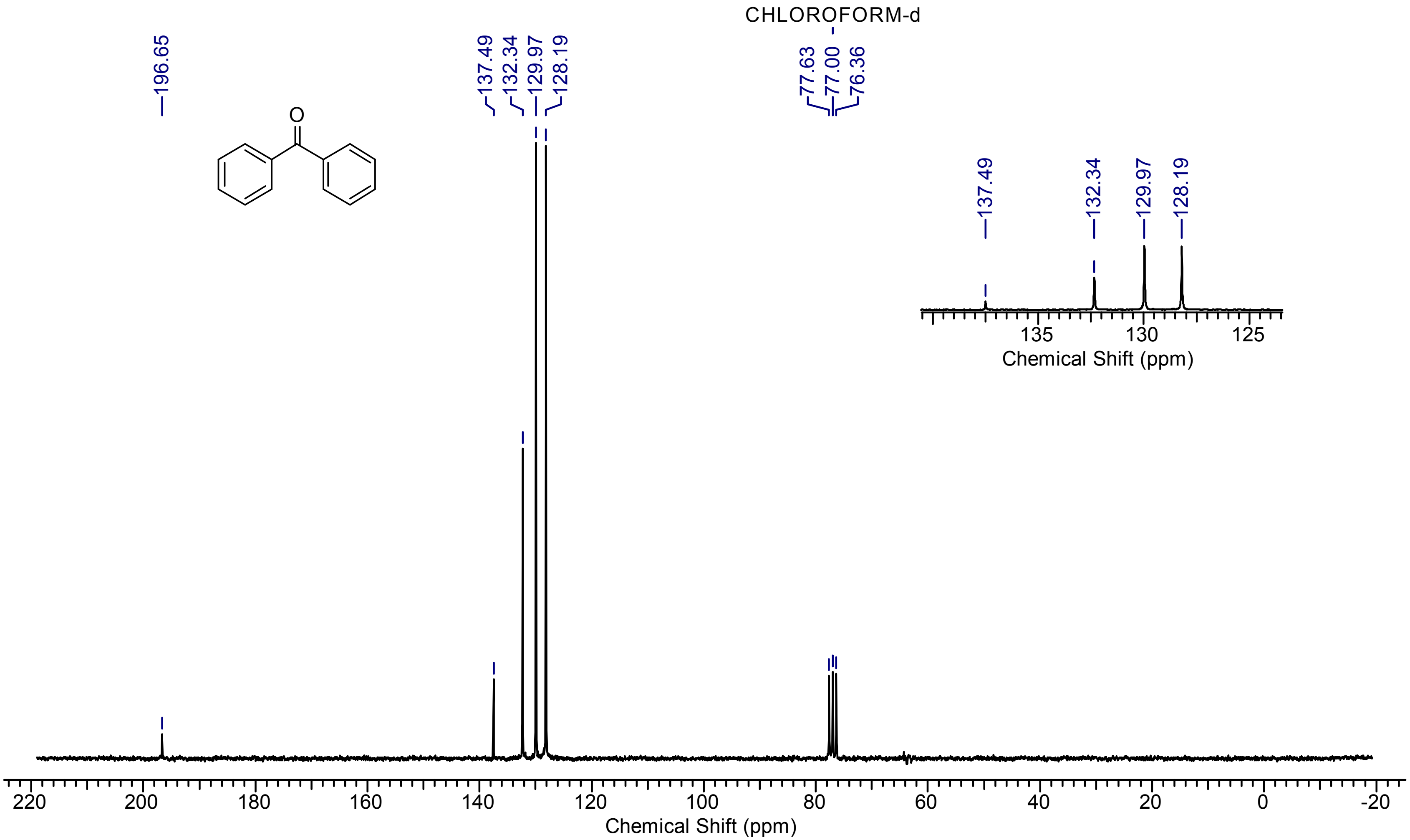

Supplementary Figure 77. ^{13}C NMR of 11a

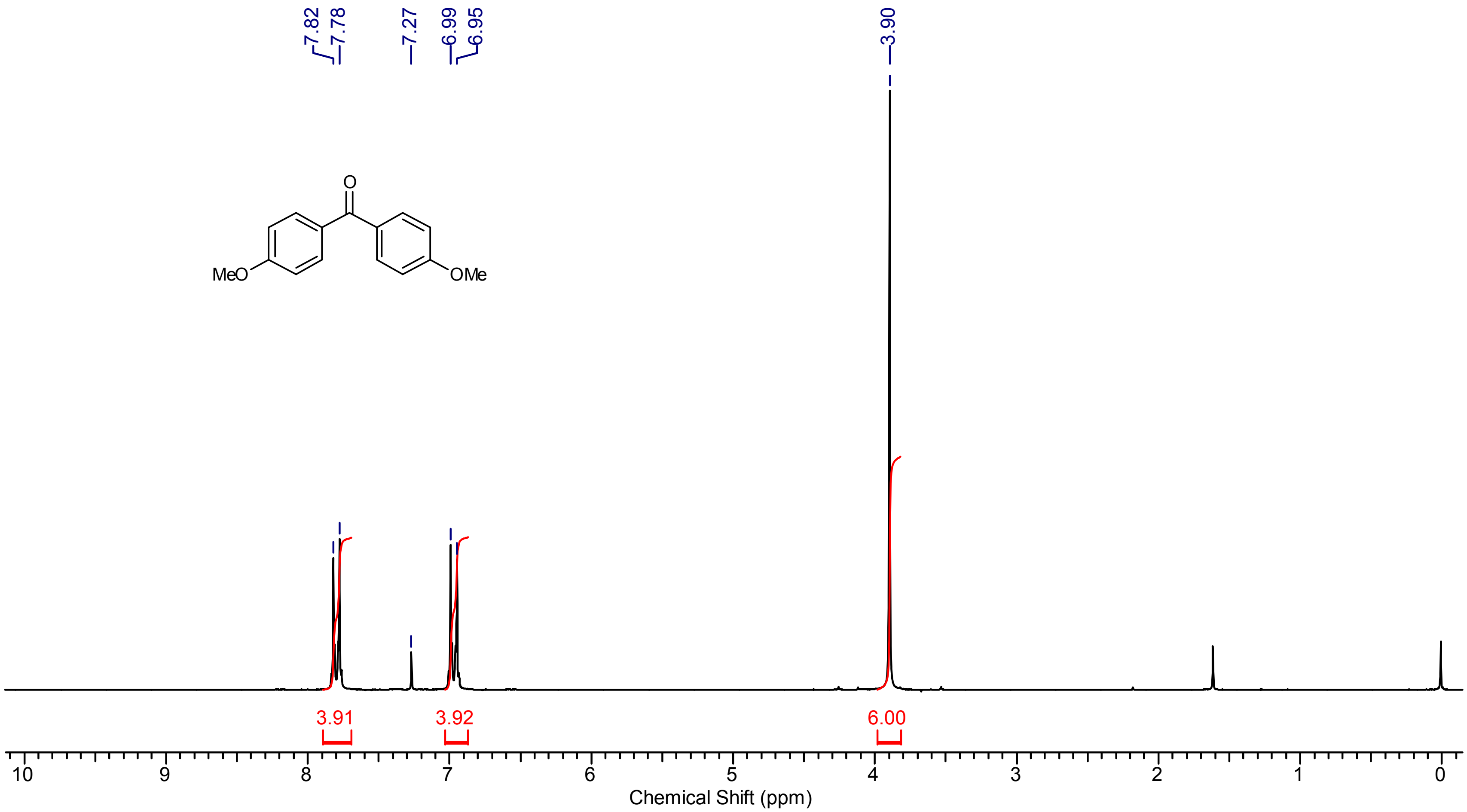

Supplementary Figure 78. **^{1}H NMR of 11b**

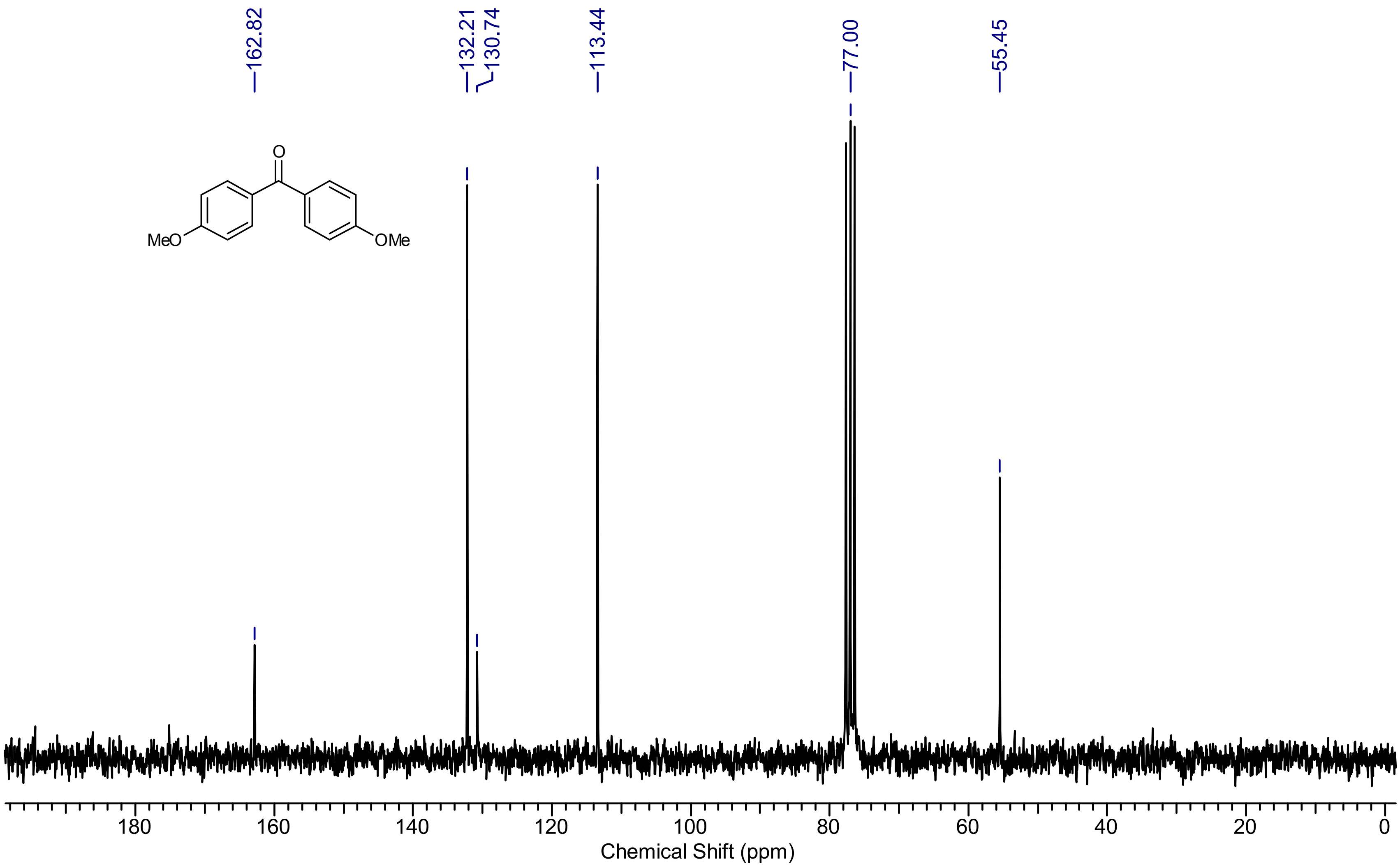

Supplementary Figure 79. ^{13}C NMR of 11b

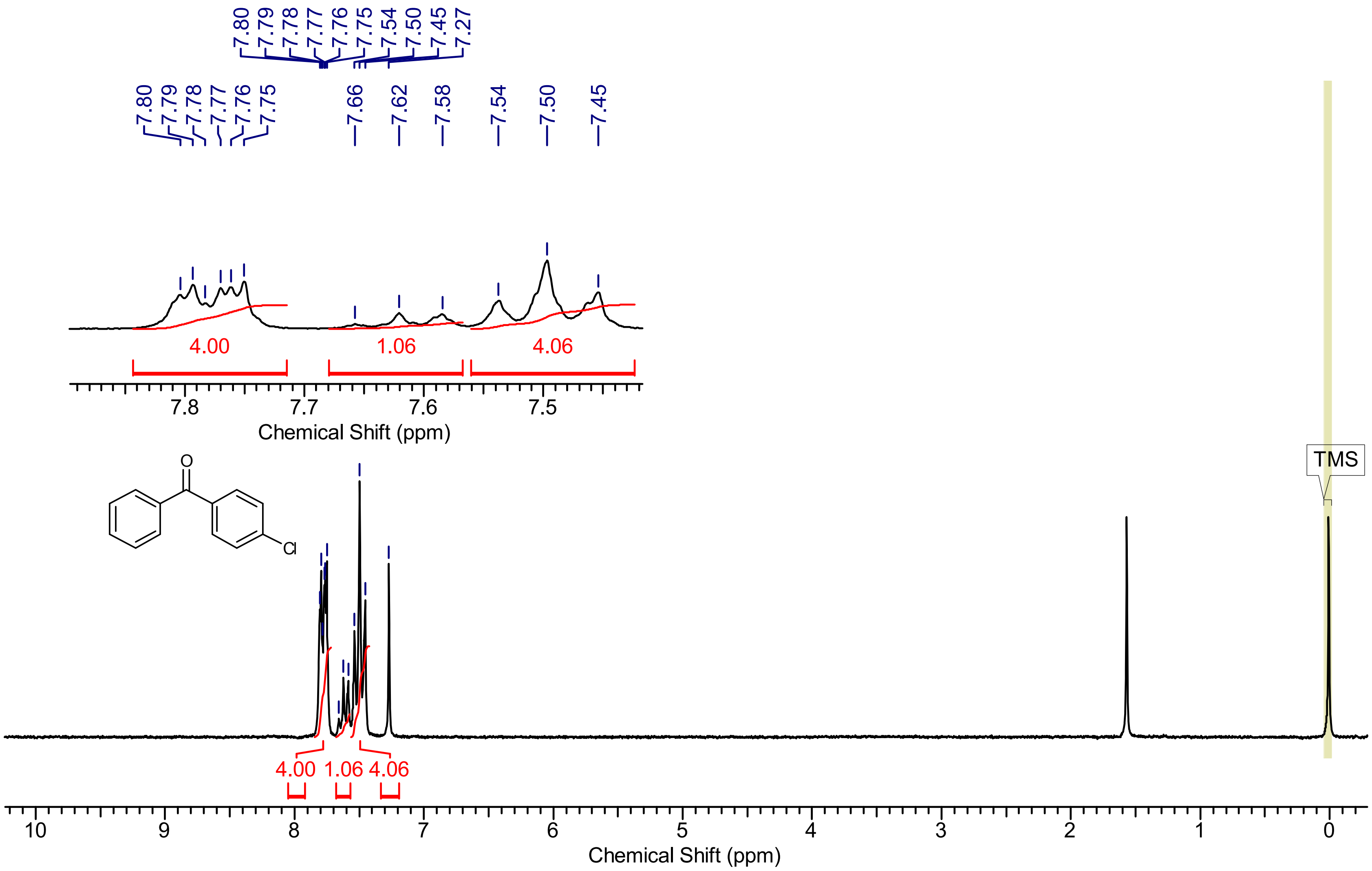

Supplementary Figure 80. ^{1}H NMR of 11c

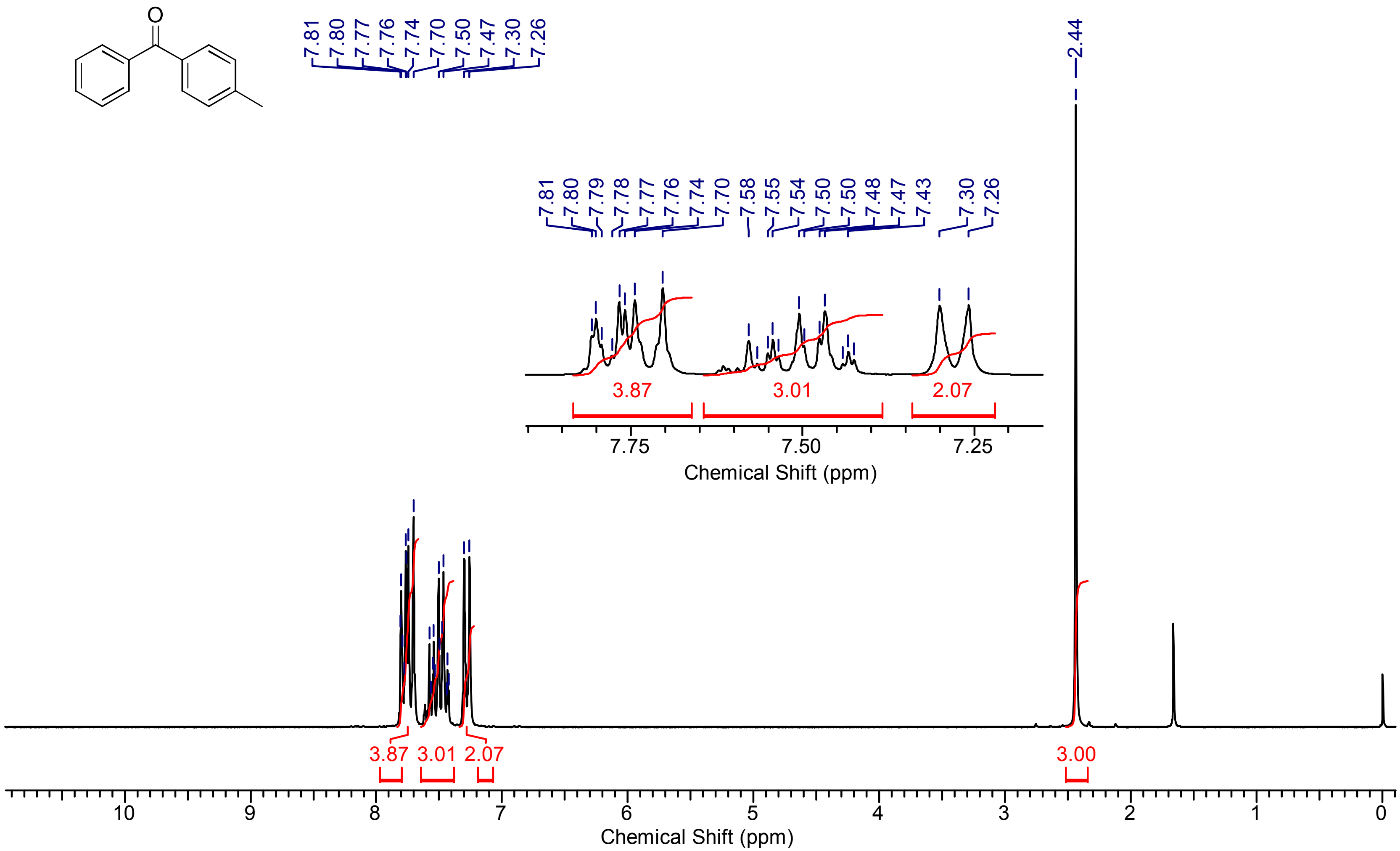

Supplementary Figure 81. ^{1}H NMR of 11d

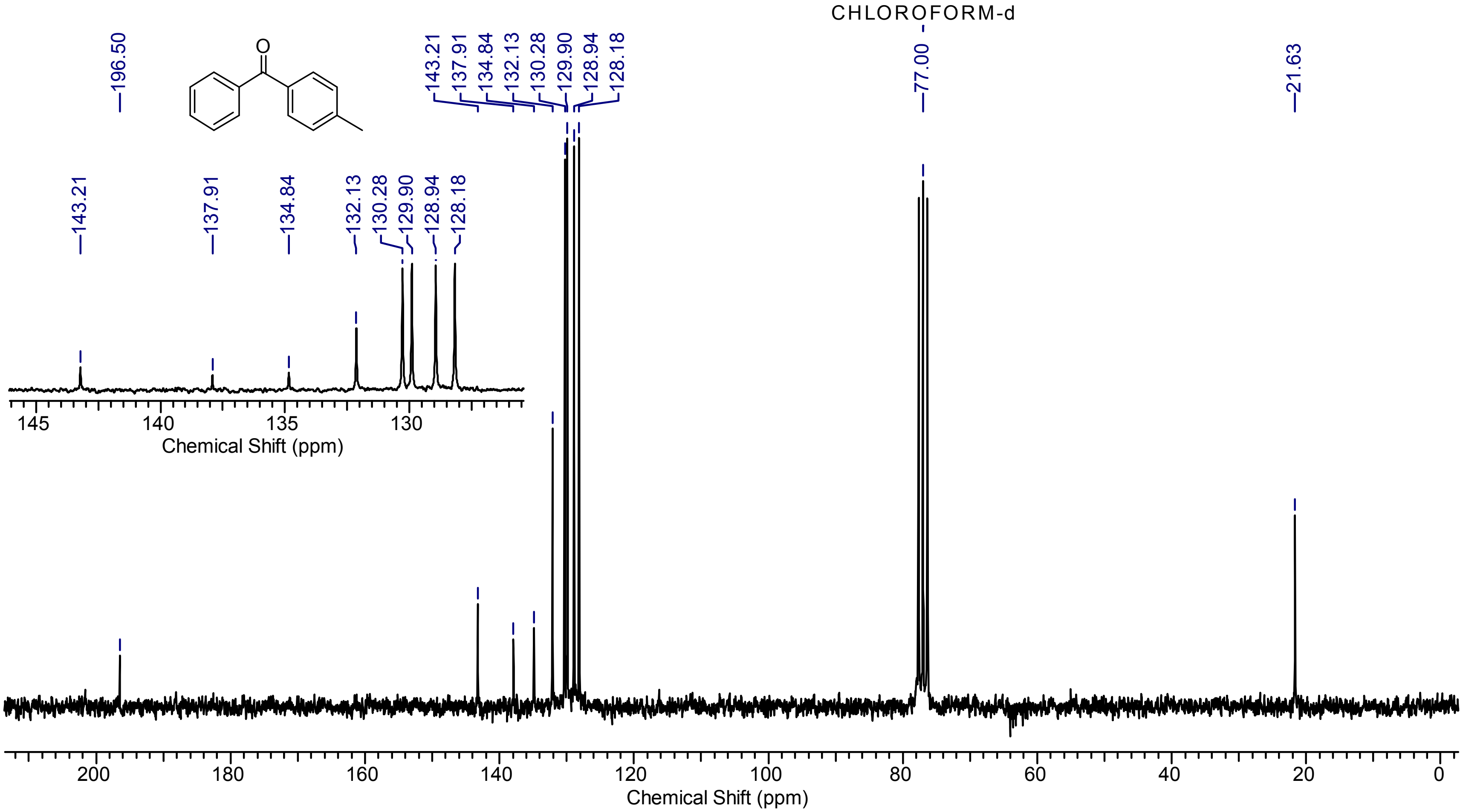

Supplementary Figure 82. ^{13}**C NMR of 11d**

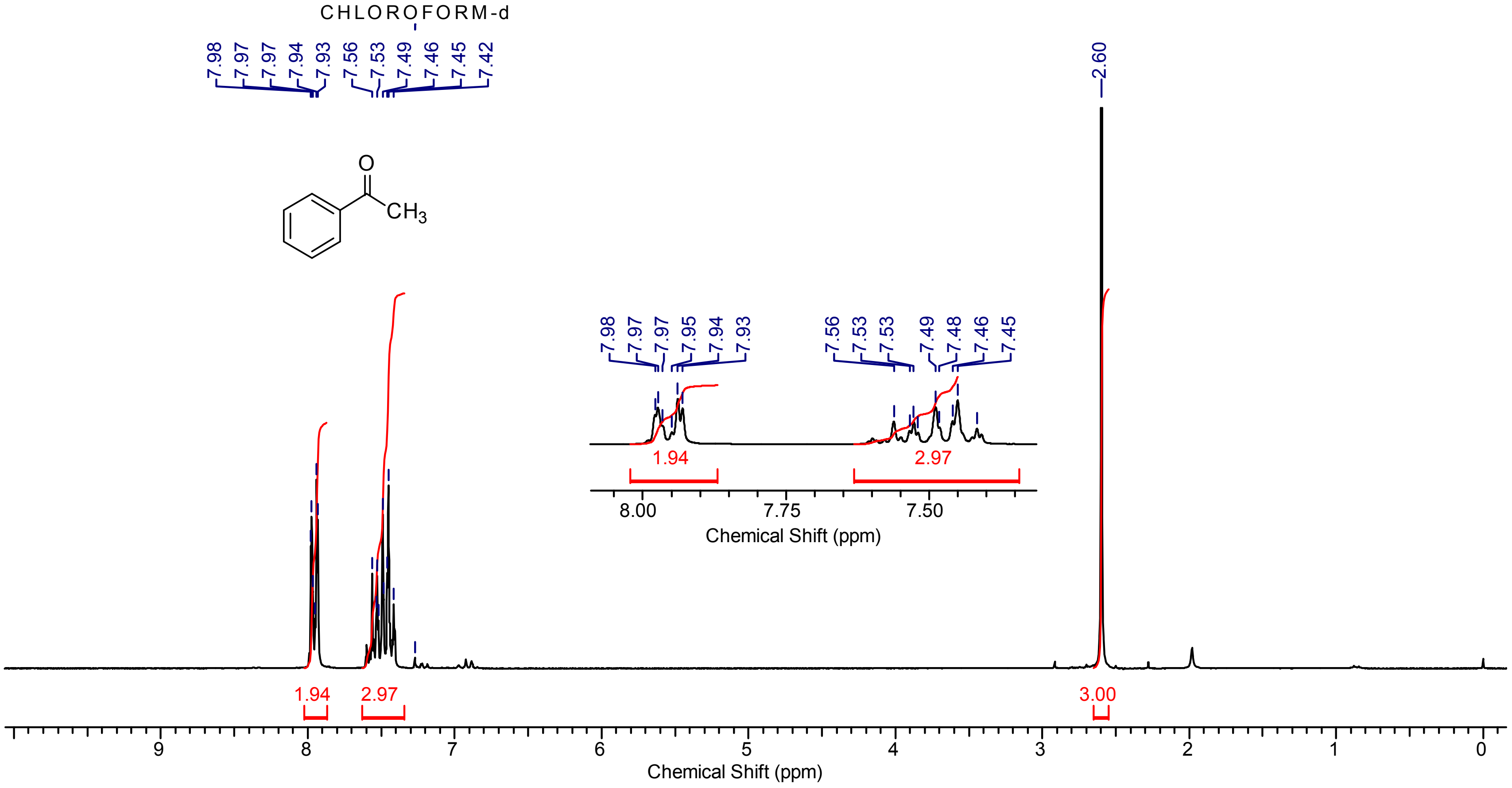

Supplementary Figure 83. ^{1}H NMR of 11e

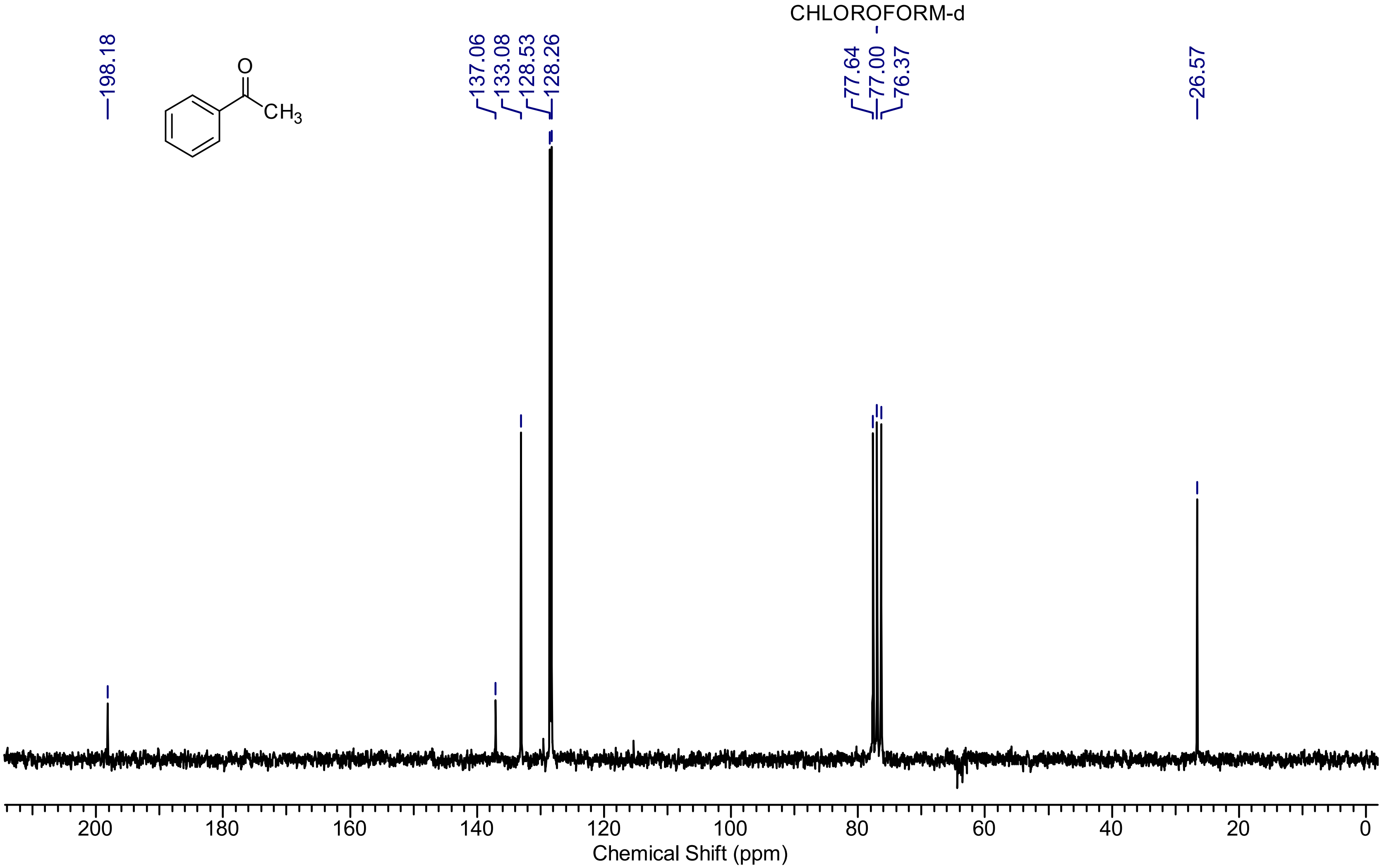

Supplementary Figure 84. ^{13}C NMR of 11e

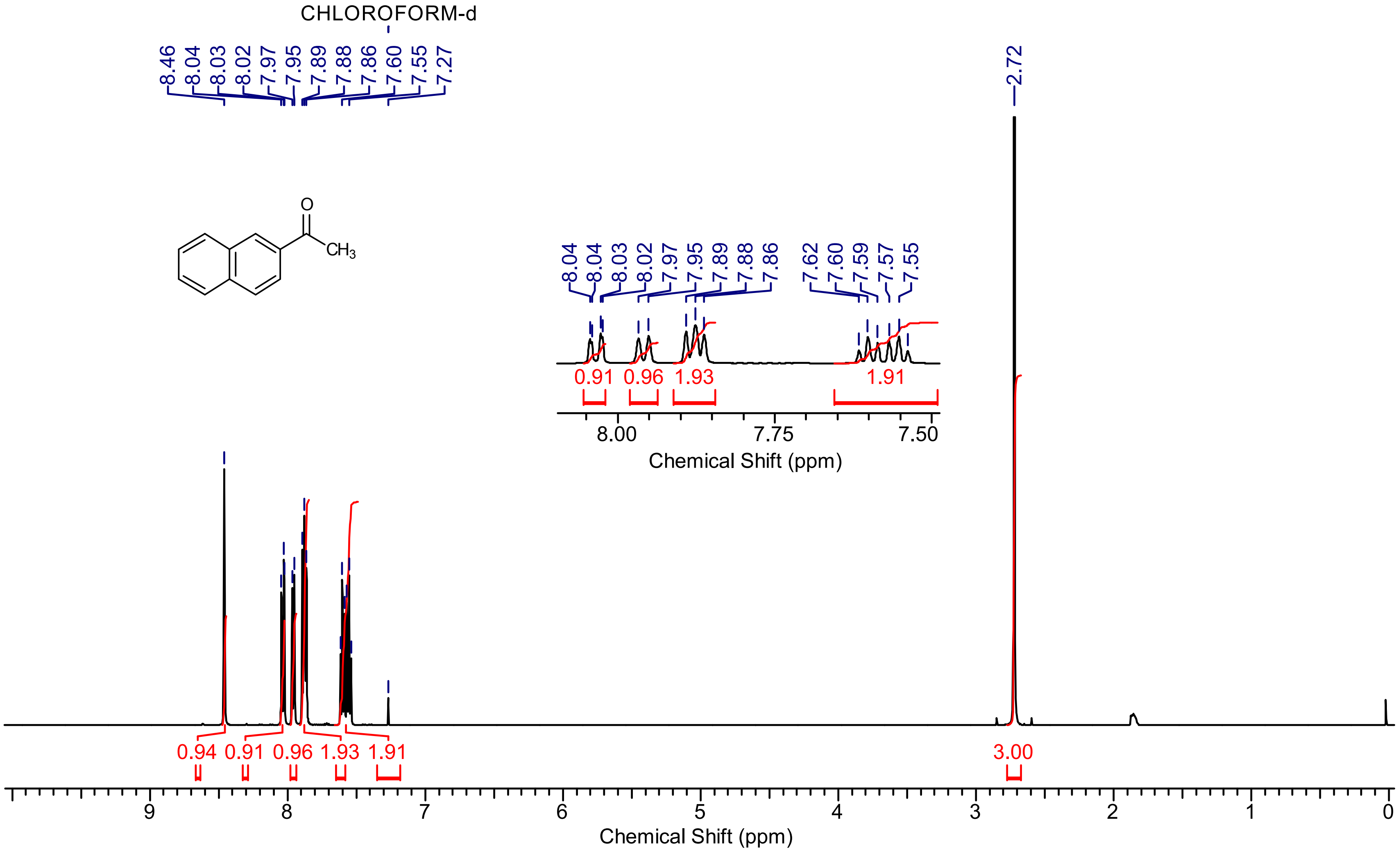

Supplementary Figure 85. ^{1}H NMR of 11f

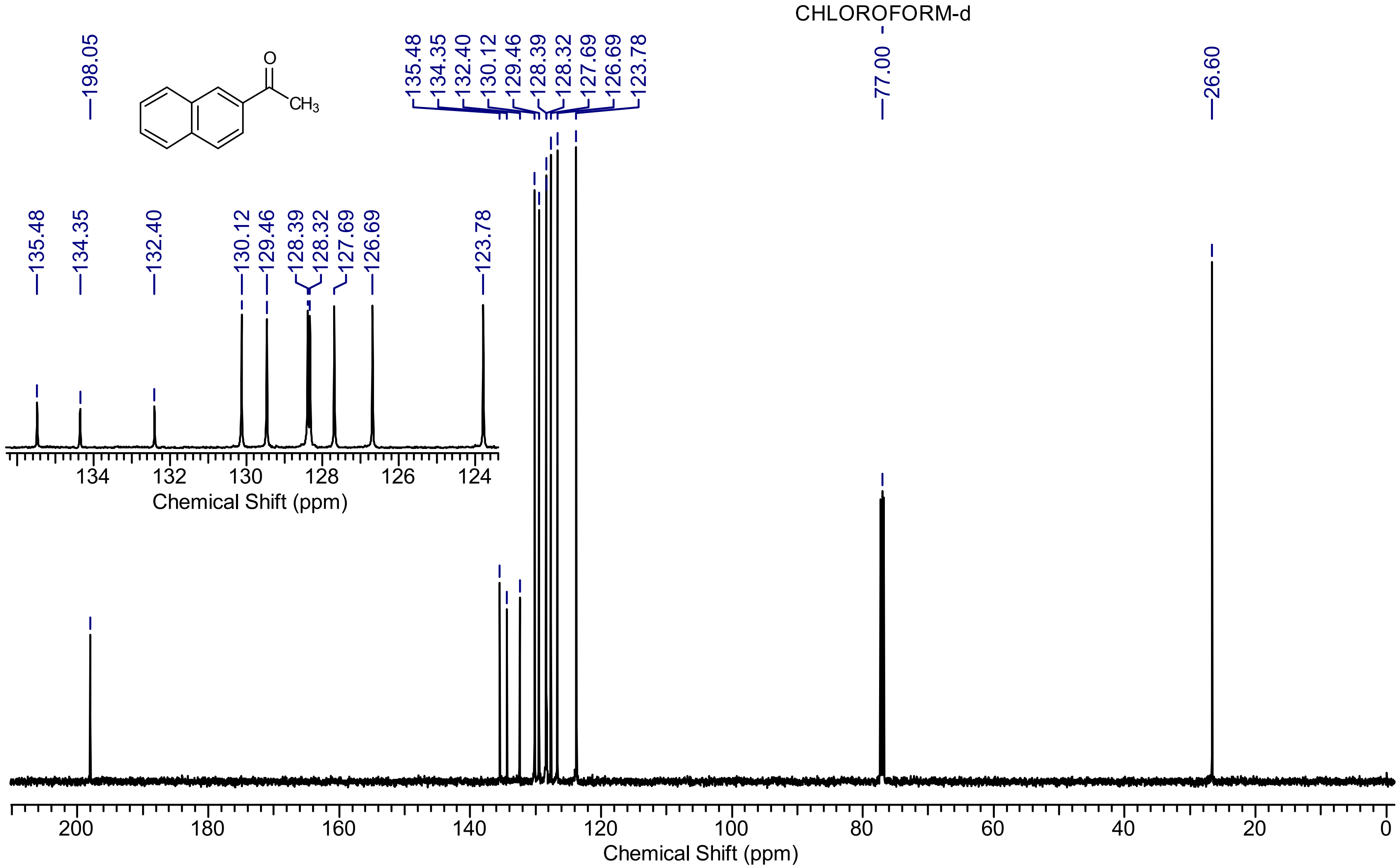

Supplementary Figure 86. ^{13}**C NMR of 11f**

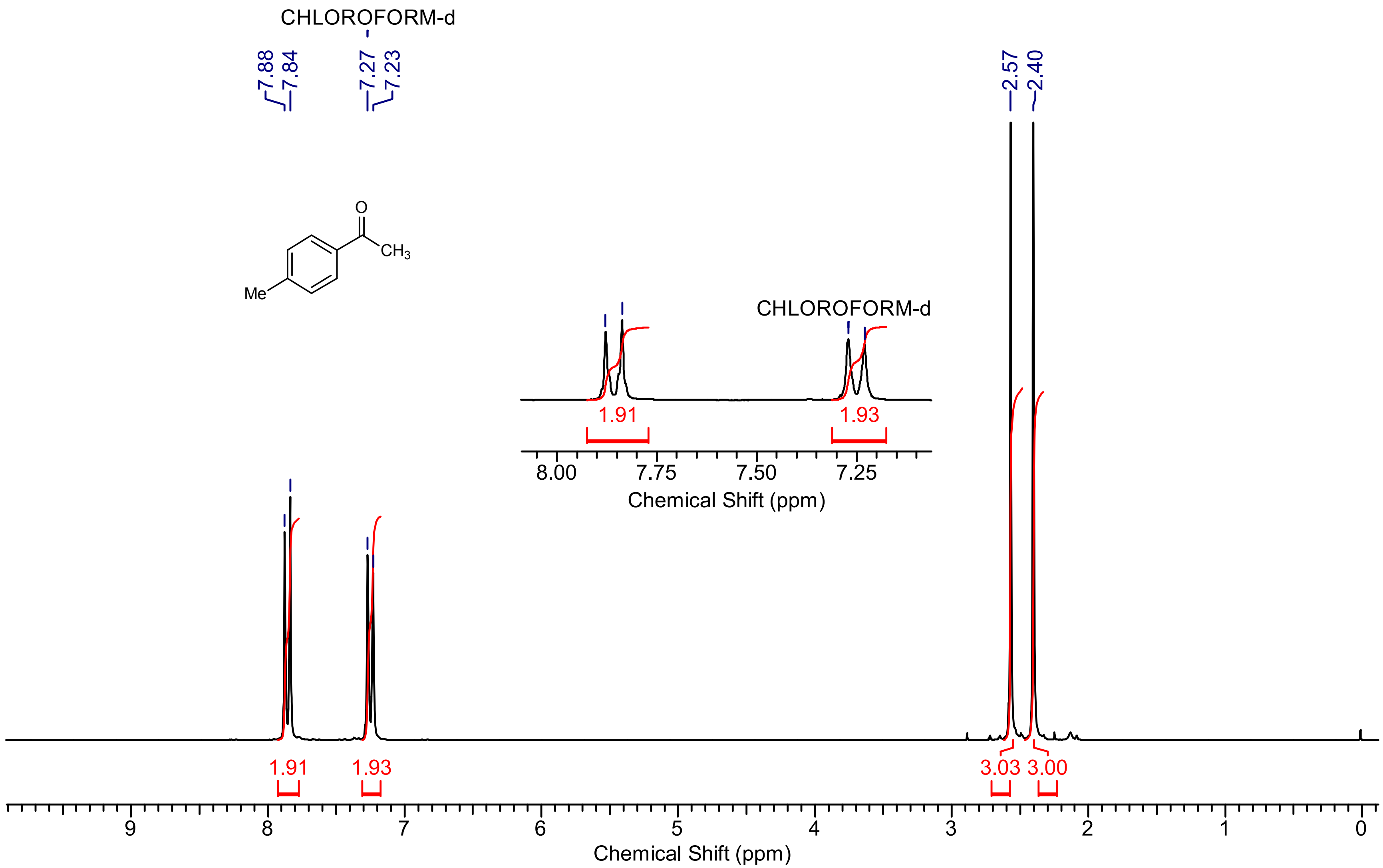

Supplementary Figure 87. ^{1}H NMR of 11g

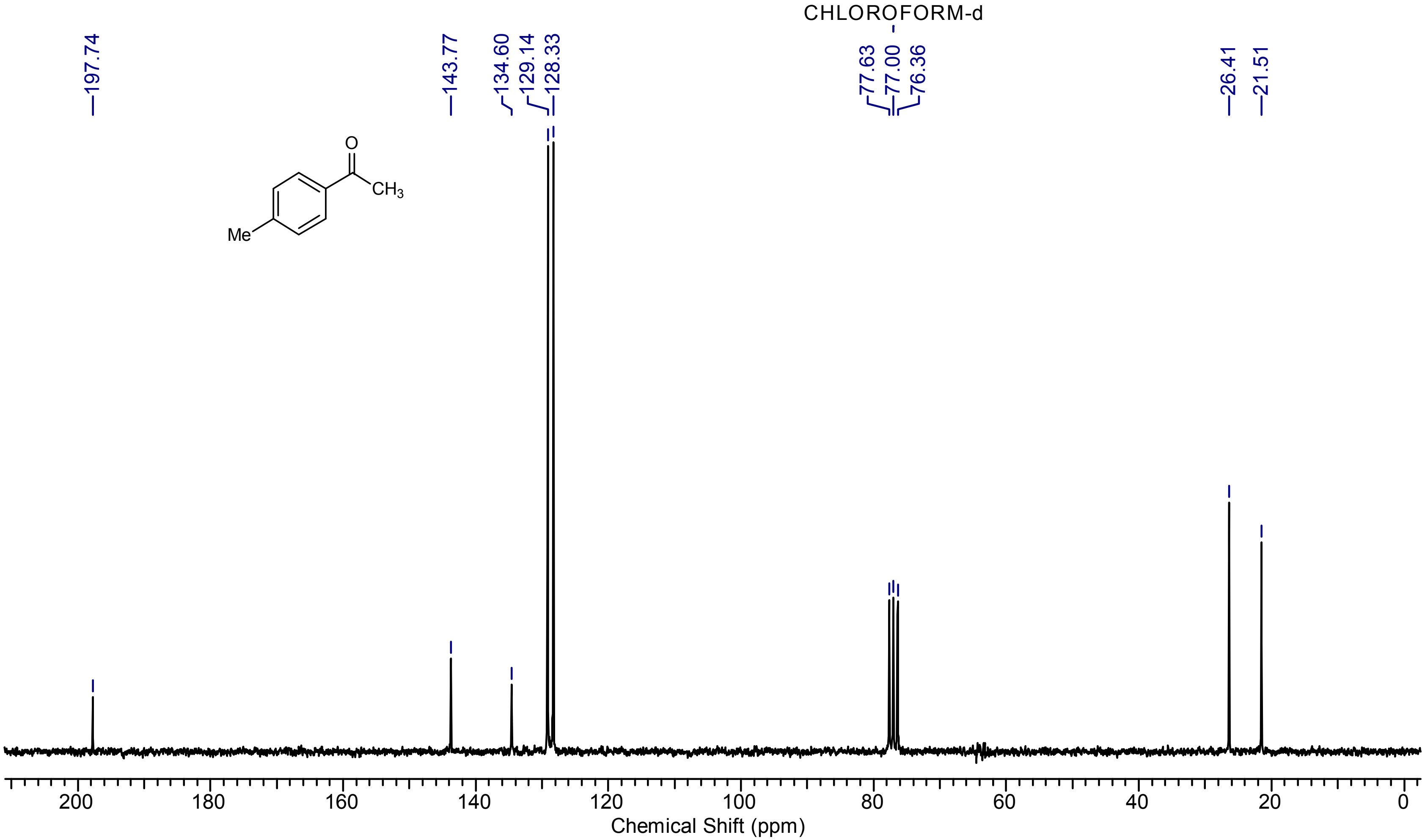

Supplementary Figure 88. ^{13}C NMR of 11g

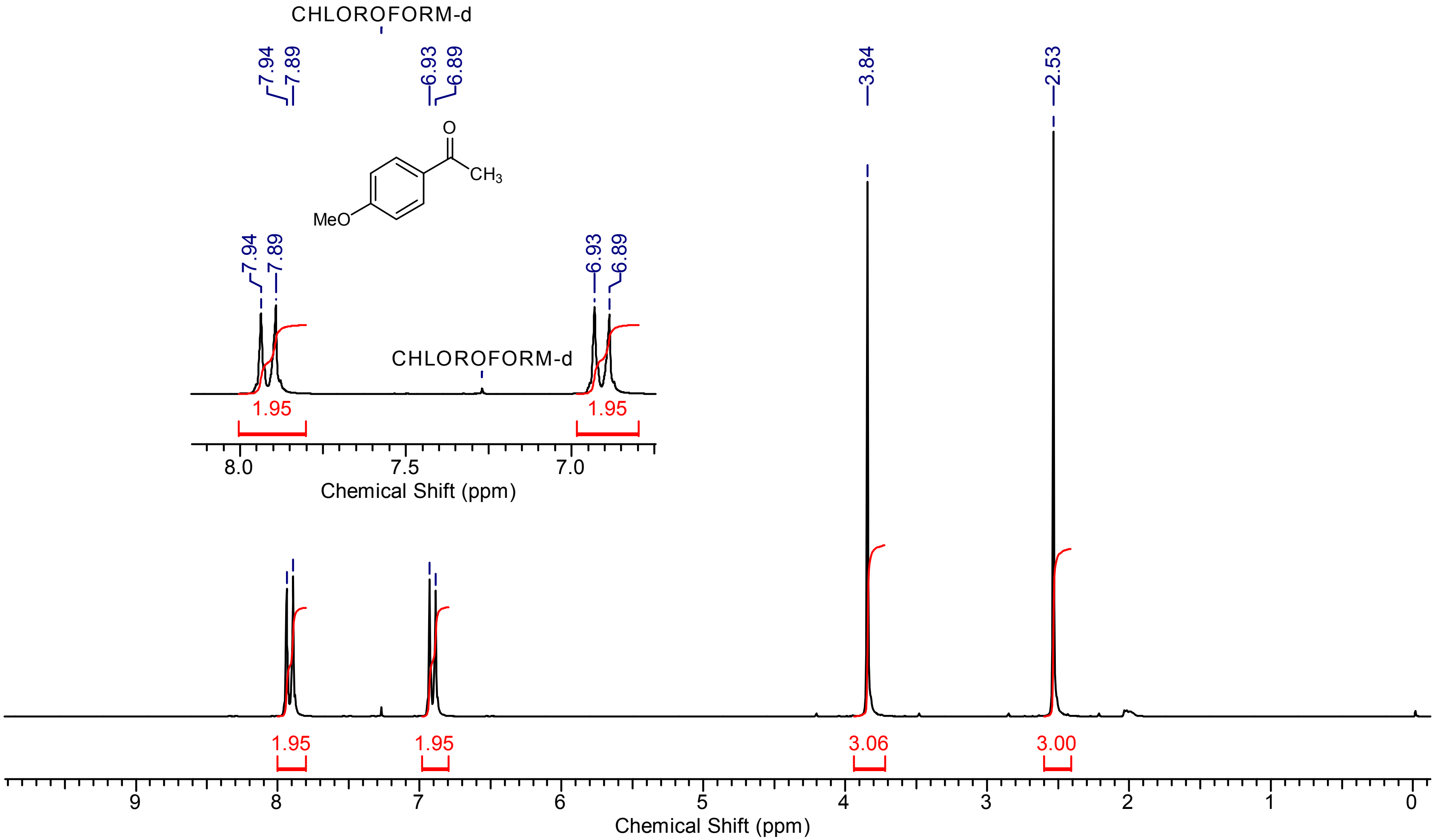

Supplementary Figure 89. ^{1}H NMR of 11h

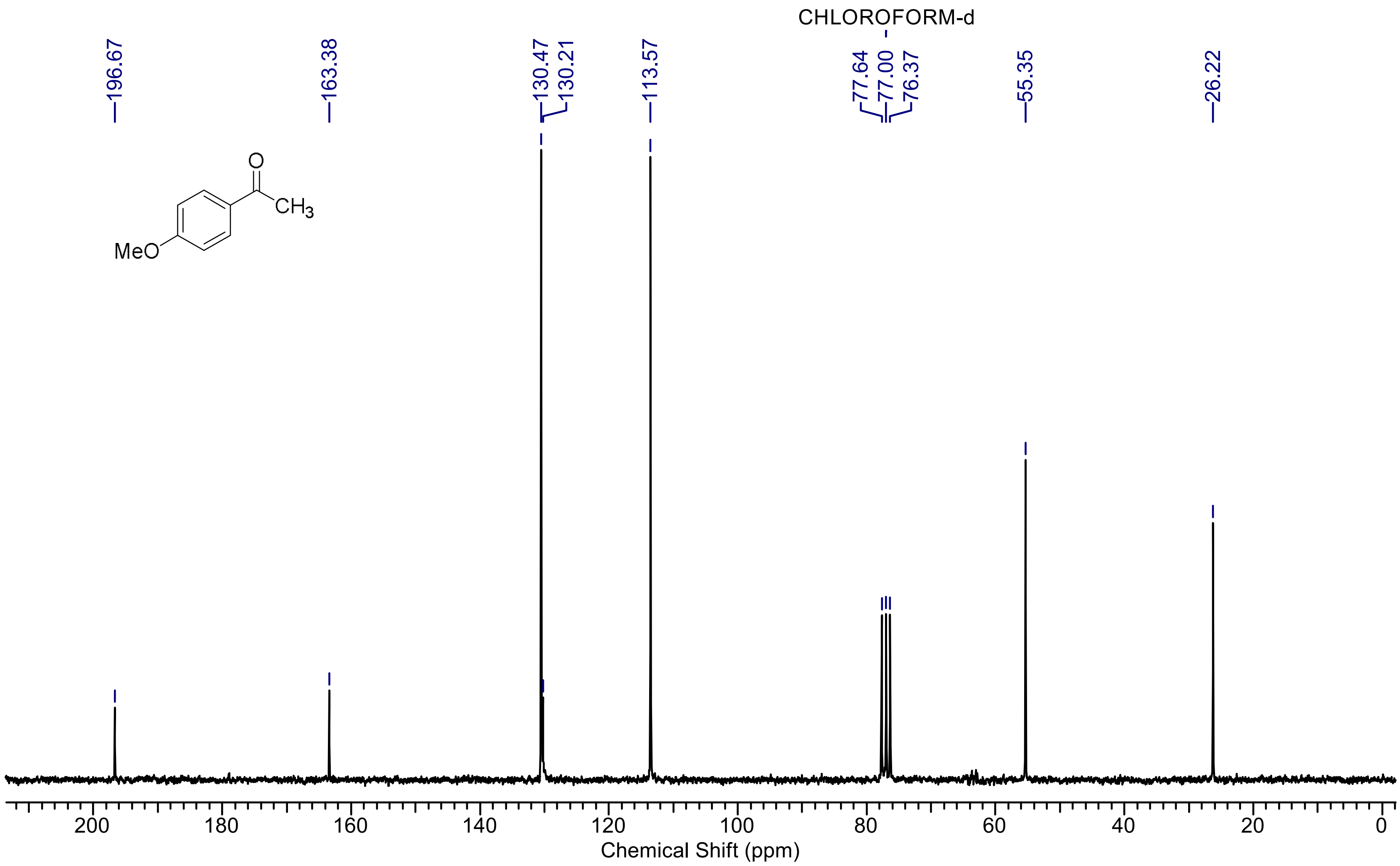

Supplementary Figure 90. ^{13}C NMR of 11h

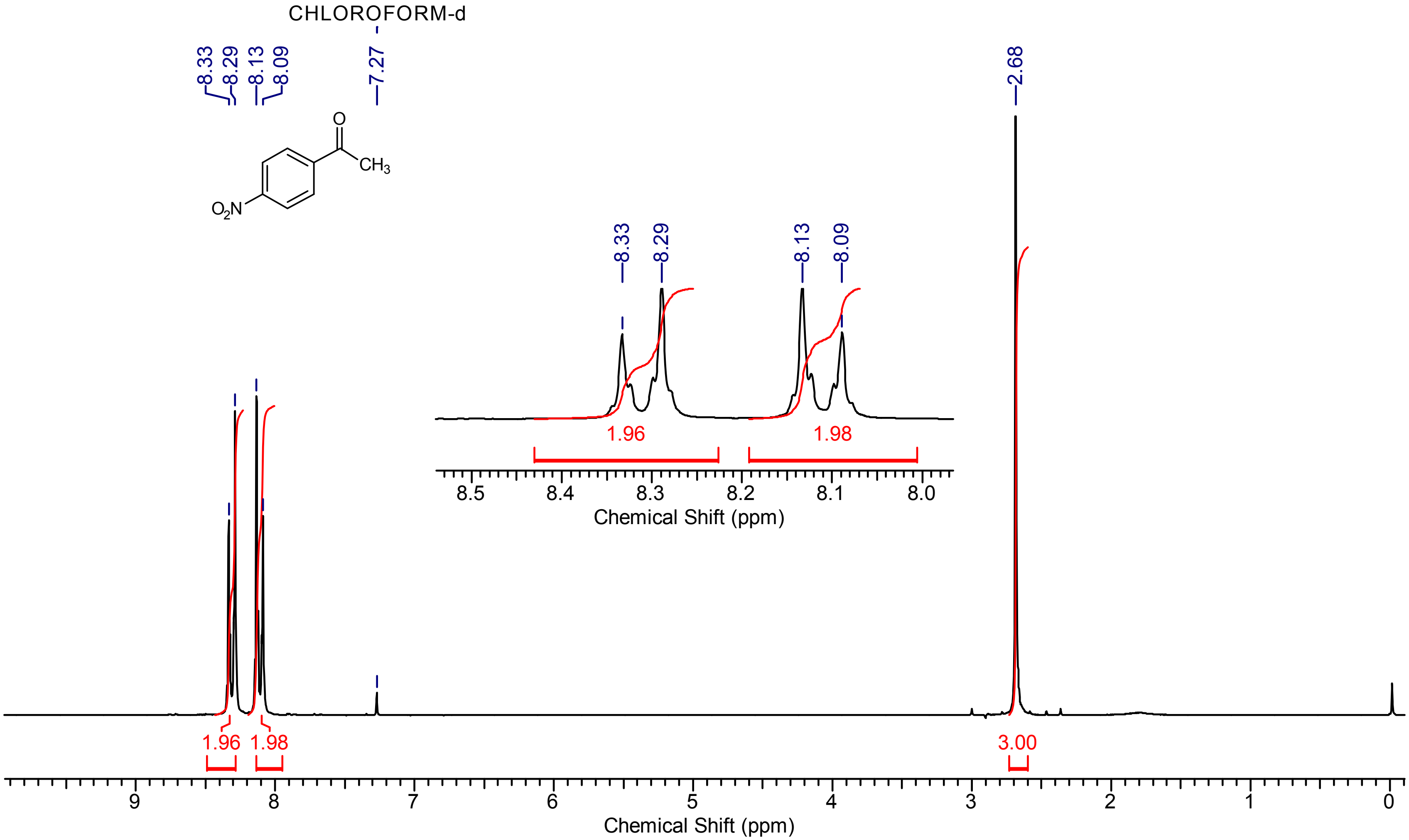

Supplementary Figure 91. ^{1}H NMR of 11i

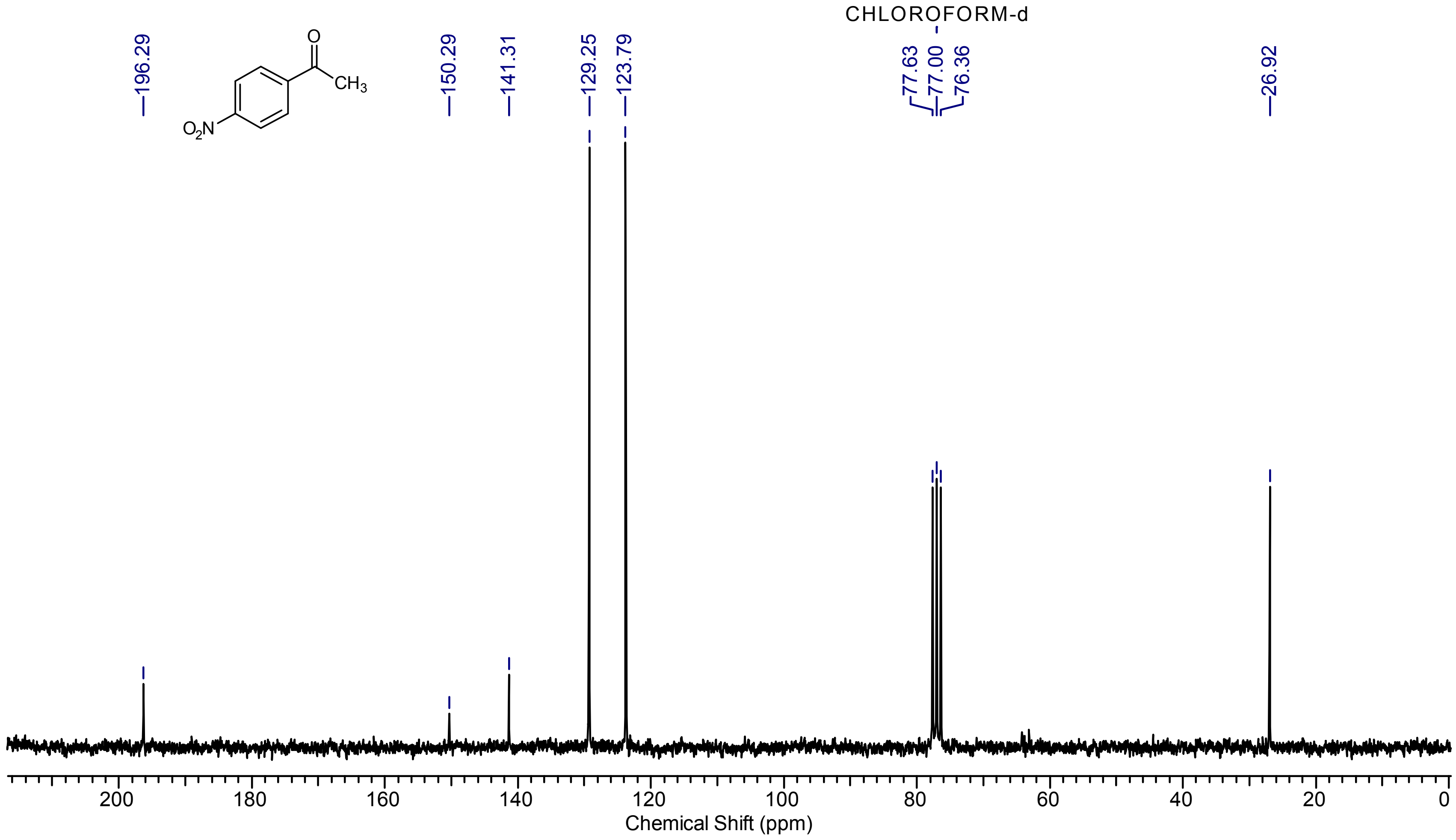

Supplementary Figure 92. ^{13}C NMR of 11i

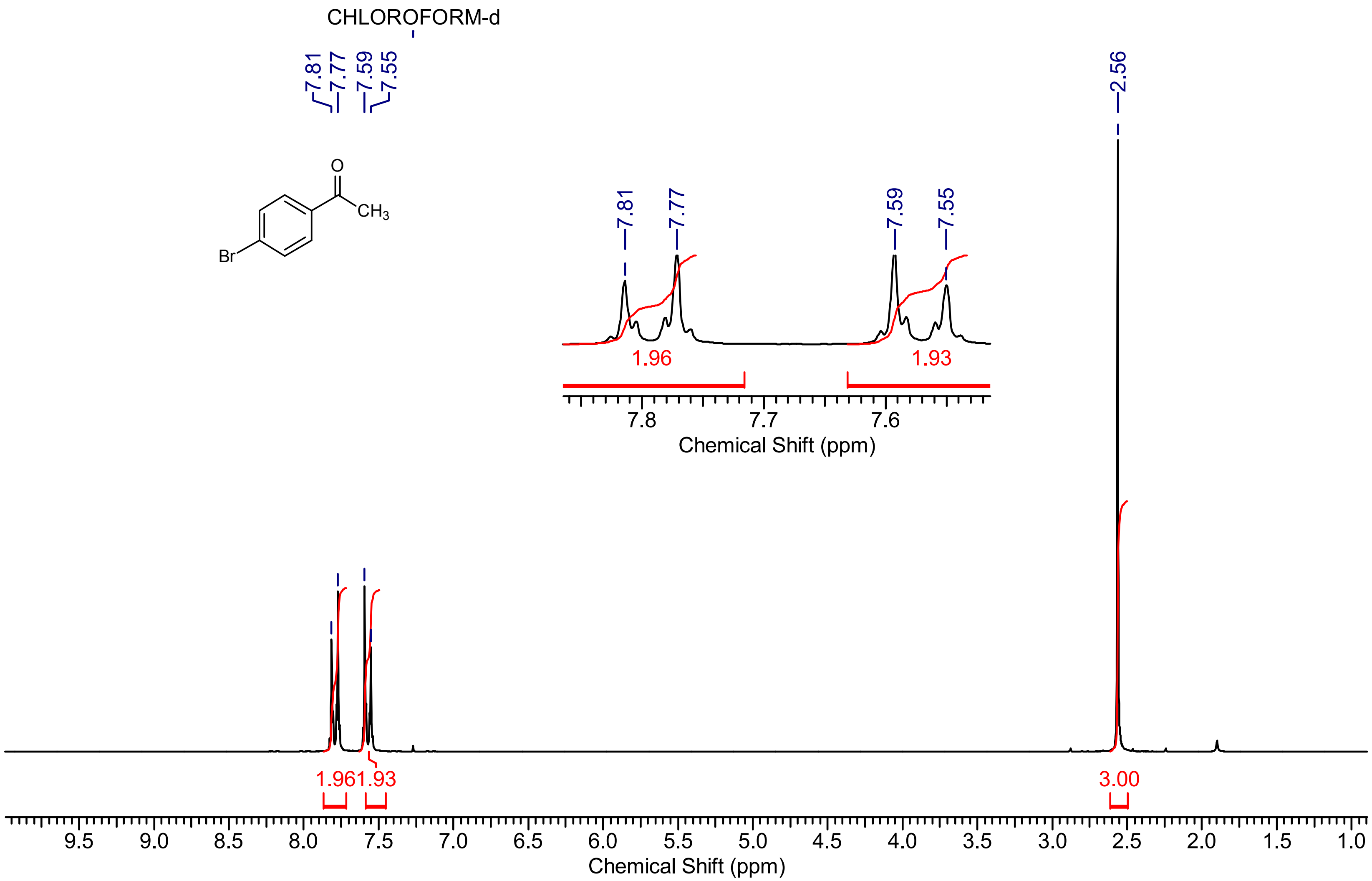

Supplementary Figure 93. ^{1}H NMR of 11j

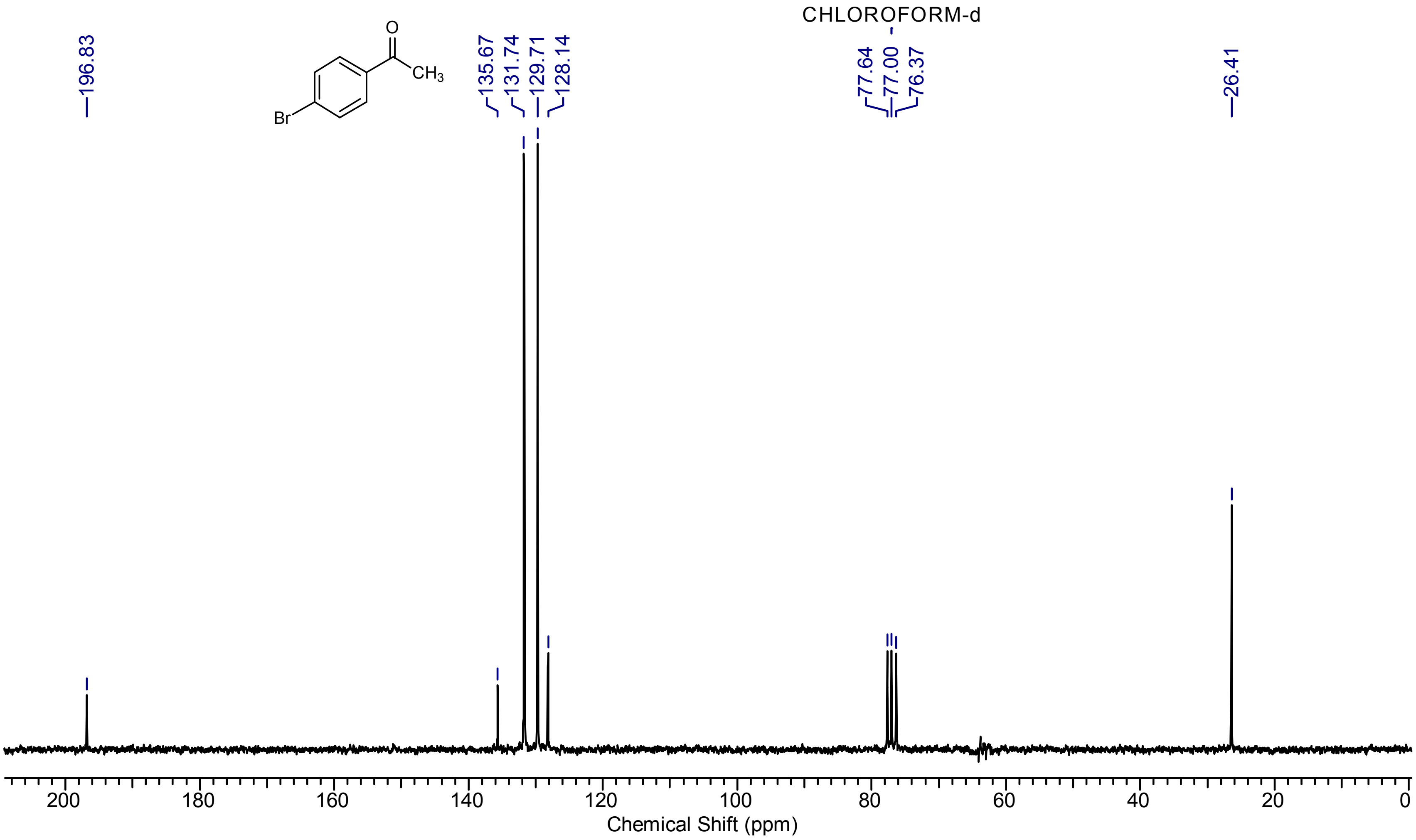

Supplementary Figure 94. ^{13}C NMR of 11j

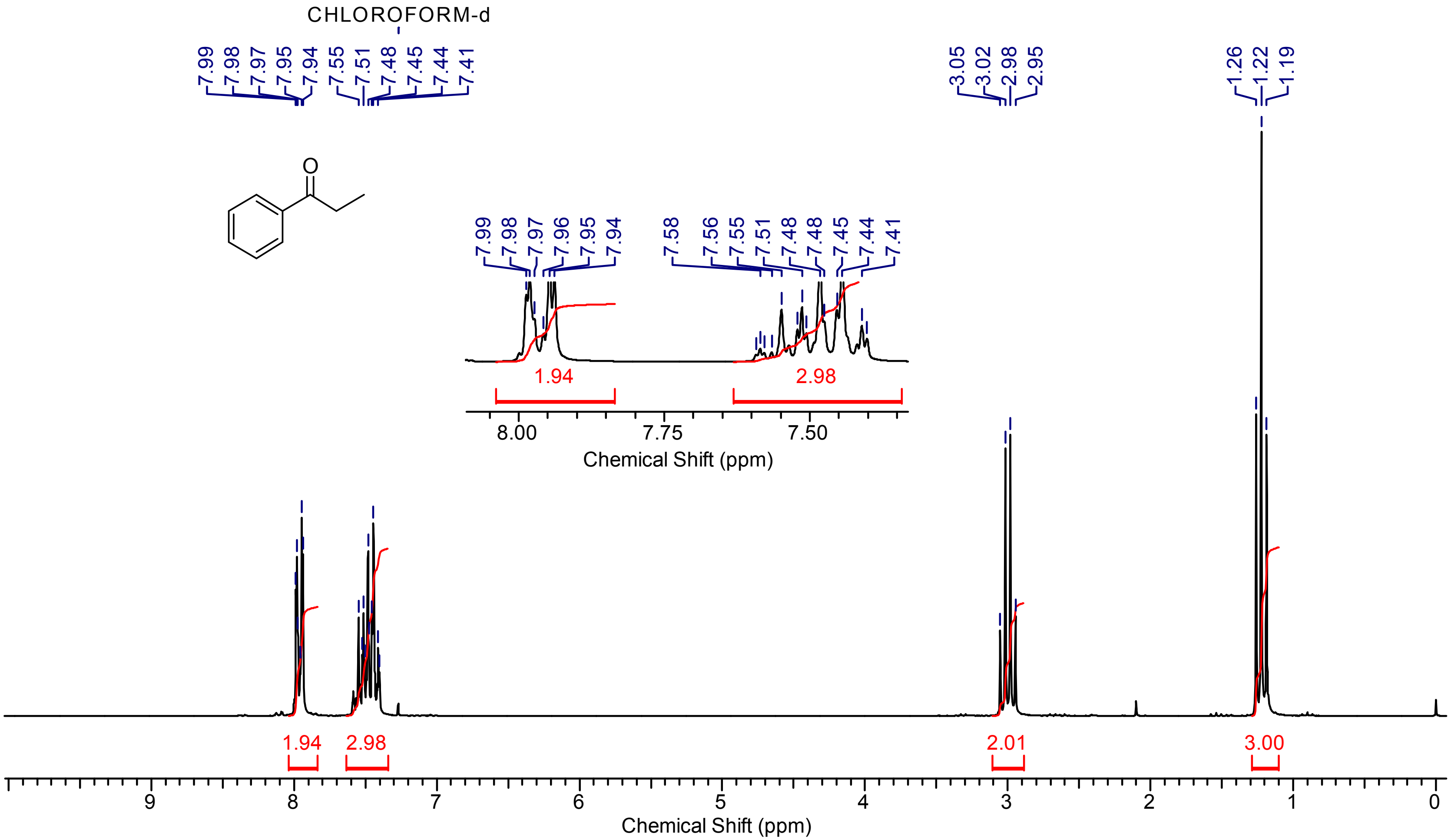

Supplementary Figure 95. ^{1}**H NMR of 11k**

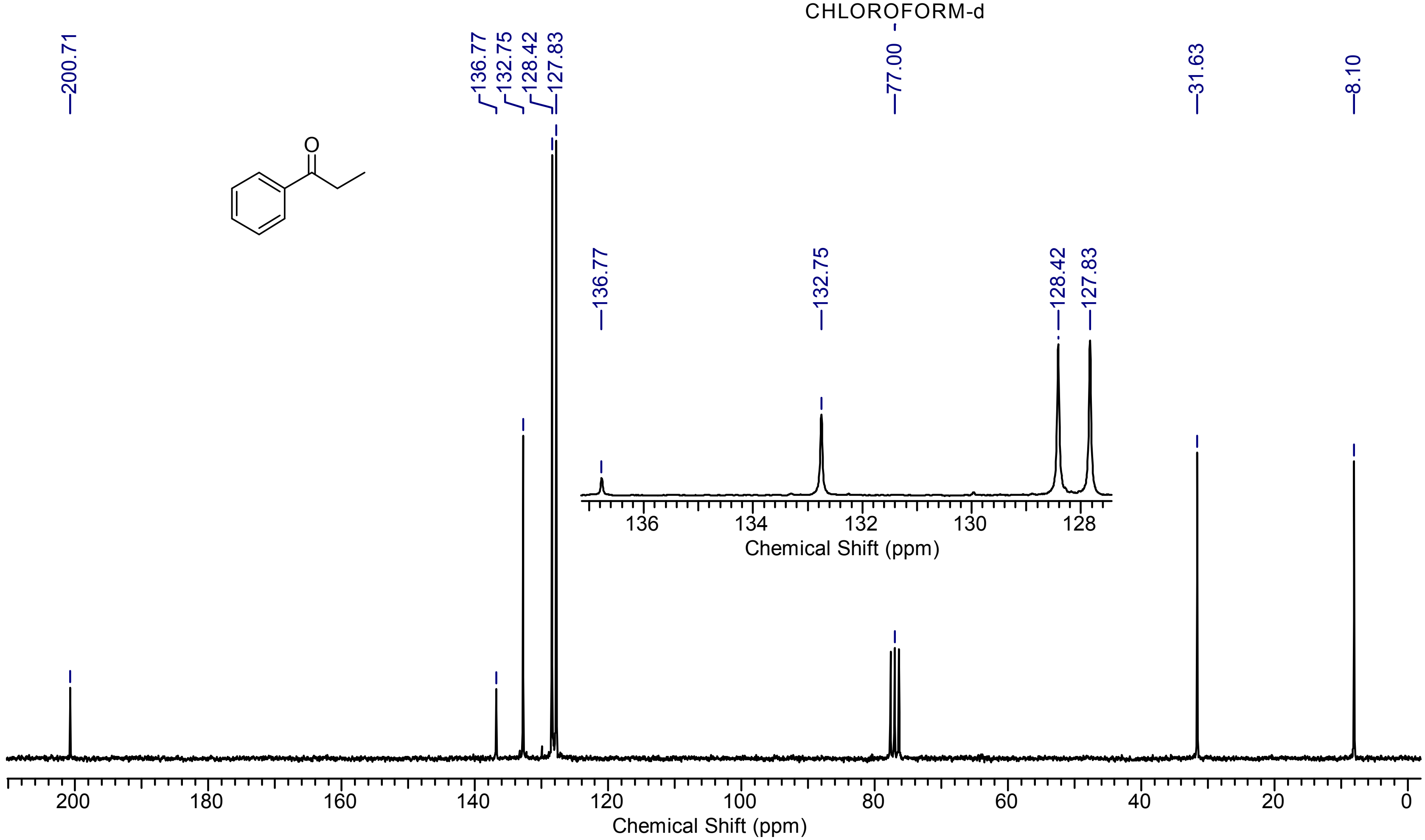

Supplementary Figure 96. ^{13}C NMR of 11k

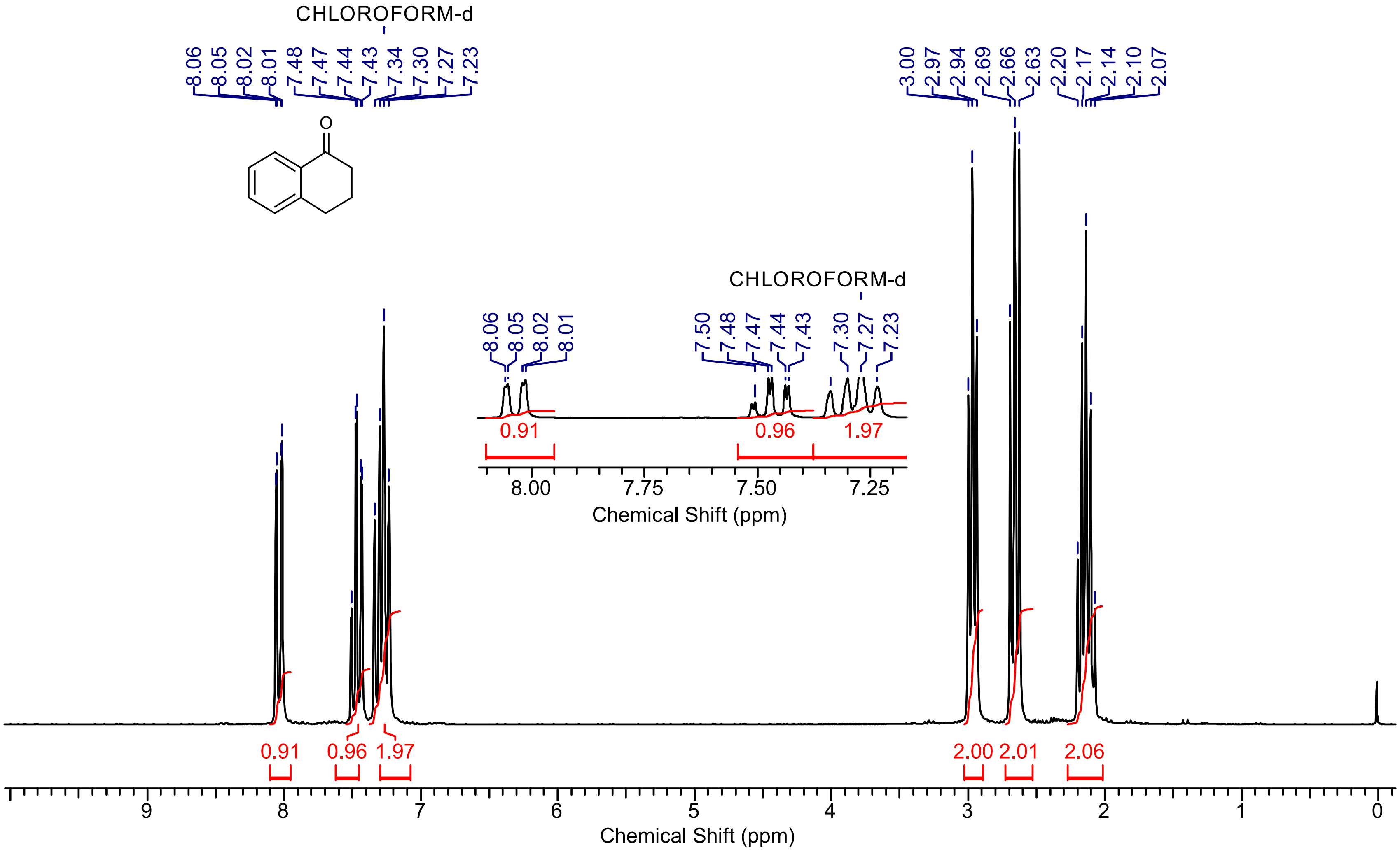

Supplementary Figure 97. ^{1}H NMR of 11l

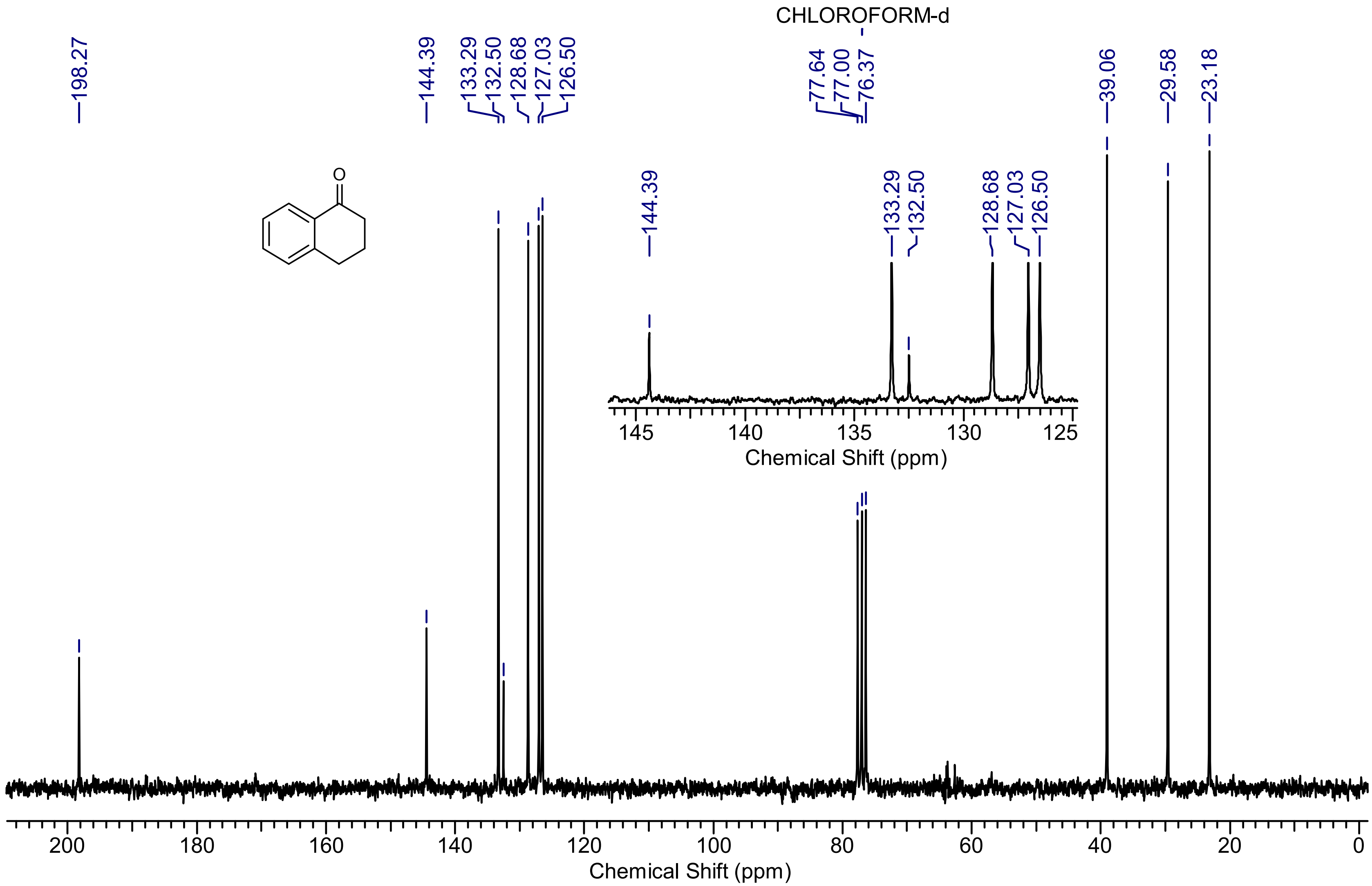

Supplementary Figure 98. ^{13}C NMR of 11l

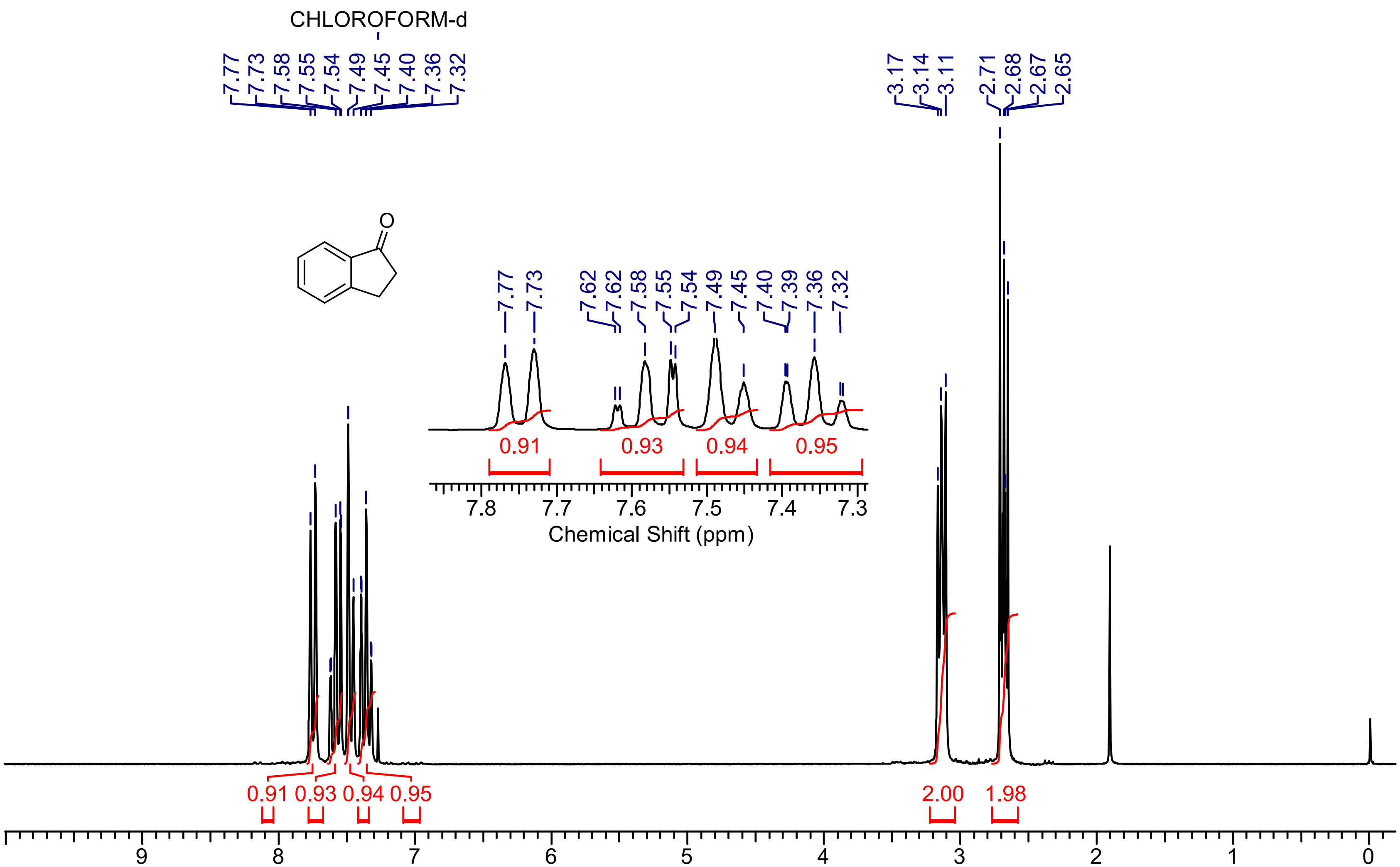

Supplementary Figure 99. ^{1}H NMR of 11n

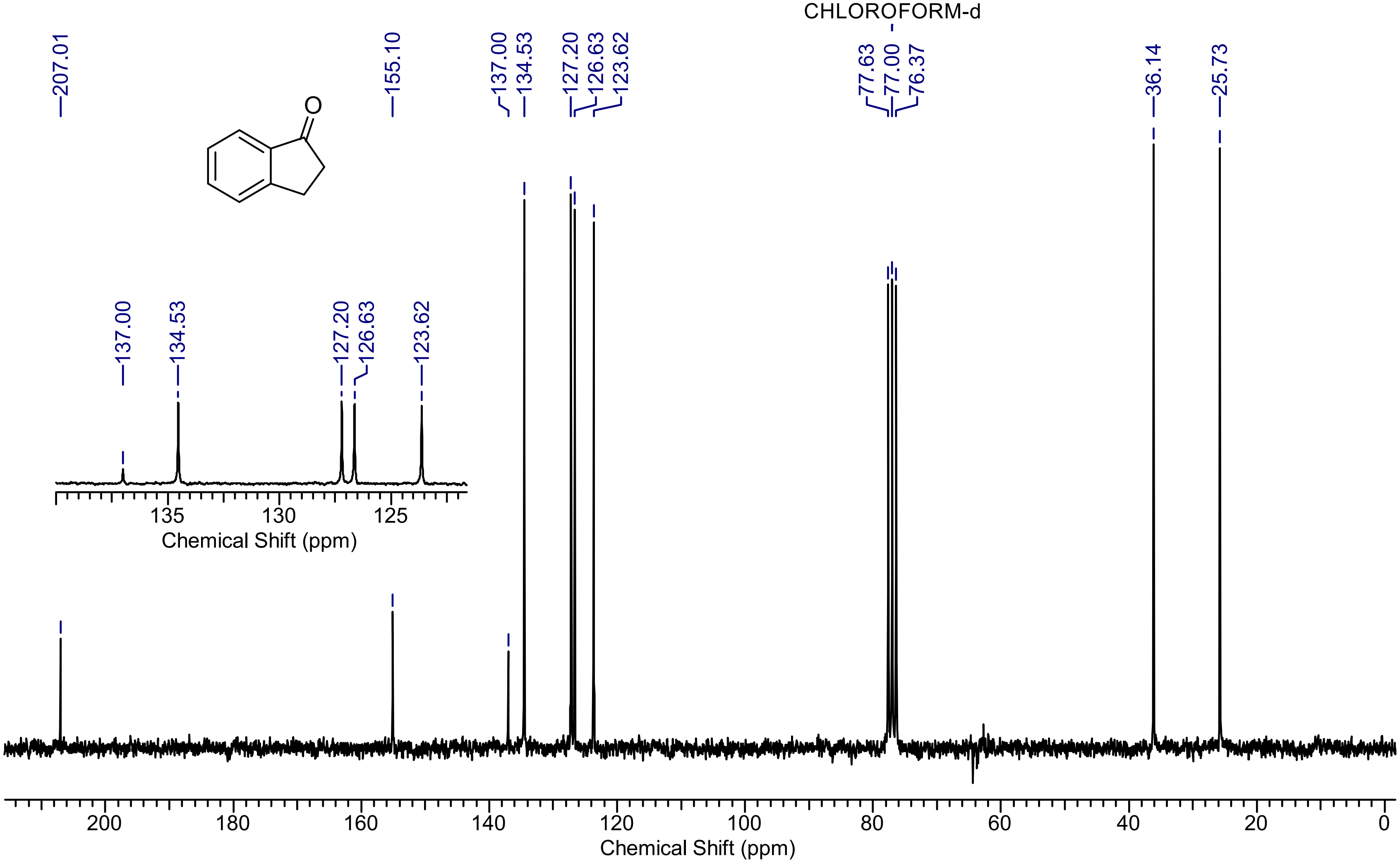

Supplementary Figure 100. ^{13}C NMR of 11n

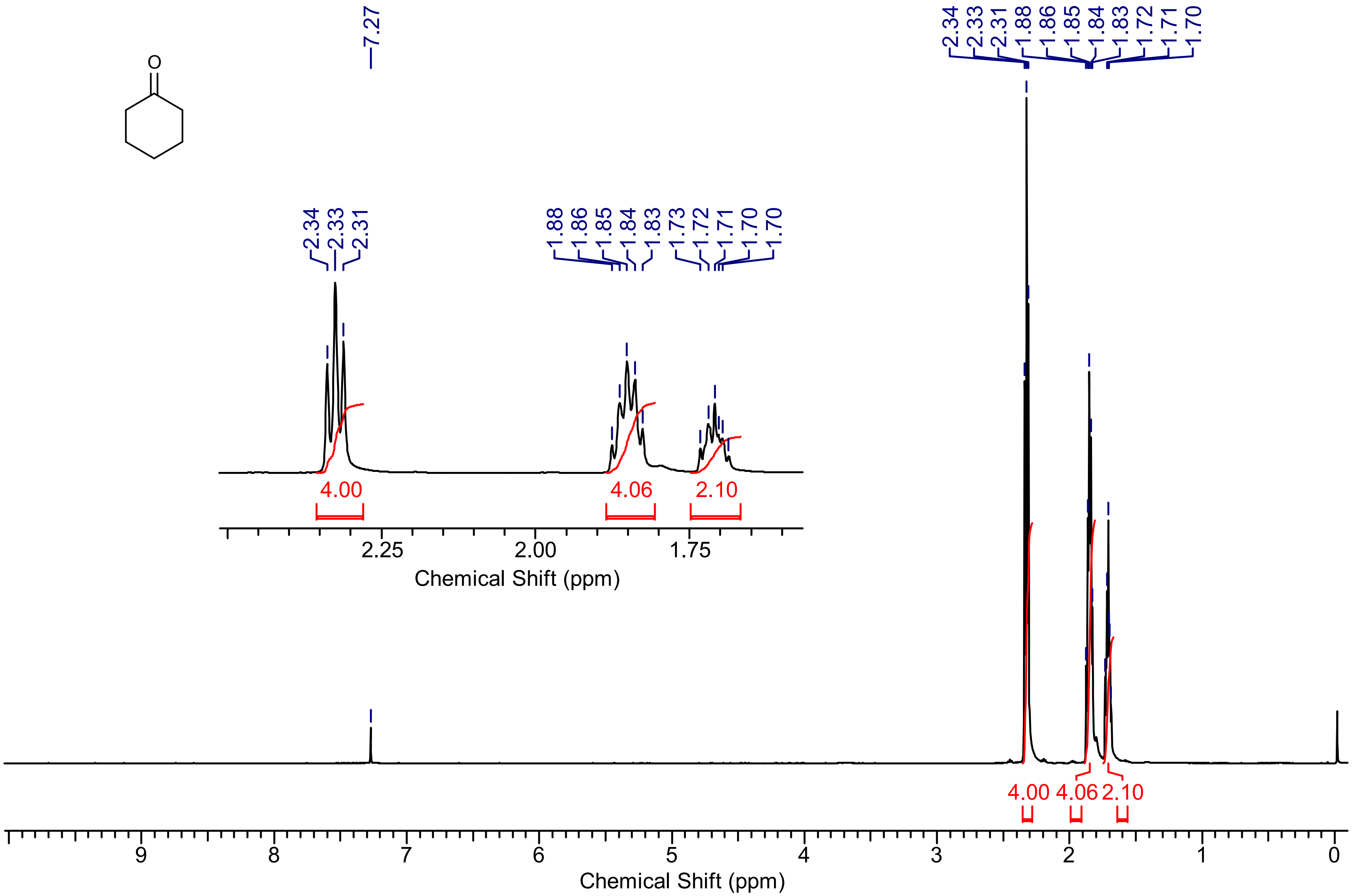

Supplementary Figure 101. ^{1}H NMR of 11o

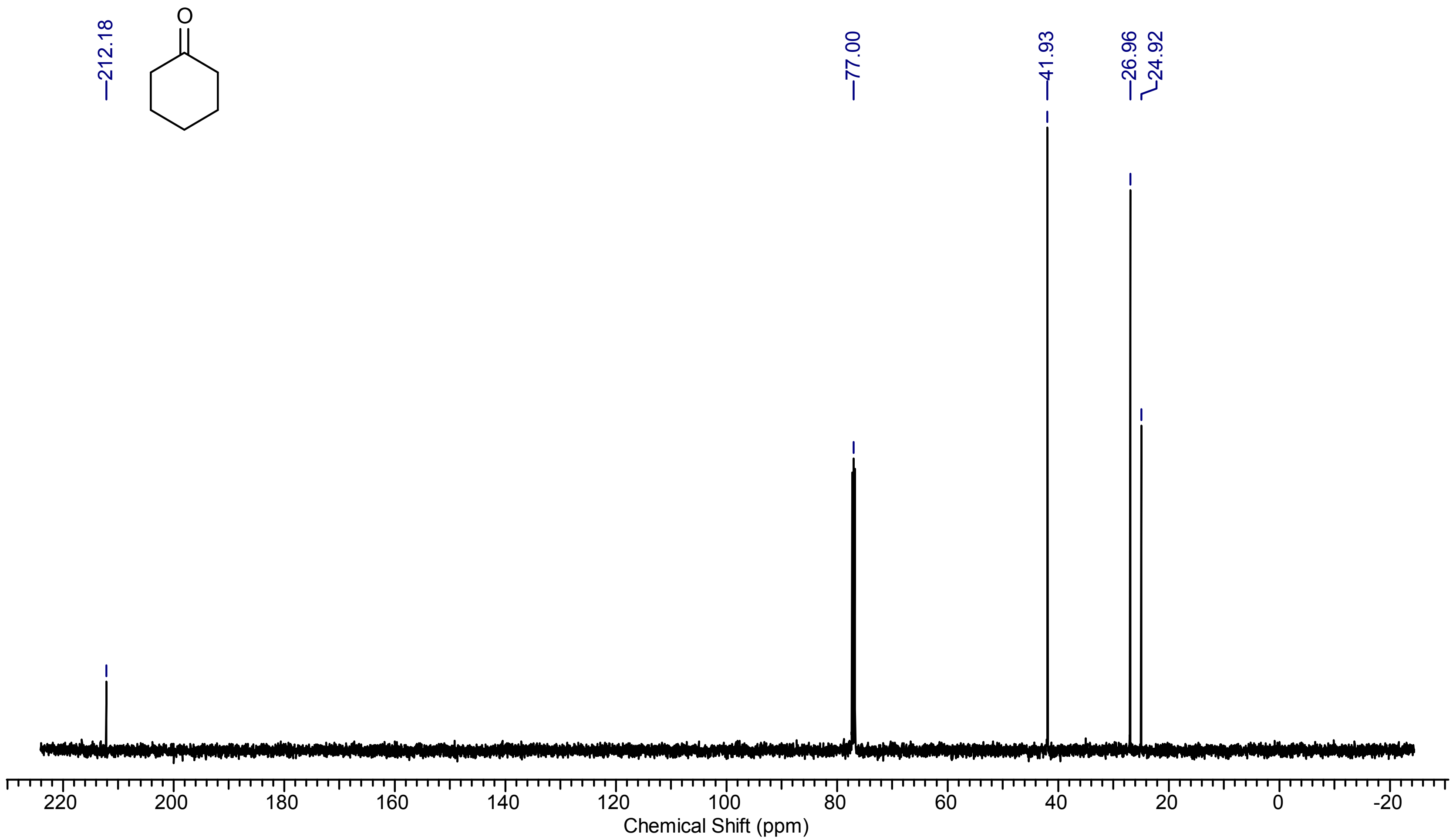

Supplementary Figure 102. ^{13}C NMR of 11o

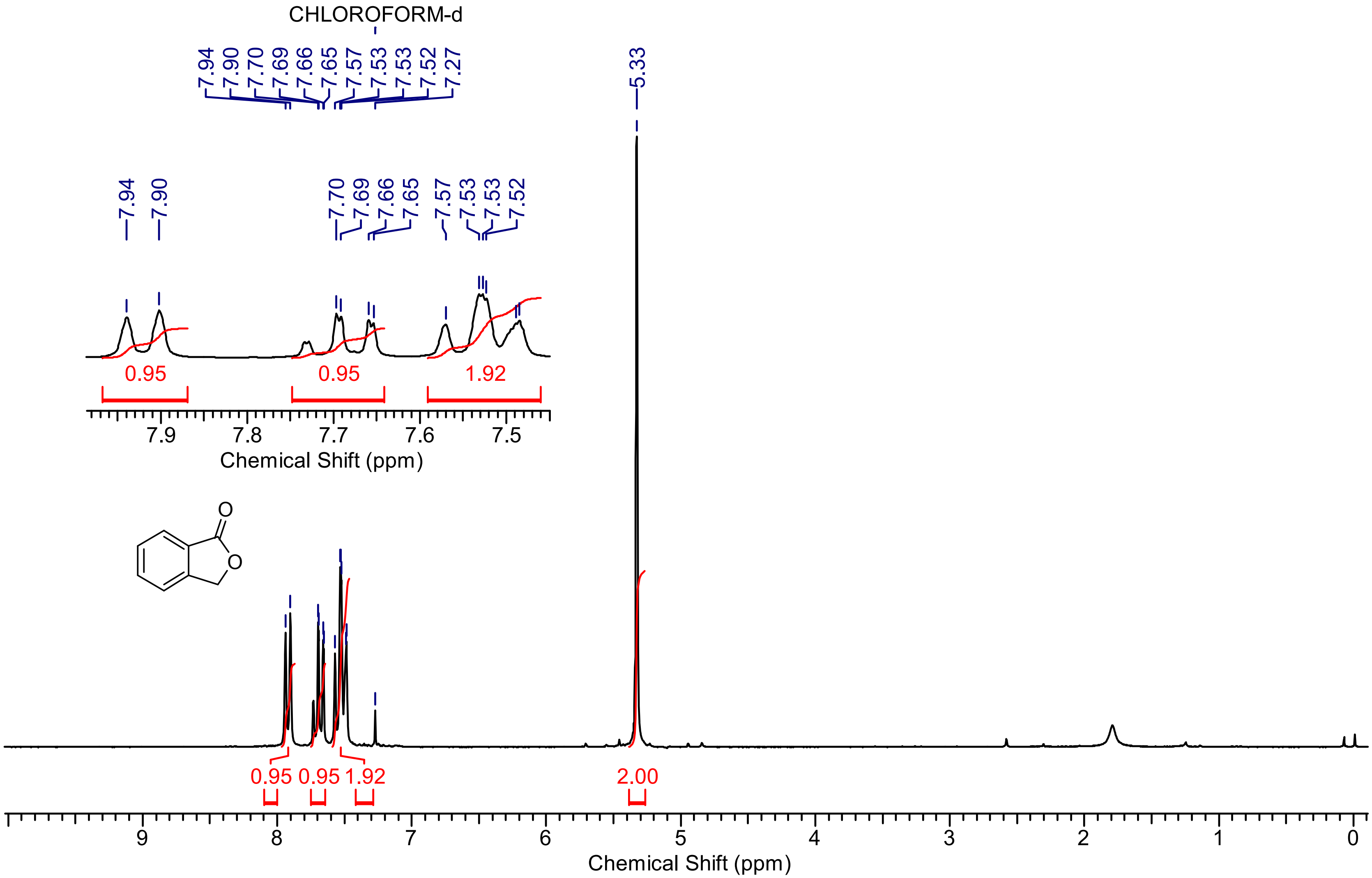

Supplementary Figure 103. ^{1}H NMR of 13

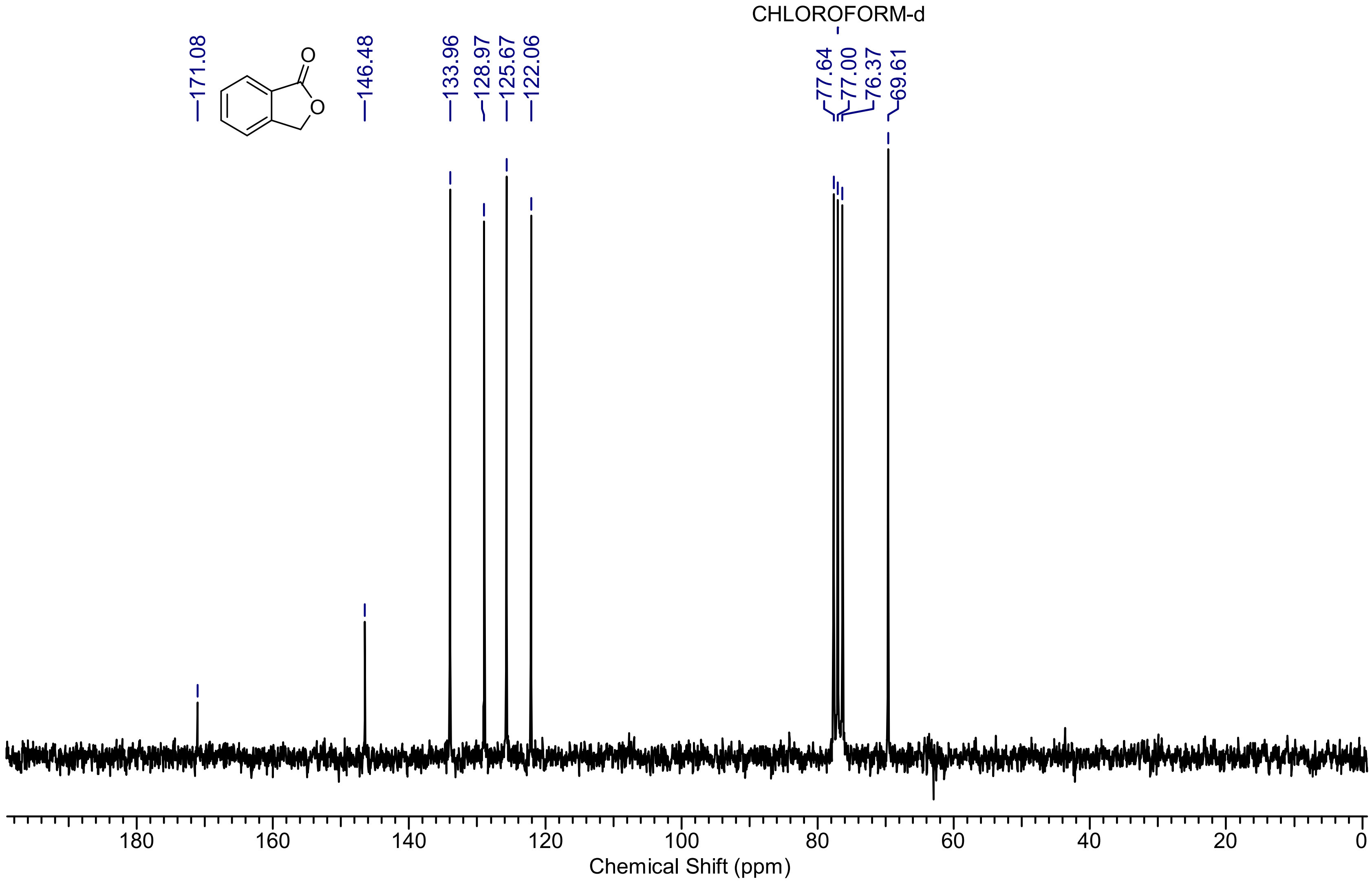

Supplementary Figure 104. ^{13}C NMR of 13

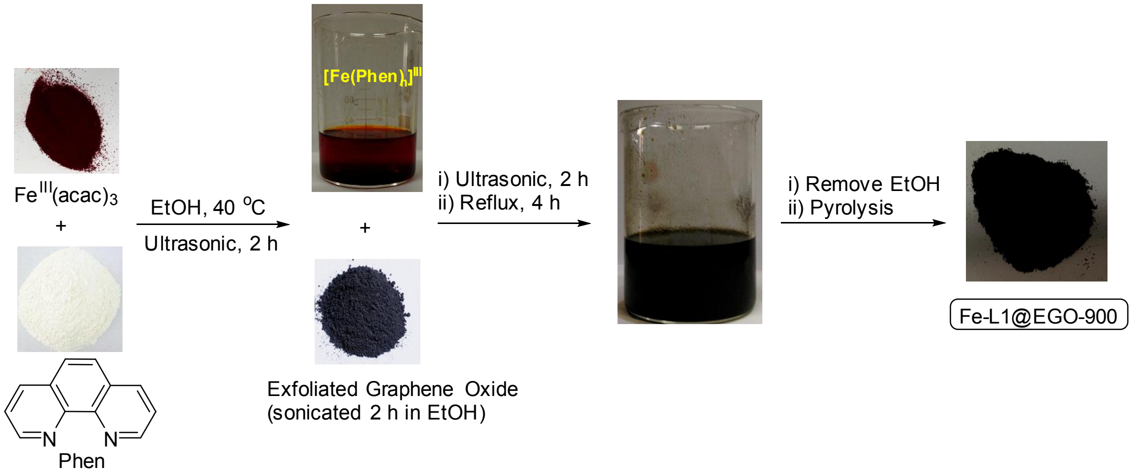

Supplementary Figure 105. Preparation of Fe-L1@EGO-900 catalyst

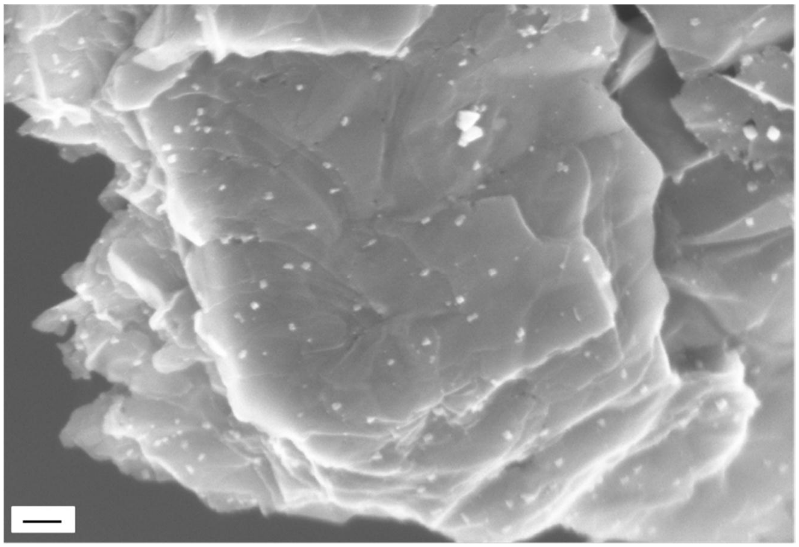

Supplementary Figure 106. FESEM image of Fe-L1@EGO-900 (scale bar 100 nm).

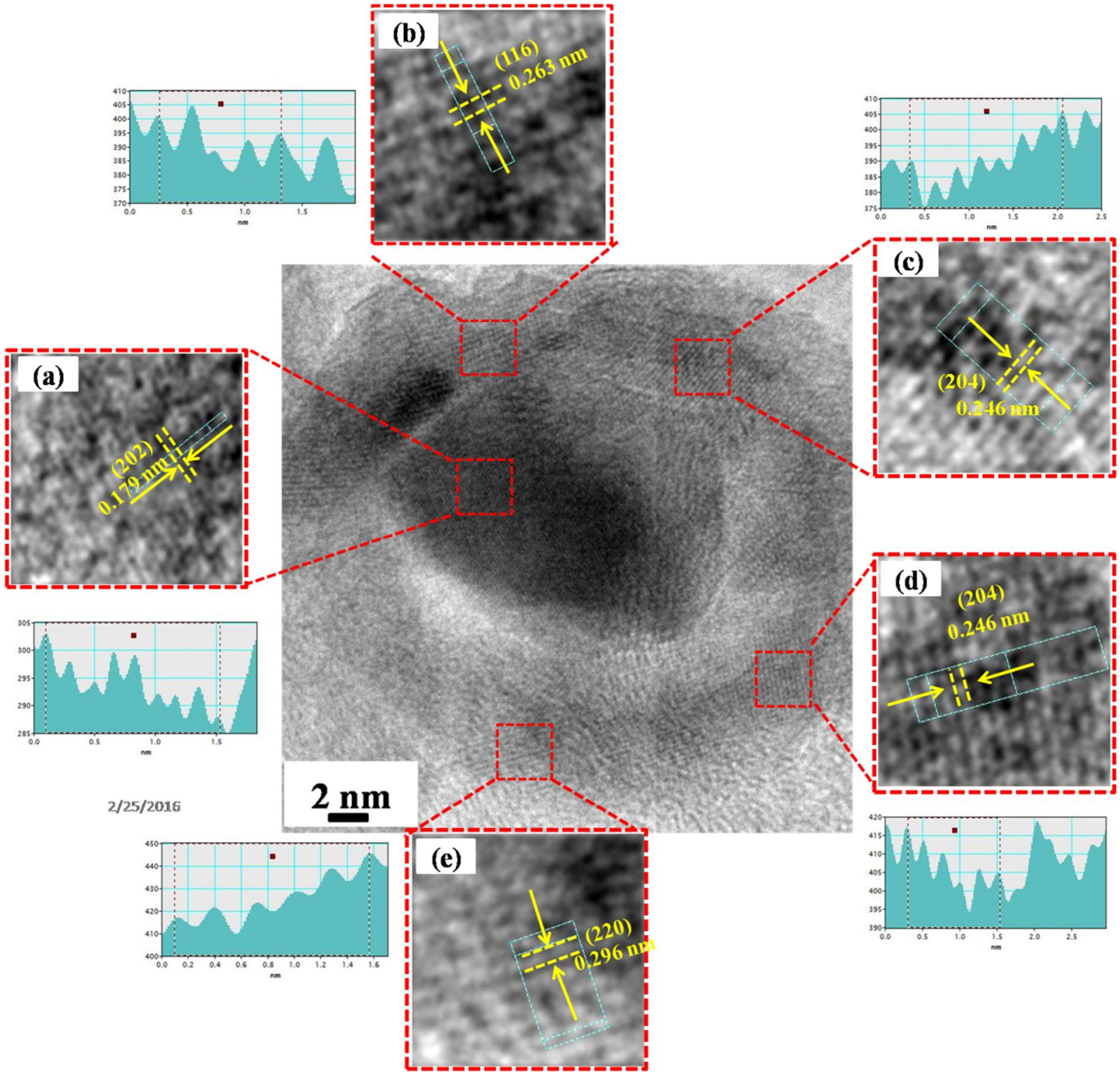

Supplementary Figure 107. HRTEM of Fe-L1@EGO-900. a, Lattice fringes of 1.79 Å corresponding to the d spacing of (202) plane of Fe_7C_3. b, Lattice fringes of 2.63 Å corresponding to the d spacing of (116) plane of Fe_3O_4 phase. c & d, Lattice fringes of 2.46 Å corresponding to the d spacing of (204) plane of β''Fe_2O_3. e, Lattice fringes of 2.96 Å corresponding to (220) plane of Fe_3O_4.

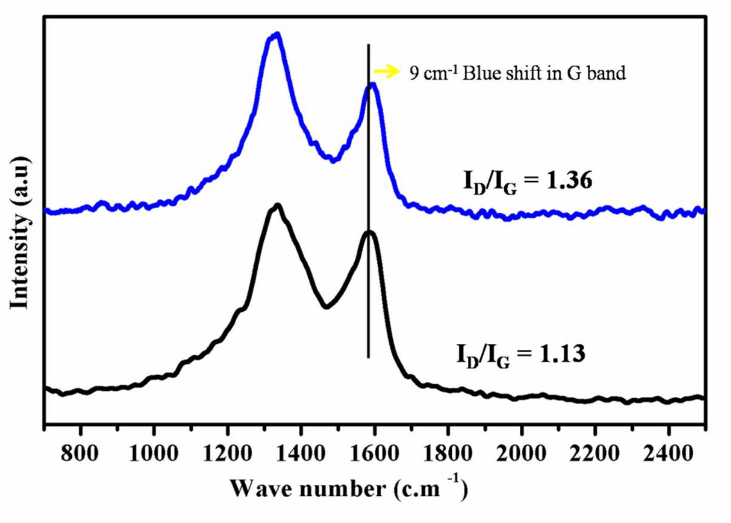

Supplementary Figure 108. Raman Spectra of reduced graphine (black) and Fe-L1@EGO-900 (Blue).

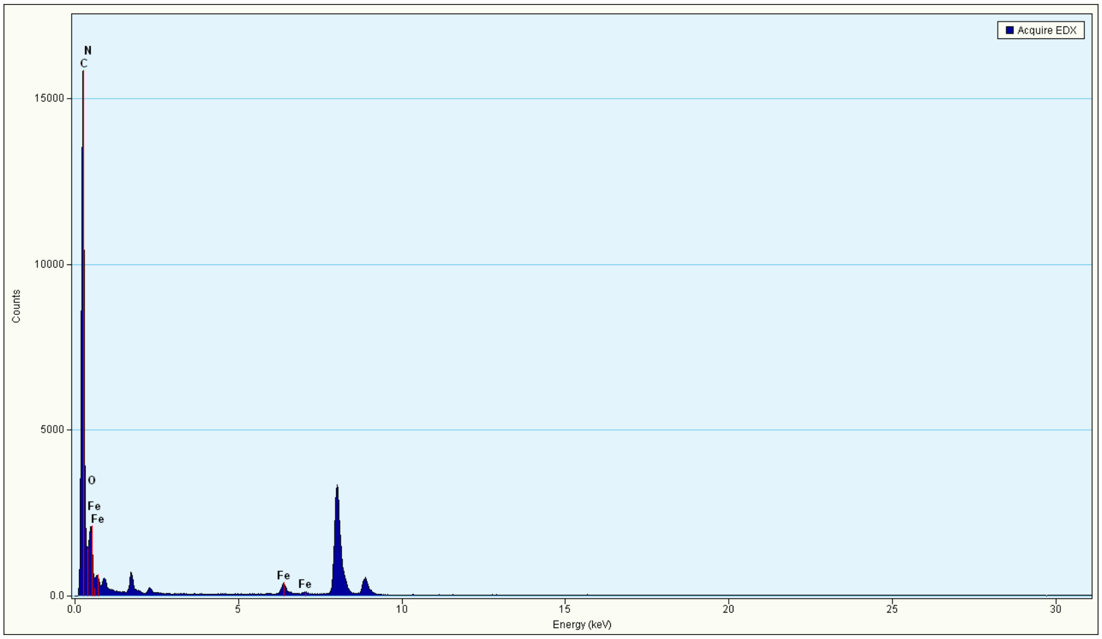

Supplementary Figure 109. EDX analysis of Fe-L1@EGO-900 catalyst.

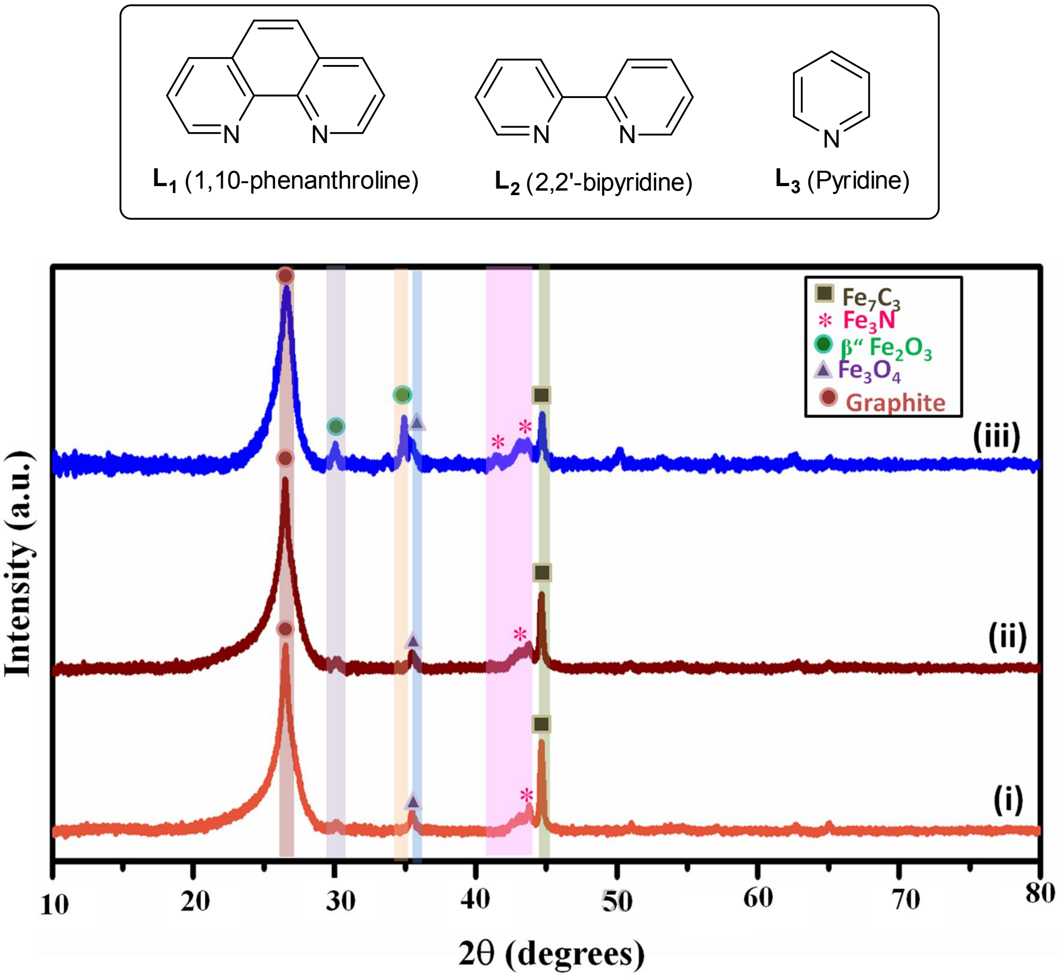

Supplementary Figure 110. (i) PXRD of Fe-L3@EGO-900, (ii) Fe-L2@EGO-900, (iii) Fe-L1@EGO-900 catalyst.

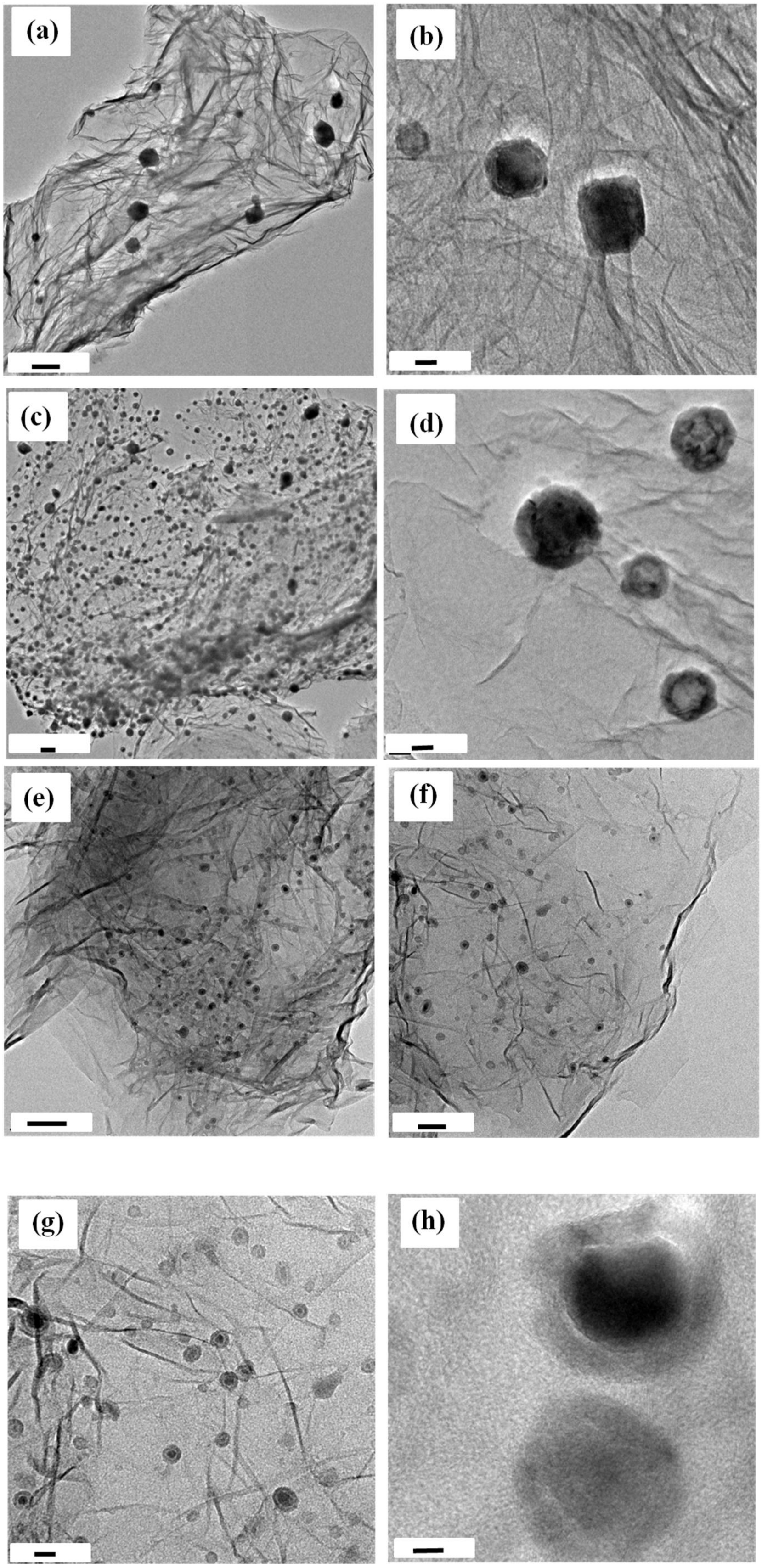
(a)
(b)
(c)
(d)
(e)
(f)
(g)
(h)

Supplementary Figure 111. a & b) TEM images of Fe-L3@EGO-900 at the scale bar of 200 nm and scale bar of 20 nm; c & d) TEM images Fe-L2@EGO-900 at the scale bar of 200 nm and the scale bar of 20 nm; e, f, g & h) TEM images of Fe-L1@EGO-900 at the scale bar of 100 nm, scale bar 50 nm, scale bar of 20 nm and scale bar of 5 nm.

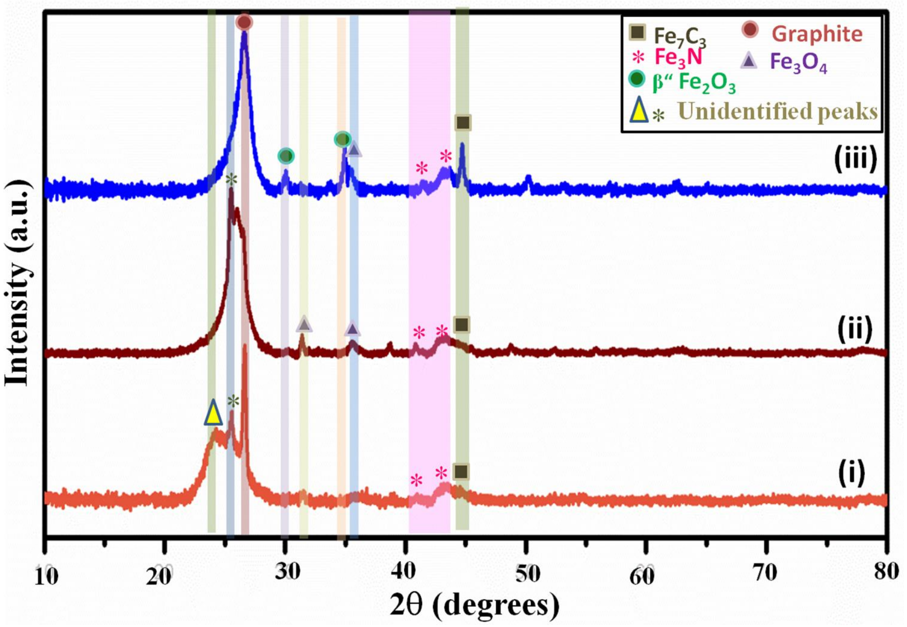

Supplementary Figure 112. (i) PXRD of Fe-L1@EGO-400, (ii) Fe-L1@EGO-600, (iii) Fe-L1@EGO-900 catalyst.

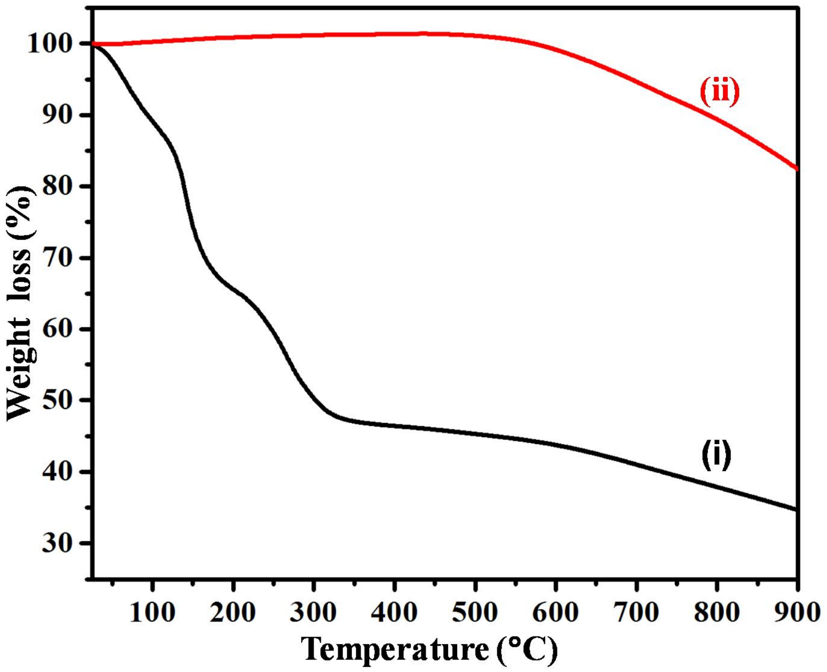

Supplementary Figure 113. TGA of exfoliated graphene oxide (black) and Fe-phenothroline complex on graphene oxide support (red).

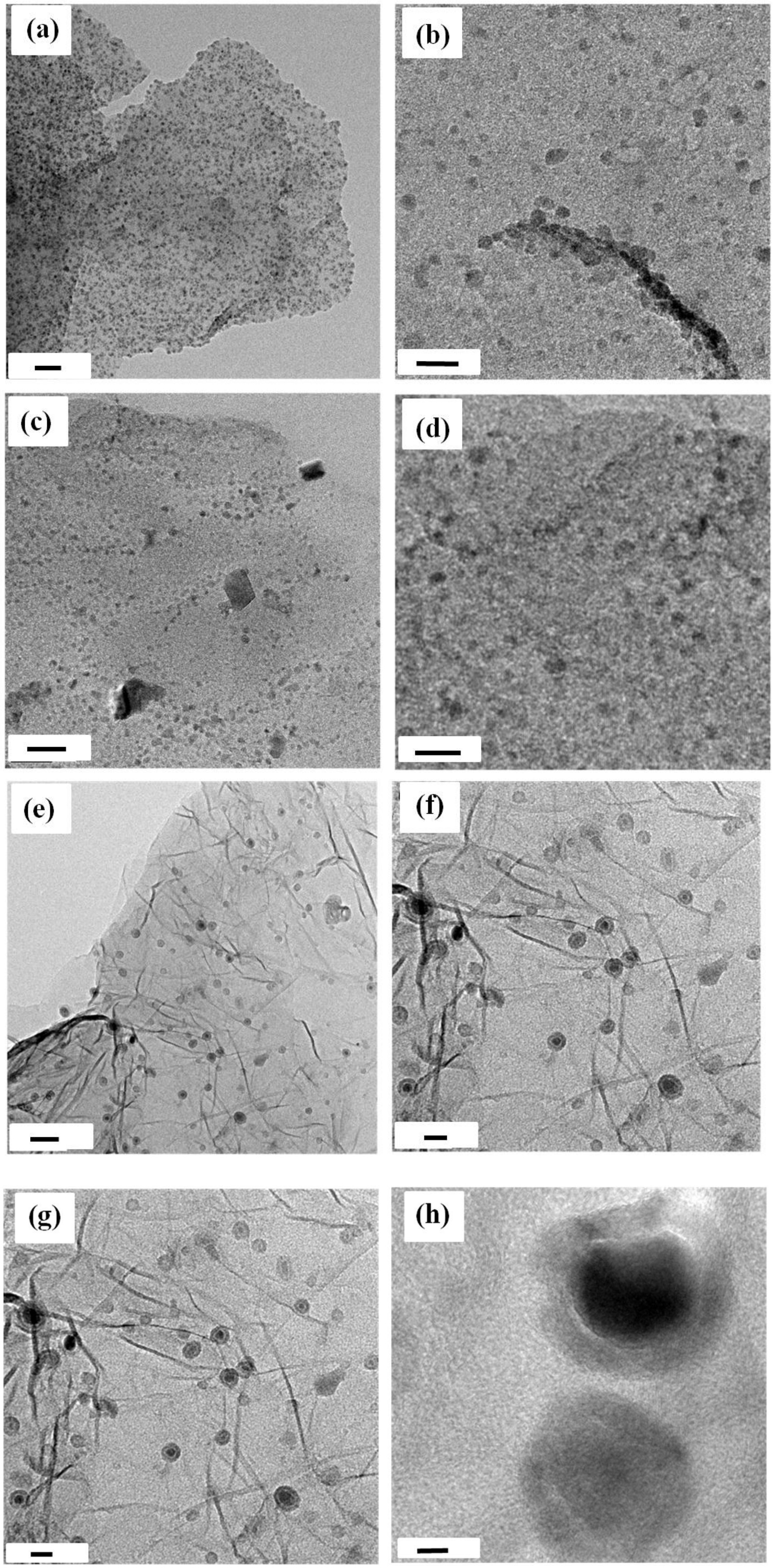
(a)
(b)
(c)
(d)
(e)
(f)
(g)
(h)

Supplementary Figure 114. a & b) TEM images of Fe-L1@EGO-400 prepared at 400 °C at the scale bar of 50 nm and scale bar of 20 nm; c & d) TEM images Fe-L1@EGO-600 prepared at 600 °C at the scale bar of 50 nm and scale bar of 20 nm; e, f, g & h) TEM images of Fe-L1@EGO-900 prepared at 900 °C at the scale bar of 50 nm, scale bar of 20 nm and scale bar of 5 nm.

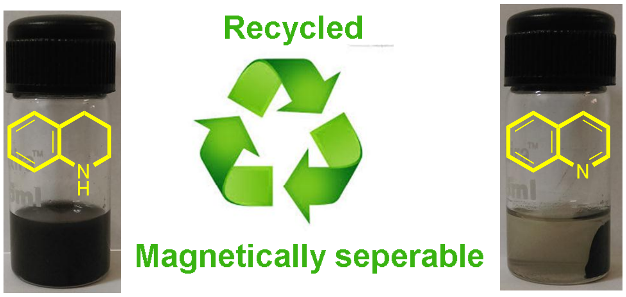

Supplementary Figure 115. Digital photograph showing a) the fine dispersion, and b) magnetic separation of Fe-L1@EGO-900.

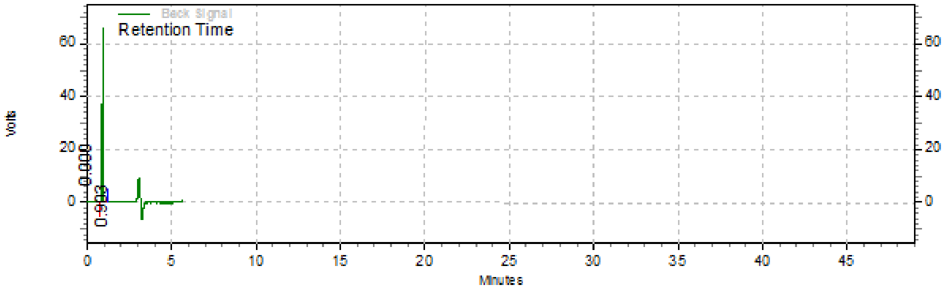

Back Signal
Results

Retention Time	Area	Area %	Height	Height %
0.000	0	0.00	0	0.00
0.903	1459661	100.00	506268	100.00
Totals	1459661	100.00	506268	100.00

Supplementary Figure 116. Gas gromatography (detection of dihydrogen).

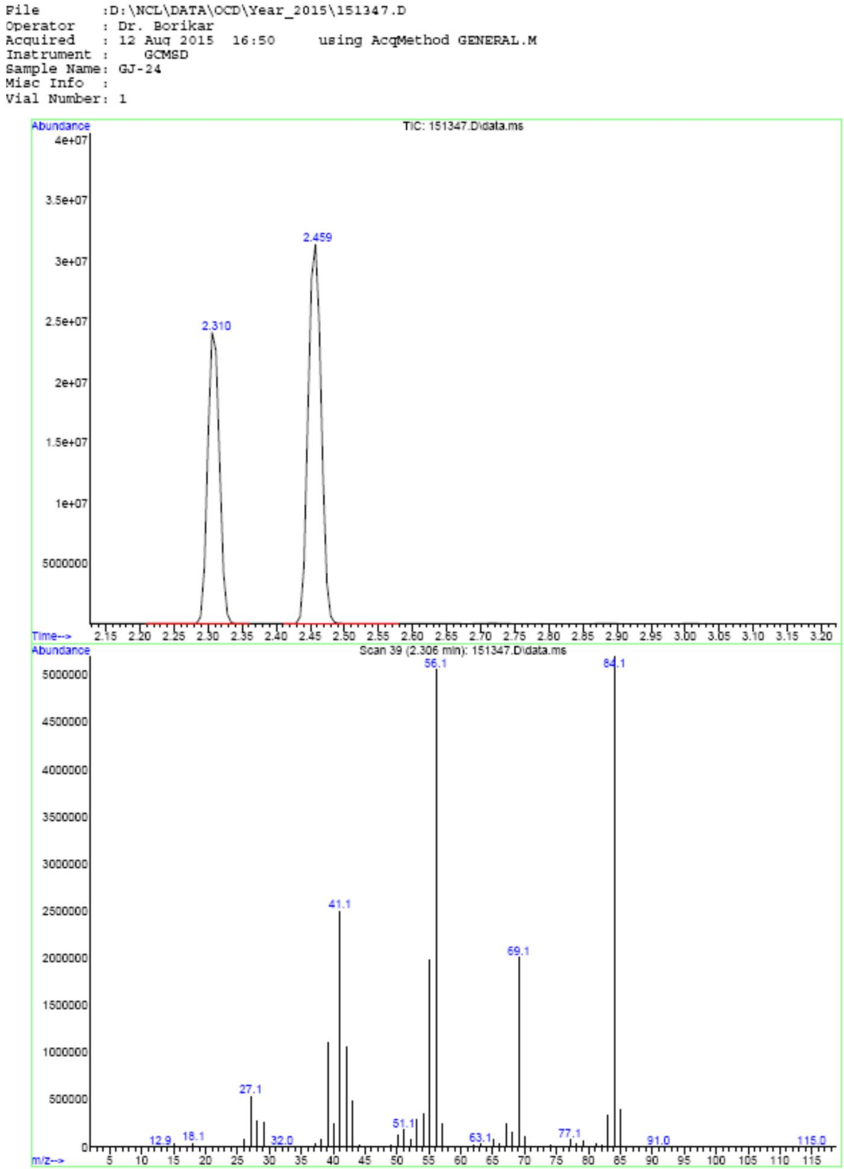

Supplementary Figure 117. GC-MS of hydrogenation of cyclohexene by *in situ* generated hydrogen gas *via* dehydrogenation of 1a.

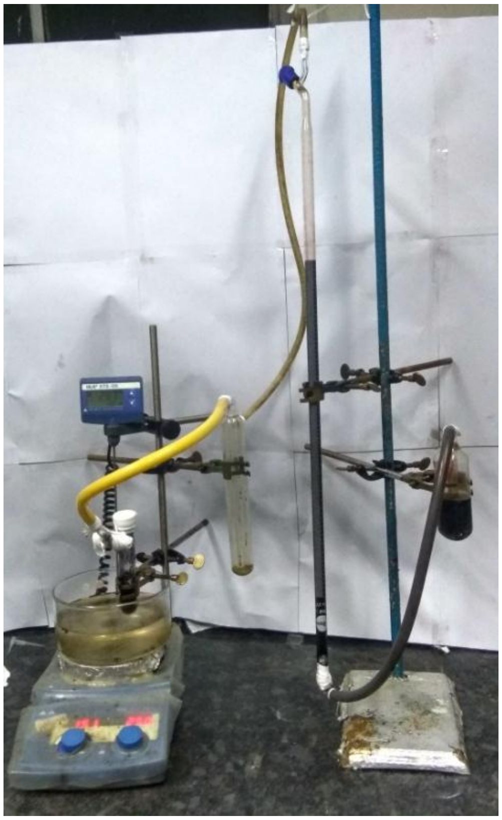

Supplementary Figure 118. Setup for hydrogen gas evolution (a volumetric quantitative analysis).

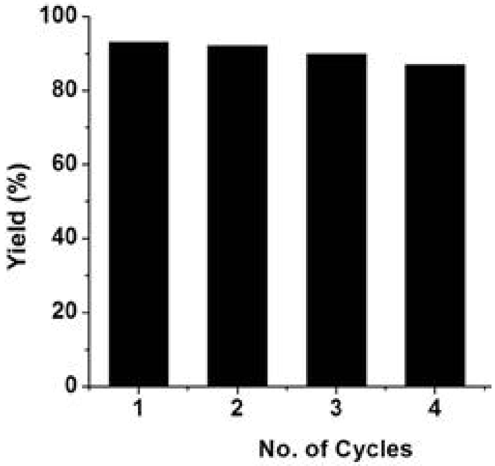

Supplementary Figure 119. Recylabilty experiments.

Supplementary Tables:

Supplementary Table 1. The weight percent of different elements in the Fe-L1@EGO-900 catalyst (from EDAX analysis)

Element	Weight %	Atomic %	Uncert. %	Correction	k-Factor
C(K)	86.67	92.80	0.68	0.26	3.940
N(K)	2.01	1.84	0.12	0.26	3.826
O(K)	4.78	3.84	0.07	0.49	1.974
Fe(K)	6.53	1.50	0.09	0.99	1.403

Supplementary Table 2. Conversion of 2-chlorobenzyl alcohol (**8c**) and selectivity of 2-chlorobenzaldehyde (**9c**) using different Fe-based catalysts in pure phases.

Entry	Catalyst	Conversion (%)*	Selectivity (%)*
1	Fe-L1@EGO-900	95	93
2	Fe_3O_4	15	55
3	Fe_2O_3	38	40
4	Fe_xN	15	80
5	Fe_3C	12	70

* Yields are based on GC.

From Supplementary Table 2, it is clear that the Fe-L1@EGO-900 with mixed phases in core-shell morphology shows excellent conversion and selectivity. Other pure distinct phases of iron are not as active for this catalysis. Hence, the mixed phases of iron having specific core-shell morphology are necessary for the superior activity.

Supplementary Methods:

General Information

All catalytic experiments were carried out using standard Schlenk techniques. All solvents were reagent grade or better. Deuterated solvents were used as received without any additional purification. Most of the chemicals used in catalysis reactions were purified according to standard procedure (or by vacuum distillation/sublimation).[1] Thin layer chromatography (TLC) was performed using silica gel precoated glass plates, which were visualized with visualized with UV light at 254 nm or under iodine. Column chromatography was performed with SiO_2 (Silicycle Siliaflash F60 (230-400 mesh). ^{1}H NMR (400, 200 or 500 MHz), ^{13}C{^{1}H} NMR (100 MHz) spectra were recorded on the NMR spectrometer. Deuterated chloroform was used as the solvent, and chemical shift values (ŭ) are reported in parts per million relatives to the residual signals of this solvent [ŭ 7.27 for ^{1}H (chloroform-d), ŭ 77.0 for ^{13}C{^{1}H} (chloroform-d). Abbreviations used in the NMR follow-up experiments: br, broad; s, singlet; d, doublet; t, triplet; q, quartet; m, multiplet. GC analysis was carried out using an HP-5 column (30 m, 0.25 mm, 0.25ε). Mass spectra were obtained on a GCMS-QP 5000 instruments with ionization voltages of 70 eV. High-resolution mass spectra (HRMS) were obtained on a High-resolution mass spectra (HRMS) were obtained by fast atom bombardment (FAB) using a double focusing magnetic sector mass spectrometer and electron impact (EI) ionization technique (magnetic sector-electric sector double focusing mass analyzer).

Materials and General Analytical Methods:

PXRD: Powder XRD samples were analysed on an Xpert Pro model PANalytical diffractometer from Philips PANalytical XôPRET PRO instruments operated at a voltage of

40 kV and a current of 30 mA with Cu Kα radiation (λ=1.5406 Å). The samples were scanned in a 2ď range from 10° to 80° with a scan rate of 0.39° per minute.

TEM: Samples dissolved in ethanol were drop cast onto separate 200 mesh carbon coated copper grids and studied using a transmission electron microscope (TEM, FEI model TECNAI G2 F20) operating at an accelerating voltage of 200 kV.

Electron Dispersive X-ray Analysis: Energy dispersive X-ray analysis (EDX) measurements on the Iron supported graphene sample was Energy dispersive X-ray analysis (EDX) measurement on active catalyst was performed using transmission electron microscope (TEM, FEI model TECNAI G2 F20) operating at an accelerating voltage of 200 kV.

ICP analysis: Inductively couple plasma atomic emission spectroscopy (ICP-AES) were acquired for the elemental analysis of absolute iron content within the sample. Analysis performed by SPECTRO analytical instruments GmbH, model ARCOS simultaneous ICP spectrometer, Germany.

Raman analysis: LabRam spectrometer (HJY, France) was used for Raman analysis with a laser wavelength of 632 nm.

X-ray Photoelectron spectroscopy: XPS was done on a VG Microtech Multilab ESCA 3000 spectrometer that was equipped with a Mg KŬ X-ray source ($h\upsilon$ = 1253.6 eV). The XPS peaks were fitted on XPSPEAK 4.1 having 70% Gaussian and 30% Lorentzian character,

after performing a Shirley background subtraction. In the fitting procedure, the full width at half-maximum (FWHM) values were fixed 1.5 eV for all the peaks.

EPR: EPR study was performed on EOL, Japan having JES - FA200 model with X and Q band in which EStandard Frequency (X band) - 8.75-9.65 GHz.

STEM and EDX elemental Analysis: HAADF-STEM images were captured on a UHR FEG-TEM, JEOL JEM-2100F electron microscope using a 200 kV electron source.

Catalyst Synthesis:

The modified Hummers method was followed to synthesize graphene oxide from graphite powder.[2] Graphitic Oxide was heated at 160 ^{o}C for 12 h for exfoliation to get exfoliated graphene oxide (EGO).

In a 100 ml beaker Fe(III)acetylacetonate precursor (0.5 mmol) and 1,10-phenanthroline ligand (0.5 mmol) were dissolved in 30 mL of ethanol and sonicated for 2 h to form Fe-phenonthroline complex. In another 250 mL beaker 560 mg of EGO support was taken in 70 mL of ethanol and sonicated for 2 h. The above obtained EGO suspension and Fe-phenanthroline complex solution were mixed together in 250 mL beaker and further sonicated for 2 h. The suspension was refluxed at 85 ^{o}C for 4 h and after cooling down to room temperature ethanol was evaporated in vacuum. The solid sample obtained was dried at 80 ^{o}C for 14 h. Then, it was ground to a fine powder followed by calcination at 900 ^{o}C under a stream of argon with the flow rate of 30 mL/min and the heating rate: 25 ^{o}C/min for about 4 h to obtain a catalyst Fe-L1@EGO-900 (Supplementry Figure 105). ICP-AES analysis was done to determine amount of Iron present and was found to be 5.32%.

For the synthesis of other conventional based supports (SiO_2,TiO_2, Al_2O_3) synthesis of $FeNSiO_2$, $FeNTiO_2$ and $FeNAl_2O_3$ catalysts was done using 560 mg of the respective support. Other steps in the synthesis were identical as per explained in the synthesis of Fe-L1@EGO-900.

Supplementary Discussion:

Characterization of catalysts

FESEM image in Supplementary Figure 106 shows the morphology of the as-prepared Fe-L1@EGO-900. In the dark field, FE-SEM of Fe-L1@EGO-900 was taken using secondary electrons in which metal has high surface electrons density appears brighter than RGO. It can be observed that Fe-rich particles are distributed spatially apart on the graphene layers.

Raman spectra of Fe-L1@EGO-900 and reduced graphine (RG) is shown in Supplementary Figure 108. It shows two characteristic peaks. The first characteristic D band was seen at 1327 cm^{-1} for Fe-L1@EGO-900 and 1330 cm^{-1} for RG arises due to vibration mode of A_{1g} symmetry of the sp^2 carbon of graphite lattice. It characterizes structural defects or edges that can break the symmetry and selection rule. The second characteristic G was located at 1595 cm^{-1} for Fe-L1@EGO-900 and at 1586 cm^{-1} for RG band that appears due to the first-order scattering of the E_{2g} observed for sp^2 carbon domains. G band represents the highly ordered graphite carbon materials. I_D/I_G increased to 1.36 in Fe-L1@EGO-900 compared to I_D/I_G=1.13 in RG which may be due to increased in disorderliness of graphene due to deposition of iron nanoparticles in Fe-L1@EGO-900 sample. G band showed a blue shift of 9 cm^{-1} in G band showed of Fe-L1@EGO-900 seen as compared to RG which is due to charge transfer from graphene to iron nanoparticles.[3-4]

Supplementary Figure 113 trace (i) shows TGA curve of exfoliated graphene oxide prepared by Hummers method that was used as the starting material to prepare our catalyst. A weight loss at 150 ^{o}C was due t to loss of physisorbed moisture. The weight loss at 350 ^{o}C is characteristic of the loss of oxygen functional groups that are present on the surface of graphitic oxide in the form of epoxide, alcohol and carbonyl groups. In comparison to trace (i), trace (ii) did not show any weight loss at 150 and 350 ^{o}C. Trace (ii) is graphitic oxide loaded with Fe-phenonthroline complex. The percentage weight loss due to loss of functional groups is not seen due to the presence of relatively heavier Fe element. In addition, Fe is also oxidized to Fe_2O_3 and Fe_3O_4.

General procedure for the acceptorless dehydrogenation of *N*-heterocycles

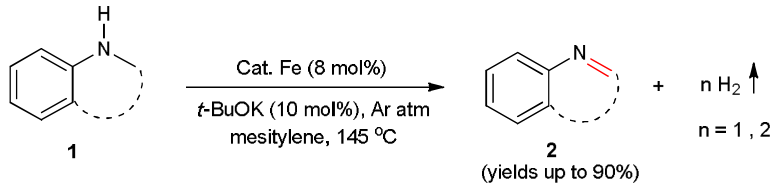

To a oven dried schlenk tube (25 mL), Fe-L1@EGO-900 catalyst (43 mg, 8 mol%), *t*-BuOK (10 mol%), *N*-heterocycles (0.5 mmol), mesitylene (2 mL) were added under argon atmosphere. The solution was heated at 145 ^{o}C with stirring under open argon flow for 18-24 h. After cooling down the reaction mixture to room temperature the catalyst was separated from the reaction mixture by centrifugation and the reaction mixture was analyzed by GC and GC-MS. The supernatant was transferred into another flask, and the catalyst was washed with EtOAc (2 x 4 mL) and the washings were collected. The solvent was evaporated from the reaction mixture, and the crude product was subjected to silica gel column chromatography using EtOAc : petroleum ether to afford the product.

General procedure for the acceptorless dehydrogenation of amines to imines

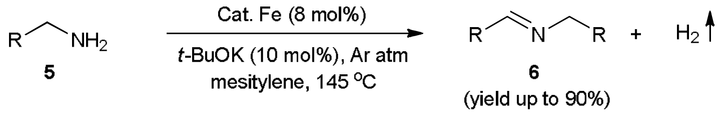

To a oven dried schlenk tube (25 mL), Fe-L1@EGO-900 catalyst (43 mg, 8 mol%), *t*-BuOK (10 mol%), an amine (0.5 mmol), mesitylene (2 mL) were added under argon atmosphere. The solution was heated at 145 ^{o}C with stirring under open argon flow for 16-24 h. After cooling down the reaction mixture to room temperature the catalyst was separated from the reaction mixture by centrifugation and the reaction mixture was analyzed by GC and GC-MS. The supernatant was transferred into another flask, and the catalyst was washed with EtOAc (2 x 4 mL) and the washings were collected. The solvent was evaporated from the reaction mixture, and the crude product was subjected to silica gel column chromatography using EtOAc : petroleum ether to afford the product.

General procedure for the iron-catalyzed alcohol dehydrogenation

To an oven dried schlenk tube (25 mL), Fe-L1@EGO-900 catalyst (43 mg, 8 mol%), *t*-BuOK (10 mol%), alcohol (0.5 mmol), n-octane (2 mL) were added under argon atmosphere. The solution was refluxed with stirring under open argon flow for 16-24 h. After cooling down the reaction mixture to room temperature the catalyst was separated from the reaction mixture by centrifugation and the reaction mixture was analyzed by GC and GC-MS. The supernatant was transferred into another flask, and the catalyst was washed with EtOAc (2 x 4 mL) and the washings were collected. The solvent was evaporated from the reaction mixture, and the crude product was subjected to silica gel column chromatography using EtOAc : petroleum ether to afford the corresponding carbony compound.

Application in synthesis of precursor (4) for nM5- lipoxygenase inhibitor[5]

Synthesis of 4

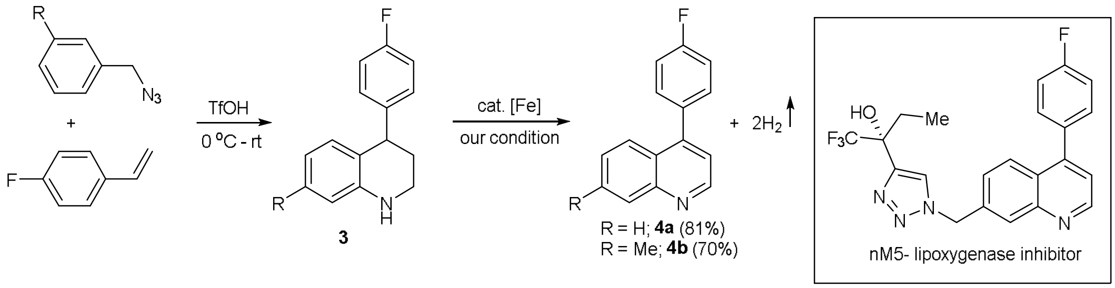

To a oven dried schlenk tube (25 mL), Fe-L1@EGO-900 catalyst (17 mg, 8 mol%), *t*-BuOK (10 mol%), **3**[5] (0.2 mmol), mesitylene (1 mL) were added under argon atmosphere. The solution was heated at 145 °C with stirring under open argon flow for 24 h. After cooling down the reaction mixture to room temperature the catalyst was separated from the reaction mixture by centrifugation. The supernatant was transferred into another flask, and the catalyst was washed with EtOAc (2 x 4 mL) and the washings were collected. The solvent was evaporated from the reaction mixture, and the crude product was subjected to silica gel column chromatography using EtOAc : petroleum ether to afford the product **4a** in 81%.

Magnetic separation of catalyst

The presence of Fe_3O_4 in the catalyst was exploited to magnetically separate them after the reaction. In the Supplementary Figure 115 (a), digital photograph show the catalyst in highly dispersed form during the reaction. After the reaction was completed, the catalysts were easily separated using a permanent magnet as shown in Supplementary Figure 115(b). Efficient magnetic separation of the catalyst was possible even after several cycles of reaction suggesting no phase transformation or leaching of the magnetic phase Fe_3O_4. This property of the catalyst is a significant advantage compared to the homogeneous and heterogeneous catalysts which are known to catalyze this reaction.

Determination of hydrogen gas formation

Qualitative analysis of hydrogen gas formation

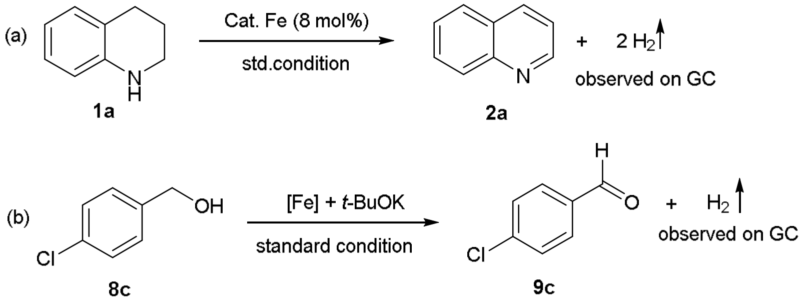

Under standard conditions the dehydrogenation of 1,2,3,4-tetrahydroquinaldine (**1a**), and (4-chlorophenyl)methanol (**8c**) were carried out indepentently using the J. Young NMR tube. After 24 h, the gas was also collected by a gas-tight syringe and qualitatively analyzed by GC-TCD with a Carbon plot capillary column gas chromatography which showed the presence of H_2 gas at retention time 0.903 (Supplementary Figure 116).

Quantitative analysis of hydrogen gas by dual catalysis

I For the detection of hydrogen dual reaction involving dehydrogenation of 1,2,3,4-tetrahydroquinoline and hydrogenation of cyclohexene was peformed.

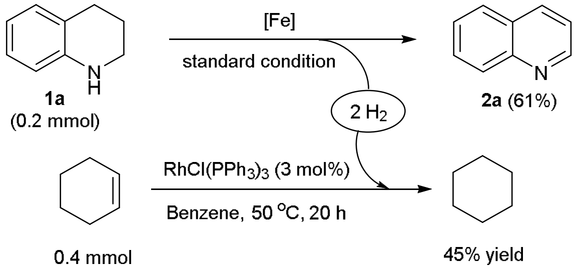

To schlenk tube, Fe-L1@EGO-900 catalyst (8 mol%), *t*-BuOK (10 mol%), 1,2,3,4-tetrahydroquinoline (0.2 mmol), mesitylene (2 mL) were added under argon atmosphere. The entire system was degassed and flushed with argon for 5 minutes (three times) and packed

with rubber septum. To another 25 mL Schlenk tube, $RhCl(PPh_3)_3$ (3 mol%) catalyst, and cyclohexene (0.4 mmol) were dissolved in benzene (2 mL). Both the flasks were connected through a double headed syringe and and allowed to equilibrate for 5 minutes. The mixture in the former flask was heated at 145 ^oC, while the mixture in the latter flask was stirred at 50 °C. After 12 hours, the organic entities present in the latter flask were analyzed by GC-MS (Supplementary Figure 117) which showed a clean conversion (45%) of the cyclohexene to cyclohexane (yield of **2a** = 61%).

II To an oven dried schlenk tube (25 mL), Fe-L1@EGO-900 catalyst (8 mol%), *t*-BuOK (10 mol%), cinnamyl alcohol (0.5 mmol), $RhCl(PPh_3)_3$ (3 mol%), and n-octane (2 mL) were added under argon atmosphere and packed with rubber septum. Reaction mixture was heated at 120 °C for 24 h and analyzed. In the rection mixture cinnamaldehyde (25%) as well as reduced product 3-phenylpropanal (45%), and 3-phenylpropan-1-ol (5%) with the conversion of 74% of **8q**. This result clearly confirms the *in situ* generation of hydrogen (*via* dehydrogenation of alcohol).

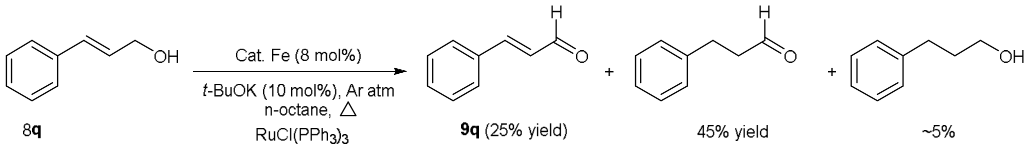

III Hydrogen gas quantification: A volumetric quantitative analysis

To an oven dried schlenk tube (25 mL), Fe-L1@EGO-900 catalyst (43 mg, 8 mol%), *t*-BuOK (10 mol%), 1,2,3,4-tetrahydroquinoline **1a** (0.4 mmol), mesitylene (2 mL) were added under argon atmosphere and packed with rubber septum. The vessel was connected to the gas

collection apparatus (standard water displacement apparatus, using a graduated cylinder to determine volume Supplementary Figure 118) and the entire system was flushed with argon for 5 minutes and allowed to equilibrate for 5 minutes. Reaction tube was palced preheated oil-bath to the appropriate temperature (145 °C). The reaction was stirred vigorously at a constant temperature until gas evolution ceased. The volume of collected gas was noted and the presence of hydrogen in the collected gas was confirmed by GC. After 24 h, the reaction mixture (contains mesitylene) was removed to give a crude product which was analyzed by ^{1}H NMR and confirmed 85% yield of the quinoline product (**2a**). The collected volume of gas in the experiment above was 19 mL, which corresponds to 0.80 mmol of dihydrogen and consisted with the release of 2 equivalents of H_2 per mole of 1,2,3,4-tetrahydroquinoline.

Reaction under presence of radical ($O\cdot^{-2}$) scavenger

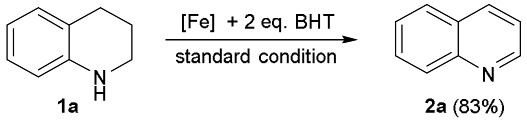

To a 25 mL oven dried schlenk tube, Fe-L1@EGO-900 catalyst catalyst (8 mol%), *t*-BuOK (10 mol%), 1,2,3,4-tetrahydroquinoline (0.5 mmol), 2,6-di-tert-butyl-4-methylphenol (BHT) (242 mg, 1.1 mmol), and n-mesiylene (2 mL) were added under argon atmosphere. The schlenk tube was equipped with a reflux condenser and the solution was refluxed under argon atmosphere for 24 h. After cooling to 30 ^{o}C the reaction mixture was subjected to centrifugation and the supernatant was collected and the obtained solid was washed with EtOAc (2 x 4 mL) and the washings were collected. The collected reaction mixture was concentrated on rotavapor under reduce pressure. The crude product was purified

(deactivated silica gel column chromatography and the eluvent is a mixture of petroleumether and ethyl acetate) and the yield of quinoline is 83%.

Hot Filtration Test

To a 25 mL oven dried schlenk tube, Fe-L1@EGO-900 catalyst (8 mol%), *t*-BuOK (10 mol%), 1,2,3,4-tetrahydroquinoline (0.5 mmol), were added under argon atmosphere. The schlenk tube was equipped with a reflux condenser, and the solution was heated at 145 ^{o}C with stirring under open argon flow for 10 h. After cooling to 30 ^{o}C the catalyst was separated from the reaction mixture by an external permanent magnet (at this stage the crude reaction mixture was analyzed by GC (43 % of **2a**). Then, the reaction mixture was transferred into another 25 mL oven dried schlenk tube under an argon atmosphere and was equipped with a reflux condenser, and the solution was heated at 145 ^{o}C with stirring under open argon flow further 12 h. After cooling to room temperature, the crude reaction mixture was quantitatively analyzed by GC and observed that no change in the yield of **2a**.

Leaching Test

To crude sample (after removal the catalyst) sulfuric acid and aqua regia were added. Then the volume of the residue was adjusted to 50 mL using water to give a sample for Inductively coupled plasma (ICP) for the measurement of the leaching of Iron and the analyses confirmed that the iron concentration in the filtrate was less than 0.24 ppm.

Recylability of the catalyst

To a oven dried schlenk tube (25 mL) Fe-L1@EGO-900 catalyst (43 mg, 8 mol%), *t*-BuOK (10 mol%), 1,2,3,4- tetrahydroqinoline (0.5 mmol), mesitylene (2 mL) were added under argon atmosphere. The solution was refluxed at 145 ^{o}C with stirring under open argon flow for 24 h. After cooling down the reaction mixture to room temperature the catalyst was separated from the reaction mixture by an external permanent magnet and washed several time with mesitylene. Obtained catalyst was dried under vacuum at 60 °C for 12 h. Then the catalyst was reused for the next cycle, and no deactivation of the material was observed up to four cycles (Supplementary Figure 119). All yields (GC) are averages from at least 2 separate runs.

Charecterization of products

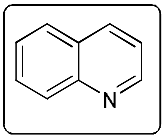

Quinoline (2a)

Compound **2a** was prepared according to the general procedure as described and was purified by silica gel column chromatography using petroleum ether/ethyl acetate. Yield: 88% (57.2 mg); ^{1}H NMR (200 MHz, $CDCl_3$) ŭ 7.27-7.79 (m, 4H), 8.09-8.15 (m, 2H), 8.90 (s, 1H); ^{13}C NMR (125 MHz, $CDCl_3$) ŭ 120.9, 126.4, 127.6, 128.1, 129.2, 135.96, 148.03, 150.173; HRMS (ESI) calculated for C_9H_7N $[M+H]^+$: 130.0651; found: 130.0651.

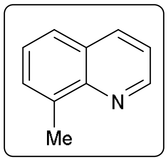

8-Methylquinoline (2b)

Compound **2b** was prepared according to the general procedure as described and was purified by silica gel column chromatography using petroleum ether/ethyl acetate. Yield: 59% (42 mg); ^{1}H NMR (500 MHz,CDCl$_3$) ŭ 2.83 (s, 3H), 7.35-7.45 (m, 2H), 7.53-7.57 (d, J = 8.0 Hz, 1H), 7.66-7.62 (d, J = 8.0 Hz, 1H), 8.08-8.12 (d, J = 8.0 Hz, 1H), 8.93-8.96 (m, 1H); ^{13}C NMR (125 MHz, CDCl$_3$) ŭ 18.0, 120.6, 125.7, 126.1, 128.0, 129.4, 136.1, 136.8, 147.1, 149.0; HRMS (ESI) calculated for $C_{10}H_9N$ $[M+H]^+$: 144.0807; found 144.0808.

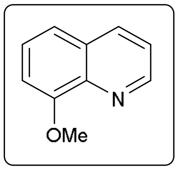

8-Methoxyquinoline (2c)

Compound **2c** was prepared according to the general procedure as described and was purified by silica gel column chromatography using petroleum ether/ethyl acetate; Yield: 61%. ^{1}H NMR (300 MHz, DMSO-d_6) ŭ: 3.96 (s, 3H), 7.17 (m, 1H), 7.51 (m, 3H), 8.29 (m, 1H), 8.84 (m, 1H). The spectral data is identical with the literature compound.[6]

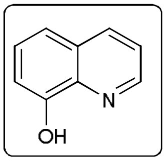

Quinolin-8-ol (2d)

Compound **2d** was prepared according to the general procedure as described and was purified by silica gel column chromatography using petroleum ether/ethyl acetate. Yield: 45% (33.5 mg); ^{1}H NMR (500 MHz, $CDCl_3$) ŭ 7.18-7.52 (m, 4H), 8.17 (dd, *J* = 8.4 Hz 1H), 8.80 (dd, *J* = 4.2 Hz *1*H); ^{13}C NMR (126 MHz, $CDCl_3$) δ 76.7, 77.3, 110.0, 117.9, 121.8, 127.7, 128.5, 136.1, 138.3, 147.9, 152.2.

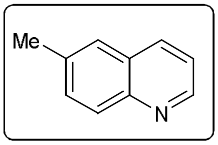

6-Methylquinoline (2e)

Compound **2e** was prepared according to the general procedure as described and was purified by silica gel column chromatography using petroleum ether/ethyl acetate. Yield: 75% (53.6 mg); ^{1}H NMR (500 MHz, $CDCl_3$) ŭ 2.47 (s, 3H), 7.26-7.29 (d, *J* = 4.2 Hz, 1H), 7.48-7.49 (m, 2H), 7.97-8.00 (t, *J* = 8.5 Hz, 2H), 7.80-8.81 (dd, *J* = 3.9 Hz, 1H); ^{13}C NMR (125 MHz, $CDCl_3$) ŭ 21.3, 120.8, 126.4, 128.1, 128.8, 131.5, 135.1, 136.1, 146.6, 149.2. HRMS (ESI) calculated for $C_{10}H_9N$ $[M+H]^+$ 144.0807; found: 144.0808.

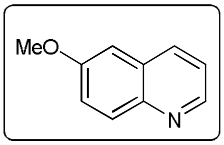

6-Methoxyquinoline (2f)

Compound **2f** was prepared according to the general procedure as described and was purified by silica gel column chromatography using petroleum ether/ethyl acetate. Yield: 87% (61.3 mg); ^{1}H NMR (500 MHz, $CDCl_3$) ŭ 3.92 (s, 3H), 7.05-7.06 (d, *J* = 2.7 Hz, 1H), 7.32-7.34 (q, *J* = 4.2 Hz, 1H), 7.35-7.38 (dd, *J* = 9.1 Hz, 1H), 7.99-8.01 (d, *J* = 9.1 Hz, 1H), 8.02-8.04 (d, *J* = 8.2 Hz, 1H), 8.75-8.76 (d, *J* = 4.2 Hz, 1H); ^{13}C NMR (125 MHz, $CDCl_3$) ŭ 55.4, 105.1,

121.3, 122.2, 129.3, 130.8, 134.7, 144.4, 147.9, 157.7; HRMS (ESI) calculated for $C_{10}H_9NO$ $[M+H]^+$: 160.0756; found: 160.0756.

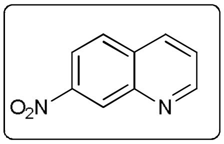

7-Nitroquinoline (2g)

Compound **2g** was prepared according to the general procedure as described and was purified by silica gel column chromatography using petroleum ether/ethyl acetate. Yield: 33% (28.7 mg); ^{1}H NMR (200 MHz, $CDCl_3$) ŭ 7.61-7.63 (q, J = 4.1Hz, 1H), 7.96-8.01 (q, J = 8.9 Hz, 1H), 8.26-8.35 (dt, J = 8.9 Hz, 2H), 9.00-9.10 (d, J = 2.1 Hz, 1H), 9.07-9.10 (dd, J = 1.6 Hz, 1H); ^{13}C NMR (125 MHz, $CDCl_3$) ŭ 120.1, 123.9, 125.8, 129.5, 131.4, 135.9, 147.1, 148.1, 152.7; HRMS (ESI) calculated for $C_9H_6N_2O_2$ $[M+H]^+$: 175.0501; found: 175.0502.

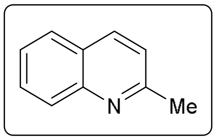

2-Methylquinoline (2h)

Compound **2h** was prepared according to the general procedure as described and was purified by silica gel column chromatography using petroleum ether/ethyl acetate. Yield: 33% (23.5 mg); ^{1}H NMR (500 MHz, $CDCl_3$) ŭ 2.70 (s, 1H), 7.18-7.20 (t, J = 8.2Hz, 1H), 7.40-7.43 (t, J = 7.9 Hz, 1H), 7.61-7.64 (t, J = 8.5Hz, 1H), 7.68-7.70 (d, J = 7.9 Hz, 1H), 7.94-7.95 (d, J = 8.5 Hz, 1H), 7.99-8.01 (d, J = 8.5 Hz, 1H); ^{13}C NMR (125 MHz, $CDCl_3$) ŭ 25.2, 121.8, 124.4, 126.3, 127.3,, 128.4, 129.2, 135.9, 147.7, 158.8; HRMS (ESI) calculated for $C_{10}H_9N$ $[M+H]^+$ 144.087144.087; found: 144.0808.

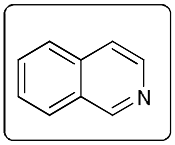

Isoquinoline (2i)

Compound **2i** was prepared according to the general procedure as described and was purified by silica gel column chromatography using petroleum ether/ethyl acetate. Yield: 76% (50 mg); ^{1}H NMR (200 MHz, $CDCl_3$) ŭ 7.63-7.95 (m, 4H), 8.09-8.13 (m, 2H), 9.24 (s, 1H); ^{13}C NMR (25 MHz, $CDCl_3$) ŭ 120.3, 126.3, 127.1, 127.5, 128.5, 130.26, 130.64, 142, 152, HRMS (ESI) calculated for C_9H_7N $[M+H]^+$: 130.0651; found: 130.0651.

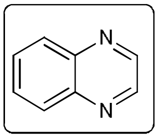

Quinoxaline (2j)

Compound **2j** was prepared according to the general procedure as described and was purified by silica gel column chromatography using petroleum ether/ethyl acetate. Yield: 69% (44.8 mg): ^{1}H NMR (500 MHz, $CDCl_3$) ŭ 7.41 (s, 2H), 7.78-7.81 (m, 2H), 8.51-8.54 (m, 2H); ^{13}C NMR (125 MHz, $CDCl_3$) ŭ 128.8, 129.3, 142.2, 144.3. HRMS (ESI) calculated for $C_8H_6N_2$ $[M+H]^+$: 131.0606; found: 131.0604.

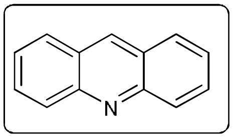

Acridine (2k)

Compound **2k** was prepared according to the general procedure as described and was purified by silica gel column chromatography using petroleum ether/ethyl acetate. Yield: 90% (81

mg); ^{1}H NMR (500 MHz, $CDCl_3$) ŭ 7.49-7.51 (q, J = 5.4 Hz, 2H), 7.74-7.78 (m, 2H), 7.94-7.97 (t, J = 8.2 Hz, 2H), 8.24-8.25 (t, J = 8.8 Hz, 2H), 8.69-8.71 (d, J = 9.7 Hz, 1H); ^{13}C NMR (125 MHz, $CDCl_3$) ŭ 125.6, 126.5, 128.1, 129.3, 130.1, 149.0.

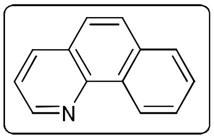

Benzo[*h*]quinoline (2l)

Compound **2l** was prepared according to the general procedure as described and was purified by silica gel column chromatography using petroleum ether/ethyl acetate. Yield: 78% (71.7 mg); ^{1}H NMR (500 MHz, $CDCl_3$) ŭ 7.49-7.51 (q, J = 4.2 Hz, 1H), 7.65-7.67 (d, J = 8.8 Hz, 1H), 7.70-7.73 (td, J = 6.7 Hz, 1H), 7.75-7.81 (m, 2H), 7.91-7.92 (d, J = 8.2 Hz, 1H), 8.13-8.15 (dd, J = 7.9 Hz, 1H), 9.02-9.03 (dd, J = 4.5 Hz, 1H), 9.34-9.36 (d, J = 7.6 Hz, 1H); ^{13}C NMR (125 MHz, $CDCl_3$) ŭ 121.6, 124.3, 125.2, 126.3, 126.9, 127.7, 128.1, 131.4, 133.5, 135.6, 146.5, 146.5, 148.7.

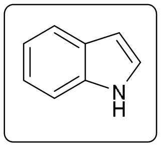

1*H*-indole (2m)

Compound **2m** was prepared according to the general procedure as described and was purified by silica gel column chromatography using petroleum ether/ethyl acetate. Yield: 88% (51.5 mg); ^{1}H NMR (200 MHz, $CDCl_3$) ŭ 6.62-6.65 (m, 1H), 7.15-7.32 (m, 3H), 7.44-7.49 (d, J = 8.2 Hz, 1H), 7.70-7.75 (d, J = 7.5 Hz, 1H), 8.19 (s, 1H); ^{13}C NMR (25 MHz, $CDCl_3$) ŭ 102.6, 110.9, 119.8, 121.9, 124.1, 127.8, 135.7.

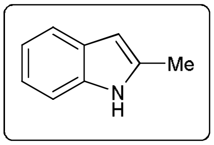

2-Methyl-1*H*-indole (2n)

Compound **2n** was prepared according to the general procedure as described and was purified by silica gel column chromatography using petroleum ether/ethyl acetate. Yield: 58% (38 mg); ^{1}H NMR (500 MHz, $CDCl_3$) ŭ 2.46 (s, 3H), 6.29 (s, 1H), 7.15-7.21 (m, 2H), 7.30-7.31 (d, J = 7.6 Hz, 1H), 7.60-7.11 (d, J = 7.6 Hz, 1H), 7.75 (s, 1H); ^{13}C NMR (125 MHz, $CDCl_3$) ŭ 13.6, 100.2, 110.2, 119.5, 120.8, 128.9, 135.1, 135.9.

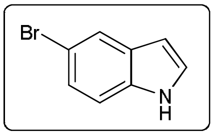

5-Bromo-1*H*-indole (2o)

Compound **2o** was prepared according to the general procedure as described and was purified by silica gel column chromatography using petroleum ether/ethyl acetate. Yield 82% (38 mg); ^{1}H NMR (500 MHz, $CDCl_3$) ŭ 6.52-6.53 (s, 1H), 7.27-7.30 (m, 3H), 7.29 (s, 1H), 8.22 (bs, 1H); ^{13}C NMR (125 MHz, $CDCl_3$) ŭ 120.3, 112.4, 113.0, 123.2, 124.8, 125.3, 129.6, 134.4.

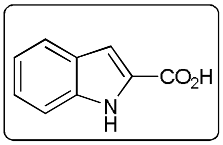

1*H*-indole-2-carboxylic acid (2p)

Compound **2p** was prepared according to the general procedure as described and was purified by silica gel column chromatography using petroleum ether/ethyl acetate. Yield 74% (60

mg); ^{1}H NMR (500 MHz, $CDCl_3$) ŭ 7.18-7.21 (t, *J* = 7.6 Hz, 1H), 7.36-7.40 (m, 2H), 7.46-7.47 (d, *J* = 8.2 Hz, 1H), 7.73-7.75 (d, *J* = 8.2 Hz, 1H), 8.97 (bs, 1H); ^{13}C NMR (125 MHz, $CDCl_3$) ŭ 110.8, 111.9, 121.1, 122.9, 126.1, 127.4, 137.3, 166.1; HRMS (ESI) calculated for $C_9H_7NO_2$ $[M+H]^+$: 162.0546; found: 162.0550.

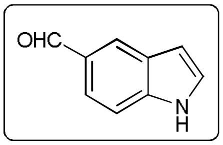

1*H*-indole-5-carbaldehyde (2q)

Compound **2q** was prepared according to the general procedure as described and was purified by silica gel column chromatography using petroleum ether/ethyl acetate. Yield 80% (58 mg); ^{1}H NMR (500 MHz, $CDCl_3$) ŭ 6.72-6.73 (s, 1H), 7.33-7.34 (t, *J* = 3.0 Hz, 1H), 7.49-7.50 (d, *J* = 8.2 Hz, 1H), 7.78-7.80 (dd, *J* = 8.5 Hz, 1H), 8.20 (s, 1H), 8.95 (bs, 1H), 10.06 (s, 1H); ^{13}C NMR (125 MHz, $CDCl_3$) ŭ 104.3, 111.8, 122.2, 126.3, 127.7, 129.6, 139.4, 192.8; HRMS (ESI) calculated for C_9H_7NO $[M+H]^+$: 146.0599; found: 146.0600.

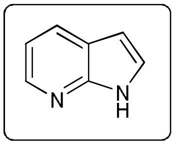

1*H*-pyrrolo[2,3-b]pyridine (2r)

Compound **2r** was prepared according to the general procedure as described and was purified by silica gel column chromatography using petroleum ether/ethyl acetate. Yield: 94% (58 mg); ^{1}H NMR (500 MHz, $CDCl_3$) ŭ 6.54-6.54 (d, *J* = 3.0 Hz, 1H), 7.11-7.14 (q, *J* = 4.8 Hz, 1H), 7.41-7.41 (q, *J* = 3.3 Hz, 1H), 8.00-8.02 (d, *J* = 7.6Hz, 1H), 8.35-8.35 (d, *J* = 4.5 Hz, 1H), ^{13}C NMR (125 MHz, CDCl3) ŭ 100.8, 115.8, 120.7, 125.4, 129.4, 141.9, 148.2.

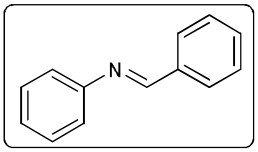

(*E*)-*N*-benzylideneaniline (2s)

Compound **2s** was prepared according to the general procedure as described and was purified by silica gel column chromatography using petroleum ether/ethyl acetate. 84% (38 mg); ^{1}H NMR (500 MHz, $CDCl_3$) ŭ 7.25-7.29 (m, 3H), 7.42-7.45 (t, *J* = 7.6Hz, 2H), 7.50-7.51 (m, 3H), 7.93-7.95 (dd, *J* = 3.6 Hz, 2H), 8.49 (s, 1H); ^{13}C NMR (125 MHz, $CDCl_3$) ŭ 120.8, 125.9, 128.7, 128.8, 129.1, 131.3, 136.1, 152.0, 160.4.

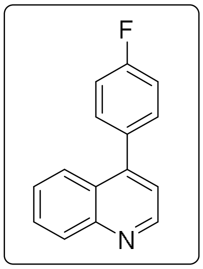

Compound **4a** was prepared according to the general procedure as described and was purified by silica gel column chromatography using petroleum ether/ethyl acetate. Yield: 81%; ^{1}H NMR (500 MHz, $CDCl_3$) ŭ = 7.29-7.16 (m, 3 H), 7.32 (d, *J* = 4.3 Hz, 1 H), 7.57 - 7.45 (m, 3 H), 7.75 (ddd, *J* = 1.2, 6.9, 8.3 Hz, 1 H), 7.98-7.83 (m, 1 H), 8.19 (d, *J* = 8.5 Hz, 1 H), 8.95 (d, *J* = 4.3 Hz, 1 H), ^{13}C NMR (125 MHz, $CDCl_3$) ŭ 115.6, 115.7, 121.4, 125.6, 126.7, 126.8, 129.4, 129.9, 131.2, 133.9, 147.4, 148.6, 149.9, 161.9, 163.9.

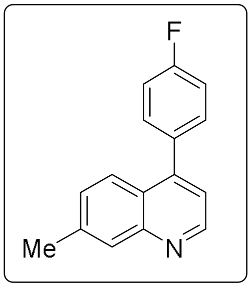

Compound **4b** was prepared according to the general procedure as described and was purified by silica gel column chromatography using petroleum ether/ethyl acetate. Yield: 70%; H NMR (500 MHz, $CDCl_3$) ŭ 2.60 (s, 3H), 7.30-7.20 (m, 3H), 7.36 (d, *J* = 4.5 Hz, 1H), 7.47-7.38 (m, 1H), 7.55-7.46 (m, 2H), 7.82 (d, *J* = 8.6 Hz, 1H), 8.15 (brs, 1H), 8.90 (m, 1H). The spectral data is identical with the literature compound.[5]

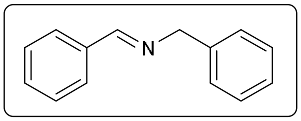

(*E*)-*N*-benzylidene-1-phenylmethanamine (6a)

Yield: 82% (41 mg); ^{1}H NMR (200 MHz, $CDCl_3$) ŭ 4.74 (s, 2H), 7.15-7.35 (m, 8H), 7.68-7.72 (m, 2H), 8.31 (s, 1H); ^{13}C NMR (125 MHz, $CDCl_3$) ŭ 65.0, 126.9, 127.9, 128.2, 128.5, 128.6, 130.7, 136.1, 139.2, 162.0; HRMS (ESI) calculated for $C_{14}H_{13}N$ $[M+H]^+$: 196.1181; found: 196.1121.

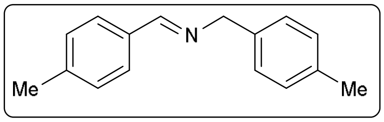

(*E*)-*N*-(4-methylbenzylidene)-1-p-tolylmethanamine (6b)

Yield: 79% (44.3 mg); ^{1}H NMR (500 MHz, $CDCl_3$) ŭ 2.35 (s, 3H), 2.39 (s, 3H), 4.78 (s, 2H), 7.15-7.17 (d, *J* = 7.9 Hz, 2H), 7.22-7.28 (d, *J* = 6.4 Hz, 4H), 7.67-7.68 (d, *J* = 7.9 Hz, 2H), 8.35 (s, 1H); ^{13}C NMR (125 MHz, $CDCl_3$) ŭ 21.1, 21.4, 64.7, 27.9, 128.2, 129.1, 129.3,

133.6, 136.3, 136.5, 140.9, 161.7; HRMS (ESI) calculated for $C_{16}H_{17}N$ $[M+H]^+$: 224.1433; found: 224.1434.

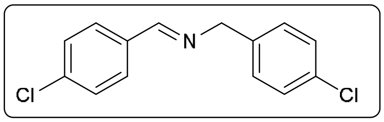

(*E*)-*N*-(4-chlorobenzylidene)-1-(4-chlorophenyl)methanamine (6c)

Yield: 86% (57 mg); ^1H NMR (200 MHz, $CDCl_3$) ŭ 4.66 (s, 2H), 7.11-7.28 (m, 6H), 7.49-7.53 (m, 1H), 7.69-7.70 (s, 1H), 8.20 (s, 1H); ^{13}C NMR (125 MHz, $CDCl_3$) ŭ 64.1, 125.9, 126.6, 127.2, 127.8, 129.9, 129.7, 129.8, 130.8, 134.3, 134.8, 137.6, 141.0, 160.8; HRMS (ESI) calculated for $C_{14}H_{11}Cl_2N$ $[M+H]^+$: 264.0338; found: 264.0341.

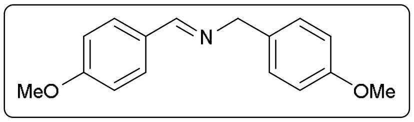

(*E*)-*N*-(4-methoxybenzylidene)-1-(4-methoxyphenyl)methanamine (6d)

Yield: 76% (48.5 mg); ^1H NMR (500 MHz, $CDCl_3$) ŭ 3.69 (s, 3H), 3.72 (s, 3H), 4.63 (s, 2H), 6.76-6.84 (m, 4H), 7.15 (d, J = 8.6 Hz, 4H), 7.62 (d, J = 8.6 Hz, 2H), 8.19 (s, 1H); ^{13}C NMR (125 MHz, $CDCl_3$) ŭ 55.2, 55.2, 64.3, 113.8, 113.9, 129.1, 129.7, 131.6, 158.5, 160.8, 161.6. HRMS (ESI) calculated for $C_{16}H_{17}NO_2$ $[M+H]^+$: 256.1328; found: 256.1332.

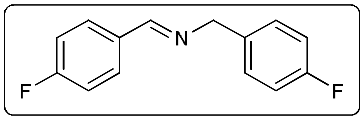

(*E*)-*N*-(4-fluorobenzylidene)-1-(4-fluorophenyl)methanamine (6e)

Yield: 90% (52 mg); ^1H NMR (500 MHz, $CDCl_3$) ŭ 4.78 (s, 2H), 7.04-7.07 (t, J = 8.5 Hz, 2H), 7.10-7.14 (t, J = 8.5 Hz, 2H), 7.30-7.33 (q, J = 5.4 Hz, 2H), 7.78-7.81 (q, J = 5.4 Hz,

2H), 8.35 (s, 1H); ^{13}C NMR (125 MHz, $CDCl_3$) ŭ 64.0, 115.1, 115.6, 115.7, 129.3, 129.4, 130.0, 130.1, 132.2, 132.3, 134.8, 134.9, 160.4, 160.9, 162.9, 163.3, 165.3; HRMS (ESI) calculated for $C_{12}H_{13}N_2$ $[M+H]^+$: 232.0943; found: 232.0932.

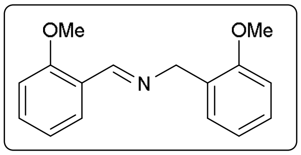

(*E*)-*N*-(2-methoxybenzylidene)-1-(2-methoxyphenyl)methanamine (6f)

Yield: 69% (44 mg); ^{1}H NMR (500 MHz, $CDCl_3$) ŭ 3.77-3.80 (m, 6H), 4.76(s, 2H), 6.78-6.94 (m, 4H), 7.13-7.31 (m, 3H), 7.94-7.98 (m, 1H), 8.77 (s, 1H); ^{13}C NMR (125 MHz, $CDCl_3$) ŭ 55.3, 55.5, 59.6, 110.1, 110.9, 120.5, 120.7, 127.5, 127.9, 129.1, 131.7, 158.3; HRMS (ESI) calculated for $C_{16}H_{17}NO_2$ $[M+H]^+$: 256.1328; found: 256.1332.

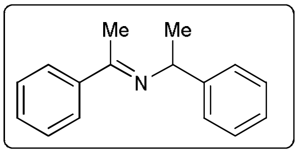

(*E*)-1-phenyl-*N*-(1-phenylethylidene)ethanamine (6h)

Yield: 71 % (40 mg); ^{1}H NMR (500 MHz, $CDCl_3$) ŭ 1.57-1.58 (m, 3H), 2.28(s, 3H), 4.85 (q, J = 6.4 Hz, 1H), 7.31-7.50 (m, 8H), 7.83-7.88 (m, 2H), 8.26 (s, 1H); HRMS (ESI) calculated for $C_{16}H_{17}N[M+H]^+$: 224.1433; found: 224.1434.

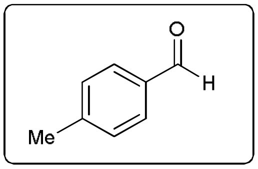

4-Methylbenzaldehyde (9a)

Yield: 91 % (54.6 mg); ^{1}H NMR (500 MHz, $CDCl_3$) ŭ 2.45 (s, 3H), 7.34-7.35 (d, *J* = 7.9 Hz, 2H), 7.78-7.80 (d, *J* = 7.9 Hz, 2H), 9.97 (s, 1H); ^{13}C NMR (125 MHz, $CDCl_3$) ŭ 21.8, 129.7, 129.8, 134.1, 145.5, 192.0.; HRMS (ESI) calculated For C_8H_8O $[M+H]^+$: 121.651; found 121.0648.

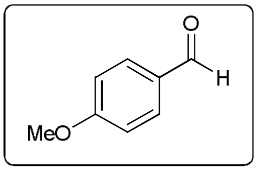

4-Methoxybenzaldehyde (9b)

Yield: 85 % (57.8 mg); ^{1}H NMR (200 MHz, $CDCl_3$) ŭ 3.87 (s, 3H), 6.96-7.01 (d, *J* = 8.8Hz, 2H), 7.80-7.84 (d, *J* = 8.8Hz, 2H), 9.86 (s, 1H); ^{13}C NMR (125 MHz, $CDCl_3$) 55.5, 114.2, 132.0, 190.9; HRMS (ESI) calculated for $C_8H_8O_2$ $[M+H]^+$: 137.0598; found: 137.0597.

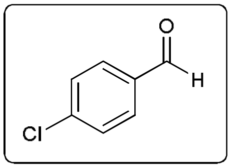

4-Chlorobenzaldehyde (9c)

Yield: 93% (65.1 mg); ^{1}H NMR (500 MHz, $CDCl_3$) ŭ 7.50-7.52 (d, *J* = 8.5 Hz, 2H), 7.81-7.83 (d, *J* = 8.5 Hz, 2H), 9.98 (s, 1H); ^{13}C NMR (125 MHz, $CDCl_3$) ŭ 129.4, 130.9, 134.6, 140.9, 190.0; HRMS (ESI) calculated for C_7H_5ClO $[M+H]^+$: 141.0103; found: 141.0102.

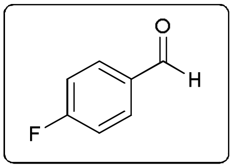

4-Fluorobenzaldehyde (9d)

Yield: 51% (31.6 mg); ^{1}H NMR (500 MHz, $CDCl_3$) ŭ 7.18-7.22 (t, *J* = 8.5Hz, 2H), 7.89-7.91 (q, *J* = 5.4 Hz, 2H), 9.95 (s, 1H); ^{13}C NMR (125 MHz, $CDCl_3$) ŭ 116.2, 116.4, 132.1, 132.2, 132.8, 132.9, 165.4, 167.5, 190.5; HRMS (ESI) calculated for C_7H_5FO $[M+H]^+$: 125.0399; found: 125.0397.

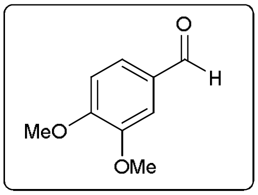

3,4-Dimethoxybenzaldehyde (9e)

Yield: 69% (57.3 mg); ^{1}H NMR (200 MHz, $CDCl_3$) ŭ 3.94 (s, 3H), 3.96 (s, 3H), 6.96-7.00 (d, *J* = 8.2 Hz, 1H), 7.40-7.48 (m, 2H), 9.85 (1H); ^{13}C NMR (125 MHz, $CDCl_3$) ŭ 55.9, 56.1, 108.7, 110.3, 126.8, 130.1, 149.5, 154.4, 190.8; HRMS (ESI) calculated for $C_9H_{10}O_3$ $[M+H]^+$: 167.0701; found: 167.0703.

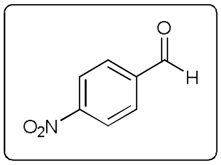

4-Nitrobenzaldehyde (9f)

Yield: 45% (34 mg); ^{1}H NMR (200 MHz, $CDCl_3$) ŭ 8.05-8.10 (d, *J* = 8.7 Hz, 2H), 8.38-8.41 (d, *J* = 8.7 Hz, 2H), 10.16 (s, 1H); ^{13}C NMR (25 MHz, $CDCl_3$) ŭ 124.2, 130.4, 139.9, 151.0, 190.2.

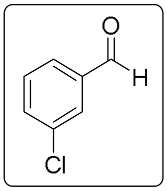

3-Chlorobenzaldehyde (9g)

Yield: 94%; ^{1}H NMR (400 MHz, $CDCl_3$): ŭ 7.49 (t, J = 7.9 Hz, 1H), 7.61 (m, 1H), 7.77 (dt, J = 7.9 Hz, 1.3 Hz, 1H), 7.86 (t, J = 2.0 Hz, 1H), 9.98 (s, 1H). ^{13}C NMR (125 MHz, $CDCl_3$): ŭ 127.9, 129.3, 130.3, 134.4, 135.4, 137.8, 190.8.

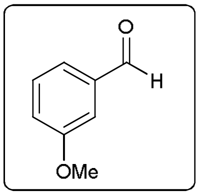

3-Methoxybenzaldehyde (9h)

Yield: 78% (53 mg); ^{1}H NMR (200 MHz, $CDCl_3$) ŭ 3.85 (s, 3H), 7.14-7.19 (m, 1H), 7.37-7.38 (t, J = 2.2 Hz, 1H), 7.42-7.45 (m, 2H), 9.86 (s, 1H); ^{13}C NMR (125 MHz, $CDCl_3$) ŭ 55.4, 112.0, 121.4, 123.5, 129.9, 137.7, 160.1, 192.1.

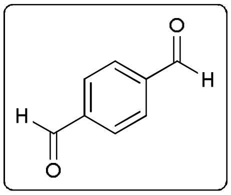

Terephthalaldehyde 9i

Yield: 70% (47 mg); ^{1}H NMR (200 MHz, $CDCl_3$) ŭ 8.05 (s, 4H), 10.12 (s, 2H); ^{13}C NMR (125 MHz, $CDCl_3$) ŭ 130.1, 139.9, 191.5; HRMS (ESI) calculated for $C_8H_6O_2$ $[M+H]^+$: 135.0440; found: 135.0441.

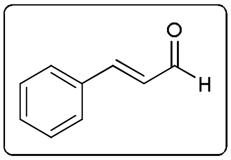

Cinnamaldehyde (9j)

Yield: 69 % (42 mg); ^{1}H NMR (500 MHz, $CDCl_3$) ŭ 7.69-7.74 (q, *J* = 7.6 Hz, 1H), 7.43-7.49 (m, 4H), 7.56-7.58 (m, 2H), 9.70-9.71 (t, *J* = 7.6 Hz, 1H); ^{13}C NMR (125 MHz, $CDCl_3$) ŭ 128.4, 129.0, 131.1, 133.9, 152.7, 193.6.

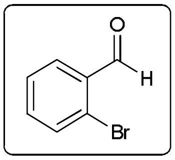

2-Bromobenzaldehyde (9k)

Yield: 71% (65 mg); ^{1}H NMR (200 MHz, $CDCl_3$) ŭ 7.39-7.49 (m, 3H), 7.60-7.65 (m, 1H), 7.86-7.92 (m, 1H), 10.33 (s, 1H); ^{13}C NMR (125 MHz, $CDCl_3$) ŭ 127.8, 129.7, 133.7, 135.2, 191.7; HRMS (ESI) calculated for C_7H_5BrO $[M+H]^+$: 184.9595; found: 184.9597.

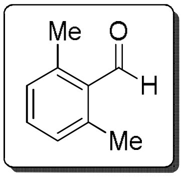

2,6-Dimethylbenzaldehyde (9l)

Yield: 52%. ^{1}H NMR (200 MHz, $CDCl_3$) ŭ 2.62 (s, 6H). 7.10 (d, *J* = 7.5 Hz, 2H), 7.33 (t, *J* = 7.5 Hz, 1H), 10.64 (s, 1H), ^{13}C NMR (125 MHz, $CDCl_3$) ŭ 20.7, 129.9, 132.7, 133.2, 141.3, 193.8.

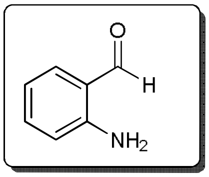

2-Aminobenzaldehyde (9m)

^{1}H NMR (400 MHz, $CDCl_3$): ŭ 9.86 (s, 1H), 7.46 (d, *J* = 7.2 Hz, 1H), 7.30 (t, *J* = 6.8 Hz, 1H), 6.73 (t, *J* = 6.6 Hz, 1H), 6.64 (d, *J* = 8.0 Hz, 1H), 6.13 (s, 2H). ^{13}C NMR (125 MHz, $CDCl_3$): ŭ 116.1, 116.4, 118.9, 135.2, 135.7, 150.0, 194.1.

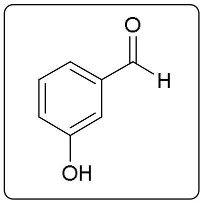

3-Hydroxybenzaldehyde (9n)

Yield: 61% (37 mg); ^{1}H NMR (200 MHz, $CDCl_3$) ŭ 6.42 (s, 1H), 7.14-7.20 (m, 1H) 7.40-7.46 (m, 3H), 9.95 (s, 1H); ^{13}C NMR (125 MHz, $CDCl_3$) ŭ 114.7, 122.2, 123.5, 130.4, 137.7, 156.5, 192.7; HRMS (ESI) calculated for $C_7H_6O_2$ $[M+H]^+$: 123.0443; found: 123.0441.

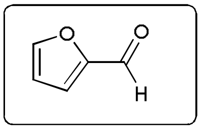

Furan-2-carbaldehyde (9o)

Yield: 55% (26.5 mg); ^{1}H NMR (200 MHz, $CDCl_3$) ŭ 6.61-6.64 (q, *J* = 1.6 Hz, 1H), 7.27-7.29 (q, *J* = 3.6 Hz, 1H), 7.71-7.72 (t, *J* = 0.7 Hz, 1H), 9.67 (s, 1H); ^{13}C NMR (125 MHz, $CDCl_3$) ŭ 112.5, 121.1, 147.9, 152.8, 177.7; HRMS (ESI) calculated for $C_5H_4O_2$ $[M+H]^+$: 97.0289; found: 97.0284.

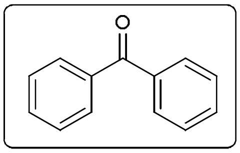

Benzophenone (11a)

Yield: 97% (88 mg); ^{1}H NMR (200 MHz, $CDCl_3$) ŭ 7.42-7.60 (m, 6H), 7.76-7.81 (m, 4H); ^{13}C NMR (125 MHz, $CDCl_3$) ŭ 128.2, 129.9, 132.3, 137.5, 196.6. HRMS (ESI) calculated for $C_{13}H_{10}O$ $[M+H]^+$: 183.0803; found: 183.0804.

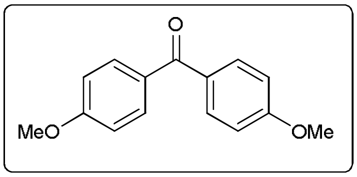

Bis(4-methoxyphenyl)methanone (11b)

Yield: 98% (118 mg); ^{1}H NMR (200 MHz, $CDCl_3$) ŭ 3.90 (s, 6H), 6.97 (d, *J* = 9 Hz, 4H), 7.80 (d, *J* = 9 Hz, 4H); ^{13}C NMR (50 MHz, $CDCl_3$) ŭ 55.5, 113.4, 130.7, 132.2, 162.8.

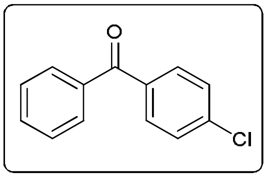

(4-Chlorophenyl)(phenyl)methanone (11c)

Yield: 95% (102 mg); ^{1}H NMR (200 MHz, $CDCl_3$) ŭ 7.45-7.54 (m, 4H), 7.58-7.66 (m, 1H), 7.75-7.80 (m, 4H); HRMS (ESI) calculated for $C_{13}H_9ClO$ $[M+H]^+$: 217.0415; found: 217.0415.

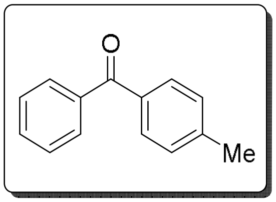

Phenyl(p-tolyl)methanone (11d)

Yield: 90% (88 mg); ^{1}H NMR (200 MHz,$CDCl_3$) ŭ 2.44 (s, 3H), 7.26-7.30 (d, *J* = 8.4 Hz, 2H), 7.43-7.62 (m, 3H), 7.70-7.81 (m, 4H); ^{13}C NMR (125 MHz, $CDCl_3$) ŭ 21.6, 128.2, 128.9, 129.9, 130.2, 132.1, 134.8, 137.9, 143.2, 196.5; HRMS (ESI) calculated for $C_{14}H_{12}O$ $[M+H]^+$: 197.0961; found: 197.0961.

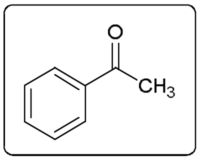

Acetophenone (11e)

Yield: 73 % (43.8 mg); ^{1}H NMR (200 MHz, $CDCl_3$) ŭ 2.60 (s, 3H), 7.42-7.56 (m, 3H), 7.93-7.88 (m, 2H); ^{13}C NMR (25 MHz, $CDCl_3$) ŭ 26.6, 128.3, 128.5, 133.0, 137.0, 198.1; HRMS (ESI) calculated for C_8H_8O $[M+H]^+$: 120.0651; found: 120.648.

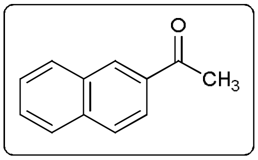

1-(Naphthalen-2-yl)ethanone (11f)

Yield: 77% (65.5 mg); ^{1}H NMR (500 MHz, $CDCl_3$) ŭ 2.72 (s, 3H), 7.54-7.62 (m, 2H), 7.86-7.89 (d, *J* = 7.3Hz, 2H), 7.95-7.97 (d, *J* = 7.9 Hz, 1H), 8.02-8.04 (d, *J* = 8.5 Hz, 1H), 8.46 (s, 1H); ^{13}C NMR (125 MHz, $CDCl_3$) ŭ 26.6, 123.8, 126.7, 127.7, 128.3, 128.4, 129.5, 130.1,

132.4, 134.3, 135.5, 198.0; HRMS (ESI) calculated for $C_{12}H_{10}O$ $[M+H]^+$: 171.804; found: 171.0804.

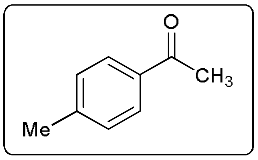

1-*p*-tolylethanone (11g)

Yield: 71% (46.8 mg); ^{1}H NMR (200 MHz,$CDCl_3$) ŭ 2.40 (s, 3H), 2.57 (s, 3H), 7.23-7.27 (d, *J* = 8.3 Hz, 2H), 7.84-7.88 (d, *J* = 8.0 Hz, 2H); ^{13}C NMR (125 MHz, $CDCl_3$) ŭ 21.5, 26.4, 128.3, 129.1, 134.6, 143.8, 197.7; HRMS (ESI) calculated for $C_9H_{10}O$ $[M+H]^+$: 135.0804; found: 135.0804.

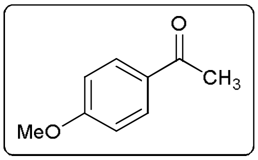

1-(4-Methoxyphenyl)ethanone (11h)

Yield: 80% (60 mg); ^{1}H NMR (200 MHz, $CDCl_3$) ŭ 2.53 (s, 3H), 3.84 (s, 3H), 6.89-6.93 (d, *J* = 8.9 Hz, 2H), 7.89-7.94 (d, *J* = 8.9 Hz, 1H); ^{13}C NMR (125 MHz, $CDCl_3$) ŭ 26.2, 55.3, 113.6, 130.2, 130.4 163.4, 196.7; HRMS (ESI) calculated for $C_9H_{10}O_2$ $[M+H]^+$: 151.0752; found: 151.0754.

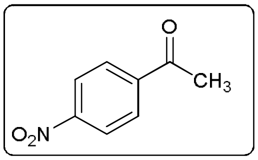

1-(4-Nitrophenyl)ethanone (11i)

Yield: 69% (57 mg); ^{1}H NMR (200 MHz, $CDCl_3$) ŭ 2.68 (s, 3H), 8.09-8.13 (t, J = 8.8 Hz, 2H), 8.29-8.33 (d, J = 8.8 Hz, 2H); ^{13}C NMR (25 MHz, $CDCl_3$) ŭ 26.9, 123.8, 129.2, 141.3, 150.3, 196.3; HRMS (ESI) calculated for $C_8H_7NO_3$ $[M+H]^+$: 166.0498; found: 166.0499.

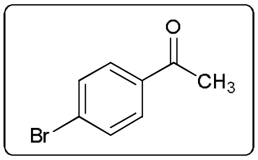

1-(4-Bromophenyl)ethanone (11j)

Yield: 72% (71 mg); ^{1}H NMR (200 MHz, $CDCl_3$) ŭ 2.56 (s, 2H), 7.55-7.59 (d, J = 8.5 Hz, 2H), 7.77-7.81 (d, J = 8.5 Hz, 2H); ^{13}C NMR (125 MHz, $CDCl_3$) ŭ 26.4, 128.1, 129.7, 131.7, 135.8, 196.8; HRMS (ESI) calculated for C_8H_7BrO $[M+H]^+$: 198.9751; found: 198.9753.

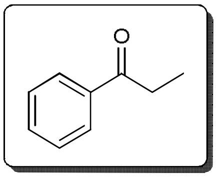

Propiophenone (11k)

Yield: 43% (29 mg); ^{1}H NMR (200 MHz, $CDCl_3$) ŭ 1.19-1.26 (t, J = 7.3 Hz, 3H), 2.95-3.05 (d, J = 7.2 Hz, 2H), 7.40-7.55 (m, 3H), 7.54-7.55 (m, 2H); ^{13}C NMR (125 MHz, $CDCl_3$) ŭ 8.1, 31.6, 127.8, 128.4, 132.7, 136.8, 200.7.

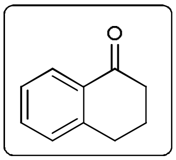

3,4-Dihydronaphthalen-1(2*H*)-one (11l)

Yield: 54% (39.5 mg); ^{1}H NMR (200 MHz, $CDCl_3$) ŭ 2.07-2.20 (m, J = 6.5 Hz, 2H), 2.63-2.69 (t, J = 6.9 Hz, 2H), 2.94-3.00 (t, J = 6.0 Hz, 2H), 7.23-7.34 (m, 2H), 7.43-7.51 (m, 1H), 8.01-8.06 (d, J = 7.8 Hz, 1H); ^{13}C NMR (125 MHz, $CDCl_3$) ŭ 23.1, 29.6, 39.1, 126.5, 127.0, 128.7, 132.5, 133.2, 144.4, 198.2; HRMS (ESI) calculated for $C_{10}H_{10}O$ $[M+H]^+$: 147.0803; found: 147.0804.

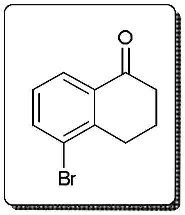

5-Bromo-3,4-dihydronaphthalen-1(2*H*)-one (11m)

Yield: 36%; ^{1}H NMR: ŭ 2.1-2.2 (m, 2H), 2.65 (m, 2H), 3.0 (t, J = 7.0 Hz, 2H), 7.15-7.25 (t, J = 6.9 Hz, 1H) 7.7 (d, J = 7.5 Hz, 1H), 8.0 (d, J = 8.0 Hz, 1H).

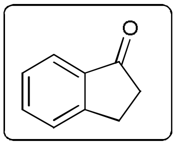

2,3-Dihydro-1*H*-inden-1-one (11n)

Yield: 51% (31.11 mg); ^{1}H NMR (200 MHz, $CDCl_3$) ŭ 2.65-2.71 (t, J = 6.2 Hz, 2H), 3.11-3.17 (t, J = 6.2 Hz, 2H), 7.36-7.40 (t, J = 7.7 Hz, 1H), 7.45-7.49 (d, J = 7.7 Hz, 1H), 7.54-7.62 (t, J = 6.9 Hz, 1H), 7.73-7.74 (d, J = 7.7 Hz, 1H); ^{13}C NMR (125 MHz, $CDCl_3$) ŭ 25.7, 36.1. 123.6, 126.6, 127.2, 134.5, 137.0, 155.1, 207.0; HRMS (ESI) calculated for C_9H_8O $[M+H]^+$: 133.0648; found: 133.0648.

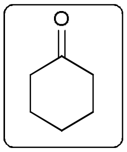

Cyclohexanone (11o)

Yield: 40% (20 mg); ^{1}H NMR (500 MHz, $CDCl_3$) ŭ 1.69-1.73 (m, 2H), 1.83-1.88 (m, 4H), 2.31-2.34 (m, 4H); ^{13}C NMR (125 MHz, $CDCl_3$) ŭ 128.2, 129.9, 132.3, 137.5, 196.6; HRMS (ESI) calculated for $C_6H_{10}O$ $[M+H]^+$: 99.0808; found: 99.0804.

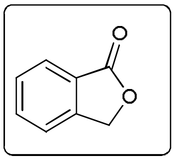

Isobenzofuran-1(3*H*)-one (13)

Yield: 97 % (65 mg); ^{1}H NMR (200 MHz, $CDCl_3$) ŭ 5.33 (s, 2H), 7.49-7.57 (t, J = 7.5 Hz, 2H), 7.65-7.73 (d, J = 7.5 Hz, 1H), 7.90-7.94 (d, J = 7.5 Hz, 1H); ^{13}C NMR (125 MHz, $CDCl_3$) ŭ 69.6, 120.0, 125.6, 128.9, 133.9, 146.5, 171.1; HRMS (ESI) calculated for C_8H_6O $[M+H]^+$: 135.0440; found: 135.0441.

Suplementary References:

1 Armarego, W. L. F. & Perrin, D. D. Purification of laboratory chemicals (Pergamon Press, Oxford, 1988) ed 3.

2 Marcano, D. C., Kosynkin, D. V., Berlin, J. M., Sinitskii, A., Sun, Z., Slesarev, A., Alemany, L. B., Lu, W. & Tour, J. M. Improved synthesis of graphene oxide. *ACS Nano* **8**, 4806-4814 (2010).

3 McCann, R., Roy, S. S., Papakonstantinou, P., McLaughlin, J. A. & Ray, S. C. Spectroscopic analysis of *a*-C and *a*-CNx films prepared by ultrafast high repetition rate pulsed laser deposition. *J. Appl. Phys*. **97**, 073522 (2005).

4 Zhou, J., Song, H., Ma, L. & Chen, X. Magnetite/graphene nanosheet composites: interfacial interaction and its impact on the durable high-rate performance in lithium-ion batteries. *RSC Adv*. **1**, 782-791 (2011).

5 Cui, X., Li, Y., Bachmann, S., Scalone, M., Surkus, A., Junge, K., Topf, C. & Beller, M. Synthesis and characterization of ironï nitrogen-doped graphene/coreï shell catalysts: fficient oxidative dehydrogenation of *N*-heterocycles. *J. Am. Chem. Soc*. 137, 10652-10658 (2015).

6. Iosub, A. V. & Stah, S. S. Catalytic aerobic dehydrogenation of nitrogen heterocycles using heterogeneous cobalt oxide supported on nitrogen-doped carbon. *Org. Lett*. **17**, 4404-4407 (2015).
